# Epigenetic loss of RNA-methyltransferase NSUN5 in glioma targets ribosomes to drive a stress adaptive translational program

**DOI:** 10.1007/s00401-019-02062-4

**Published:** 2019-08-19

**Authors:** Maxime Janin, Vanessa Ortiz-Barahona, Manuel Castro de Moura, Anna Martínez-Cardús, Pere Llinàs-Arias, Marta Soler, Daphna Nachmani, Joffrey Pelletier, Ulrike Schumann, Maria E. Calleja-Cervantes, Sebastian Moran, Sonia Guil, Alberto Bueno-Costa, David Piñeyro, Montserrat Perez-Salvia, Margalida Rosselló-Tortella, Laia Piqué, Joan J. Bech-Serra, Carolina De La Torre, August Vidal, María Martínez-Iniesta, Juan F. Martín-Tejera, Alberto Villanueva, Alexandra Arias, Isabel Cuartas, Ana M. Aransay, Andres Morales La Madrid, Angel M. Carcaboso, Vicente Santa-Maria, Jaume Mora, Agustin F. Fernandez, Mario F. Fraga, Iban Aldecoa, Leire Pedrosa, Francesc Graus, Noemi Vidal, Fina Martínez-Soler, Avelina Tortosa, Cristina Carrato, Carme Balañá, Matthew W. Boudreau, Paul J. Hergenrother, Peter Kötter, Karl-Dieter Entian, Jürgen Hench, Stephan Frank, Sheila Mansouri, Gelareh Zadeh, Pablo D. Dans, Modesto Orozco, George Thomas, Sandra Blanco, Joan Seoane, Thomas Preiss, Pier Paolo Pandolfi, Manel Esteller

**Affiliations:** 1 Cancer Epigenetics and Biology Program (PEBC), Bellvitge Biomedical Research Institute (IDIBELL), L’Hospitalet, Barcelona, Catalonia Spain; 2 Department of Medicine and Pathology, Cancer Research Institute, Beth Israel Deaconess Cancer Center, Beth Israel Deaconess Medical Center, Harvard Medical School, Boston, MA USA; 3 Molecular Mechanisms and Experimental Therapy in Oncology Program, Metabolism and Cancer Group, Bellvitge Biomedical Research Institute (IDIBELL), L’Hospitalet, Barcelona, Catalonia Spain; 4 EMBL-Australia Collaborating Group, Department of Genome Sciences, The John Curtin School of Medical Research, The Australian National University, Garran Road, Acton, ACT 2601 Australia; 5 Proteomics Unit, Bellvitge Biomedical Research Institute (IDIBELL), L’Hospitalet, Barcelona, Catalonia Spain; 6 Department of Pathology, Hospital Universitari de Bellvitge, IDIBELL, L’Hospitalet del Llobregat, Barcelona, Catalonia Spain; 7 Centro de Investigacion Biomedica en Red Cancer (CIBERONC), Madrid, Spain; 8 Program Against Cancer Therapeutic Resistance (ProCURE), Catalan Institute of Oncology (ICO), Bellvitge Institute for Biomedical Research (IDIBELL), Oncobell Program, L’Hospitalet del Llobregat, Barcelona, Catalonia Spain; 9 Vall d’Hebron Institute of Oncology (VHIO), Vall d’Hebron University Hospital, Universitat Autònoma de Barcelona, Barcelona, Catalonia Spain; 10 Centro de Investigación Bimédica en Red de Enfermedades hepáticas y Digestivas (CIBERehd), CIC bioGUNE, 801A Bizkaia Technology Park, 48160 Derio, Spain; 11 Department of Pediatric Hematology and Oncology, Hospital Sant Joan de Déu, Barcelona, Catalonia Spain; 12 Pediatric Neuro-Oncology Unit, Department of Pediatric Hematology and Oncology, Hospital Sant Joan de Déu, Barcelona, Catalonia Spain; 13 Preclinical Therapeutics and Drug Delivery Research Program, Fundacio Sant Joan de Deu, Barcelona, Catalonia Spain; 14 Institute of Oncology of Asturias (IUOPA), Instituto de Investigación Sanitaria del Principado de Asturias (ISPA-FINBA), 33011 Oviedo, Spain; 15 Nanomaterials and Nanotechnology Research Center (CINN-CSIC), Universidad de Oviedo-Principado de Asturias, Oviedo, Spain; 16 Pathology-Brain Bank, Hospital Clínic de Barcelona-CDB-August Pi i Sunyer Biomedical Research Institute (IDIBAPS), Barcelona, Catalonia Spain; 17 Glioma and Neural Stem Cell Group and Translational Genomics and Targeted Therapeutics in Solid Tumors Team, IDIBAPS, Barcelona, Catalonia Spain; 18 Clinical and Experimental Neuroimmunology, IDIBAPS, Barcelona, Catalonia Spain; 19 Faculty of Medicine and Health Sciences-Bellvitge, Universitat de Barcelona, IDIBELL, L’Hospitalet del Llobregat, Barcelona, Catalonia Spain; 20 Department of Pathology, Hospital Universitari Germans Trias i Pujol, Universitat Autònoma de Barcelona, Badalona, Catalonia Spain; 21 Catalan Institute of Oncology, Hospital Germans Trias i Pujol, 08916 Badalona, Barcelona, Catalonia Spain; 22 Department of Chemistry, University of Illinois at Urbana-Champaign, Urbana, IL 61801 USA; 23 Institute for Molecular Biosciences, Goethe University, Frankfurt, Germany; 24 Division of Neuropathology, Institute of Medical Genetics and Pathology, University Hospital Basel, 4031 Basel, Switzerland; 25 Division of Neurosurgery, Toronto Western Hospital, University of Toronto, Toronto, ON Canada; 26 Joint IRB-BSC Program on Computational Biology, Institute for Research in Biomedicine (IRB Barcelona), The Barcelona Institute of Science and Technology (BIST), 08028 Barcelona, Catalonia Spain; 27 Department of Biochemistry and Biomedicine, University of Barcelona, 08028 Barcelona, Spain; 28 Physiological Sciences Department, School of Medicine and Health Sciences, University of Barcelona (UB), Barcelona, Catalonia Spain; 29 Division of Hematology/Oncology, Department of Internal Medicine, University of Cincinnati Medical School, Cincinnati, OH 45267-0508 USA; 30 CIC bioGUNE, 801A Bizkaia Technology Park, 48160 Derio, Spain; 31 Molecular Mechanisms Program, Centro de Investigación del Cáncer and Instituto de Biología Molecular y Celular del Cáncer, Consejo Superior de Investigaciones Científicas (CSIC), University of Salamanca, 37007 Salamanca, Spain; 32 Institucio Catalana de Recerca i Estudis Avançats (ICREA), Barcelona, Catalonia Spain; 33 Victor Chang Cardiac Research Institute, Darlinghurst (Sydney), NSW 2010 Australia; 34 Josep Carreras Leukaemia Research Institute (IJC), Ctra de Can Ruti, Camí de les Escoles s/n, 08916 Badalona, Barcelona, Catalonia Spain

**Keywords:** Glioma, Epitranscriptomics, RNA methylation, Clinical outcome

## Abstract

Tumors have aberrant proteomes that often do not match their corresponding transcriptome profiles. One possible cause of this discrepancy is the existence of aberrant RNA modification landscapes in the so-called epitranscriptome. Here, we report that human glioma cells undergo DNA methylation-associated epigenetic silencing of NSUN5, a candidate RNA methyltransferase for 5-methylcytosine. In this setting, NSUN5 exhibits tumor-suppressor characteristics in vivo glioma models. We also found that NSUN5 loss generates an unmethylated status at the C3782 position of 28S rRNA that drives an overall depletion of protein synthesis, and leads to the emergence of an adaptive translational program for survival under conditions of cellular stress. Interestingly, NSUN5 epigenetic inactivation also renders these gliomas sensitive to bioactivatable substrates of the stress-related enzyme NQO1. Most importantly, NSUN5 epigenetic inactivation is a hallmark of glioma patients with long-term survival for this otherwise devastating disease.

**Electronic supplementary material:**

The online version of this article (10.1007/s00401-019-02062-4) contains supplementary material, which is available to authorized users.

## Introduction

Cellular homeostasis requires accurate regulation of protein levels [46]. In pathological states, such as cellular transformation, the proteome undergoes massive changes [16]. Much effort has been devoted to the study of how events leading to aberrant RNA transcription regulation generate cancer transcriptomes [35]. However, there is an important disjunction between overall transcriptional activity and protein abundance [49]. Defects in protein degradation, such as those affecting the von Hippel–Lindau ubiquitin ligase complex [52] and the endoplasmic reticulum-associated protein degradation pathway [25], might partially explain this discrepancy, but a picture has emerged of how protein levels of key cancer genes are dysregulated by alterations in de novo protein synthesis [43, 45, 48]. Aberrant translational control occurs during the origin, maintenance, and progression of tumoral cells [43, 45, 48]. The enhanced growth of cancer cells often requires an increase in global protein synthesis that it is strongly linked to increased ribosome activity [43, 45, 48]. However, it has recently become clear that this picture is even more complex than previously thought, because oncogenic signaling and stress-related conditions can enhance translation of specific transcripts [2, 47], even in the context of restricted overall protein synthesis [5, 6].

Although several cellular factors may be involved in the aforementioned phenotypes with respect to RNA translation and protein production, it is likely that alterations in components of the machinery of ribosome biogenesis and protein synthesis play a central role in these processes. In this regard, mutations in ribosomal proteins such as RPS19, RPL5, RPS26, and RPL11 occur in Diamond–Blackfan anemia, a syndrome that it is characterized by increased susceptibility to cancer [7]. Interestingly, in X-linked dyskeratosis congenita (X-DC), another syndrome related to increased cancer risk, the main mutated gene is DKC1 [18], a pseudouridine synthase that modifies ribosomal RNA (rRNA) at the post-transcriptional level [37, 53]. The defect in DKC1 gives rise to an altered internal ribosome entry site-dependent translational program [3, 4, 21, 53]. Most importantly, in addition to pseudouridine, ribosomal RNA exhibits another post-transcriptional modification: the presence of 5-methylcytosine [11, 39]. Neither the existence of distorted rRNA 5-methylcytosine profiles in human cancer nor the role of RNA methyltransferases for 5-methylcytosine in rRNA for cellular transformation has been properly characterized [11, 39]. Here, we show that NSUN5, acting as the RNA methyltransferase for the C3782 position of human 28S rRNA, is an enzyme with tumor-suppressor properties that undergoes epigenetic loss in gliomas, leading to an overall deficiency in protein synthesis and the initiation of a specific translational program for adaptation to cellular stress conditions. Most important, and as it also occurs with the isocitrate dehydrogenase (IDH1) mutations [32, 50], the presence of NSUN5 DNA methylation-associated silencing in human gliomagenesis identifies patients with good clinical outcome, which is an exceptional feature of people affected by this tumor type.

## Materials and methods

### Cell lines

Human glioma cell lines DBTRG-05MG, M059J, A172, and LN229 were purchased from the American Type Culture Collection; CAS-1 was obtained from The Biological Bank of the IRCCS Azienda Ospedaliera Universitaria San Martino—IST Istituto Nazionale per la Ricerca sul Cancro; and KS-1 was purchased from the Japanese Collection of Research Bioresources Cell Bank. Cell lines were all cultivated in Dulbecco’s Modified Eagle’s Medium (DMEM) supplemented with 10% v/v fetal bovine serum (FBS) (Innovative, Novi, MI) and 1% v/v penicillin/streptomycin (Gibco). Human grade III glioma cell lines SW1088 and BT142 mut/- were purchased from the American Type Culture Collection and MOG-G-CCM was purchased from Sigma-Aldrich. These cell lines were cultivated regarding to suppliers recommandations. All cell lines were authenticated by short tandem repeat profiling (LGS Standards SLU) and tested for the absence of mycoplasma.

### DNA methylation analyses

DNA methylation status of the 5′-end promoter-associated CpG Island of the NSUN5 gene was determined by bisulfite genomic sequencing and DNA methylation microarrays. For all methods, genomic DNA was bisulfite-converted using an EZ DNA Methylation Gold kit (Zymo Research, Orange, CA, USA). For bisulfite genomic sequencing, a minimum of eight single clones were interrogated for each sample and the methylation frequency was calculated in each case. Results were analyzed with BioEdit software and methylated cytosines were mapped using BSMap software. NSUN5 CpG methylation status in pediatric gliomas was determined by pyrosequencing using the primer sequences shown in the supplementary Key Resources Table. The DNA methylation array used was the Infinium HumanMethylation450 BeadChip (Illumina). Raw fluorescence intensity values were normalized with Illumina Genome Studio software (V2011.1) using ‘control normalization’ with background correction. Normalized intensities were then used to calculate DNA methylation levels (beta values).

### Expression analyses

For real-time quantitative reverse transcription-PCR experiments, total RNA was extracted using the SimplyRNA kit (Promega) on a Maxwell RSC device (Promega) and retrotranscribed using the ThermoScript RT-PCR system (ThermoFisher) with oligo(dT) primers. The reaction was carried out following the methods for the use of SYBR Green (Applied Biosystems), and GAPDH was used as housekeeping genes to enable normalization. Primers for qPCR are listed in the supplementary Key Resources Table. Reactivation treatments with the demethylating agent 5-aza-2′-deoxycytidine (AZA; Sigma) were performed at 0.5 µM for 72 h. For immunoblotting assays, we extracted cell pellets and brain white matter samples with 300 µL of RIPA buffer containing protease and phosphatase inhibitors cocktail cOmplete™ (Roche). Antibodies used in this study are described in supplementary Key Resources Table.

### NSUN5 transfection

The cDNA sequence of NSUN5 containing a FLAG-Tag in the amino-terminal end was cloned into the pLVX-IRES-ZsGreen1 expression plasmid (Clontech Laboratories) between the EcoRI and XbaI restriction sites. Lentiviruses containing this construct were produced by cotransfecting HEK-293T cells with the recombinant pLVX-IRES-ZsGreen1, psPAX2 (Addgene), and pMD2.G (Addgene), using jetPRIME^®^ Transfection Reagent (Polyplus Transfection) and following the supplier instructions. The transfection cocktail was removed after 6 h and replaced by fresh medium. After 72 h, viral containing supernatant was collected, 0.45 µM-filtered, and stored at 4 °C before infection. The recombinant product was randomly inserted by lentiviral transduction in the genome of the A172 and LN229 glioma cell lines. After 5 passages, the green fluorescent cells were sorted by FACS and cultured in DMEM medium supplemented with 10% FBS and 1% v/v penicillin/streptomycin (Gibco). For primers sequences, please refer to supplementary **Key Resources Table**.

### NSUN5 depletion

Four different sequence gene-specific short hairpin RNA molecules (shRNAs) for NSUN5 mRNA were designed and transduced into DBTRG-05MG and CAS-1 glioma cell line (for sequences please refer to supplementary Key Resources Table). shRNA against the MSS2 yeast mRNA (not present in mammals) was used as scrambled (control). All shRNA molecules were ligated into pLVX-shRNA2-ZsGreen plasmid (Clontech). 10 ug of each encoding plasmid was mixed with 7.5 μg of ps-PAX2 and 2.5 μg of PMD2.G plasmid (Addgene), using jetPRIME^®^ Transfection Reagent (Polyplus Transfection). Upon 10 min of RT incubation, the transfection mix was added dropwise on a 10 cm culture plate containing HEK293-TLV lentiviral packaging cells at 80% confluence. After 72 h, medium with high-titer lentiviral particles was 0.45 μm filtered. DBTRG-05MG and CAS-1 glioma cell lines were cultured in virus containing medium for 24 h. After 5 passages, the green fluorescent cells were sorted by FACS and cultured in DMEM medium supplemented with 10% FBS and 1% v/v penicillin/streptomycin (Gibco).

### Brain tumor xenografts

#### IVIS images

All mouse experiments were approved and performed in accordance with the guidelines of the Institutional Animal Care Committee of the Vall d’Hebron Research Institute. 1 × 10^6^ cells were stereotactically inoculated into the corpus striatum of the right brain hemisphere of 9-week-old athymic mice Nude-Foxn1nu mice (Charles River Laboratories). To estimate the size of tumors, the luciferase activity of inoculated tumor cells was quantified in a Xenogen–CCD camera from IVIS. All mouse experiments were approved by and performed according to the guidelines of the Institutional Animal Care Committee of the Vall d’Hebron Research Institute in agreement with the European Union and national directives. 3 × 10^5^ A172 and LN229 (EV or NSUN5) or DBTRG-05MG (scramble or shNSUN5) cells were stereotactically inoculated into the corpus striatum of the right brain hemisphere (1 mm anterior and 1.8 mm lateral to the lambda; 2.5 mm intraparenchymal) of 8-week-old Balb/c nude mice (Janvier Labs). We inoculated 7 animals per group. Tumor progression was monitored by bioluminescence measurements using the Xenogen IVIS^®^ Spectrum. Mice were euthanized at day 17 after inoculation.

#### Xenografts growth

All mouse experiments were approved and performed in accordance with the guidelines of the Institutional Animal Care Committee of the Bellvitge Biomedical Research Institute. For tumor growth into the brain, 3 × 10^5^ or 1.5 × 10^6^ cells, respectively, for LN229 (EV or NSUN5) and DBTRG-05MG (scramble or shNSUN5) were inoculated into the corpus striatum of 9 (scramble and shNSUN5) or 10 (EV and NSUN5) mice for each cell line individually.

### NSUN5 immunoprecipitation and RT-qPCR

Immunoprecipitations were carried out from total LN229 extracts (transfected with empty vector or NSUN5) overnight with 10 μl of Anti-Flag⨁ M2 Magnetic Beads (Sigma) in RIP buffer (150 mM KCl, 25 mM Tris pH7.4, 5 mM EDTA, 0.5 mM DTT, 0.5% NP40, protease inhibitors). Beads were then washed three times with RIP buffer, and 10% of beads were boiled in Laemmli buffer 1× and loaded on a protein gel to check immunoprecipitation efficiency. The pulled-down RNA from the remaining 90% of beads was extracted by adding 1 mL of TRIzol⨁ Reagent (ThermoFisher). After phenol extraction and isopropanol precipitation, a DNase treatment step was performed, and the final pellet was resuspended in water. Equal amounts of each sample were then retrotranscribed and analyzed by quantitative PCR (Applied Biosystems 7900HT Fast Real-Time PCR System). The equivalent amount of input RNA was processed in parallel to estimate pull-down efficiency.

### RNA methylation analyses

RNA bisulfite conversion and sequencing was performed following Epigentek Methylamp RNA bisulfite conversion Kit instructions. The resulting bisulfite-modified RNA was converted in cDNA using the ThermoScript RT-PCR system (ThermoFisher) with random hexamer primers. PCR primers for the sequence of interest are available in supplementary Key Resources Table. The amplicon PCR band was purified and ligated into pGEM^®^-T Easy vector (Promega), transformed, and sequenced as described previously in DNA methylation analyses. Bisulfite RNA deep-sequencing (Bis-seq) was performed as described in Suppl. Methods.

### Global determination of protein synthesis

Overall protein synthesis in the different experimental conditions was assessed by the incorporation of O-propargyl-puromycin (OP-Puro) into nascent proteins using the Click-iT^®^ OPP Reagent (Thermo Fisher) [5] and [3H] leucine incorporation.

### Assessment of translational efficiency

Translational efficiency for each RNA was determined by normalizing the ribosome profiling deep-sequencing data (Ribo-seq) to transcript length and total transcript abundance according to deep-sequencing RNA transcriptome (RNA-seq) data, as described in Suppl. Methods.

### Gene functional-enrichment analysis and sequence motifs recognition

Gene sets were used to perform a gene set over-representation analysis over the GO-biological processes and KEGG pathways included in the public GSEA signature database collections and the DAVID web service. The top gene clusters resulting from the hypergeometric test with an FDR adjusted *P* value < 0.05 was finally considered. 5′UTR DNA sequences from selected genes were used to perform a motif recognition analysis. RegRNA 2.0 and FIMO software from MEME suite 4.12.0 were used to perform CERT, TOP, PRTE, and uORF motif scanning. IRESPred web service and Infernal software were used to identify IRES motifs. G4-quadruplexes were identifies using QGRS Mapper software.

### Stable isotope labeling by amino acids in cell culture (SILAC)

SILAC was performed as previously described [25]. SILAC labeling was performed using SILAC-Lys8- Arg10-Kit media (Silantes). Peptide mixes were analyzed using an OrbitrapFusion Lumos mass spectrometer (Thermo Scientific, San Jose, CA, USA) coupled to an EasyLC (Thermo Scientific (Proxeon), Odense, Denmark). All data were acquired with Xcalibur software v3.0.63. Proteome Discoverer software suite (v2.0, Thermo Fisher Scientific) and the Mascot search engine (v2.5, Matrix Science (1)) were used for peptide identification and quantification. Samples were searched against a SwissProt database containing entries corresponding to Human (version of April 2016) a list of common contaminants and all the corresponding decoy entries. Resulting data files were filtered for FDR < 1%.

### Deoxynyboquinone and isobutyl-deoxynyboquinone drug assays

IC50 studies in the glioma cell lines upon drug exposure were performed using the sulforhodamineB (SRB) assay. Briefly, 48 h after exposure to nine increasing concentrations of deoxynyboquinone (2 nM–15 µM), culture medium was removed and 100 µL of 10% trichloroacetic acid was added to the wells to fix cells for 1 h at 4 °C. Cells were then washed twice with distilled water and stained with 100 µL of 0.4% SRB in 1% acetic acid during 30 min, light-protected, and washed twice with 1% acetic acid. SRB was then solubilized in Tris base (10 mM; pH 10.0) and 540 nm-optical density was determined using a microplate reader (Perkin Elmer Viktor 3).

### Patients

DNA methylation data in the discovery set of glioma cases was collected from The Cancer Genome Atlas (TCGA) Data Portal (https://tcga-data.nci.nih.gov/tcga/). For the initial glioma validation cohort, 115 formalin-fixed paraffin-embedded (FFPE) tumor tissues from glioma patients were retrospectively collected in four different centers (Hospital Universitari de Bellvitge, Hospital Germans Trias i Pujol, Hospital Clínic de Barcelona and University Hospital Basel) from 1989 to 2018 and were histologically reviewed. Most of patients had received treatment based on temozolamide combined or not with radiotherapy, and molecular analyses of IDH1 mutational status and MGMT methylation were available. Co-deletion 1p19q status was only available in 23 of the 115 cases. Patients gave their informed consent to participate in the research, which had received ethical approval from the review board of each institution. The expanded glioma validation cohort included 303 additional glioma patients from which NSUN5 methylation, IDH1 mutation, co-deletion of 1p19q, MGMT methylation, and progression-free survival are available [1, 22]. These cases had signed written informed consent and were histologically reviewed as described in the respective publications [1, 22].

### Statistical analysis

The associations between variables were assessed by *χ*^2^ tests, Fisher’s exact test, Mann–Whitney test, Welch’s *t* test, Wilcoxon paired test, or Spearman correlation whenever indicated. Kaplan–Meier plots and log-rank test were used to estimate Overall Survival (OS). Statistical analysis was performed using SPSS for Windows (Armonk, NY) and GraphPad Prism 5 (La Jolla, CA) for Windows. *P* values less than 0.05 were considered statistically significant. All statistical tests were two-sided. Methylation and expression values for GBM and LGG TCGA primary tumor samples were obtained from the NCI’s Genomic Data Commons (GDC). The values corresponding to glioma cell lines were obtained from COSMIC cell line database. Correlations were obtained by calculating Spearman’s rank correlation test and the associated rho coefficient.

### Data availability

All the obtained deep-sequencing data have been deposited at the SRA (https://www.ncbi.nlm.nih.gov/sra) with the following links:

RNA-Seq: BioProject: PRJNA395552

https://dataview.ncbi.nlm.nih.gov/object/PRJNA395552?reviewer=60ndb08i1g6na5nkoaaiad9a6o

bsRNA-Seq: BioProject: PRJNA395575

https://www.ncbi.nlm.nih.gov/bioproject/395575

Ribo-Seq: BioProject: PRJNA395570

https://www.ncbi.nlm.nih.gov/bioproject/395570

## Results

### Identification of NSUN5 CpG island promoter hypermethylation-associated transcriptional silencing in glioma cells

**Fig. 1:**
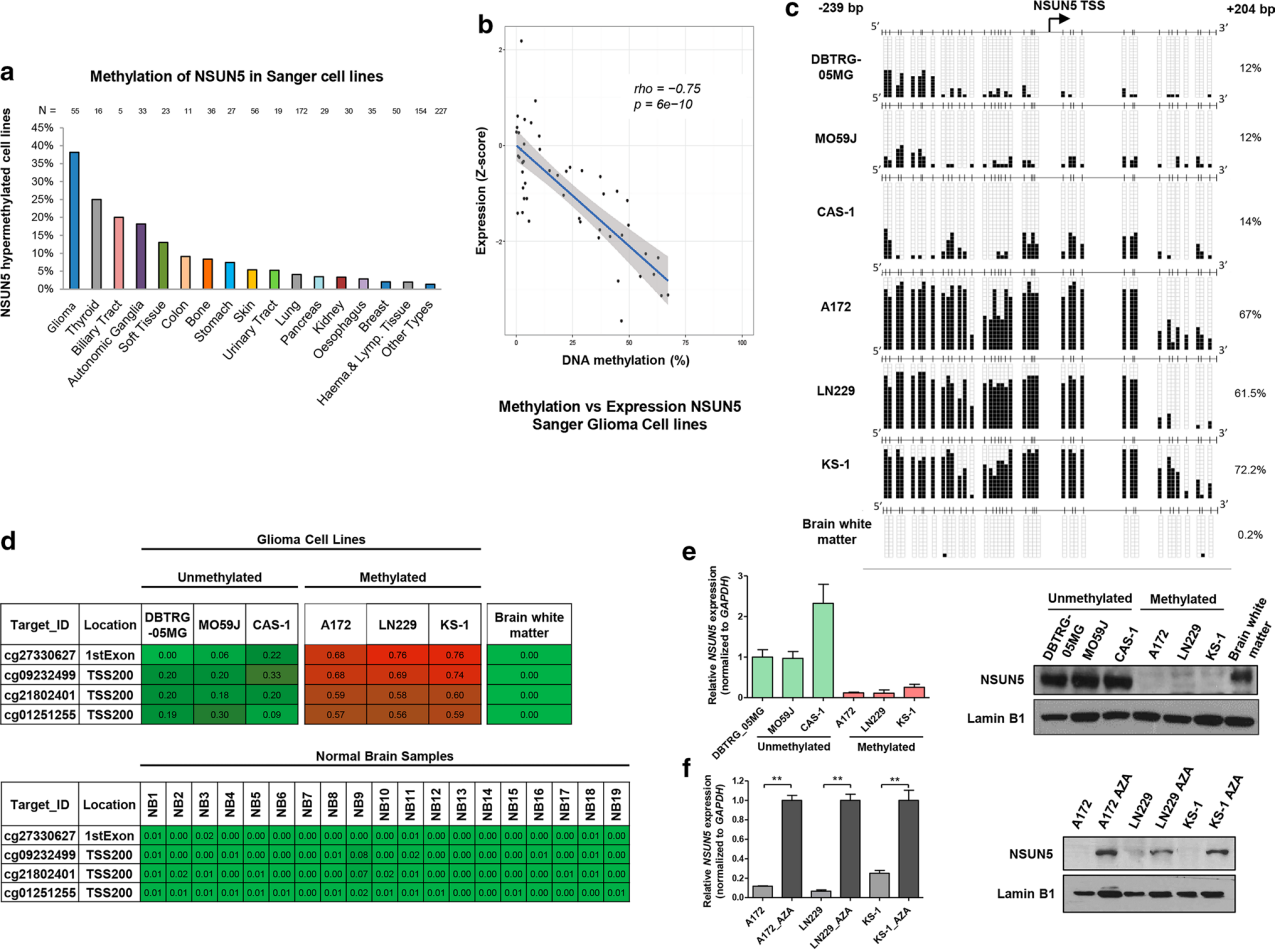
Transcriptional silencing of NSUN5 by promoter CpG island hypermethylation in human glioma cells. **a** Percentage of NSUN5 methylation in the Sanger panel of cancer cell lines by tumor type. **b** NSUN5 methylation is associated with loss of the transcript in the glioma cell lines from Sanger (*n* = 48). Correlation analysis between methylation beta values and expression *Z*-score are shown. The *P* value corresponding to Spearman’s rank correlation test and the associated rho coefficient are indicated in the figure. **c** Bisulfite genomic sequencing of NSUN5 promoter CpG Island in glioma cells lines and brain white matter. CpG dinucleotides are represented as short vertical lines and the transcription start site (TSS) is represented as a long black arrow. Single clones are shown for each sample. Presence of an unmethylated or methylated cytosine is indicated by a white or black square, respectively, and percentage of methylation is indicated on the right. **d** DNA methylation profile of the CpG island promoter for the NSUN5 gene analyzed by the 450 K DNA methylation microarray. Single CpG absolute methylation levels (0–1) are shown. Green, unmethylated; red, methylated. Data from the studied six glioma cell lines, brain white matter and nineteen normal brain samples are shown. **e** NSUN5 expression levels in glioma cell lines determined by real-time PCR (data shown represent mean ± S.D. of biological triplicates) and western blot. **f** Expression of the NSUN5 RNA transcript and protein was restored in the A172, LN229 and KS-1 cells by treatment with the demethylating drug 5-aza-2′-deoxycytidine (AZA). Data shown represent the mean ± S.D. of biological triplicates and *P* values were obtained by the Mann–Whitney test. **P < 0.01

To identify candidate genetic and epigenetic defects in the proposed ribosomal RNA cytosine methyltransferases NSUN5, NSUN1 (28S rRNA C4447), and NSUN4 (12S mitochondrial rRNA C841) in human tumorigenesis, we first data-mined a collection of 1001 human cancer cell lines in which we had recently determined the exome mutational, transcriptomic, gene copy number, and DNA methylation profiles [20]. The available genomic data did not indicate the presence of NSUN5, NSUN1, or NSUN4 mutations, amplifications, or deletions in the cell lines considered (Suppl. Dataset 1). Although genetic defects in the described genes were not found, transcriptional silencing by promoter CpG island hypermethylation is another mechanism for inactivating genes in human cancer [12]. The NSUN1 and NSUN4 promoter-associated CpG islands were unmethylated in the cell lines studied (Suppl. Datasets 1b and c). In contrast, the NSUN5 promoter CpG island was methylated in 38% (21 of 55) of the glioma-derived cell lines studied (Fig. 1a and Suppl. Dataset 1a). Data-mining of microarray expression results [20] showed that NSUN5 hypermethylation was associated with transcript downregulation in the glioma cell lines (Fig. 1b). Beyond glioma-derived malignancies, the NSUN5 promoter CpG island was mostly found to be unmethylated in the other tumor types available in our data set, with the exception of thyroid cancer (4 of 16, 25%), biliary tract (1 of 5, 20%), autonomic ganglia (6 of 33, 18%), and soft-tissue sarcoma (3 of 23, 13%) (Fig. 1a and Suppl. Dataset 1a). The NSUN5 promoter CpG island was unmethylated in all the normal human tissues studied (Suppl. Dataset 1d).

Having identified the aforementioned NSUN5 CpG island methylation profiles, we studied in greater detail their association with the possible transcriptional inactivation of the NSUN5 gene at the RNA and protein levels in glioma cell lines. We performed bisulfite genomic sequencing of multiple clones in the glioblastoma cell lines DBTRG-05MG, MO59J, CAS-1, A172, LN229, and KS-1 using primers that encompassed the transcription start site-associated CpG island. We found the 5′-end region of NSUN5 of the LN229, A172, and KS-1 glioma cell lines to be hypermethylated in comparison with normal brain white matter (Fig. 1c), whereas the DBTRG-05MG, CAS-1, and MO59 J cells were unmethylated (Fig. 1c). These results validated the DNA methylation patterns obtained by the microarray approach (Fig. 1d). Normal astrocytes, fetal brain, and different brain regions (such as frontal cortex, posterior cingulate cortex, and hippocampus) were unmethylated for NSUN5 in all cases (**Dataset 1e**). Positive and negative technical controls for the DNA methylation assays are shown in Suppl. Dataset 1f. Most notably, the NSUN5-hypermethylated glioma cell lines LN229, A172 and KS-1 minimally expressed the NSUN5 RNA transcript and protein, as determined by quantitative real-time PCR and western blot, respectively (Fig. 1e). Conversely, the glioma cell lines unmethylated at the NSUN5 promoter (DBTRG-05MG, CAS-1, and MO59J) expressed NSUN5 RNA and protein (Fig. 1e). Treatment of the methylated glioblastoma cell lines with the DNA-demethylating agent 5-aza-2-deoxycytidine restored NSUN5 expression at the RNA and protein levels (Fig. 1f) in association with hypomethylation events at the promoter CpG island (Suppl. Fig. S1a). We extend the study to three grade III glioma cell lines: BT142 mut/−, harboring a homozygous R132H mutation [26], and SW1088 and MOG-G-CCM, wild type for IDH1. We found DNA methylation-associated silencing of NSUN5 in BT142 mut/- cells, whereas the two remaining cell lines (SW1088 and MOG-G-CCM) were unmethylated and expressed NSUN5 (Suppl. Fig. S1b and c). Treatment of the methylated cell line BT142 mut/- with the DNA methylation inhibitor restored NSUN5 expression at the RNA and protein levels (Suppl. Fig. S1d). The study of 16 patient-derived xenograft (PDX) from primary gliomas engrafted in nude mice showed that NSUN5 was hypermethylated in three cases (19%). RNA was available for four cases (two unmethylated and two methylated) and NSUN5 hypermethylation was associated with diminished transcript levels (Suppl. Fig. S1e). Overall, these results indicate that cancer-specific promoter CpG island hypermethylation-associated silencing of the NSUN5 gene occurs in glioma cells.

**Fig. 2:**
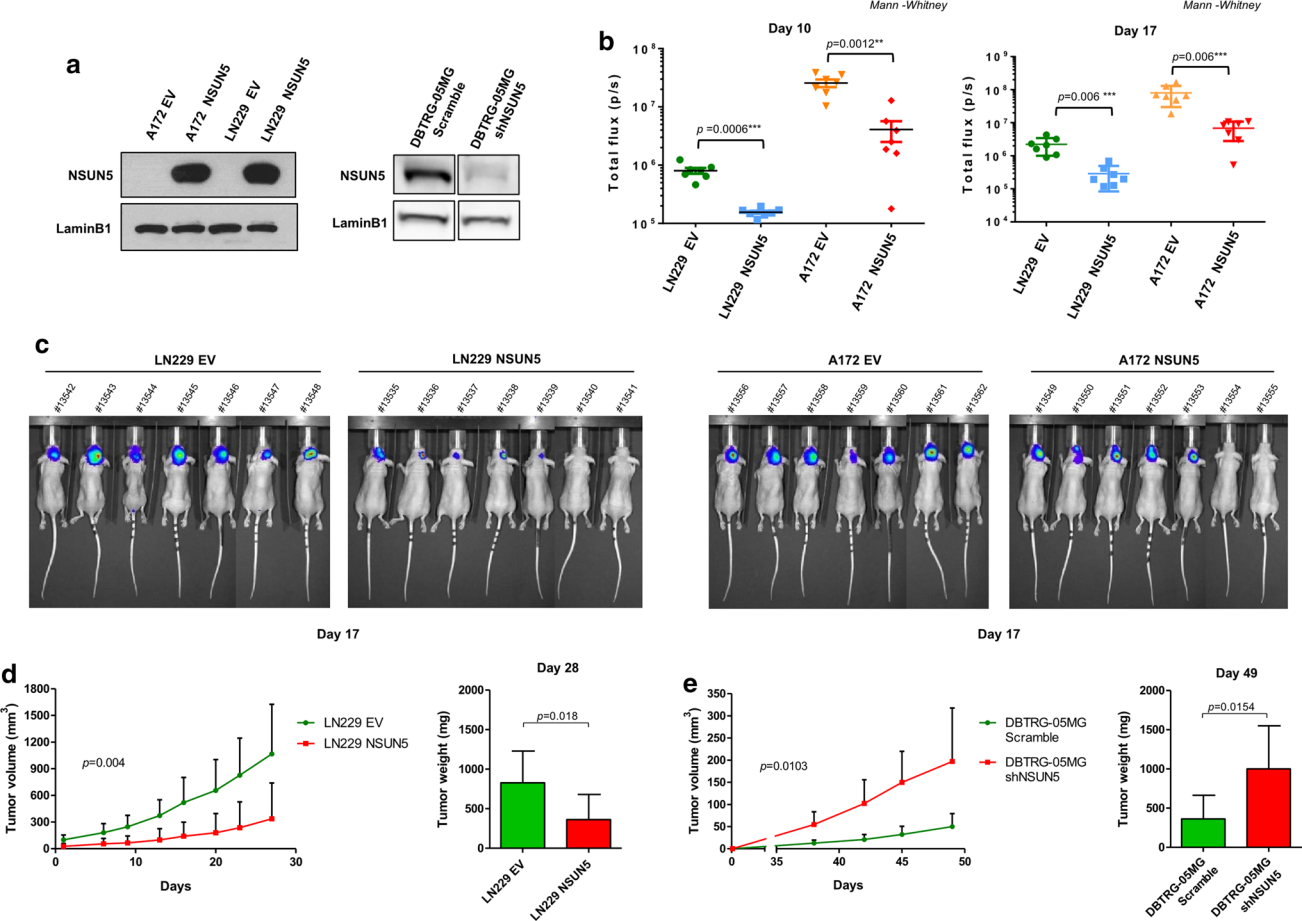
Restoration of NSUN5 impairs glioma tumor growth in vivo. **a** Western blot to show efficient restoration of NSUN5 protein expression upon stable transfection in A172 and LN299 glioma cells and efficient depletion of NSUN5 protein expression in NSUN5-shRNA DBTRG-05MG glioma cells. *EV* empty vector. An equal number of the indicated A172 and LN299 cells populations were stereotactically inoculated into the brain of athymic mice. The size of the tumors was estimated at 10 and 17 days post-inoculation (DPI) by the quantification of luciferase activity in the tumor cells. **b** Scatter plots showing the individual size of the indicated LN229 and A172 tumors after 10 and 17 DPI. **c** Representative images of the luciferase signal from mice inoculated with the indicated LN229 and A172 tumors after 17 DPI. **d** LN229-EV and LN229-NSUN5 cells were injected in the left or right flank of 10 mice, respectively. Tumor volume measured over time (*left panel*) and tumor weight upon sacrifice (*right panel*) are shown. *P* values obtained by Student’s *t* test. Error bars show means ± s.d. **e** Scramble and NSUN5-shRNA-depleted DBTRG-05MG cells were injected in the left or right flank of 10 mice, respectively. Tumor volume measured over time (left panel) and tumor weight upon sacrifice (right panel) are shown. *P* values obtained by Student’s *t* test. Error bars show means ± s.d

Once we had demonstrated the existence of NSUN5 CpG island hypermethylation-associated transcriptional loss in glioma cell lines, we studied its contribution to the growth of these cells. To this end, we analyzed the effect of the restoration of NSUN5 expression in the NSUN5-hypermethylated glioblastoma cell lines LN229 and A172. Upon efficient transfection of NSUN5 (Fig. 2a), we studied the growth of glioma cells in vivo by performing intracerebral mouse xenotransplantation experiments using LN229 and A172 GFP-Luc cell lines, and quantified tumor growth over time by measuring luciferase activity. Tumors obtained from NSUN5-transfected LN229 and A172 cell lines showed a significant reduction in size at 10 and 17 day post-inoculation in comparison with the respective empty-vector-transfected cells (Fig. 2b, c). In the reverse experiment, we observed that tumors arising from unmethylated DBTRG-05MG cells undergoing NSUN5 shRNA-mediated depletion showed a significant increase in size in comparison with those originated from scramble shRNA cells (Suppl. Fig. S2a, b and c). The study of subcutaneously injected cancer cells into nude mice provided similar results: tumors originated from NSUN5-transfected LN229 cells had a significantly lower volume and weight than empty-vector-transfected-derived tumors (Fig. 2d), whereas NSUN5 shRNA-mediated depletion in the unmethylated DBTRG-05MG cell line increased the volume and weight of the derived tumors (Fig. 2e). Comparing NSUN5 shRNA-depleted vs scramble DBTRG-05MG cells injected orthotopically in the brain of nude mice, we found that NSUN5 downregulation was associated with shorter overall survival (Suppl. Fig. S2e), whereas transfection-mediated recovery of NSUN5 expression in LN229 hypermethylated cells was associated with longer overall survival (Suppl. Fig. S2d). NSUN5 restoration by transfection in hypermethylated glioblastoma cells also diminished proliferation in vitro (Suppl. Fig. S3a), whereas its shRNA-mediated downregulation in NSUN5 unmethylated cells increased cell viability (Suppl. Fig. S3b). The glioma-related genes IDH1, TET1, or TET3 are wild type in the studied cells lines (COSMIC) and we observed a similar expression of the three proteins in the three NSUN5 unmethylated and methylated glioma cell lines (Suppl. Fig. S3c). The expression of these proteins did not change upon NSUN5 restoration in LN229 cells or depletion in DBTRG-05MG cells (Suppl. Fig. S3c). These results suggest that NSUN5 has a specific tumor-suppressor role in gliomas.

### NSUN5 is the RNA methyltransferase for the C3782 position of 28S rRNA

**Fig. 3:**
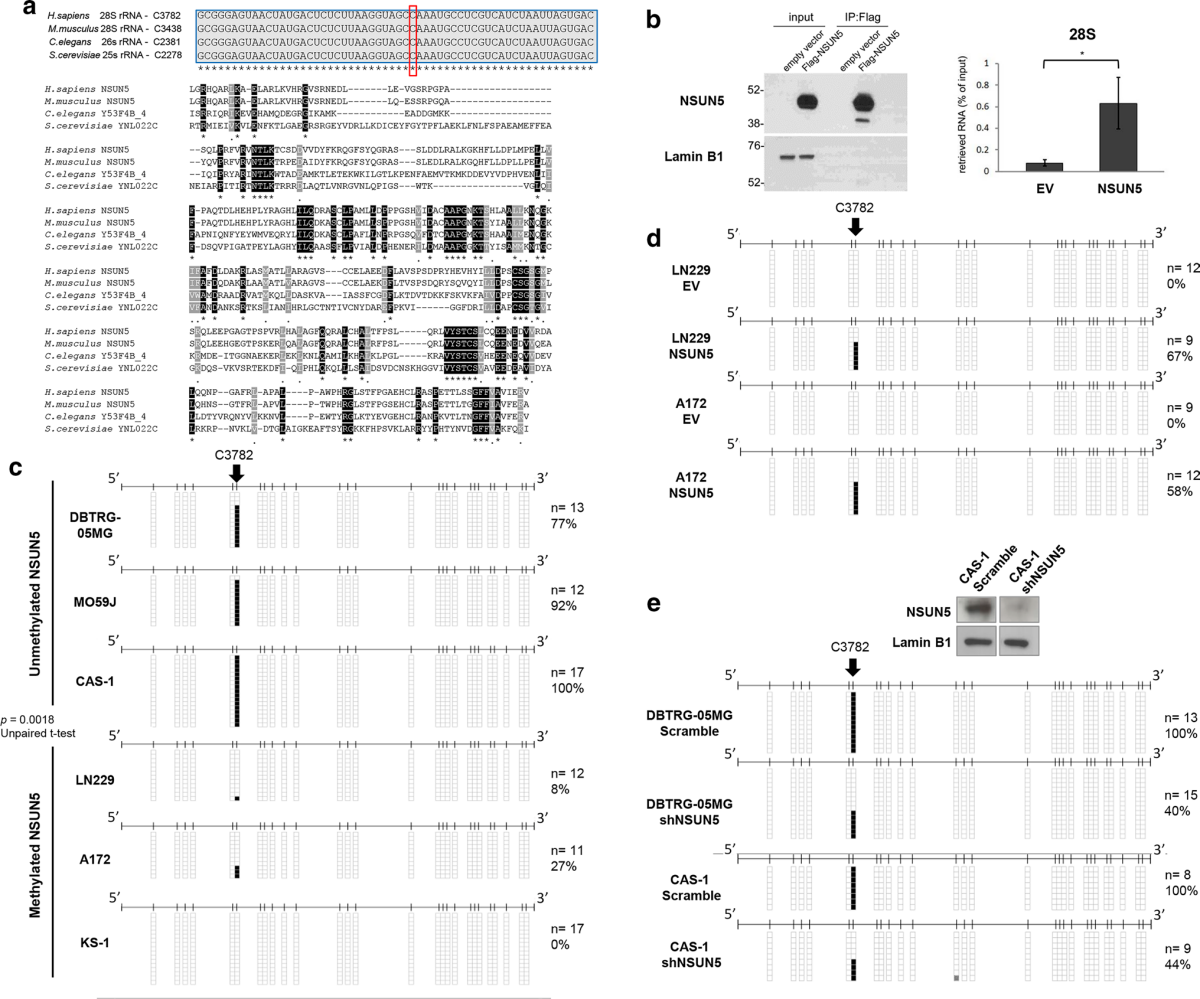
NSUN5 epigenetic inactivation abrogates the methylation of the C3782 position of human 28S rRNA. **a***Top*, RNA sequence alignment of the conserved human 28S rRNA C3782 position (black square) in the corresponding 26S, 25S and 28S rRNAs orthologues from *C. elegans*, *S. cerevisiae* and *M. musculus*. *Below*, Protein sequence alignment of human NSUN5 with its recognized rRNA 5-methylcytosine RNA-methyltransferase orthologues in *C. Elegans*, *S. Cerevisiae* and *M. musculus*. Highlighted in black and grey the identical and physicochemically similar (scoring > 0.5 in the Gonnet PAM 250 matrix) residues, respectively. The aligned region includes the RNA-methyltransferase domain. **b** NSUN5 interaction with 28S rRNA. Total extracts from LN229 cells, either transfected with empty vector (EV) or NSUN5 were immunoprecipitated with an anti-Flag antibody (*left panel*), followed by analysis of the retrieved RNA by quantitative RT-PCR (*right panel*). **c** RNA bisulfite sequencing of the 28S rRNA in glioma cells lines according to NSUN5 epigenetic status. Cytosines are represented as short vertical lines and the C3782 site is represented as a long black arrow. Single clones are shown for each sample. Presence of an unmethylated or methylated cytosine is indicated by a white or black square, respectively. **d** RNA bisulfite sequencing of the 28S rRNA in empty-vector (EV) and NSUN5-transfected LN229 and A172 glioma cells. **e** RNA bisulfite sequencing of the 28S rRNA in scramble and NSUN5-shRNA-depleted DBTRG-05MG and CAS-1 glioma cells. For CAS-1, western-blot to show efficient NSUN5 depletion is shown above

The activity and targets of NSUN5 in human cells have not been formally identified, but the yeast *S. cerevisiae* (Rcm1) [13, 38, 40] and worm *C. elegans* (*nsun*-*5*) [38] homologues are responsible for methylating C2278 in the 25S rRNA and C2381 in the 26S rRNA, respectively. Phylogenetic analyses show that the C2278 and C2381 positions in the yeast and worm 25S and 26S rRNAs, respectively, correspond to the C3782 position of the human 28S rRNA (Fig. 3a). This site is placed in the vicinity of the peptidyltransferase center of the large ribosomal subunit [36, 38], suggesting that it is relevant for core ribosomal function. Thus, we set out to demonstrate that NSUN5 is responsible for methylating C3782 28S rRNA in our glioma models.

For this purpose, we first showed that 28S rRNA represents a binding target of NSUN5, since immunoprecipitation of NSUN5 specifically retrieved this RNA sequence in the RNA pull-down assay (Fig. 3b). We next performed comparative bisulfite sequencing of multiple clones of the 28S rRNA extracted from the NSUN5 unmethylated glioblastoma cell lines DBTRG-05MG, MO59J, and CAS-1, and the NSUN5 hypermethylated and transcriptionally silenced LN229, A172, and KS-1 glioblastoma cell lines. We observed that while the NSUN5-expressing DBTRG-05MG, MO59J, and CAS-1 cells showed high levels of C3782 28S rRNA methylation, the LN229, A172, and KS-1 cells undergoing NSUN5 epigenetic inactivation displayed a lack of C3782 28S rRNA methylation (Fig. 3c). Treatment of the NSUN5 methylated glioblastoma cell lines with the DNA-demethylating agent 5-aza-2-deoxycytidine not only restored NSUN5 expression (Fig. 1f), but also recovered C3782 28S rRNA methylation (Suppl. Fig. S4a). The LN229 and A172 cells, showing NSUN5 epigenetic silencing, also recovered C3782 28S rRNA methylation upon restoration of NSUN5 activity by transfection (Fig. 3d). Importantly, we constructed an NSUN5 catalytic inert form due to the presence of multiple engineered mutations (Suppl. Fig. S4b and c) that was unable to restore C3782 28S rRNA methylation (Suppl. Fig. S4d). The mung bean nuclease protection assay (Suppl. Methods) confirmed the RNA bisulfite sequencing data for C3782 28S rRNA methylation (Suppl. Fig. S4e, f and g). LN229 cells with NSUN5 epigenetic loss were depleted for C3782 28S rRNA methylation in both nuclear and cytoplasmic fraction, whereas NSUN5 transfection restored the methylation status at both compartments (Suppl. Fig. S5a). To demonstrate the specificity of our experimental approach, we showed that the transfection of NSUN5 in LN229 and A172 cells did not change the levels of the other two candidate human ribosome RNA methyltransferases, NSUN1 and NSUN4 (Suppl. Fig. S5b). We also studied the stable knockdowns of NSUN5 by the short hairpin RNA (shRNA) method in the NSUN5-expressing and unmethylated glioma cell lines DBTRG-05MG and CAS-1. Upon effective NSUN5 shRNA-mediated downregulation, we observed hypomethylation at the C3782 28S rRNA site in DBTRG-05MG and CAS-1 cells (Fig. 3e). The link between NSUN5 epigenetic silencing and C3782 28S rRNA hypomethylation was also observed in the grade III glioma cell lines: BT142 mut/- cells, harboring DNA methylation-associated silencing of NSUN5, showed an unmethylated C3782 28S site, whereas SW1088 and MOG-G-CCM (both unmethylated and expressing NSUN5) showed a methylated C3782 28S rRNA position (Suppl. Fig. S1f).

We also moved from the candidate approach to a non-biased genomic setting to identify the spectrum of NSUN5 targets. To map NSUN5-dependent modified cytosine sites across the human transcriptome, we coupled bisulfite conversion of cellular RNA with next-generation sequencing (bsRNA-seq) [44] in NSUN5-transfected LN229 glioma cells and empty-vector-transfected cells. Upon efficient restoration of NSUN5 expression, we observed that the only cytosine site in RNA that reached methylation levels of over 90% following NSUN5 transfection was the C3782 position of the 28S rRNA (empirical Bayes moderated *t* test, *P* = 0.033). Interestingly, the specificity of the approach was further confirmed by identifying that the only other methylated cytosine in 28S rRNA, the C4447 position corresponding to the C4099 position in mouse 28S rRNA [23] and suggested to be mediated by the other RNA-methyltransferase NSUN1 [40], was fully methylated in both empty-vector and NSUN5-transfected cells (Suppl. Fig. S5c). Thus, all these results indicate that NSUN5 is the human C3782 28S rRNA methyltransferase and that epigenetic silencing of NSUN5 in glioma cells prevents the formation of this chemical modification in the rRNA transcript considered here.

**Fig. 4:**
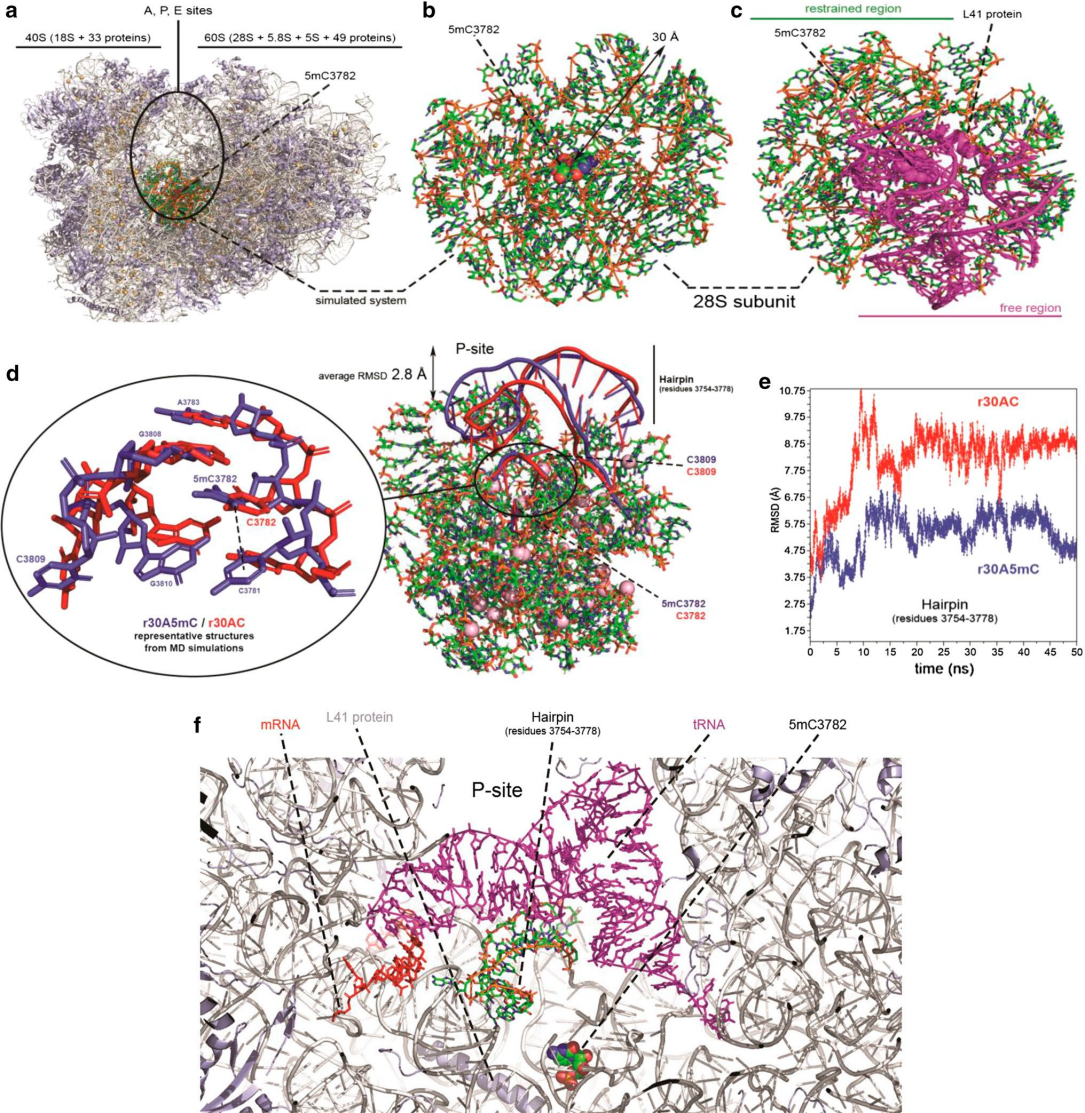
Methylation of the C3782 position of human 28S rRNA mediates the structural stability of the tertiary complex rRNA–tRNA–mRNA and its loss leads to a structural distortion at the edge of the 28S subunit in the P-site cavity. **a** Structure of the Human 80S ribosome taken from PDB code 6EK0. Proteins and RNA molecules are depicted in light-blue and white cartoons respectively, while Mg2 + are represented by pink spheres. **b** System considered in the MD simulations: 30 Å around residue C3782, which is depicted by its van der Waals radii (see “Materials and methods”). **c** Residues constrained at the experimental positions (nucleobases in green and backbone in orange), and residues completely free to move during the simulation (violet). **d** Local (left) and “long-range” (right) structural distortions observed in MD simulations after removal of the methyl group at position C3782 of the 28S subunit. Two representative structures taken from the simulations were aligned and overlapped to highlight structural differences. Methyl–*π* interaction is lost on the unmethylated system (r30AC, red), leading to a significant conformational change in the hairpin defined by residues 3754–3778. **e** Root-Mean-Squared-Deviation (RMSD) of the heavy atoms in the P-site hairpin (residues 3754–3778) along time. The hairpin in the r30A5mC system adopted a conformation in average 5.4 ± 0.8 Å apart from the cryoEM structure, while the unmethylated systems (r30AC) deviates 8.2 ± 1.2 Å. **f** Eukaryotic 80S ribosome taken from the cryoEM structure (5.5 Å-resolution) deposited with the PDB code 4V6I, highlighting the interaction between the P-site hairpin (nucleobases in green and backbone in orange), the tRNA (violet), and the mRNA (red)

To evaluate the structural impact of removing the methyl group of the C3782 position of the 28S rRNA, we simulated by means of Molecular Dynamics (MD) a sub-region of the human 28S subunit around 5mC3782 (see “Materials and methods” and Fig. 4a–c). The simulation of the system with methylated C3782 (labelled r30A5mC) showed a stable methyl-π interaction between methylated C3782 and C3781 (Fig. 4d), while this interaction was lost in the unmethylated system (r30AC). The removal of the methyl group altered significantly the C3781-G3810 base pair and the conformation of the bulge at position C3809 (Fig. 4d). The bulge interacts with a small hairpin (residues 3742–3778) which is located at the edge of the 28S subunit, exposed to the P-site of the ribosome (Fig. 4a–d). In the unmethylated system, the hairpin undergoes a conformational change moving away from the P-site cavity, showing an average displacement of 2.8 Å with respect to the methylated form (Fig. 4e). The role of this small hairpin is to interact directly through several residues with the tRNA molecule at the P-site, and even with the mRNA [13], being crucial to ensure the structural stability of the tertiary complex rRNA–tRNA–mRNA (Fig. 4f). The distortion on the hairpin observed in the simulation upon the removal of the methyl group (r30AC system) might be sufficient to impair normal protein synthesis by altering the P-site conformation.

### NSUN5 epigenetic loss restrains overall protein synthesis

**Fig. 5:**
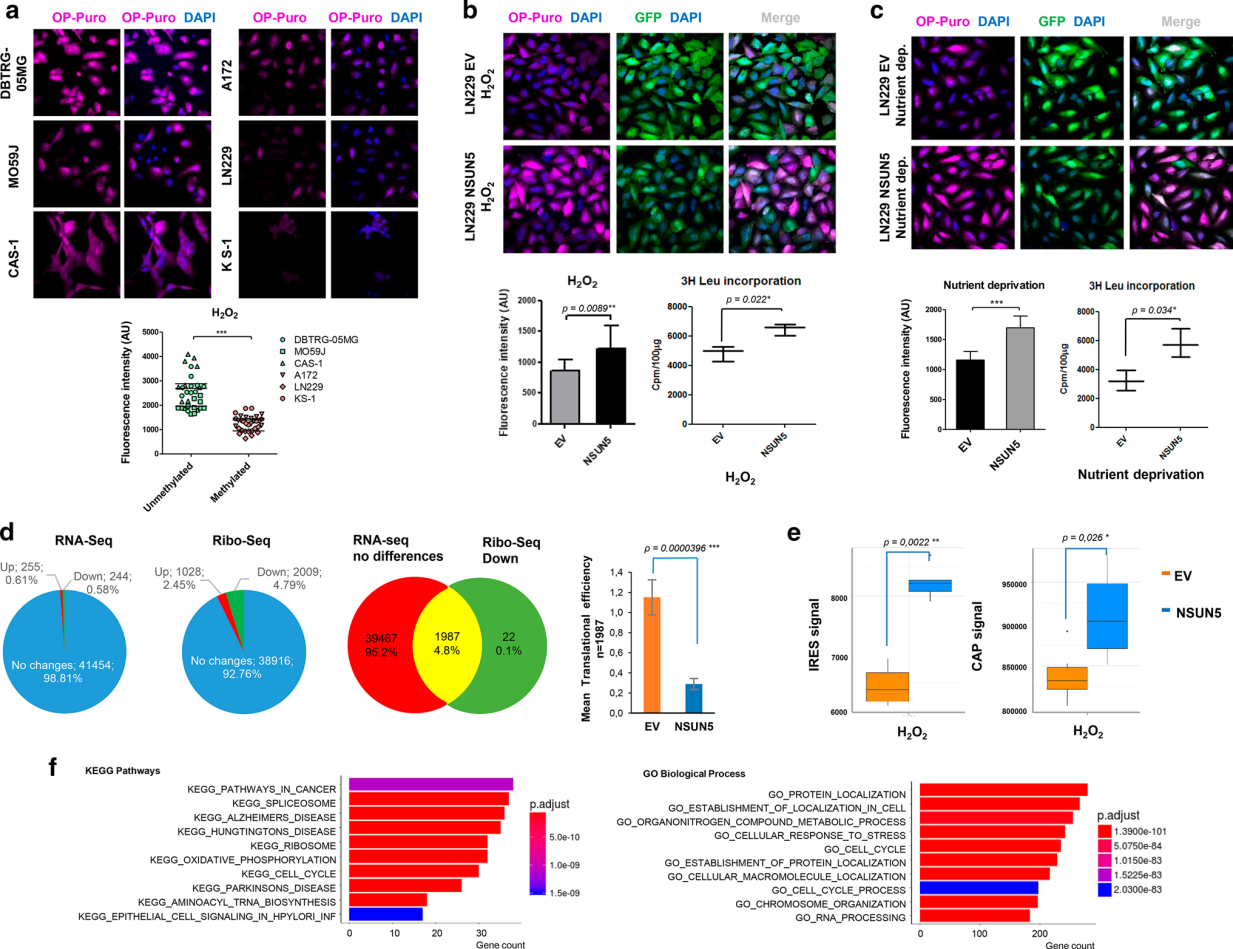
NSUN5 epigenetic loss is associated with depletion of global protein synthesis and the emergence of a stress-response translational program. **a** NSUN5 unmethylated glioma cell lines DBTRG-05MG, MO59J, and CAS-1 show higher overall protein synthesis assessed by OP-Puro under oxidative stress (100 mM H_2_O_2_) than the NSUN5 methylated cells (A172, LN229 and KS-1). **b** Restoration of NSUN5 function by transfection in epigenetically inactive LN299 cells increases overall protein synthesis under oxidative stress (100 mM H_2_O_2_) assessed by OP-Puro. Enhancement of global protein synthesis upon NSUN5 recovery in LN299 cells is also observed by the [3H] leucine incorporation assay. **c** Similar results were obtained upon nutrient deprivation. **d** Comparison of the total RNA (RNA-seq) and ribosome-protected RNA (Ribo-seq) deep-sequencing profiles to identify those RNAs with enhanced translational efficiency in NSUN5 deficient cells. 1987 RNAs that did not change in the RNA-seq of LN229 cells upon NSUN5-transfection were upregulated in the Ribo-seq of empty-vector-transfected cells indicating enhanced translational efficiency. **e** NSUN5 affects both CAP-dependent and CAP-independent translation according to the use of a reporter plasmid encoding for Firefly (IRES) and Renilla (CAP) luciferases. **f** Gene set enrichment analysis (GSEA) of the RNAs with increased translational efficiency in NSUN5 deficient cells (hypergeometric test with a FDR adjusted *P* value < 0.05)

We did not observe major effects in ribosome distribution, rRNA maturation and total rRNA levels comparing empty-vector and NSUN5-transfected LN229 cells (**Suppl. Fig. S5d and e**); thus, we wondered about more specific ways that NSUN5 and C3782 loss could affect protein levels in cancer cells. Although global translation rates are generally increased in cancer cells [45], tumors may encounter hostile microenvironments during their growth due to various conditions such as hypoxia, nutrient deprivation, and genotoxic stress [24]. One way that cancer cells survive in such stress settings is to reduce overall translation, thereby preventing this highly energy-consuming process and avoiding the accumulation of damaged and misfolded proteins [45]. Thus, we wondered whether the loss of the methylated site in the 28S rRNA mediated by NSUN5 epigenetic inactivation in glioma cells could act in this manner. To measure global protein synthesis, we quantified the incorporation of O-propargyl–puromycin (OP–Puro) into nascent proteins [5] in our experimental models. Using H_2_O_2_ in the medium to produce oxidative stress, we found that the three glioblastoma cell lines harboring NSUN5 epigenetic loss (LN229, A172, and KS1) show overall lower global protein synthesis than the three NSUN5 unmethylated cell lines (DBTRG-05MG, MO59 J and CAS-1) (Fig. 5a). Importantly, the transfection-mediated recovery of NSUN5 expression in the NSUN5 epigenetically deficient LN229 cells significantly increased global protein synthesis (Fig. 5b). Upon another stress condition, nutrient deprivation, the NSUN5 epigenetically deficient LN229 cells transfected with the empty vector also showed low overall level of protein synthesis, whereas the transfection-mediated recovery of NSUN5 expression significantly increased global protein synthesis (Fig. 5c). Similar results were obtained using [3H] leucine incorporation to measure global protein synthesis: the restoration of NSUN5 activity in the epigenetically inactivated LN229 cell significantly enhanced [3H] leucine incorporation and, thereby, global protein synthesis (Fig. 5b, c). NSUN5 shRNA-mediated downregulation in the NSUN5 unmethylated CAS-1 cell line had the opposite effect (Suppl. Fig. S5f). Overall, the results indicate that the transcriptional inactivation of NSUN5 by promoter CpG island and the mediated loss of methylated C3782 in 28S rRNA were associated with a restriction of overall protein synthesis.

### NSUN5 epigenetic loss promotes a specific mRNA translational program for stress survival

It is appropriate to mention that, in addition to the inhibition of global protein synthesis described above, human tumors manifest another method for coping with cellular stress: the activation of alternative translational programs that are involved in the adaptive response to stress [24, 45]. To identify the existence of a candidate translational program that directly depends on the loss of C3782 28S rRNA methylation upon NSUN5 epigenetic inactivation, we performed both RNA-Seq and Ribo-seq analyses comparing empty-vector and NSUN5-transfected LN229 cells in the absence of H_2_O_2_ stress. Glioma cells with restored NSUN5 expression presented RNA abundance differences in less than 2% transcripts (255 upregulated and 244 downregulated) remaining the 98% transcript abundance unchanged (absolute log2 fold change > 2) (Fig. 5d).

On the other hand, we analyzed the differences in the ribosome occupancy for the same conditions. We observed 7.2% transcripts (1028 over-occupied and 2009 down-occupied) with ribosome profiling differences (*P* value < 0.01 and absolute log2 fold change > 0.5) (Fig. 5d). RNAs with enhanced translational efficiency were defined as those unaltered in the RNA-seq, but upregulated in Ribo-seq [5, 51]. We identified 1987 transcripts with greater translation efficiency in the LN229 empty-vector-transfected cells (harboring NSUN5 epigenetic silencing) in comparison with NSUN5-transfected cells (Fig. 5d, Suppl. Table S1). We confirmed these observations by calculating translation efficiency values from over-dispersion analysis of both RNA-seq and Ribo-seq measurements by applying a general linear model (GLM) (Suppl. Methods).

We observed no over-representation of known regulatory motifs in the 5′-untranslated regions of these NSUN5-controlled transcripts, which included CAP-independent elements, such as internal ribosome entry sites (IRES), or CAP-dependent elements such as G-quadruplexes, cytosine-enriched regulator of translation (CERT), terminal oligopyrimidine tract (TOP), pyrimidine-rich translational sequences (PRTE) and upstream Open Reading Frames (uORFs) (*P* value = 0.8722 Welch Two sample *t* test) (Suppl. Fig. S6a). The use of a bicistronic reporter plasmid encoding both Firefly (IRES) and Renilla (CAP) luciferases further demonstrated the effect of NSUN5 on CAP-dependent and CAP-independent translation (Fig. 5e). To better characterize the described set of 1987 RNAs with significantly higher translational efficiency in NSUN5 epigenetically deficient cells, we developed a gene functional annotation by performing gene set enrichment analysis (GSEA). Using KEGG and GO signature collections, we observed an over-representation of cellular pathways and biological terms related to cancer, cell cycle, and the ribosome. Following the GSEA and sequence motifs recognition analysis described in “Materials and methods”, we applied a hypergeometric test with a FDR adjusted *P* value < 0.05 over the TE candidate genes using DAVID and GSEA signature database collections. Most strikingly, we noted an enhancement of ontologies related to cellular response to stress and metabolic adaptation (Fig. 5f).

**Fig. 6:**
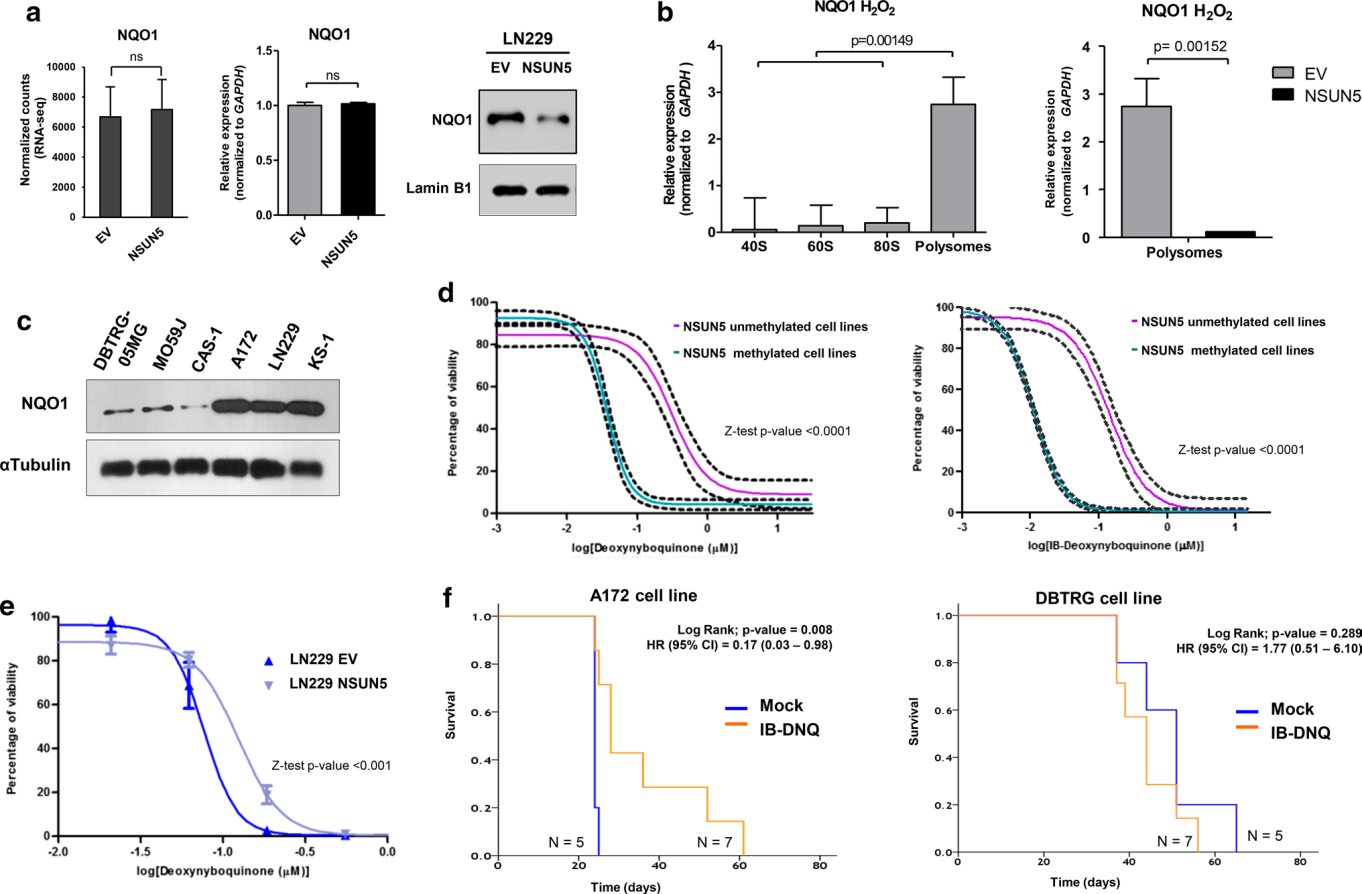
NSUN5 epigenetic silencing activates stress-related protein and confers growth inhibition sensitivity to NQO1-targeting molecules. **a** Validation of NSUN5 translational regulation of the identified stress-related target NQO1 expression at the RNA level determined by RNA-seq counts (left) and real-time quantitative PCR (middle) do not change upon NSUN5 transfection, but NQO1 expression decreased at the protein level (right) upon NSUN5 restoration. **b** qPCR shows enrichment of the NQO1 transcript in the polysome fraction of the empty-vector LN229 cells in comparison to NSUN5 transfected-LN229 cells. **c** NQO1 expression levels in glioma cell lines determined by western blot according to NSUN5 methylation status. **d** IC50 determination using the SRB assay in the glioma cell lines grouped by NSUN5 methylation status. Black dashed curves represent the 95% confidence band for each group. Glioma cells harboring NSUN5 methylation-associated NQO1 overexpression (A172, LN229 and KS-1) show increased sensitivity to deoxynyboquinone (DNQ) and IB-DNQ in comparison to NSUN5 unmethylated cells (DBTRG-05MG, MO59 J, and CAS-1). Drug-response curves were generated using GraphPad Prism software and analyses were performed with the drc R package. For each cell line and the drug, we fit a four-parameter generalized log-logistic model. Comparison of the IC50 values calculated from the slopes were obtained by means of a *z* test (*P* < 0.0001). **e** IC50 determination using the SRB assay in NSUN5-transfected LN299 cells in comparison with empty-vector-transfected cells (EV) shows enhanced resistance to DNQ-mediated growth inhibition in the cells with restored NSUN5 expression. Comparison of the IC50 values calculated from the slopes was obtained by means of a *z* test (*P* < 0.001). **f** Kaplan–Meier analysis of Survival according treatment conditions (IB-DNQ treated vs mock group) in a set of mice models with implanted tumors derived from the NSUN5 methylated cell line A172 (left) or the NSUN5 unmethylated cell line DBTRG (right). Significance of the log-rank test is shown. Results of the univariate Cox regression analysis are represented by the hazards ratio (HR) and 95% confidence interval (CI)

To confirm this scenario, in which NSUN5 epigenetic silencing promotes a global reduction in protein synthesis, but a specific protein production program emerges that is associated with enhanced translational efficiency to deal with cellular stress, we carried out stable isotopic labeling of amino acids in cell culture (SILAC) in empty-vector-transfected LN229 cells in comparison with NSUN5 stably transfected LN229 cells. At the level of resolution employed in the SILAC analyses, 128 proteins were identified that had significantly different expression upon NSUN5 transfection in LN229 cells (Suppl. Table S2). With the aim to improve the characterization of the proteins with significantly different expression upon NSUN5 transfection, we developed a gene functional annotation by performing gene set enrichment analysis (GSEA). Following the GSEA and sequence motifs recognition analysis described in “Materials and methods”, we applied a hypergeometric test with an FDR adjusted *P* value < 0.05 over the candidate genes using DAVID and GSEA signature database collections. Using KEGG and GO signature collections, we observed an over-representation of cellular pathways and biological terms related to cell signaling, response to stress by mismatch repair and RNA processing (Suppl. Fig. S5g). Most proteins (73%; 94 of 128) were found to be upregulated upon restoration of NSUN5, whereas the remaining 27% (34 of 128) were downregulated (Suppl. Table S2). Western-blot validation of three downregulated proteins upon NSUN5 transfection is shown in Suppl. Fig. S5h. Thus, these data are consistent with the idea that the loss of NSUN5 in glioma cells restricts general protein synthesis. They also suggest the existence of a selective synthesis of specific proteins in the context of NSUN5 deficiency. In this regard, 26% (9 of 34) of these proteins were derived from RNAs that showed increased translational efficiency upon NSUN5 loss (Fig. 5d). We selected one of these targets, the stress-activated protein NQO1 [9], for western-blot and quantitative reverse transcription–PCR analyses, which confirmed that its NSUN5 regulation occurred at the mRNA translational level and was not associated with a difference in RNA levels (Fig. 6a). In addition, NQO1 was enriched in the polysome fraction of the empty-vector LN229 cells (where NSUN5 is epigenetically silenced) in comparison with NSUN5-transfected LN229 cells (Fig. 6b). Further development of additional RNA-seq experiments comparing empty-vector and NSUN5-transfected LN229 cells, but in the presence of H_2_O_2_ stress, confirmed that NSUN5 transfection had almost no effect in the transcriptome, remaining 99.5% of the transcripts unchanged (Suppl. Table S3). Ribo-seq analysis in these cells, undergoing H_2_O_2_ stress, further confirmed that NQO1 showed greater translation efficiency in the LN229 empty-vector-transfected cells (having NSUN5 epigenetic loss) in comparison with NSUN5-transfected cells (Suppl. Table S1). We also performed RNA-seq in the absence of H_2_O_2_ in another glioblastoma cell line, A172, that it also shows NSUN5 epigenetic inactivation obtaining near identical results: 99% of transcripts levels were unchanged upon NSUN5 transfection, including NQO1 (Suppl. Table S3).

The above-described scenario was also validated in loss-of-function experiments. To identify the existence of a candidate translational program that directly depends on NSUN5 loss, we performed both RNA-Seq and Ribo-seq analyses in NSUN5 shRNA-depleted vs scramble shRNA DBTRG-05MG cells. Glioma cells with depleted NSUN5 expression presented RNA abundance differences in less than 1% transcripts, remaining the 99% transcript abundance unchanged (absolute log2 fold change > 2) (**Suppl. Fig. S6b and Suppl. Table S4**), whereas for ribosome occupancy, we observed a 2.2% transcripts with ribosome profiling differences (absolute log2 fold change > 0.5) (**Suppl. Fig. S6b and Suppl. Table S4**). RNAs with enhanced translational efficiency were defined as those unaltered in the RNA-seq, but upregulated in Ribo-seq [5, 51]. We identified 787 transcripts with greater translation efficiency in NSUN5 shRNA-depleted DBTRG-05MG cells in comparison with scramble shRNA cells (**Suppl. Fig. S6b and Suppl. Table S4**). We confirmed these observations by calculating translation efficiency values from over-dispersion analysis of both RNA-seq and Ribo-seq measurements by applying a general linear model (GLM) (**Suppl. Methods**).

With the aim to improve the characterization of the described set of 787 RNAs with significantly higher translational efficiency in NSUN5 shRNA-mediated deficient cells, we developed a gene functional annotation by performing gene set enrichment analysis (GSEA). Using KEGG and GO signature collections, we observed an over-representation of cellular pathways and biological terms related to cancer and the ribosome. Following the GSEA and sequence motifs recognition analysis described in “Materials and methods”, we applied a hypergeometric test with an FDR adjusted *P* value < 0.05 over the TE candidate genes using DAVID and GSEA signature database collections. As we had previously described for the LN229 model (Fig. 5f), we noted an enhancement of ontologies related to cellular response to stress and metabolic adaptation (**Suppl. Fig. S6c**). NQO1, that was characterized above as having an enhanced translational efficiency (**Suppl. Table S1** and Fig. 6a, b) and overexpression (**Suppl. Table S2** and Fig. 6a) in LN229 cells with NSUN5 epigenetic loss, was also identified as a target of enhanced translational efficiency in DBTRG-05MG cells with shRNA-mediated depletion of NSUN5 (**Suppl. Table S4**). Western-blot and quantitative reverse transcription–PCR analyses confirmed that NQO1 upregulation at the protein level in NSUN5 depleted cells was not associated with a difference in RNA levels (**Suppl. Fig. S6d**). Altogether, these data are consistent with the idea that the loss of NSUN5 in glioma cells restricts general protein synthesis while activating the selective synthesis of specific stress-related proteins such as NQO1.

### NSUN5 DNA methylation-associated silencing confers sensitivity to drugs that target NQO1

The finding that NSUN5 epigenetic silencing was associated with NQO1 overexpression prompted us to study whether these glioma cells might be more vulnerable to compounds targeting this particular stress-related protein. Drugs that generate reactive oxygen species have been suggested to be useful for pushing cancer cells over an oxidative stress threshold and into cell death [15]. In this regard, deoxynyboquinone (DNQ) and isobutyl–deoxynyboquinone (IB-DNQ) are NQO1 bioactivatable substrates that cause the production of large amounts of ROS that cannot be tolerated by the cancer cells, leading to their death [19, 27, 30]. Thus, we assessed whether an enhanced growth inhibitory effect of these compounds was dependent on NSUN5 methylation. First, we demonstrated by western-blot analyses that the three studied glioma cell lines with NSUN5 hypermethylation (LN229, A172, and KS-1) showed high levels of NQO1, whereas minimal expression of NQO1 was observed in the three NSUN5 unmethylated cell lines (DBTRG-05MG, MO59 J, and CAS-1) (Fig. 6c). These data confirmed the above-described role of NSUN5 expression in the regulation of NQO1 protein levels (Fig. 6a). Most importantly, the determination of the corresponding IC50 values, according to the SRB assay for cell viability, showed that NSUN5 hypermethylated glioma cells were significantly more sensitive to the DNQ and IB-DNQ drugs than the NSUN5 unmethylated glioma cell lines (*z* test, *P* < 0.0001) (Fig. 6d). Furthermore, we extended these assays to NSUN5 methylated LN229 glioma cells, where we have exogenously restored NSUN5 activity and thus depleted the NQO1 protein (Fig. 6a). We observed that upon NSUN5 transfection the cells become significantly more resistant to the NQO1 bioactivatable substrate in comparison to empty-vector-transfected cells (*z* test, *P* < 0.001) (Fig. 6e). Similar results were also found in vivo for cancer cells injected orthotopically in the brain of nude mice (Fig. 6f). The use of the NQO1 bioactivatable substrate increased survival in those nude mice with tumors originated from NSUN5 hypermethylated A127 glioma cells in comparison with the mock-treated group (Fig. 6f), whereas no differences in survival between drug and mock treatment were observed for mice carrying tumors derived from the NSUN5 unmethylated cell line DBTRG-05MG (Fig. 6f). Thus, the occurrence of NSUN5 methylation pinpoints glioma cells that are more sensitive to the cytotoxic effect of NQO1 substrates.

### NSUN5 epigenetic loss is a hallmark of human primary gliomas with good clinical outcome

**Fig. 7:**
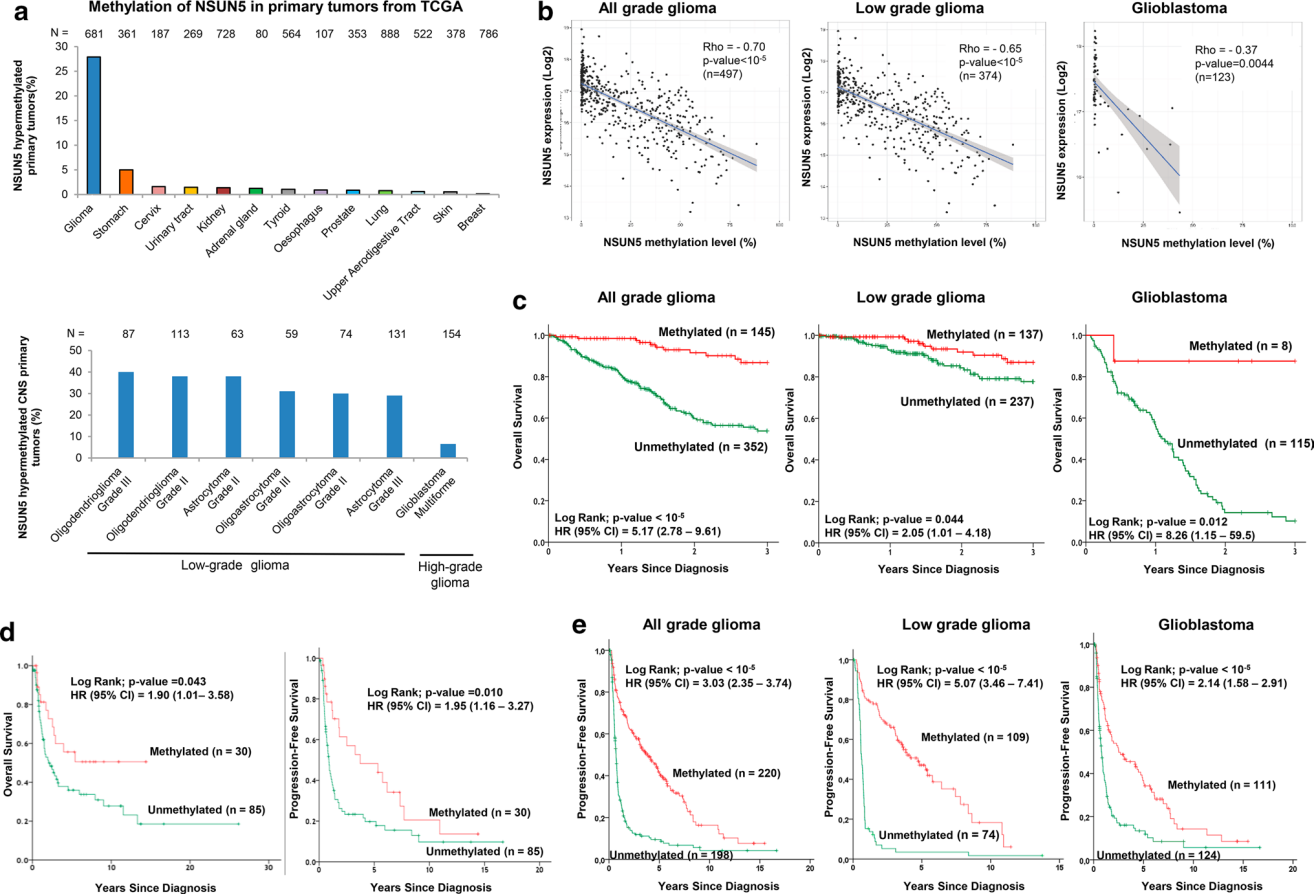
NSUN5 epigenetic inactivation occurs in human primary gliomas in association with good clinical outcome. **a** Percentage of NSUN5 methylation in the TCGA data set of primary tumors according to cancer type (top) and according to the cellular grade of the glioma (down). **b** NSUN5 promoter CpG island methylation is associated with the loss of the transcript among all cellular grades in primary glioma, in low-grade glioma and in glioblastoma. **c** Kaplan–Meier analysis of overall survival (OS) across overall glioma grades and in low- and high-grade glioma with respect to NSUN5 methylation status. Significance of the log-rank test is shown. Results of the univariate Cox regression analysis are represented by the hazards ratio (HR) and 95% confidence interval (CI). **d** Kaplan–Meier analysis of OS in IDH1 wild-type gliomas according to NSUN5 methylation status. **e** Kaplan–Meier analysis of OS in gliomas without 1p/19q deletion according to NSUN5 methylation status. **f** Kaplan–Meier analysis of OS in gliomas with unmethylated MGMT according to NSUN5 methylation status. For all graphs, the *P* value corresponds to the Log-Rank test. Cox regression univariate analysis is represented by HR with a 95% CI

Once we had determined in glioma cell lines and mouse models the effects of NSUN5 epigenetic silencing, we studied the impact of NSUN5 promoter CpG island hypermethylation in human primary gliomas. The analyses of the collection of human gliomas available from The Cancer Genome Atlas (TCGA) (https://tcga-data.nci.nih.gov/tcga/), which used the same DNA methylation microarray platform as that used here for our initial Sanger cell line screening, showed the presence of NSUN5 promoter CpG island methylation in 28% (190 of 681) of gliomas of different grades (Fig. 7a and **Suppl. Table S5**). TCGA RNA-sequencing data showed that NSUN5 hypermethylation was associated with transcript downregulation across glioma samples (Spearman’s rank correlation, *ρ* = − 0.7, *P* < 10^−5^) (Fig. 7b). The histological grade based on the degree of anaplasia, as set out in the World Health Organization (WHO) Classification of Tumors of the Central Nervous System, has a major effect on the natural history of the disease. Low-grade gliomas (LGGs; grades I, II, and III) have a more favorable prognosis, whereas the high-grade gliomas (grade IV), also known as glioblastoma multiforme (GBM), have an extremely unfavorable outcome [32, 50]. We observed the enrichment of NSUN5 hypermethylation in low-grade gliomas (34%, 180 of 527) relative to GBM (6%, 10 of 154) (Fisher’s exact test, *P* = 3.8 10^−9^) (Fig. 7a). TCGA RNA-sequencing data showed NSUN5 hypermethylation to be associated with transcriptional downregulation in low- and high-grade gliomas (Fig. 7b). Beyond adult gliomas, we also detected NSUN5 hypermethylation in pediatric brain tumors, such as diffuse intrinsic pontine glioma (DIPG) (50%, 5 of 10) and medulloblastoma (25%, 2 of 8). Importantly, we were able to obtain RNA from three DIPG tumors, where we observed that the two NSUN5 hypermethylated cases showed hypomethylation of the C3782 position of the 28S rRNA, while the NSUN5 unmethylated patient displayed high levels of rRNA methylation at this site (Suppl. Fig. S6e and f).

Given the known relation between glioma grade and clinical outcome, we wondered whether NSUN5 hypermethylation also conferred any prognostic value. We observed that NSUN5 hypermethylation was associated with increased overall survival (OS) in all glioma grades in the TCGA cohort [log-rank; *P* < 10^−5^; hazard ratio (HR) = 5.17, 95% CI = 2.78–9.61] (Fig. 7c). Interestingly, the value of NSUN5 hypermethylation as a predictor of better outcome was observed for low- and high-grade gliomas (Fig. 7c). NSUN5 hypermethylation was associated with higher OS in LGG (log-rank; *P* = 0.044; HR = 2.05; 95% CI = 1.01–4.18) and GBM (log-rank; *P* = 0.012; HR = 8.26; 95% CI = 1.15–59.50) (Fig. 7c). These data are particularly relevant in the context of GBM because of its dismal outcome in 90% of patients. However, the remaining 10% of individuals are long-term survivors, for whom the molecular factors involved have not been fully identified [32, 50].

Low levels of the NSUN5 RNA transcript were also associated with increased OS in the TCGA cohort (log-rank; *P* < 10^−5^; HR = 4.57; 95% CI = 2.40–9.04) (Suppl. Fig. S7a). Upon division by grade, low levels of NSUN5 RNA transcript were also associated with increased OS in LGG (log-rank; *P* = 0.014; HR = 2.37; 95% CI = 1.16–4.84) (Suppl. Fig. S7b). A trend between low expression of NSUN5 and shorter OS was found in GBMs, but due to the small number of cases with low expression (*n* = 2) according to the established cutoff, it lacked statistical power (Suppl. Fig. S7c).

We confirmed the TCGA glioma findings in a newly obtained validation cohort of 115 primary gliomas (Suppl. Table S5), where NSUN5 methylation status was determined by the pyrosequencing assay: NSUN5 promoter hypermethylation was associated with and increased OS (log-rank; *P* = 0.043; HR = 1.90; 95% CI = 1.01–3.58) and extended progression-free survival (PFS) (log-rank; *P* = 0.010; HR = 1.95; 95% CI = 1.16–3.27) (Fig. 7d). We further expanded this initial validation cohort with the inclusion of two independent glioma cohorts of 303 patients, where NSUN5 methylation status is available from the same DNA methylation microarray used in the TCGA glioma cases [1, 22]. Suppl. Table S5 shows the clinicopathological and molecular characteristics of the overall extended validation cohort. Related to clinical outcome in this large validation cohort, only PFS was available from the three set of cases (pyrosequencing plus the two data-mined cohorts), and thus, it was the assessed variable. We confirmed that NSUN5 promoter hypermethylation was associated with increased PFS (log-rank; *P* value 10^−5^; HR = 3.03; 95% CI = 2.35–3.74) (Fig. 7e). Importantly, the impact of NSUN5 methylation on PFS is maintained when the glioma validation samples are divided into grades. NSUN5 methylation is associated with extended PFS in low-grade gliomas (LGG, *n* = 183) (log-rank; *P* value 10^−5^; HR = 5.07; 95% CI = 3.46–7.41) (Fig. 7e) and glioblastoma (GBM, n = 235) (log-rank; *P* value 10^−5^; HR = 2.14; 95% CI = 1.58–2.91) (Fig. 7e).

We examined whether other genetic and epigenetic alterations of clinical relevance in glioma [32, 50] helped define patients with NSUN5 methylation-associated extended survival. In this regard, the presence of IDH1 mutations in gliomas is known to be a predictor of improved outcome [32, 50]. We found this to be the case for the gliomas included in the TCGA repository: IDH1 mutations are associated with longer OS (**Suppl. Fig. S8a**). Interestingly, for those cases with a wild-type IDH1, which are all supposed to have an adverse prognosis, the presence of NSUN5 hypermethylation highlighted a subset of cases with long OS (Suppl. Fig. S8a). A similar clinical observation than the one observed for IDH1 mutants was found related to the co-deletion of chromosome arms 1p and 19q, another predictor of good outcome [32, 50], that it was also associated with longer OS in the gliomas from the TCGA repository (Suppl. Fig. S8b). Importantly, for those patients without 1p/19q co-deletion, which are all expected to have a poor outcome, NSUN5 hypermethylation pinpointed a subgroup with improved OS (Suppl. Fig. S8b). Interestingly, an additional complementary molecular scenario was discovered related to another key marker in human glioma that is also used in clinical practice: the DNA repair enzyme MGMT [32, 50]. Epigenetic inactivation of MGMT is linked to an enhanced response to chemotherapy-alkylating agents and increased OS in gliomas [10, 17]. We found this to be the case for the TCGA gliomas: MGMT hypermethylation is associated with longer OS (Suppl. Fig. S8c). In addition, here again, for those gliomas unmethylated at MGMT, which are supposed to show poor outcome, NSUN5 hypermethylation defined a subset with extended OS (Suppl. Fig S8c). The above-described TCGA results were mostly confirmed in our expanded glioma validation cohort. First, in this set of cases, we confirmed that the IDH1 mutation, the co-deletion of chromosome arms 1p and 19q and MGMT hypermethylation were all associated with longer PFS (Suppl. Fig. S8d, e, and f). Importantly, for those patients without 1p/19q co-deletion, NSUN5 hypermethylation pinpointed a subgroup with improved PFS (Suppl. Fig. S8e). Related to IDH1 mutational status, we observed a trend between NSUN5 hypermethylation and longer PFS in the cases with a wild-type IDH1 that did not reach statistical significance (Suppl. Fig. S8d). Interestingly, NSUN5 hypermethylation defined a subset with extended PFS for both MGMT unmethylated and hypermethylated patients (Suppl. Fig. S8f).

For both TCGA and validation cohorts, we observed that NSUN5 methylation was associated with other biomarkers of clinical benefit such as IDH1 mutation (Fisher’s exact test *P* < 10^−5^), co-deletion of 1p/19q (Fisher’s exact test *P* < 10^−5^), and MGMT methylation (Fisher’s exact test *P* < 10^−5^) (Suppl. Table S5). Importantly, all these three last molecular markers are also significantly associated between them (IDH1 mutation/co-deletion 1p/19q, Fisher’s exact test *P* < 10^−5^; IDH1 mutation/MGMT methylation, Fisher’s exact test *P* < 10^−5^; co-deletion 1p/19q/MGMT methylation, Fisher’s exact test *P* < 10^−5^) (Suppl. Table S6). It is also worth to mention that NSUN5 methylation was not associated with the glioma CpG island methylator phenotype (gCIMP) (Fisher’s exact test, *P* = 0.094) (Suppl. Table S5) (Suppl. Methods), a common phenomenon in IDH1 mutant patients [29]. As expected, the presence of the gCIMP phenotype in the TCGA glioma data set is associated with improved clinical outcome (Suppl. Fig. S9a). Strikingly, when we stratified the TCGA data set according to gCIMP status and NUSN5 methylation, we observed that for those cases with a lack of the gCIMP phenotype, which are all supposed to have a poor clinical outcome, the presence of NSUN5 hypermethylation highlighted a subset of cases with long overall survival (Suppl. Fig. S9b).

**Fig. 8:**
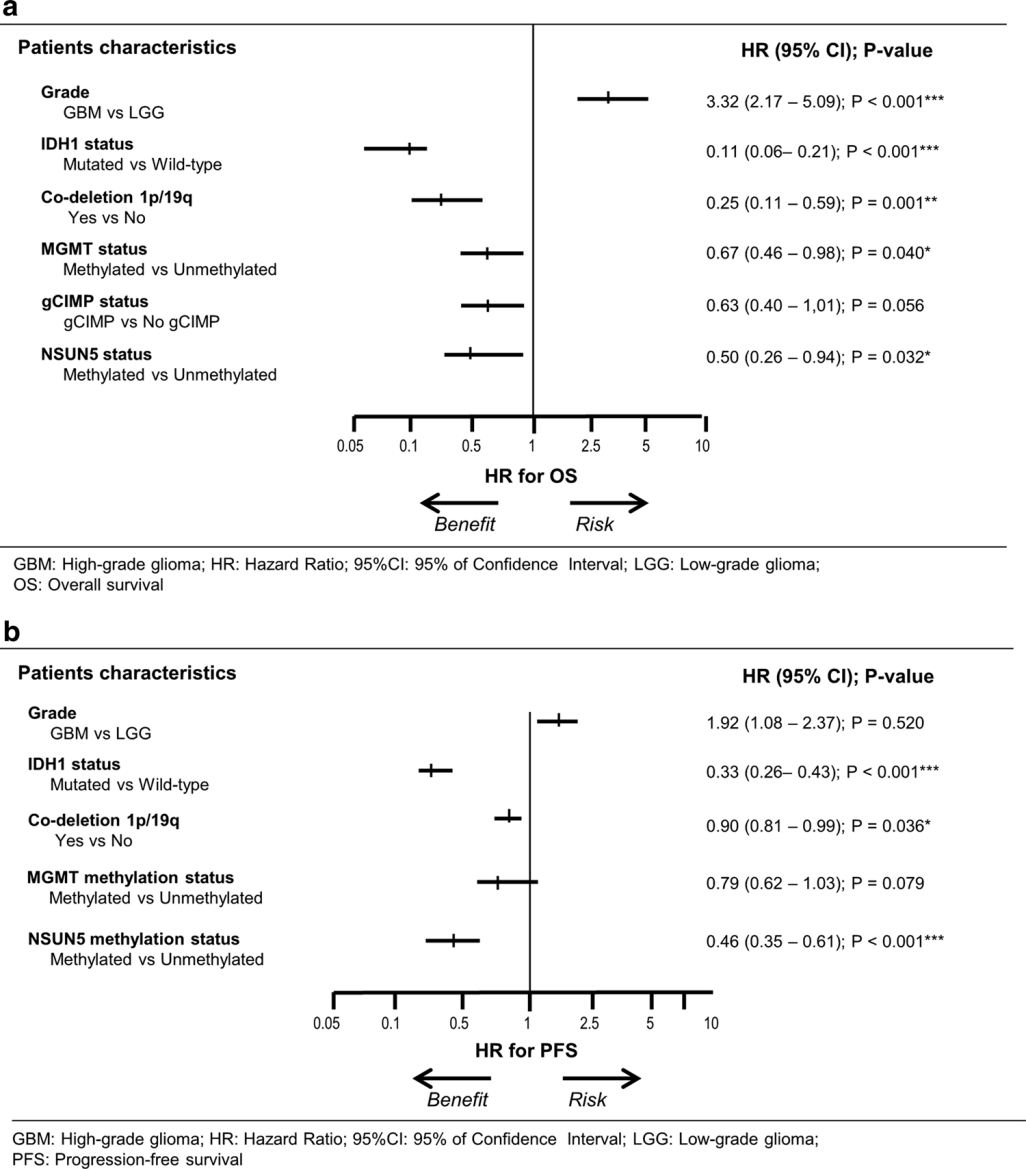
Forest plots of the multivariable Cox regression analysis for clinical outcome in the glioma cohorts studied for NSUN5 methylation status taking into account different prognostic factors. *P* values (*P*) correspond to hazard ratios (HR), with a 95% of confidence interval (95%CI), associated with OS. Co-variables with associated *P* value under 0.05 were considered as independent prognostic factor (*P < 0.05, **P < 0.01, ***P < 0.001). **a** Overall Survival (OS) multivariate Cox regression analysis in the TCGA set of glioma patients (*n* = 497). **b** Progression-free survival (PFS) multivariate Cox regression analysis in the expanded validation set of glioma patients (*n* = 418)

Finally, and most importantly, to determine the value of NSUN5 methylation as a candidate independent biomarker, we performed multivariate analysis for NSUN5 methylation status and the described known biomarkers of clinical outcome (IDH1 mutational, 1p/19q co-deletion and MGMT methylation status) for the both studied glioma cohorts. Multivariate Cox regression analysis showed that NSUN5 hypermethylation was an independent predictor of OS in the TCGA glioma cohort (HR = 0.50; 95% CI = 0.26–0.94; *P* = 0.032) (Fig. 8a) and an independent predictor of PFS in the interrogated validation cohort of glioma patients (HR = 0.46; 95% CI = 0.35–0.61; *P* < 0.001) (Fig. 8b).

## Discussion

More than 100 distinct nucleotide modifications have been reported in the RNA molecule across the range of all life forms [10, 11, 27], many of which are also present in human RNA molecules such as N6-methyladenosine, N1-methyladenosine, pseudouridine, or 5-methylcytosine [10, 11, 27]. The chemical modifications of RNA, referred to in a global context as the epitranscriptome, carry out functions critical to cellular homeostasis, contributing to RNA stability, export, localization, structure, splicing, processing, recoding, targeting, and translation [8, 11, 14, 34]. We are at the dawn of our understanding of how disruption of the epitranscriptome can initiate and promote tumorigenesis, but the emerging findings indicate that altered RNA modification patterns play a fundamental role in cancer.

The occurrence of mutations in the pseudouridine synthase DKC1 in X-linked dyskeratosis congenita (X-DC), a disorder characterized by cancer susceptibility [18], is particularly interesting, because it directly affects a key cellular element, the ribosome. DKC1 is the pseudouridine synthase that mediates the introduction of this modified base in ribosomal RNA [37, 53]. Mice that are hypomorphically mutant for DKC1 exhibit decreased pseudouridine in their RNAs and a defect in translation that particularly affects mRNAs containing Internal Ribosome Entry Sites (IREs) [3, 4, 18, 21, 53]. Translational control is also impaired in human X-DC patients [3, 53]. These results indicate that ribosomes are not passive structures in cellular transformation, but actively contribute to the phenotype of cancer cells. This idea is also supported by the existence of specialized ribosomes and complex machineries associated with these structures and rRNAs [41, 42], in addition to the presence of mutations in ribosomal proteins in cancer-associated syndromes [7]. Significantly for rRNA, in addition to pseudouridine, another modification, that of 5-methylcytosine, seems to have a central regulatory role [31]. This RNA chemical modification also acts at the level of tRNAs, where loss of the 5-methylcytosine RNA-methyltransferase NSUN2 drives a global reduction in protein synthesis [5, 6, 34] and altered translational programs [5, 6], in a similar manner to that which occurs upon the loss of DKC1 [3, 4, 37, 53].

In this study, we combined genomic, epigenomic, and epitranscriptomic approaches to characterize the 5-methylcytosine RNA methyltransferase, NSUN5, and showed how its epigenetic silencing produces a hypomethylation event at a particular position of the 28S rRNA that depletes the overall protein synthesis, but engages glioma cells in a particular translational program for survival under conditions of cellular stress. Interestingly, we also show by molecular dynamics that the methylation of the C3782 position of human 28S rRNA could mediate the structural stability of the tertiary complex rRNA–tRNA–mRNA, leading it loss to a structural distortion at the edge of the 28S subunit in the P-site cavity that might impair the physiological protein synthesis [28].

The observed “translational paradox”, the emergence of a translational program in the context of globally depleted protein production, is a well-known mechanism used by cells to exert a strong and timely influence on gene expression to deal with stress conditions [24]. As tumors progress, they encounter many obstacles and unfriendly environments that frequently produce oxidative, replicative, genotoxic, proteotoxic, or metabolic stress [45]. Even oncogenic mutations that promote tumorigenesis in the early stages subsequently become a nuisance if they generate high levels of ROS that are toxic to cancer cells [24]. One of the main adaptations of tumors to these conditions is the global inhibition of protein synthesis that shuts down a highly energy-demanding process and at the same time prevents the accumulation of misfolded or damaged proteins [45]. However, there is a simultaneous enabling of translation of selected mRNAs that are vital for the adaptive cell response [24]. Another illustrative case is provided by NQO1, a multifunctional antioxidant enzyme regulated by the Keap1/Nrf2/ARE pathway [9], whose translational efficiency increases upon the loss of NSUN5 in glioma, as reported here, to overcome the many types of oxidative stress encountered by these transformed cells. NSUN5 methylation-mediated overexpression of NQO1 can also constitute a therapeutic opportunity in these gliomas, because they become more sensitive to ROS-induced death upon the use NQO1-bioactivatable molecules [19, 27, 30]. Thus, NSUN5 DNA methylation-associated inactivation, through its linked defect in 28S rRNA methylation, represents a bona fide mechanism that explains how human gliomas adapt to challenging cellular stress conditions by depleting overall synthesis while also promoting specific translational programs. Furthermore, although we did not observe NSUN5 genomic defects in the studied models, more comprehensive studies are required to discard additional genetic alterations.

This role of NSUN5 epigenetic silencing in the toleration of cellular stress settings resembles another key molecular event in gliomagenesis: the occurrence of IDH1 mutations. We have demonstrated that the loss of NSUN5 activity extends cell viability in vivo and in vitro, but it is associated with improved clinical outcome. In a similar manner, the loss of IDH1 normal catalytic activity contributes to gliomagenesis, but it is also associated with good OS. Once again, we can consider how cancer cells avoid death when confronted by damaging stress conditions, because, as we have shown for NSUN5, the disruption of IDH1 is another way of dealing with metabolic and hypoxic stress [33]. Thus, those patients with gliomas exhibiting NSUN5 epigenetic silencing, like those carrying IDH1 mutations, have tumor cells that probably are on the verge of death, whose last resort in their struggle to survive is to restrict overall protein synthesis and to instigate particular emergency translational programs to deal with multiple stress conditions.

In conclusion, NSUN5 loss in glioma provides a link between an epigenetic event, the promoter CpG island hypermethylation of its promoter, and an epitranscriptomic event, the hypomethylated status of the C3782 position of the human 28S rRNA. Most significantly, we have shown that NSUN5 inactivation is a likely pathway used by glioma cells to overcome hostile stress conditions by restraining high energy-consuming global protein synthesis while stimulating the translation of adaptive proteins such as NQO1. These findings, if validated in larger sets of prospective clinical studies, might be useful for the management of glioma patients, because, in the context of a disease, whose prognosis is usually dismal, the NSUN5 epigenetic lesion contributes to identify those patients who are likely to have a good outcome.

## Electronic supplementary material

Below is the link to the electronic supplementary material.

Supplementary material 1 (PDF 7113 kb)

Supplementary material 2 (XLSX 1436 kb)
