# Supplementary material for: Epigenetic loss of RNA-methyltransferase NSUN5 in glioma targets ribosomes to drive a stress adaptive translational program
Source: Acta Neuropathol. 2019 Aug 19;138(6):1053–74. doi: 10.1007/s00401-019-02062-4 (PMC6851045; doi:10.1007/s00401-019-02062-4)
Supplement: Supplementary file 1 — Supplementary material 1 (PDF 7113 kb) [file 401_2019_2062_MOESM1_ESM.pdf]

Supplementary Material for:

Epigenetic Loss of RNA Methyltransferase NSUN5 in Glioma Targets Ribosomes to Drive a Stress Adaptive Translational Program

Maxime Janin,[1] Vanessa Ortiz-Barahona,[1] Manuel Castro de Moura,[1] Anna Martínez-Cardús,[1] Pere Llinàs-Arias,[1] Marta Soler,[1] Daphna Nachmani,[2] Joffrey Pelletier,[3] Ulrike Schumann,[4] Maria E. Calleja-Cervantes,[1] Sebastian Moran,[1] Sonia Guil,[1] Alberto Bueno-Costa,[1] David Piñeyro,[1] Montserrat Perez-Salvia,[1] Margalida Rosselló-Tortella,[1] Laia Piqué,[1] Joan J. Bech-Serra,[5] Carolina De La Torre,[5] August Vidal,[6,7] María Martínez-Iniesta,[8] Juan F. Martín-Tejera,[8] Alberto Villanueva,[8] Alexandra Arias,[9] Isabel Cuartas,[9] Ana M. Aransay,[10] Andres Morales La Madrid,[11,12] Angel M. Carcaboso,[13] Vicente Santa-Maria,[11,12] Jaume Mora,[11] Agustin F. Fernandez,[14] Mario F. Fraga,[15] Iban Aldecoa,[16] Leire Pedrosa,[17] Francesc Graus,[18] Noemi Vidal,[6] Fina Martínez-Soler,[19] Avelina Tortosa,[19] Cristina Carrato,[20] Carme Balañá,[21] Matthew W. Boudreau,[22] Paul J. Hergenrother,[22] Peter Kötter,[23] Karl-Dieter Entian,[23] Jürgen Hench,[24] Stephan Frank,[24] Sheila Mansouri,[25] Gelareh Zadeh,[25] Pablo D. Dans,[26] Modesto Orozco,[26,27] George Thomas,[3,28,29] Sandra Blanco,[30,31] Joan Seoane,[7,9,32] Thomas Preiss,[4,33] Pier Paolo Pandolfi,[2] Manel Esteller[1,7,27,32,34*]

Corresponding Author: Manel Esteller, Josep Carreras Leukaemia Research Institute (IJC), Ctra de Can Ruti, Camí de les Escoles s/n 08916 Badalona, Barcelona, Catalonia, Spain. Phone: 34-93-5572800; Fax: 34-93-4651472 E-mail: mesteller@carrerasresearch.org

[1]*Cancer Epigenetics and Biology Program (PEBC), Bellvitge Biomedical Research Institute (IDIBELL), L'Hospitalet, Barcelona, Catalonia, Spain.*

[7]*Centro de Investigacion Biomedica en Red Cancer (CIBERONC), Madrid, Spain.*

[27]*Department of Biochemistry and Biomedicine, University of Barcelona, 08028 Barcelona, Spain.*

[32]*Institucio Catalana de Recerca i Estudis Avançats (ICREA), Barcelona, Catalonia, Spain.*

[34]*Josep Carreras Leukaemia Research Institute (IJC), Badalona, Barcelona, Catalonia, Spain.*

Supplementary Methods

Supplementary Figures (S1 to S9)

Supplementary Tables (S1 to S6)

Key Resources Table

SUPPLEMENTARY METHODS

Cell lines

Human glioma cell lines DBTRG-05MG, MO59J, A172 and LN229 were purchased from the American Type Culture Collection; CAS-1 was obtained from The Biological Bank of the IRCCS Azienda Ospedaliera Universitaria San Martino – IST Istituto Nazionale per la Ricerca sul Cancro; and KS-1 was purchased from the Japanese Collection of Research Bioresources Cell Bank. Cell lines were all cultivated in Dulbecco's Modified Eagle's Medium (DMEM) supplemented with 10% v/v fetal bovine serum (FBS) (Innovative, Novi, MI), and 1% v/v penicillin/streptomycin (Gibco). Human grade III glioma cell lines SW1088 and BT142 mut/- were purchased from the American Type Culture Collection and MOG-G-CCM was purchased from Sigma-Aldrich. These cell lines were cultivated regarding to suppliers recommendations. All cell lines were authenticated by short tandem repeat profiling (LGS Standards SLU) and tested for the absence of mycoplasma.

DNA methylation analyses

DNA methylation status of the 5'-end promoter-associated CpG island of the NSUN5 gene was determined by bisulfite genomic sequencing and DNA methylation microarrays. DNA was extracted from cell lines and brain white matter pellets by the addition of 4.5 mL of lysis buffer (10 mM Tris, 5 mM EDTA, 100 mM NaCl, pH 8), and re-suspended with 500 µL of SDS 10% and 50 µg of proteinase K at 10 mg/mL. Samples were incubated overnight at 37°C. After addition of 2.5 mL of NaCl 5M and vortex, samples were centrifuged at 4000 RPM during 15 minutes. Upper phases were collected and 5.6 mL of isopropanol were added to precipitate DNA. The DNA was recovered and washed by ethanol 70%. After re-suspension in water, all DNA samples were quantified by NanoDrop. For the 9 cell lines and the brain white matter, we performed bisulfite conversion of 500 ng of genomic DNA using an EZ DNA Methylation Gold kit (ZymoResearch, Orange, CA, USA). For bisulfite genomic sequencing primer sequences please refer to the Key Resources Table. Primers (from Sigma-Aldrich) were designed by MethPrimer (Peking Union Medical College Hospital (PUMCH), Chinese Academy of Medical Sciences). After PCR, the amplicon product (purified using a Macherey-Nagel purification kit) was ligated into pGEM®-T Easy vector (Promega) overnight at 16°C. *E. coli* was transformed with the ligated vectors on ice during 30 minutes, followed by thermal shock at 42°C one minute and 2 minutes on ice. After 45 minutes at 37°C with vortex, bacteria culture was centrifuged 4 minutes at 4000 RPM and the pellet was plated in 10 cm LB plus ampicillin petri dishes (supplemented with X-Gal and IPTG) and incubated overnight at 37ºC. Positive white colonies were grown in 1.5 mL of LB medium with ampicillin incubated overnight at 37°C. A minimum of eight single clones were extracted using the Nucleospin® 96 Plasmid kit (Macherey-Nagel) and the products were submitted to sequencing PCR using BigDye Terminator V3.1 cycle sequencing kit (Applied Biosystems) and sequenced in a Hitachi 3730 DNA sequencer (Applied Biosystems). Results were analyzed using BioEdit software and methylated cytosines were mapped using BSMap software. NSUN5 CpG methylation status in pediatric gliomas was determined by pyrosequencing using

primer sequences shown in the Key Resources Table**.** Bisulfite converted DNA (200 ng) was used for hybridization on the HumanMethylation450 or Infinium MethylationEPIC BeadChip (Illumina). Raw fluorescence intensity values were normalized with Illumina Genome Studio software (V2011.1) using 'control normalization' with background correction. Normalized intensities were then used to calculate DNA methylation levels (beta values).

Expression Analyses

For real time quantitative reverse transcription PCR experiments, total RNA extraction was performed on three independent cellular pellets from each cell line, or one sample of brain white matter, using the SimplyRNA kit (Promega) on a Maxwell RSC device (Promega). The extracted RNA was stored at -80°C. Three µg of DNase treated RNA were converted to cDNA using the ThermoScript RT-PCR system (ThermoFisher) with oligo(dT) primers. Quantitative PCR was performed using SYBR® Green PCR Master Mix (ThermoFisher) on a QuantStudio5 (AppliedBiosystems) with QuantStudio™ Design&Analysis Software v1.3.1. Primers for qPCR are listed in the Key Resources Table. Reactivation treatments with the demethylating agent 5-aza-2′-deoxycytidine (AZA; Sigma) were performed at 0.5 µM for 72 h, beginning with confluence at 60% approximately. RNA extraction and RT-PCR were carried out as described before. For immunoblotting assays, we extracted cell pellets and brain white matter samples with 300 µL of RIPA buffer (50 mM Tris pH 7.5, 150 mM NaCl, 1 mM EDTA and EGTA, 1% NP40, 0.5% of sodium deoxycholate, 0.1% of SDS) containing protease and phosphatase inhibitors cocktail cOmplete™ (Roche) for 30 min on ice, followed by centrifugation at maximum speed for 20 min at 4ºC. Protein levels were quantified using Pierce™ BCA protein assay kit (Thermo Scientific) following manufacturer's recommendations. 80 µg of total cellular proteins were loaded per lane of SDS-polyacrylamide gels. Proteins were transferred to nitrocellulose membranes, then membranes were blocked in 5% non-fat dry milk in Tris-buffered saline supplemented with Tween20 (TBS-T, Biorad) and incubated with primary antibodies diluted in milk for 60 min at RT or overnight at 4ºC. Membranes were incubated with horseradish peroxidase-conjugated secondary antibodies for 1 hour before developing with enhanced chemiluminescence (ECL, Biorad). Antibodies used in this study are described in the Key Resources Table.

NSUN5 transfection

The cDNA sequence of NSUN5 containing a FLAG-Tag in the amino-terminal end was cloned into the pLVX-IRES-ZsGreen1 expression plasmid (Clontech Laboratories) between the EcoRI and XbaI restriction sites. Lentiviruses containing this construct were produced by cotransfecting HEK-293T cells with the recombinant pLVX-IRES-ZsGreen1, psPAX2 (Addgene) and pMD2.G (Addgene), using jetPRIME® Transfection Reagent (Polyplus Transfection) and following the supplier instructions. The transfection cocktail was removed after 6h and replaced by fresh medium. After 72h, viral containing supernatant was collected, 0.45 µM-filtered and stored at 4 ºC before infection. The recombinant product was randomly inserted by lentiviral transduction into the genome of A172 and LN229 glioma cell lines. After 5 passages, the green fluorescent cells were sorted by FACS and cultured in DMEM medium supplemented with 10% FBS. For primers sequences please refer to the Key Resources Table.

NSUN5 mutated transfection

The cDNA of NSUN5 from the DBTRG-05MG cell line was used to incorporate 4 specific point mutations at the 3 SAM-binding sites and the active site. The point mutations were D258L, R263L, D305L and C359V, and for every substituted amino acid the most frequent nucleotide codon was chosen. We used PCR fusion method with 3 different pairs of primers, the first ones including mutations at positions 258 and 263 (for sequences please refer to the Key Resources Table). The mutated NSUN5 was cloned into the pLVX-IRES-ZsGreen1 with EcoRI and SpeI, tagged with FLAG. Another mutated construct was cloned into pLVX-IRES-ZsGreen1 changing GFP by TOMATO. The corresponding tdTomato NSUN5 wild type was done and was tagged with HA.

NSUN5 depletion

Four different gene-specific short hairpin RNA molecules (shRNAs) for NSUN5 mRNA were designed and transduced into NSUN5 expressing glioma cell lines (for sequences please refer to the Key Resources Table). shRNA against the MSS2 yeast mRNA (not present in mammals) was used as scramble (control). All shRNA molecules were ligated into pLVX-shRNA2-ZsGreen plasmid (Clontech). 10ug of each encoding plasmid was mixed with 7.5µg of ps-PAX2 and 2.5µg of PMD2.G plasmid (Addgene), using jetPRIME® Transfection Reagent (Polyplus Transfection). Upon 10 min of RT incubation, the transfection mix was added dropwise on a 10 cm culture plate containing HEK293-TLV lentiviral packaging cells at 80% confluence. After 72h, medium with high-titer lentiviral particles was 0.45µm filtered. NSUN5 expressing glioma cell lines were cultured in virus containing medium for 24 h. After 5 passages, the green fluorescent cells were sorted by FACS and cultured in DMEM medium supplemented with 10% FBS.

Brain tumor xenografts

IVIS images: All mouse experiments were approved and performed in accordance with the guidelines of the Institutional Animal Care Committee of the Vall d'Hebron Research Institute. 1 x 10^6 cells were stereotactically inoculated into the corpus striatum of the right brain hemispher of 9-week-old athymic mice Nude-Foxn1nu mice (Charles River Laboratories). In order to estimate the size of tumors the luciferase activity of inoculated tumor cells was quantified in a Xenogen-CCD camera from IVIS. All mouse experiments were approved by and performed according to the guidelines of the Institutional Animal Care Committee of the Vall d'Hebron Research Institute in agreement with the European Union and national directives. 3x10^5 A172 and LN229 (EV or NSUN5) cells were stereotactically inoculated into the corpus striatum of the right brain hemisphere (1mm anterior and 1.8mm lateral to the lambda; 2.5 mm intraparenchymal) of 8-week-old Balb/c nude mice (Janvier Labs). We inoculated 7 animals per group. Tumor progression was monitored by bioluminescence measurements using the Xenogen IVIS® Spectrum. Mice were euthanized at day 17 after inoculation.
Xenografts growth: All mouse experiments were approved and performed in accordance with the guidelines of the Institutional Animal Care Committee of the Bellvitge Biomedical Research Institute. For tumor growth into the brain, 3 x10^5

or 1,5 x10^6 cells, respectively for LN229 (EV or NSUN5) and DBTRG-05MG (Scramble or ShNSUN5) were inoculated into the corpus striatum of 9 (Scramble and ShNSUN5) or 10 (EV and NSUN5) mice for each cell line individually.

Subcutaneous tumor xenografts

Five-week-old male athymic nu/nu mice (Charles River, Wilmington, MA, USA), housed under specific pathogen-free conditions, were used in this study. In order to assess tumor growth, 2x10^6 LN229-EV, LN229-NSUN5 or 4x10^6 DBTRG-05MG-Scramble, DBTRG-05MG-ShNSUN5 glioma cells were subcutaneously injected in the flank of 8 animals for LN229 and 8 others for DBTRG-05MG. Each mouse had in one flank a cell line and in the opposite flank the respective construct. Tumors were measured in two dimensions using calipers, and volume was calculated using the formula: V (mm^3) = (L × W2)/2. Mice were sacrificed 28 days after injection, and tumors were then excised and weighed.

NSUN5 Immunoprecipitation and RT-qPCR.

Immunoprecipitations were carried out from total LN229 extracts (transfected with empty vector or NSUN5) overnight with 10 µl of Anti-Flag M2 Magnetic Beads (Sigma) in RIP buffer (150mM KCl, 25mM Tris pH7.4, 5mM EDTA, 0.5mM DTT, 0.5% NP40, protease inhibitors). Beads were then washed three times with RIP buffer, and 10% of beads were boiled in Laemmli buffer 1x and loaded on a protein gel to check immunoprecipitation efficiency. The pulled-down RNA from the remaining 90% of beads was extracted by adding 1 mL of TRIzol® Reagent (ThermoFisher). After phenol extraction and isopropanol precipitation, a DNase treatment step was performed, and the final pellet was resuspended in water. Equal amounts of each sample were then retrotranscribed and analyzed by quantitative PCR (Applied Biosystems 7900HT Fast Real Time PCR System). The equivalent amount of input RNA was processed in parallel to estimate pull-down efficiency.

RNA methylation analyses

Total RNA or cytoplasmic and nuclear RNA were extracted by SimplyRNA kit (Promega) on a Maxwell RSC device (Promega) or using PARIS™ kit (Ambion® PARIS™ system), respectively.

Amplicon-specific detection of cytosine modifications: Bisulfite conversion of cellular RNA was performed with the Methylamp RNA Bisulfite Conversion Kit (EpiGentek) following the manufacturer's instructions. Ten µL of the eluted bsRNA were converted into cDNA using the ThermoScript RT-PCR system (ThermoFisher) with random hexamer primers prior to PCR amplification. PCR primers for the sequences of interest are available in the Key Resources Table. The amplicon PCR band was purified, ligated into pGEM®-T Easy vector (Promega), transformed into *E. coli* and individual clones sequenced as described in **DNA methylation analyses**. Clones with uncompleted conversion (cytosines other than C3782 unconverted into thymines) were discarded.

Transcriptome-wide bisulfite RNA sequencing (bsRNA-seq): Three independent biological replicate samples of cellular RNA from NSUN5-transfected LN229 glioma cells and empty vector-transfected cells were prepared. RNA was isolated using the Maxwell RNA purification Kit, then DNase treated using Turbo DNase

(Ambion) and phenol/chloroform extracted using phase-lock tubes and ethanol precipitated. RNA quality was evaluated on Bioanalyzer Nano Chips (Agilent). 10ug of each RNA sample was spiked with 10ng each of two Luciferace *in vitro* transcripts to assess bisulfite conversion efficiency across the replicates. rRNA content of the samples was reduced using the Ribo-Zero rRNA Removal Kit (Illumina) prior to bisulfite conversion as previously described (Squires et al., 2012). Samples were ethanol-precipitated, re-suspended in water and the efficiency of rRNA depletion checked using the Bioanalyzer. Bisulfite conversion was carried out as described previously (Sibbritt et al., 2016) and the fragmentation of the RNA analyzed using the Bioanalyzer. Samples were supplemented with an equal volume of “Elute, Prime, Fragment High Mix” (Illumina) and incubated at 70°C for 3 minutes. Libraries were then prepared according to Illumina TruSeq Stranded Total RNA protocol and sequenced on an Illumina Hiseq2500 instrument in 2x100bp paired end mode using the Illumina HiSeq Rapid SBS kit v2. The fastq sequence files were quality checked and preprocessed with FastQC and BBDUK software (https://sourceforge.net /projects/bbmap) in order to remove the adapter sequences and low quality reads. The preprocessed reads were mapped with Hisat2-2.0.4 software (https://ccb.jhu.edu /software/hisat2/index.shtml) against the human converted transcriptome corresponding to Hg19 build. Only uniquely mapped reads were considered for the general analysis. For a specific rRNA analysis we mapped the reads against a sequence subset including RefSeq rRNA sequences corresponding to 28S, 18S, 5S and 5.8S. We used the BS-RNA software (http://bs-rna.big.ac.cn/) in order to obtain the m5C methylation value for each sample from the mapped reads. We obtained the methylation data for all cytosines per sample in both strands from which we selected only cytosines with data for all the samples. Furthermore, we filtered the data without reads present in half the samples. Finally, we normalize the read counts and obtained the cytosines differentially methylated between empty vector and NSUN5 transfected conditions by using the R package limma (Parker et al., 2017). We selected the cytosines with a multiple correction adjusted pvalue < 0.05, a logFC > 0 and a methylation difference over 0.33.

Reverse Phase-High Pressure Liquid Chromatography (RP-HPLC) and mung bean nuclease protection assay

Extraction of total RNA from the glioma cell lines was performed using phenol/chloroform to obtain 1mg. RP-HPLC analysis as well as the mung bean nuclease protection assay were performed as described previously (Yang et al., 2016). Synthetic deoxyoligonucleotides complementary to the specific sequence of C3765-3812 of human 28S rRNA were used for protection by hybridization to the rRNA. After digestion by mung bean nuclease (MBN Kit: M0250S, NEB) and 0.05 mg/ml RNase A (Sigma-Aldrich) purification of protected fragments from 8 M urea-PAGE (13%) was carried out by passive elution with 0.3 M NaAc by rotation at 4 °C overnight. Precipitation of eluted rRNA fragments was done using 100% EtOH. Isolated fragments were digested with nuclease P1 and bacterial alkaline phosphatase (Sigma Aldrich) and subsequently the nucleosides were analyzed by RP-HPLC on a Supelcosil LC-18-S HPLC column (25 cm × 4.6 cm × 5 μm) equipped with a precolumn (4.6 cm × 20 mm) at 30 °C on an Agilent 1200 HPLC system.

Molecular dynamics simulations

The Human 80S ribosome was taken from the high-resolution (2.9 Å) cryoEM structure with PDB id 6EK0 (Natchiar et al., 2017). Since the modelling of the complete 80S ribosome with all the modified bases reported (Natchiar et al., 2017) is not accessible with the current state-of-the-art methods available in the field (Šponer et al., 2018, Dans et al., 2018), we focused on a region of the ribosome 30 Å around 5mC3782, which comprise part of the 28S subunit (model labelled r30A5mC). The region contained 263 RNA residues, 21 amino acids belonging to the α-helix of the 60S ribosomal protein L41, and 13 Mg2+ ions. Several RNA chains of the 28S subunit were cut by our selection, consequently the Cartesian coordinates of all atoms in the mentioned region were fixed in the space as found experimentally by means of a harmonic potential of 5 KcalÅ2. The RNA residues 3750-3793 and 3807-3816 from the 28S subunit, the residues 15 to 24 from protein L41, and 8 Mg2+ (labelled 4405, 5186, 5188, 5215, 5252, 5299, 5632, 5639 in 6EK0) were completely free to move during the Molecular Dynamics (MD) simulation. The r30A5mC model also contained 10 different types of modified bases. PSU, 5MC, and 7MG were treated with the available parameters (Aduri et al., 2007), while OMC was converted to C, B8H to 5MU, A2M to M2A, OMG to G, B8W to G, UR3 to 3MU, and MA6 to 6MA. The model was then minimized in vacuo, neutralized (with 164 Na+ ions), solvated (with explicit waters and 0.4 M of added Na+Cl-), and minimized in solution with positional restraints on the solute using our well established multi-step protocol (Pérez et al., 2007, Dans et al., 2016). To produce the final models, the minimized structures were thermalized to 298ºC at NVT, and then simulated during 60 ns by means of Molecular Dynamics simulations at NPT (P = 1 atm). The first 10 ns of the simulations were considered as an equilibration step and were discarded for further analysis. The system labelled r30AC was built from r30A5mC by replacing 5mC3782 by C3782 (i.e. removing the methyl group).

To treat the L41 protein we used the ff12SB force field (Hornak et al., 2006), which is compatible with the modified RNA bases developed by Santa Lucia and co-workers (Aduri et al., 2007). The RNA was represented using parmbsc0 (Pérez et al., 2008) with OL3 correction (Zgarbova et al., 2011), and the entire system was surrounded by a truncated octahedral box of ~20,000 TIP3P water molecules (Jorgensen et al., 1983), applying Dang's parameters on ions (Smith et al., 1994). Ions were initially placed randomly, at a minimum distance of 5 Å from the solute and 3.5 Å from one another. All systems were simulated using the Berendsen algorithm (Berendsen et al., 1984) to control the temperature and the pressure, with a coupling constant of 5 ps. Center-of-mass motion was removed every 10 ps to limit build-up of the translational kinetic energy of the solute. SHAKE (Ryckaert et al., 1977) was used to keep all bonds involving hydrogen at their equilibrium values, which allowed us to use a 2 fs step for the integration of Newton equations of motion. Long-range electrostatic interactions were accounted for by using the Particle Mesh Ewald method (Darden et al., 1993) with standard defaults, and a real-space cut-off of 10 Å. All simulations were carried out using the PMEMD CUDA code module (Salomon-Ferrer et al., 2013) of AMBER 18 (Case et al., 2018), and analysed with CPPTRAJ (Roe et al., 2013), VMD 1.9.3 (Humphrey et al., 1996), and PYMOL 2.2.3 (Schrodinger et al., 2018).

Global determination of protein synthesis

Overall protein synthesis in the different experimental conditions was assessed by the incorporation of O-propargyl-puromycin (OP-Puro) into nascent proteins (Blanco et al., 2006) using the Click-iT® OPP Reagent (Thermo Fisher) and [3H] leucine incorporation (Kirchman et al., 1985). Cells were labeled for nascent protein synthesis using the Click-iT® OPP Reagent (Thermo Fisher, catalogue no.C10458) according to manufacturer's instructions. Briefly, following a hydrogen peroxide treatment, cells were incubated with 18 µM OPP Reagent in cell medium for 1 h. Cells were then fixed with 3.7% paraformaldehyde in PBS for 15 min, and permeabilized in PBS supplemented with 1% fetal bovine serum (Invitrogen) and 0.1% saponin (Sigma-Aldrich) for 5 min at room temperature. To conjugate OP-puro to a fluorochrome, an azide-alkyne cycloaddition was performed using the Click-iT® Plus OPP Protein Synthesis Kit (Thermo Fisher Scientific) with Alexa Fluor-647. After the 30 min reaction, cells were washed twice in PBS, and further stained for DAPI. Slides were mounted for imaging in glycerol supplemented with Mowiol 4-88 (Calbiochem catalogue no. 475904). Fluorescence images were acquired using a confocal microscope (Leica TCS SP5) at 1,024 × 1,024 dpi resolution. All the images were further processed with Fiji (ImageJ) software. To quantify protein synthesis rates, the fluorescence intensity of the OP-Puro signal was thresholded (mean OP-Puro = Integrated density/area) and divided by the total number of cells per image. The mean of OP-Puro incorporation was averaged from several images (n=10) per condition. For 3H-leucine incorporation, cells at 60-70% confluence were incubated with 10 mCi/mL 3H-leucine (Perkin Elmer, catalog no. NET116600) for 30 min. Cells were washed with phosphate-buffered saline (PBS), and lysed in RIPA buffer (50 mM Tris-HCl pH 8, 150 mM NaCl, 1% Triton X-100, 0.5% sodium deoxycholate, 0.1% SDS, 1 mM dithiothreitol (DTT)). The lysates were cleared by centrifugation and incubated with cold 10% trichloroacetic acid (TCA) for 10 min. TCA-insoluble proteins were then washed twice with 5% TCA, solubilised with 0.1 M NaOH, and analyzed using a liquid scintillation counter (Tri-Carb 2100TR, Perkin Elmer). Results were normalized to protein concentration quantified using Pierce™ BCA protein assay kit (Thermo Scientific).

To induce oxidative stress, 100µM H_2O_2 was added to the cell culture medium 16h before OP-Puro procedure. Nutrient deprivation medium consisted in DMEM medium with low Glucose, and no L-glutamine, Phenol Red and HEPES (ThermoFisher catalogue no. 11054020), supplemented with 10% dialyzed FBS, 1X GlutaMAX (ThermoFisher catalogue no.35050061) and 1mM Sodium Pyruvate (ThermoFisher catalogue no.11360070). Cells were washed three times with PBS prior to the addition of the nutrient deprivation medium and were cultured for 16h before OP-Puro procedure.

Assessment of Translational Efficiency, RNA-sequencing (RNA-seq) and Ribosomal profiling (Ribo-seq).

Translational efficiency for each RNA was determined by normalizing the ribosome profiling deep sequencing data (Ribo-seq) to transcript length and total transcript abundance according to deep sequencing RNA transcriptome (RNA-seq) data, as previously described (Wolfe et al., 2014). For RNA-seq, 2-3 replicates of RNA of each cell line A172 EV, A172 NSUN5-transfected, LN229 EV, LN229 NSUN5-transfected with and without stress stimulus, and DBTRG-05MG Scramble, shNSUN5 depleted without stress stimulus were sent to CNAG-CRG (Barcelona). RNA quality was controlled by QUBIT RNA BR Assay kit (Thermo Fisher). For both RNA-Seq and Ribo-Seq, libraries were prepared with

TruSeq stranded mRNA kit (Illumina) and sequenced in paired-end mode with a read length of 2x100bp on a HiSeq2000 (Illumina) following the manufacturer's protocol. An average of 70 to 93 million pairs of 100-bp paired-end read per sample was generated. After adapter trimming and quality score calculation, an average of 64 to 87 million reads were left on each sample. Reads showed a minimum quality of Q30 in at least 95% of base calls. Ribo-seq was performed following a modified version of the original protocol (Ingolia et al., 2012). A minimum of five replicates was performed for each sample (empty vector versus NSUN5 cells; Scramble versus shNSUN5 cells). Briefly, cells were grown until confluence, and then washed twice with PBS. An in-dish lysis was performed [20 mM Tris-Cl (pH 7.4), 150 mM NaCl, 5 mM MgCl2, 1 mM DTT, 100 µg/ml cycloheximide, 1% Triton X-100, 25 U/ml of Turbo DNase I (Thermo Fisher Scientific)]. Lysates were further triturated by passing them ten times through a 26-G needle and centrifuged at 20,000 g. for 10 min to remove nuclei and debris. Supernatant was digested with RNaseI (100 U/µl, Thermo Fisher Scientific) for 45 min at room temperature. Digestion was blocked with SUPERase*In (Invitrogen) and lysates were layered on a 1M sucrose cushion and separated by ultracentrifugation at 80 000 rpm in a TLA110 rotor for 204 min at 4ºC. Pellets were resuspended in Qiazol, and RNA was purified using the miRNeasy kit (Qiagen) according to manufacturer's instructions. Size selection of footprints with a length of 26–34 nucleotides was performed on 15% TBE-urea gels (Invitrogen). Footprints were 3′-dephosphorylated with a T4 polynucleotide kinase (New England Biolabs). 3′-adenylated and 5′-phosphorylated adapters suitable for Illumina RNA sequencing were ligated to the RNA fragments. RNA was reverse transcribed at 50°C for 1 h (SuperScript III cDNA synthesis kit, Invitrogen), followed by rDNA depletion by subtractive hybridization using oligonucleotides (as stated in the original protocol). Complementary DNAs were PCR amplified using a different primer index per sample. The libraries were sent to Macrogen (Seoul, Rep. of Korea).

For alignment and quantification of RNA-seq and Ribo-seq, read quality and adapter sequence presence was checked with FastQC (version 0.11.4) software (http://www.bioinformatics.babraham.ac.uk/projects/fastqc). Sequencing adapters were removed from the 5' and 3' ends of the reads using BBDUK (http://jgi.doe.gov/data-and-tools/bbtools/bb-tools-user-guide/bbduk-guide/).
RNA-Seq reads were aligned to the human reference transcriptome (GRCh37 build) using HISAT2 v.2.0.5 (Pertea et al. 2016). The alignments were then assembled with Stringtie v.1.3.3b (Pertea et al., 2015). The average of mapped reads per sample was 98%. RNA (RNA-seq) and ribosome-protected RNA (Ribo-seq) count tables were calculated from the StringTie output, with PrepDe.py. Differential gene expression at transcript level was analyzed using the R programming language (version 3.4) and DESeq2 package (version 1.16.1, Love et al., 2014). In the case of RNA seq data, DESeq2 default options were used. For Ribo-seq analyses, default options were turned off and an independent filtering was applied by using the Riborex R package as well as a minimum count transcript variance greater than 0 or 1, depending on the dataset quality. The cut-off for considering a transcript significantly up-expressed or down-expressed in the RNA-Seq samples was padj < 0.01 and an absolute log2-FoldChange>= 2. The latter values were calculated by collapsing data into gene symbols. For Ribo-seq data the cut off was pvalue <= 0.01 and a log2-FoldChange>= 0.5. Translational changes in genes were defined as differentially expressed, based on the ribosome profiling data. To analyze Ribo-seq ratios with expression and

identify differential translation the R package RiboDiff was used. This package compares the relative differences in the ratio of RIBO-seq mRNA fractions to total mRNA expression levels, translation efficiency was calculated (TE) (absolute fold change in TE > 0.5; pvalue<0.01). The final TE gene candidates were selected from the intersection of transcripts without RNA abundance differences, transcripts with significant less ribosome occupancy in LN229 NSUN5 cells and proteins significantly down regulated in NSUN5 transfected cells by SILAC experiments. For DBTRG-05MG cells, the final TE gene candidates were selected from the intersection of transcripts without RNA abundance differences and transcripts with significant less ribosome occupancy in DBTRG-05MG Scramble cells.

Gene functional enrichment analysis and sequence motifs recognition

Gene sets were used to perform a gene set overrepresentation analysis over the GO biological processes and KEGG pathways included in the public GSEA signature database collections and the DAVID web service (https://david.ncifcrf.gov/). The top gene clusters resulting from the hypergeometric test with a FDR adjusted p-value < 0.05 were finally considered. 5'UTR DNA sequences from selected genes were used to perform a motif recognition analysis. RegRNA 2.0 (http://regrna2.mbc.nctu.edu.tw/) and FIMO software from MEME suite 4.12.0 (http://meme-suite.org/index.html) were used to perform CERT, TOP, PRTE and uORF motif scanning. IRESPred web service (http://196.1.114.46:1800/IRESPred/home.html) and Infernal software (http://eddylab.org/infernal/) were used to identify IRES motifs. G4-quadruplexes were identified by using QGRS Mapper software (http://bioinformatics. ramapo.edu/QGRS/index.php).

Stable isotope labeling by amino acids in cell culture (SILAC)

SILAC was performed as previously described (Lopez-Serra et al., 2014). Samples were searched against a SwissProt database containing entries corresponding to Human (version of April 2016) a list of common contaminants and all the corresponding decoy entries. Resulting data files were filtered for FDR <1%. In more detail, SILAC labeling was performed by using SILAC-Lys8- Arg10-Kit media (Silantes). LN229 EV and LN229 NSUN5 transfected cells were grown during 10 days in heavy medium (Lys 8- Arg 10) or light medium (Lys 0 – Arg 0), respectively. After labeling, protein extraction was carried out by using RIPA buffer. Protein levels were quantified by Lowry and mixed in 1:1 ratio. Samples were further processed by the FASP method followed by alkylation with iodoacetamide and o/n digestion by Lys-c and trypsin over day at 37°C in 50 mM ammonium bicarbonate. Peptide mixes were analyzed using an OrbitrapFusion Lumos mass spectrometer (Thermo Scientific, San Jose, CA, USA) coupled to an EasyLC (Thermo Scientific (Proxeon), Odense, Denmark). All data were acquired with Xcalibur software v3.0.63. Proteome Discoverer software suite (v2.0, Thermo Fisher Scientific) and the Mascot search engine (v2.5, Matrix Science (1)) were used for peptide identification and quantification. Samples were searched against a SwissProt database containing entries corresponding to Human (version of April 2016) a list of common contaminants and all the corresponding decoy entries. Resulting data files were filtered for FDR <1%. Only peptide intensities corresponding to master protein category were used. The

ratios from average peptide intensities were calculated and normalized for each protein. Finally, the ratio differences between EV and NSUN5-transfected groups were calculated by applying a Student's t-test. Significant up and down-regulated proteins (p-value < 0.05) were selected.

Deoxynyboquinone and isobutyl-Deoxynyboquinone drug assay

IC50 studies in the glioma cell lines upon drug exposure were performed using the sulforhodamineB (SRB) assay. Briefly, 48h after exposure to nine increasing concentrations of deoxynyboquinone (2nM to 15µM) or isobutyl-Deoxynyboquinone (0.7nM to 5µM), culture medium was removed and 100µL of 10% trichloroacetic acid was added to the wells in order to fix cells for one hour at 4ºC. Cells were then washed twice with distilled water and stained with 100µL of 0.4% SRB in 1% acetic acid during 30 minutes, light-protected, and washed twice with 1% acetic acid. SRB was then solubilized in Tris base (10mM; pH 10.0) and 540 nm-optical densities were determined using a microplate reader (Perkin Elmer Viktor 3). Data were analyzed with GraphPad Prism 5 software (GraphPad Software, La Jolla California USA).
For in vivo treatment with isobutyl-Deoxynyboquinone (IB-DNQ), 12 mice were inoculated with $2x10^6$ A172 or DBTRG-05MG cells into the brain, and we randomly chose 7 of each group to be treated. Five doses were orally given each two days consisting of 12mg/g of the drug dissolved in 2-hydroxypropyl-beta-cyclodextrin (HP□CD). Mock groups of 5 mice received in the same conditions the HP□CD without drug.

Electronic microscopy

For transmission electron microscopy, LN229 cells at 80% of confluence were fixed in 2.5% glutaraldehyde in 0.1 M phosphate buffer, post fixed in 1.5% osmium tetroxide, and processed using the tEPON 812 embedding Kit (Tousimis®). The ultrathin sections, stained with uranyl acetate and lead citrate, were assessed at 80 kv with a JEM-1011 (JEOL) transmission electron microscope. Ribosomes were count in 10 random regions of 8 different pictures by three different researchers in blind samples and normalized by the area.

Autoradiographic Analysis of rRNA Synthesis

To label newly synthesized RNA, cells were incubated for 2 hrs with 1.2 µCi/ml of [^{3}H]-uridine (Perkin Elmer) before RNA extraction with TRIzol reagent (Invitrogen). 2µg of total RNA were resolved on a formaldehyde-containing 1.2% agarose gel and transferred to Hybond N+ membrane (GE Healthcare). After ultraviolet crosslinking, the membranes were sprayed with EN3HANCE (Perkin Elmer) and exposed to Kodak BioMax MS film (Kodak) at -80ºC for 1 week.

Patients

DNA methylation data in in the discovery set of glioma cases was collected from the The Cancer Genome Atlas (TCGA) Data Portal (https://tcga-data.nci.nih.gov/tcga/). For the initial glioma validation cohort, 115 formalin-fixed paraffin-embedded (FFPE) tumor tissues from glioma patients were retrospectively collected in four different centers (Hospital Universitari de Bellvitge, Hospital Germans Trias i Pujol, Hospital Clínic de Barcelona and

University Hospital Basel) from 1989 to 2018 and were histologically reviewed. Most of patients had received treatment based in temozolamide combined or not with radiotherapy, and molecular analyses of IDH1 mutational status and MGMT methylation were available. Co-deletion 1p19q status was only available in 23 of the 115 cases. Patients gave their informed consent to participate in the research, which had received ethical approval from the review board of each institution. The expanded glioma validation cohort included 303 additional glioma patients from which NSUN5 methylation, IDH1 mutation, co-deletion of 1p19q, MGMT methylation and Progression Free Survival are available (Bady et al. 2018; Kickingereder et al., 2018). These cases had signed written informed consent and were histologically reviewed as described in the respective publications (Bady et al. 2018; Kickingereder et al., 2018). In TCGA cohort, presence of G-CIMP phenotype was also available. We determined the sample G-CIMP status as previously described (Noushmehr et al., 2010). Briefly we analyzed the epigenetic signature of 1 hypomethylated locus, DOCK5, and 7 hypermethylated loci (ANKRD43, FAS-1, FAS-2, HFE, LGALS3, MAL, and RHO-F). We considered a sample as G-CIMP positive if at least 6 genes displayed a combination of DOCK5 DNA hypomethylation and/or hypermethylation of the remaining genes.

Statistical analysis

The associations between variables were assessed by $\chi 2$ tests, Fisher's exact test, Welch's t-test, Mann-Whitney test, Wilcoxon paired test or Spearman correlation whenever indicated. Kaplan–Meier plots and log-rank test were used to estimate Progression-Free Survival (PFS) and Overall Survival (OS). Univariate Cox regression analysis was performed in which hazard ratio with a 95% of confidence interval was evaluated. Statistical analysis was performed by using SPSS for Windows (Armonk, NY) and GraphPad Prism 5 (La Jolla, CA) for Windows. P values less than .05 were considered statistically significant. All statistical tests were two-sided. Methylation and expression values for GBM and LGG TCGA primary tumor samples were obtained from the NCI's Genomic Data Commons (GDC) (https://gdc.cancer.gov/). The values corresponding to glioma cell lines were obtained from COSMIC cell line database (http://cancer.sanger.ac.uk/cell_lines). Correlations were obtained by calculating Spearman's rank correlation test and the associated rho coefficient.

Data availability

All the obtained deep sequencing data have been deposited at the SRA (https://www.ncbi.nlm.nih.gov/sra) with the following links:

RNA-Seq: BioProject: PRJNA395552
https://dataview.ncbi.nlm.nih.gov/object/PRJNA395552?reviewer=60ndb08i1g6na5nkoaaiad9a6o

bsRNA-Seq: BioProject: PRJNA395575
https://www.ncbi.nlm.nih.gov/bioproject/395575

Ribo-Seq: BioProject: PRJNA395570
https://www.ncbi.nlm.nih.gov/bioproject/395570

References

Aduri R, Psciuk BT, Saro P, Taniga H, Schlegel, HB, SantaLucia J (2007) AMBER Force Field Parameters for the Naturally Occurring Modified Nucleosides in RNA. J. Chem. Theory Comput. 3, 1464–1475.

Armachea J-P, Jarascha A, Angera AM (2010) Localization of eukaryote-specific ribosomal proteins in a 5.5-Å cryo-EM map of the 80S eukaryotic ribosome. Proc.Nat. Acad. Sc. USA, 107, 19754-19759.

Bady P, Kurscheid S, Delorenzi M, Gorlia T, van den Bent MJ, Hoang-Xuan K et al (2018) The DNA methylome of DDR genes and benefit from RT or TMZ in IDH mutant low-grade glioma treated in EORTC 22033. Acta Neuropathol. 135, 601-15.

Berendsen HJC, Postma JPM, van Gunsteren WF, DiNola A, Haak JR (1984) Molecular dynamics with coupling to an external bath. J. Chem. Phys., 81, 3684.

Blanco S, Bandiera R, Popis M, Hussain S, Lombard P, Aleksic J, Sajini A, Tanna H, Cortés-Garrido R, Gkatza N, et al (2016) Stem cell function and stress response are controlled by protein synthesis. Nature 534, 335-340.

Case DA, Babin V, Berryman JT, Betz RM, Cai Q, Cerutti DS, Cheatham III TE, Darden, TA, Duke RE, Gohlke H, et al (2018) AMBER 18, University of California. San Francisco.

Dans PD, Danilāne L, Ivani I, Dršata T, Lankaš F, Walther J, Illa Pujagut R, Battistini F, Gelpí JL, Lavery R, Orozco M (2016) Long-timescale dynamics of the Drew-Dickerson dodecamer. Nucl. Acids Res., 44, 4052-4066.

Dans PD, Gallego D, Balaceanu A, Darré L, Gómez H, Orozco M (2018) Modeling, Simulations, and Bioinformatics at the Service of RNA Structure. CHEM, doi: 10.1016/j.chempr.2018.09.015.

Darden T, York D, Pedersen L (1993) Particle mesh Ewald: An N·log(N) method for Ewald sums in large systems. J. Chem. Phys., 98, 10089.

Gigova A, Duggimpudi S, Pollex T, Schaefer M, Kos M (2014) A cluster of methylations in the domain IV of 25S rRNA is required for ribosome stability. RNA, 20, 1632–1644.

Hornak V, Abel R, Okur A, Strockbine B, Roitberg A, Simmerling C (2006) Comparison of multiple Amber force fields and development of improved protein backbone parameters. Proteins, 65, 712–725.

Humphrey W, Dalke, A, Schulten K (1996) VMD - Visual Molecular Dynamics, J. Molec. Graphics, 14, 33-38.

Ingolia NT, Brar GA, Rouskin S, McGeachy AM, Weissman JS (2012) The ribosome profiling strategy for monitoring translation in vivo by deep sequencing of ribosome-protected mRNA fragments. Nat. Protoc. 7, 1534-50.

Jorgensen WL, Chandrasekhar J, Madura JD, Impey RW, Klein ML (1983) Comparison of simple potential functions for simulating liquid water. J. Chem. Phys., 79, 926–935.

Kickingereder P, Neuberger , Bonekamp D, Piechotta PL, Götz M, Wick , et al (2018) Radiomic subtyping improves disease stratification beyond key molecular, clinical, and standard imaging characteristics in patients with glioblastoma. Neuro Oncol. 20, 848-57.

Kirchman D, K'nees E, Hodson R (1985) Leucine incorporation and its potential as a measure of protein synthesis by bacteria in natural aquatic systems. Appl. Environ. Microbiol. 49, 599–607.

Lopez-Serra P, Marcilla M, Villanueva A, Ramos-Fernandez A, Palau A, Leal L, Wahi JE, Setien-Baranda F, Szczesna K, Moutinho C, et al (2014) A DERL3-associated defect in the degradation of SLC2A1 mediates the Warburg effect. Nat. Commun. 5, 3608.

Love MI, Huber W, Anders S (2014) Moderated estimation of fold change and dispersion for RNA-seq data with DESeq2. Genome Biol. 15, 550.

Natchiar SK, Myasnikov AG, Kratzat H, Hazemann I, Klaholz BP (2017) Visualization of chemical modifications in the human 80S ribosome structure. Nature 551, 472-477.

Noushmehr H, Weisenberger DJ, Diefes K, Phillips HS, Pujara K, Berman BP, et al (2010) Cancer Genome Atlas Research Network. Identification of a CpG island methylator phenotype that defines a distinct subgroup of glioma. Cancer Cell. 17(5):510–522.

Parker BJ (2017) Statistical Methods for Transcriptome-Wide Analysis of RNA Methylation by Bisulfite Sequencing. In A. Lusser (Ed.), RNA Methylation: Methods and Protocols (pp. 155–167). New York, NY: Springer New York.

Pérez A, Luque FJ, Orozco M (2007) Dynamics of B-DNA on the microsecond time scale. J. Am. Chem. Soc. 129, 14739-14745.

Pérez A, Marchán I, Svozil D, Sponer J, Cheatham III TE, Laughton CA, Orozco M (2008) Refinement of the AMBER force field for nucleic acids: improving the description of α/γ conformers. Biophysical journal 92, 3817-3829.

Pertea M, Kim D, Pertea G, Leek JT, Salzberg SL (2016) Transcript-level expression analysis of RNA-seq experiments with HISAT, StringTie and Ballgown. Nat. Protoc. 11, 1650-1667.

Pertea M, Pertea GM, Antonescu CM, Chang TC, Mendell JT, Salzberg SL (2015) StringTie enables improved reconstruction of a transcriptome from RNA-seq reads. Nat. Biotechnol. 33, 290-295.

Roe DR, Cheatham TE (2013) PTRAJ and CPPTRAJ: Software for Processing and Analysis of Molecular Dynamics Trajectory Data. J. Chem. Theory Comput., 9, 3084–3095.

Ryckaert J-P, Ciccotti G, Berendsen HJ (1977) Numerical integration of the cartesian equations of motion of a system with constraints: molecular dynamics of n-alkanes. J. Comput. Phys., 23, 327–341.

Salomon-Ferrer R, Götz AW, Poole D, Le Grand S, Walker RC (2013) Routine Microsecond Molecular Dynamics Simulations with AMBER on GPUs. 2. Explicit Solvent Particle Mesh Ewald. J. Chem. Theory Comput., 9, 3878–3888.

Schrodinger LLC (2018) The PyMOL Molecular Graphics System.

Sibbritt T, Shafik A, Clark SJ, Preiss T (2016) Nucleotide-Level Profiling of m^5C RNA Methylation. Methods Mol Biol. 1358, 269-284.

Smith DE, Dang LX (1994) Computer simulations of NaCl association in polarizable water. J. Chem. Phys., 100, 3757.

Šponer J, Bussi G, Krepl M, Banáš P, Bottaro S, Cunha RA, Gil-Ley A, Pinamonti G, Poblete S, Jurečka P, Walter NG, Otyepka M (2018) RNA Structural Dynamics As Captured by Molecular Simulations: A Comprehensive Overview. Chem. Rev. 118, 4177–4338.

Squires JE, Patel HR, Nousch M, Sibbritt T, Humphreys DT, Parker BJ, Suter, CM, Preiss T (2012) Widespread occurrence of 5-methylcytosine in human coding and non-coding RNA. Nucleic Acids Res. 40, 5023-5033.

Wolfe AL, Singh K, Zhong Y, Drewe P, Rajasekhar VK, Sanghvi VR, Mavrakis KJ, Jiang M, Roderick JE, Van der Meulen J, et al (2014) RNA G-quadruplexes cause eIF4A-dependent oncogene translation in cancer. Nature 513, 65-70.

Yang J, Sharma S, Watzinger P, Hartmann JD, Kötter P, Entian KD (2016) Mapping of Complete Set of Ribose and Base Modifications of Yeast rRNA by RP-HPLC and Mung Bean Nuclease Assay. Plos One 11, e0168873.

Zgarbova M, Otyepka M, Sponer J, Mladek A, Banas P, Cheatham TE, Jurecka P (2011) Refinement of the Cornell et al. Nucleic Acids Force Field Based on Reference Quantum Chemical Calculations of Glycosidic Torsion Profiles. J. Chem. Theory Comput. 7, 2886-2902.

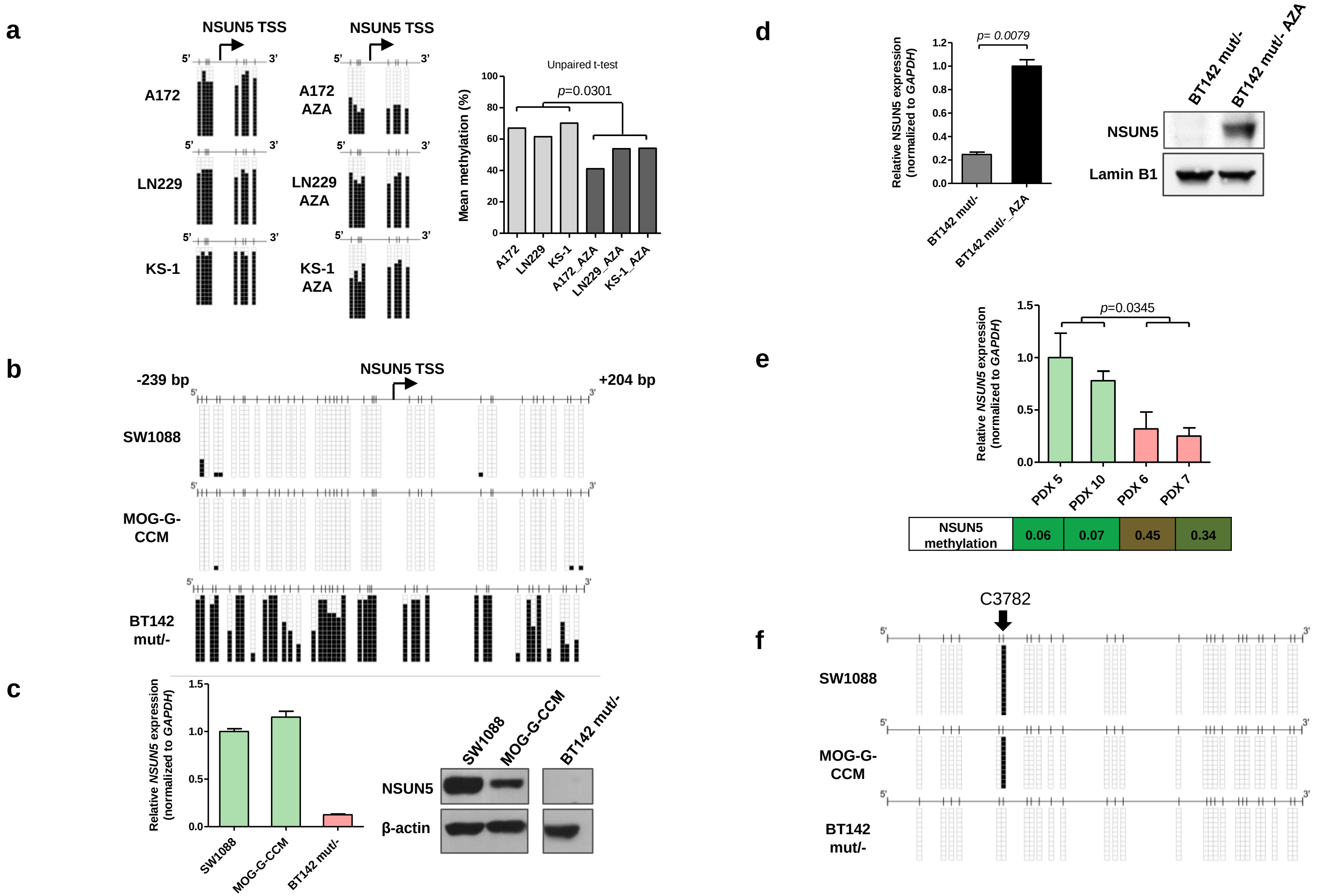

Figure S1. Related to Figure 1. (**a**) Bisulfite genomic sequencing of NSUN5 promoter in the three methylated glioblastoma cell lines in basal condition and after treatment with the demethylating drug 5-aza-2'-deoxycytidine (AZA). CpG dinucleotides around the transcription start site (TSS) are represented as short vertical lines. Single clones are shown for each sample. Presence of an unmethylated or methylated cytosine is indicated by a white or black square, respectively. Graphic represents methylation percentages of all CpGs analyzed in the island. (**b**) Bisulfite genomic sequencing of NSUN5 promoter CpG Island in three grade III glioma cell lines. (**c**) NSUN5 expression in the analyzed grade III glioma cell lines determined by real-time PCR (*left panel*) (data shown represent mean ± s.d. of biological triplicates) and western blot (*right panel*). (**d**) Expression of the NSUN5 RNA transcript and protein was restored in the BT142 mut/- cell line by treatment with AZA, analyzed by real-time PCR (*left panel*) and western blot (*right panel*). (**e**) Association between NSUN5 promoter CpG island hypermethylation determined by pyrosequencing and diminished expression of the NSUN5 transcript determined by qRT-PCR in patient-derived xenografts (PDXs) from primary gliomas. Average CpG methylation value of the NSUN5 pyrosequencing PCR product is shown in each case. Green corresponds to unmethylated samples; red/pink corresponds to methylated samples. (**f**) RNA bisulfite sequencing of the 28S rRNA in the three grade III glioma cell lines. Cytosines are represented as short vertical lines and the C3782 site is displayed by a long black arrow. Presence of an unmethylated or methylated cytosine is indicated by a white or black square, respectively.

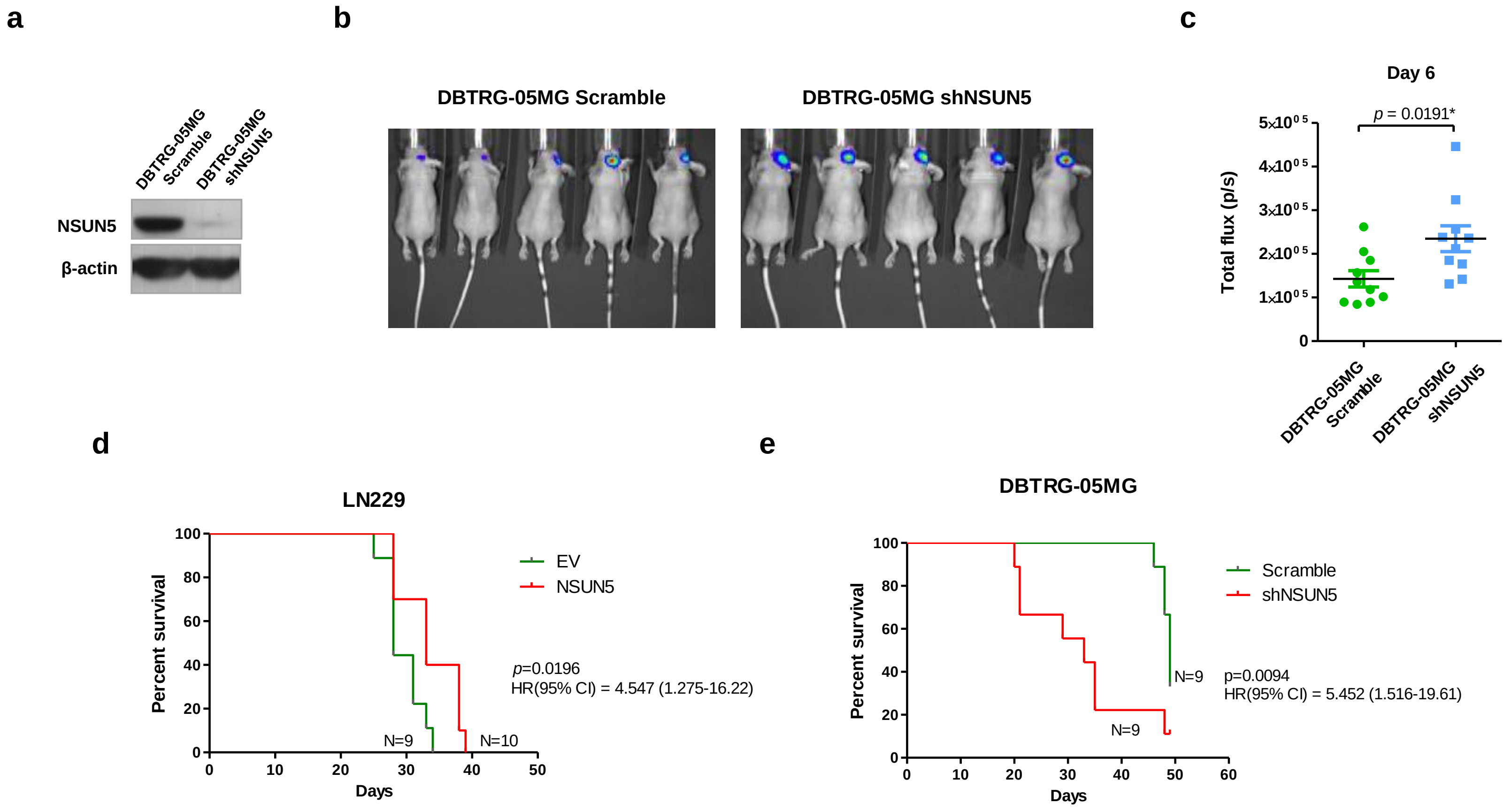

Figure S2. **Related to Figure 2.** (**a**) Western blot to show efficient depletion of NSUN5 protein expression upon stable transfection in DBTRG-05MG glioma cells**.** (**b**) An equal number of the indicated DBTRG-05MG Scramble and shNSUN5 cells populations were stereotactically inoculated into the brain of athymic mice. The size of the tumors was estimated at 6 days post-inoculation (DPI) by the quantification of luciferase activity in the tumor cells. Representative images of the luciferase signal at 6 DPI. (**c**) Scatter plots showing the individual size of the indicated DBTRG-05MG tumors at 6 DPI. (**d**) Kaplan-Meier analysis of Survival in a set of mice models with intracraneal implanted tumors derived from the NSUN5 methylated cell line LN229, empty vector transfected (EV) and NSUN5-transfected (NSUN5) or the NSUN5 unmethylated cell line DBTRG-05MG, scramble and NSUN5-shRNA depleted (**e**). Significance of the log-rank test is shown. Results of the univariate Cox regression analysis are represented by the Hazards Ratio (HR) and 95% Confidence Interval (CI).

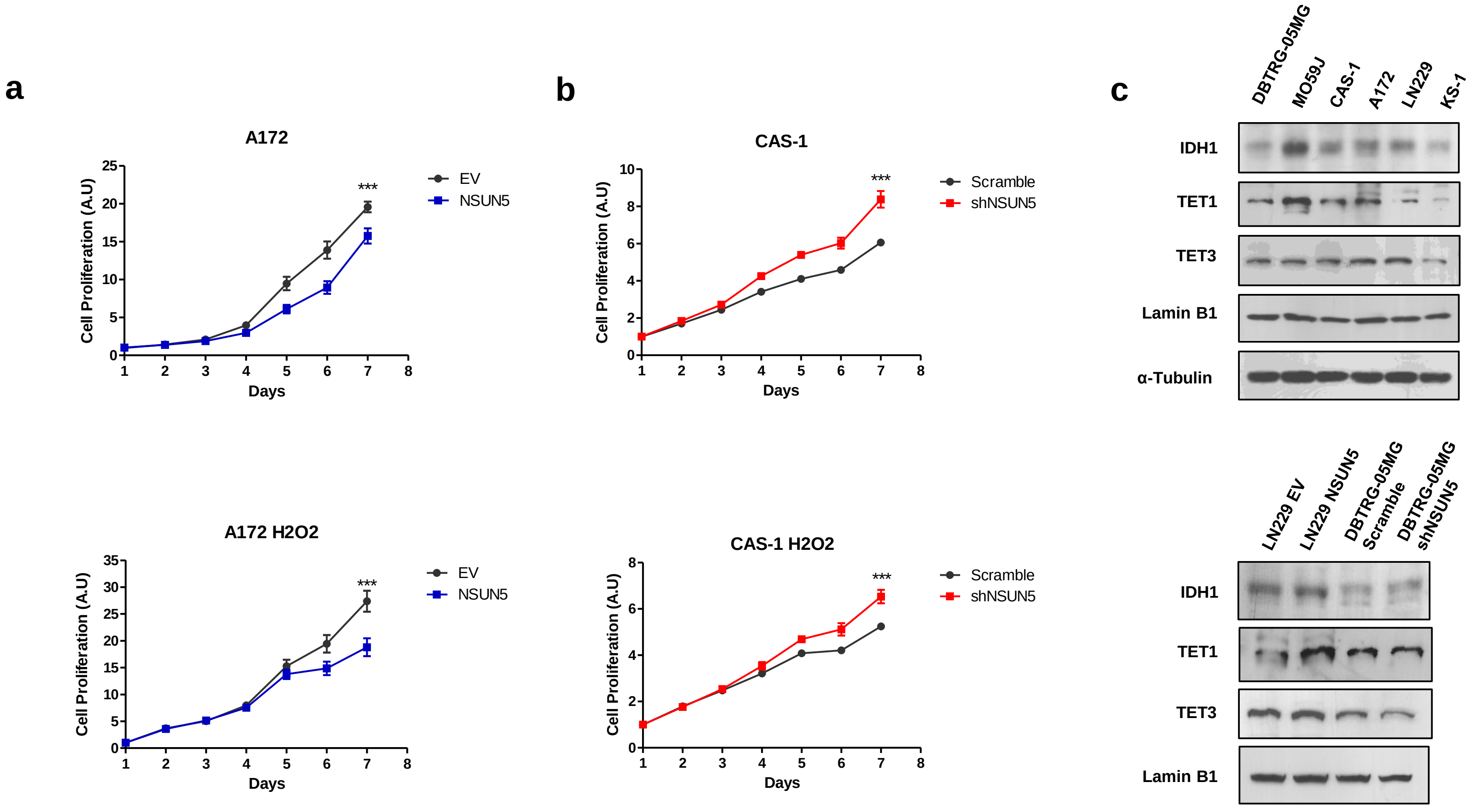

Figure S3. **Related to Figure 2.** (**a**) NSUN5 transfection reduces cell viability in the NSUN5 hypermethylated cell line A172, both in the presence (upper panel) or absence (lower panel) of H2O2 induced stress. (**b**) shRNA-mediated depletion of NSUN5 in the unmethylated glioblastoma cell line CAS-1 increases cell viability, both in the presence (upper panel) or absence (lower panel) of H2O2 induced stress. Values were analyzed from triplicates and expressed as the mean ± SD. Mann–Whitney U test, ***, $P < 0.0001$. (**c**) Expression levels of IDH1, TET1 and TET3 in NSUN5 unmethylated (DBTRG-05MG, MO59J and CAS-1) and methylated (A172, LN229 and KS-1) glioma cell lines (upper panel). Expression levels of IDH1, TET1 and TET3 upon NSUN5 transfection-mediated restoration in LN229 cells or NSUN5 shRNA-mediated depletion in DBTRG-05MG cells (lower panel).

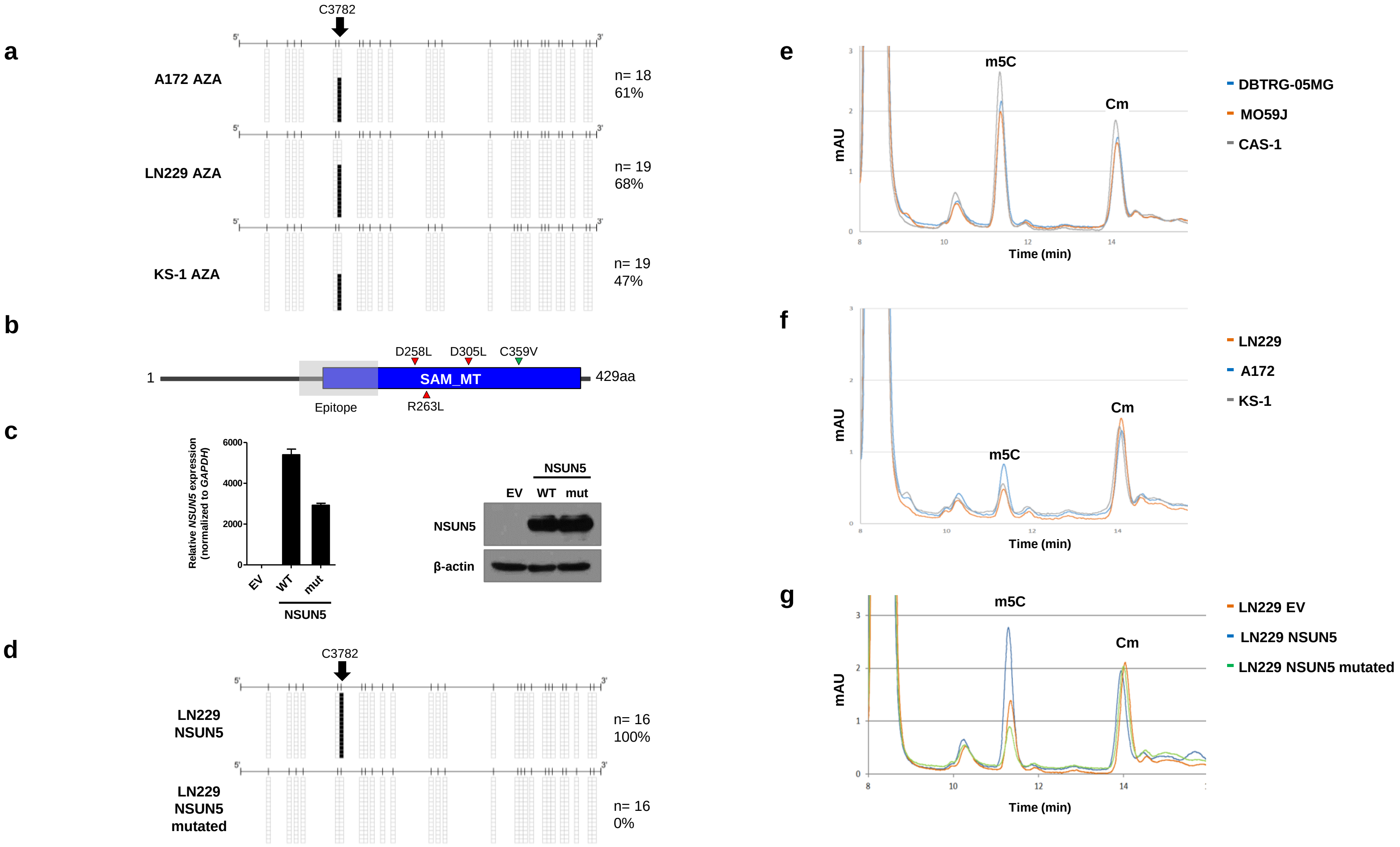

Figure S4. Related to Figure 3. (**a**) RNA bisulfite sequencing of the 28S rRNA in A172, LN229 and KS-1 glioma cells lines after treatment with the demethylating drug AZA. Cytosines are represented as short vertical lines and the C3782 site is displayed by a black arrow. Presence of an unmethylated or methylated cytosine is indicated by a white or black square, respectively. The three glioma cell lines recovered C3782 28S rRNA methylation upon restoration of NSUN5 activity by its promoter demethylation mediated by AZA treatment.(**b**) Schematic representation of the generated mutated NSUN5 protein. Each triangule points out the aminoacid substituted, with red triangules representing SAM-binding sites, and in green, the active site, with the residue directly involved in catalysis. The major functional domain is shown. (SAM_MT: SAM-dependent MTase RsmB/NOP-type domain). (**c**) NSUN5 expression determined by real-time quantitative PCR and western blot, after its tranfection into LN229 glioma cell lines. (**d**) RNA bisulfite sequencing of the 28S rRNA in NSUN5 epigenetically deficient cells transfected with wild-type NSUN5 (LN229-NSUN5-WT) or the engineered mutated NSUN5 protein (LN229-NSUN5-mutated). (**e**) and (**f**) Mung bean nuclease protection assay to determine the methylation status of C3782 at 28S rRNA in the glioma cells lines unmethylated and methylated for the NSUN5 promoter, respectively. In (**g**), recovery of C3782 28S rRNA methylation upon NSUN5 transfection in the epigenetically silenced LN229 cells.

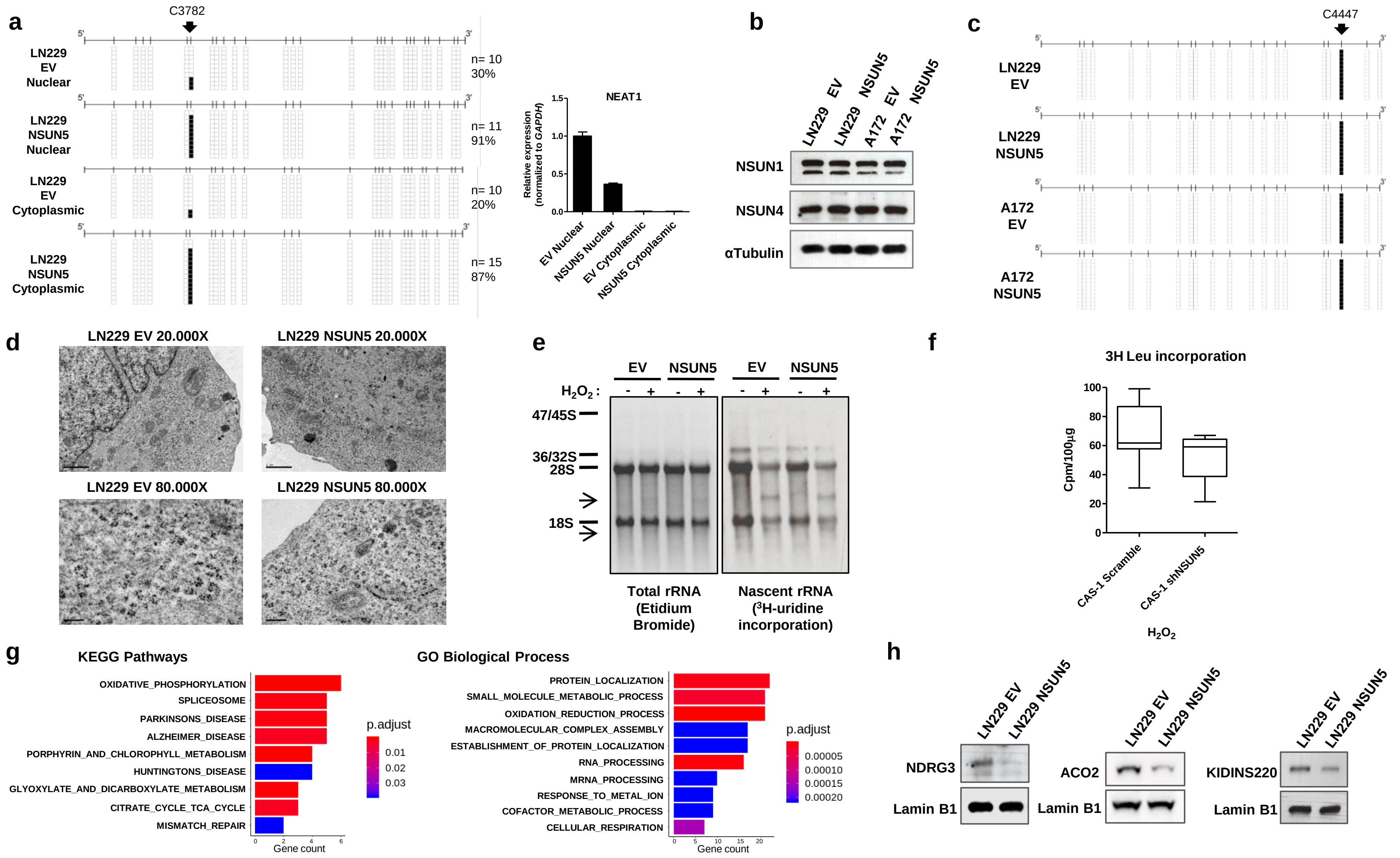

Figure S5. Related to Figure 3 and Figure 5. (**a**) RNA bisulfite sequencing of 28S rRNA in the nuclear or cytoplasmic compartments of empty vector (EV) and NSUN5-transfected LN229 glioma cells (*left panel*). Cytosines are represented as short vertical lines and the C3782 site is represented as a long black arrow. Nuclear long non-coding RNA NEAT1 expression analyzed by real-time qPCR, to validate the nuclear-cytosol fractions isolation (*right panel*) (**b**) Western blot to show how the restoration of NSUN5 protein expression upon stable transfection in A172 and LN299 glioma cells does not change the protein levels of the other two ribosomal RNA methyltransferases, NSUN1 and NSUN4. (**c**) RNA bisulfite sequencing at 28S rRNA related NSUN1 methyltransferase position of empty vector (EV) and NSUN5-transfected LN229 and A172 glioma cells. (**d**) NSUN5 restoration in LN229 cells with hypermethylation-associated silencing of NSUN5 does not induce major effects in ribosome distribution, rRNA maturation or total rRNA levels, observed by transmission electron microscopy. (**e**) Northern hybridizations of total RNA extracted from empty vector (EV) or NSUN5 transfected LN229 cells treated with hydrogen peroxide (H2O2) at 100 µM. Total rRNA was visualized by Ethdium bromide (*left*) and Nascent rRNA was visualized by 3H-uridine incorporation (*right*). The position of mature rRNA species is shown on the left of the pictures, and arrows specify major degradation products. (**f**) 3H-Leucine incorporation in CAS-1 cell line transfected with scramble or shNSUN5. (**g**) Gene set enrichment analysis (GSEA) of the proteins with different expression in the SILAC approach upon NSUN5 restoration in LN229 cells (hypergeometric test with a FDR adjusted p-value < 0.05). (**h**) Western blot of proteins down-regulated upon NSUN5 transfection according to SILAC results.

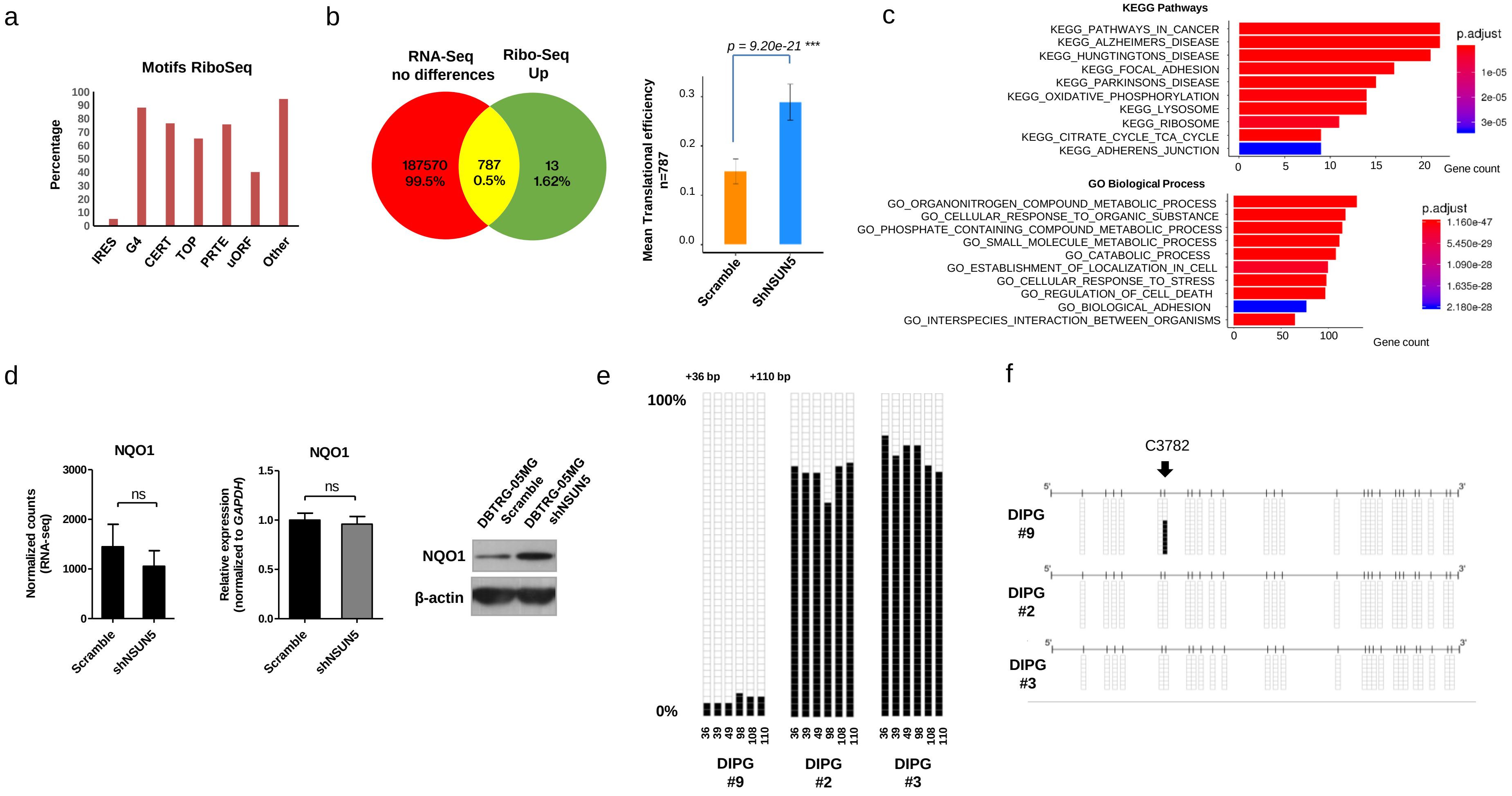

Figure S6. Related to Figure 5. (**a**) Distribution of known regulators motifs in the RNAs undergoing translational enhancement in NSUN5 deficient cells. (**b**) Translational efficiency upon NSUN5 shRNA mediated depletion in DBTRG-05MG cells. Comparing RNA-seq and ribosome-protected RNA (Ribo-seq) deep sequencing profiles, we showed that 787 RNAs did not change in the RNAseq of DBTRG-05MG cells upon NSUN5-depletion, but were upregulated in the Ribo-seq, indicating enhanced translational efficiency. (**c**) Gene set enrichment analysis (GSEA) of the RNAs with increased translational efficiency in NSUN5 deficient cells (hypergeometric test with a FDR adjusted p-value < 0.05). (**d**) Validation of NSUN5 translational regulation of the identified stress-related target NQO1. Its expression at the RNA level determined by RNA-seq counts (*left*) and real-time quantitative PCR (*middle*) do not change upon NSUN5 depletion, but NQO1 is increased at the protein level (*right*) upon NSUN5 depletion. (**e**) NSUN5 promoter methylation in Diffuse Intrinsic Pontine Glioma (DIPG). Methylation levels of six CpGs of the NSUN5 promoter CpG island (+36 bp to +110 bp from the transcription start site) determined by pyrosequencing in three DIPG cases (#9, #2 and #3). Black and white squares represent methylated or unmethylated status, respectively. (**f**) RNA bisulfite sequencing of 28S RNA in the studied primary DIPG patients. NSUN5 hypermethylation (#2 and #3 cases) is associated with an unmethylated C3782 site of 28S RNA, whereas the #9 NSUN5 unmethylated patient shows methylation of the C3782 position of ribosomal RNA.

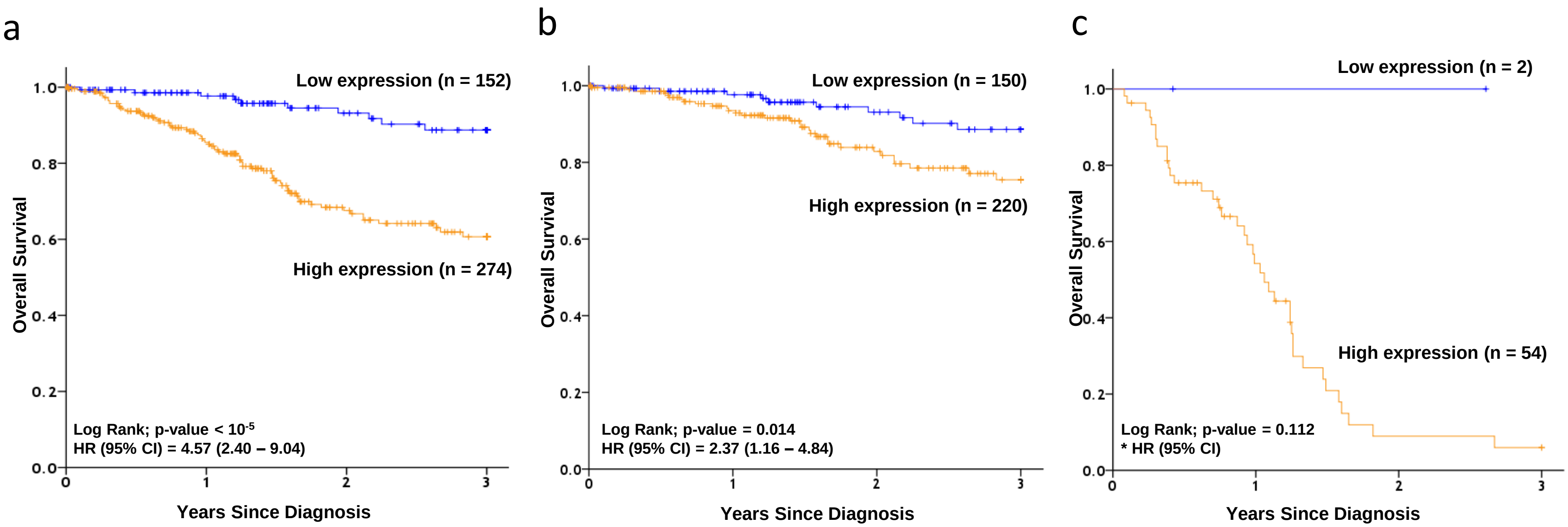

Figure S7. Related to Figure 7. Overall Survival of glioma TCGA datasets with available information for RNA expression of NSUN5, considering (**a**) all grades gliomas (N = 426), (**b**) low-grade gliomas (N = 370) and (**c**) glioblastomas (N = 56). P-value (p) is calculated using Log-rank function. Univariate Cox Regression analysis is represented by Hazard Ratio (HR) with a 95% of confidence interval (95%CI). P under 0.05 is considered as statistically significant. *HR (95% CI) = Hazard Ratio cannot be calculated since lack of events (dead) in low expression group.

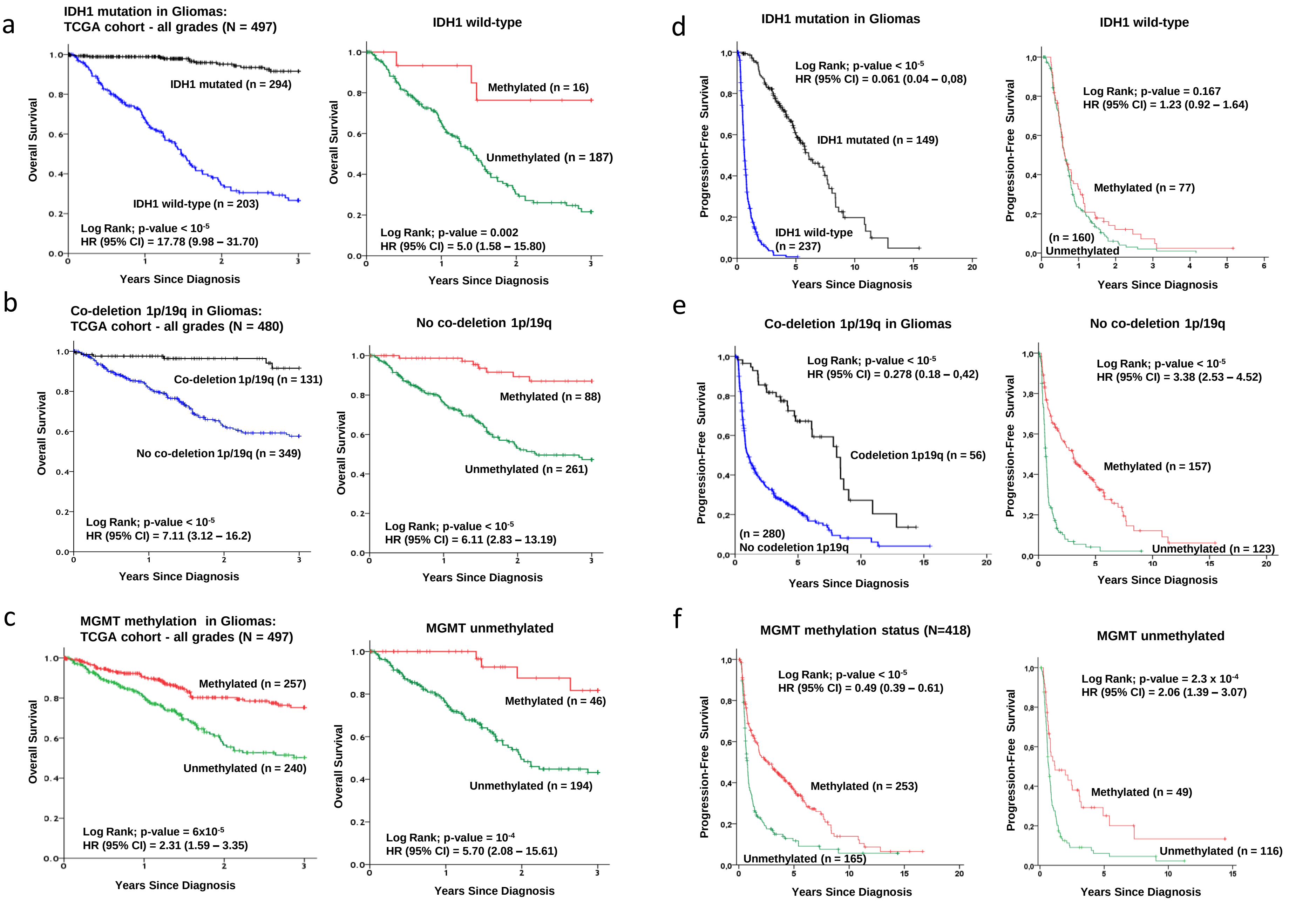

Figure S8. Related to Figure 7. (**a**) Kaplan-Meier analysis of Overall Survival (OS) across all glioma grades according to IDH1 mutational, co-deletion 1p/19q (**b**) and MGMT methylation (**c**) status. (**d**) Kaplan-Meier analysis of Progression-Free Survival (PFS) across all glioma grades according to IDH1 mutational, co-deletion 1p/19q (**e**) and MGMT methylation (**f**) status. Significance of the log-rank test is shown. Results of the univariate Cox regression analysis are represented by the Hazards Ratio (HR) and 95% Confidence Interval (CI). P under 0.05 is considered as statistically significant.

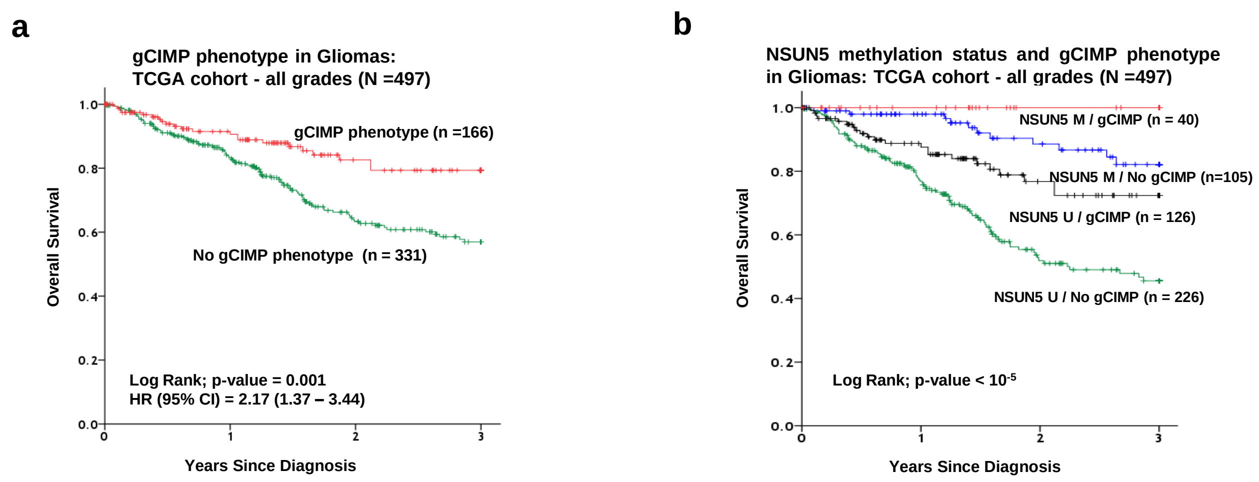

Figure S9. Related to Figure 7. (**a**) Kaplan-Meier analysis in glioma TCGA dataset of Overall Survival across all glioma grades according to gCIMP phenotype, and combination of gCIMP with NSUN5 methylation status (**b**). P-value (P) is calculated using Log-rank function. Univariate Cox Regression analysis is represented by hazard ratio (HR) with a 95% of confidence interval (95%CI). P under 0.05 is considered as statistically significant.

Table S1. Ribo-seq and RNA-seq fold-change and p-values for RNAs with higher translational efficiency in LN229 NSUN5 deficient cells. Related to Figure 5. In the absence (A) or presence (B) of H_2O_2 stress.

A. Ribo-seq and RNA-seq fold-change and p-values for RNAs with higher translational efficiency in LN229 NSUN5 deficient cells in absence of H_2O_2 stress

		Ribo-Seq						RNA-Seq					
**EnsemblID**	**Gene Name**	**Base Mean**	**log2Fold Change**	**Lfc SE**	**stat**	**pvalue**	**padj**	**Base Mean**	**log2Fold Change**	**Lfc SE**	**stat**	**pvalue**	**padj**
ENST00000318602	A2M	137.34	-2.87	0.36	-7.98	1.41E-15	7.97E-13	2757.02	-0.76	0.43	-1.76	0.078	0.648
ENST00000462568	A2M	11.71	-6.68	1.82	-3.67	2.40E-04	7.14E-03	39.90	-1.09	0.65	-1.68	0.093	0.704
ENST00000495709	A2M	4.82	-3.49	1.06	-3.30	9.58E-04	2.29E-02	19.80	0.24	1.01	0.24	0.809	1.000
ENST00000569825	AARS	32.52	-8.16	1.15	-7.09	1.30E-12	1.19E-10	311.73	0.14	0.31	0.45	0.654	1.000
ENST00000374736	ABCA1	11.84	-1.31	0.50	-2.64	8.34E-03	1.21E-01	3483.16	0.18	0.13	1.31	0.190	0.884
ENST00000454898	ABCD3	17.30	-22.08	3.03	-7.29	3.02E-13	4.06E-11	79.13	0.63	1.71	0.37	0.710	1.000
ENST00000484213	ABCD3	19.14	-22.22	3.03	-7.34	2.15E-13	3.07E-11	3060.63	-0.06	0.24	-0.24	0.806	1.000
ENST00000512653	ABL2	9.25	-21.24	3.03	-7.01	2.36E-12	1.88E-10	36.87	0.21	0.68	0.31	0.757	1.000
ENST00000431957	AC000110.1.1	50.19	-1.03	0.39	-2.65	8.01E-03	1.18E-01	1.69	-0.19	4.40	-0.04	0.966	NA
ENST00000458451	AC004014.4.1	32.43	-1.22	0.31	-3.91	9.30E-05	3.11E-03	1.20	-0.40	4.46	-0.09	0.928	NA
ENST00000427861	AC004074.3.1	54.25	-0.94	0.23	-4.08	4.56E-05	1.64E-03	70.15	-0.20	0.60	-0.34	0.733	1.000
ENST00000438989	AC007041.2.1	54.02	-0.77	0.27	-2.89	3.90E-03	7.01E-02	48.52	-0.22	0.52	-0.43	0.670	1.000
ENST00000503102	AC008427.2.1	19.87	-1.48	0.38	-3.94	8.26E-05	2.80E-03	3.73	-0.45	1.63	-0.28	0.780	NA
ENST00000456481	AC009245.3.1	344.39	-1.86	0.48	-3.86	1.12E-04	3.64E-03	601.72	0.02	0.19	0.10	0.921	1.000
ENST00000448535	AC009963.5.1	7.38	-2.85	1.08	-2.64	8.20E-03	1.20E-01	1.13	-0.19	4.59	-0.04	0.967	NA
ENST00000441321	AC016691.2.1	14.32	-1.85	0.46	-4.05	5.11E-05	1.82E-03	10.40	-0.75	1.29	-0.58	0.561	1.000
ENST00000399515	AC019178.1.1	219.35	-0.62	0.19	-3.21	1.33E-03	2.99E-02	395.40	0.12	0.26	0.45	0.653	1.000
ENST00000554351	AC027323.1	14.59	-1.66	0.47	-3.55	3.84E-04	1.07E-02	1229.88	0.59	0.56	1.06	0.289	0.966
ENST00000453625	AC074182.1.1	105.24	-0.87	0.32	-2.67	7.48E-03	1.13E-01	32.74	-0.07	0.54	-0.13	0.894	1.000
ENST00000431445	AC087650.1.1	5.20	-1.84	0.66	-2.78	5.37E-03	8.90E-02	1.37	-0.01	2.65	0.00	0.997	NA
ENST00000419844	AC091492.2.1	27.17	-1.48	0.44	-3.40	6.68E-04	1.71E-02	4.41	-0.27	1.44	-0.19	0.850	1.000
ENST00000431248	AC093106.5.1	23.60	-1.58	0.50	-3.17	1.51E-03	3.32E-02	7.93	-1.19	1.01	-1.17	0.243	0.936
ENST00000445713	AC093106.7.1	17.40	-1.27	0.49	-2.59	9.57E-03	1.33E-01	294.30	-0.23	0.20	-1.15	0.251	0.943
ENST00000516420	AC098691.1	4.61	-2.16	0.77	-2.79	5.34E-03	8.87E-02	54041.23	-0.35	0.11	-3.14	0.002	0.056
ENST00000427559	AC104651.2.1	13.88	-1.41	0.41	-3.43	6.09E-04	1.58E-02	0.79	3.03	4.85	0.62	0.532	NA
ENST00000457257	AC174470.1	9.86	-4.36	1.42	-3.07	2.16E-03	4.43E-02	1371.51	-0.21	0.22	-0.92	0.357	0.997
ENST00000326793	ACAP2	72.44	-0.93	0.36	-2.62	8.68E-03	1.25E-01	8363.49	-0.02	0.17	-0.14	0.888	1.000
ENST00000478010	ACO2	3.64	-5.00	1.70	-2.94	3.31E-03	6.22E-02	104.29	0.35	0.55	0.64	0.521	1.000
ENST00000449612	ACOT9	5.82	-5.69	1.13	-5.03	4.80E-07	2.21E-05	38.56	-0.79	1.20	-0.65	0.513	1.000
ENST00000293217	ACOX1	21.34	-22.37	3.03	-7.39	1.49E-13	2.28E-11	3659.20	-0.96	0.63	-1.52	0.129	0.790
ENST00000525230	ACP2	8.44	-6.21	1.76	-3.52	4.25E-04	1.16E-02	121.19	1.82	0.66	2.78	0.005	0.134
ENST00000357430	ACSL3	197.47	-3.17	1.03	-3.08	2.07E-03	4.28E-02	29400.69	-0.07	0.11	-0.62	0.532	1.000
ENST00000425660	ACTB	268.10	-25.81	3.03	-8.53	1.48E-17	1.72E-14	24273.07	-1.78	0.96	-1.85	0.064	0.593
ENST00000443297	ACTR3	38.78	-3.77	1.36	-2.77	5.61E-03	9.17E-02	82.54	-0.20	0.71	-0.28	0.778	1.000
ENST00000470269	ADAM10	23.47	-7.69	1.08	-7.15	8.59E-13	8.66E-11	251.76	0.00	0.28	-0.01	0.994	1.000
ENST00000310823	ADAM17	158.76	-0.78	0.28	-2.75	5.88E-03	9.52E-02	8755.08	0.14	0.14	0.95	0.344	0.991
ENST00000238561	ADCK1	3.45	-3.16	1.06	-2.99	2.80E-03	5.44E-02	354.53	-1.94	0.99	-1.97	0.049	0.521
ENST00000366779	ADCK3	27.81	-1.53	0.57	-2.68	7.47E-03	1.13E-01	4163.39	0.14	0.27	0.51	0.612	1.000
ENST00000260600	ADCY3	41.85	-1.12	0.39	-2.87	4.15E-03	7.34E-02	3951.26	0.71	0.48	1.48	0.139	0.809
ENST00000488837	ADD3	22.63	-1.23	0.40	-3.08	2.05E-03	4.25E-02	169.66	-0.30	0.66	-0.45	0.651	1.000
ENST00000495562	ADIPOR1	7.82	-6.10	1.81	-3.36	7.69E-04	1.92E-02	3304.36	3.65	1.47	2.48	0.013	0.241
ENST00000372734	ADK	166.33	-1.11	0.32	-3.42	6.33E-04	1.64E-02	1956.87	0.48	0.62	0.77	0.439	1.000
ENST00000342312	ADSL	96.29	-4.66	1.30	-3.58	3.46E-04	9.75E-03	5795.02	-0.23	0.14	-1.60	0.110	0.751
ENST00000468215	ADSS	9.53	-21.27	3.03	-7.02	2.16E-12	1.75E-10	205.65	0.56	0.42	1.33	0.185	0.879
ENST00000484865	AGPAT3	4.61	-2.64	0.97	-2.74	6.23E-03	9.92E-02	107.91	-0.24	0.33	-0.74	0.460	1.000
ENST00000264409	AGPAT9	139.91	-5.23	1.41	-3.70	2.11E-04	6.40E-03	3451.37	0.09	0.24	0.38	0.707	1.000
ENST00000357081	AGTPBP1	15.46	-1.42	0.48	-2.95	3.15E-03	5.97E-02	1051.04	-0.48	0.18	-2.62	0.009	0.190
ENST00000217426	AHCY	351.74	-11.59	1.01	-11.52	1.05E-30	8.84E-27	13466.08	0.11	0.35	0.32	0.749	1.000
ENST00000481423	AHCYL1	90.61	-1.46	0.49	-2.98	2.84E-03	5.49E-02	35.76	1.38	0.53	2.61	0.009	0.194
ENST00000555473	AHSA1	32.31	-8.15	2.29	-3.55	3.81E-04	1.06E-02	162.11	0.00	0.82	0.00	0.997	1.000

ENST00000394701	AIMP1	183.86	-0.70	0.21	-3.28	1.04E-03	2.46E-02	2450.14	0.12	0.20	0.57	0.568	1.000
ENST00000550143	AK1	8.82	-21.17	3.03	-6.99	2.76E-12	2.08E-10	22.14	-0.16	0.90	-0.18	0.859	1.000
ENST00000025301	AKAP11	130.40	-0.84	0.28	-3.02	2.54E-03	5.05E-02	13090.47	-0.07	0.17	-0.42	0.672	1.000
ENST00000475985	AKR1A1	3.96	-5.12	1.69	-3.02	2.52E-03	5.03E-02	54.11	-0.39	0.39	-1.00	0.315	0.980
ENST00000366539	AKT3	129.03	-0.87	0.19	-4.46	8.06E-06	3.29E-04	30568.00	0.03	0.13	0.22	0.822	1.000
ENST00000456007	AL133244.1.1	3.86	-2.04	0.79	-2.60	9.33E-03	1.31E-01	1.01	1.64	4.60	0.36	0.721	NA
ENST00000558098	AL589988.1	9.51	-21.27	3.03	-7.02	2.17E-12	1.76E-10	14.59	-0.12	0.76	-0.16	0.872	1.000
ENST00000310271	ALAS1	10.88	-21.46	3.03	-7.08	1.40E-12	1.26E-10	531.82	-0.23	0.25	-0.90	0.366	1.000
ENST00000346623	ALDH1A3	281.48	-25.87	3.03	-8.55	1.24E-17	1.58E-14	24700.53	2.06	1.74	1.18	0.237	0.931
ENST00000561338	ALDH1A3	17.42	-22.09	3.03	-7.30	2.94E-13	3.97E-11	92.55	-0.84	0.90	-0.94	0.350	0.994
ENST00000538148	ALDH9A1	34.01	-8.22	3.03	-2.72	6.60E-03	1.03E-01	159.12	-1.31	0.90	-1.46	0.144	0.817
ENST00000564595	ALDOA	21.07	-21.05	3.03	-6.95	3.57E-12	2.54E-10	1186.32	-0.14	0.28	-0.49	0.624	1.000
ENST00000371108	ALG6	58.44	-1.58	0.50	-3.13	1.73E-03	3.71E-02	7821.02	0.04	0.13	0.30	0.764	1.000
ENST00000540305	ALKBH2	3.24	-4.84	1.15	-4.20	2.67E-05	1.02E-03	514.32	-0.11	0.26	-0.41	0.683	1.000
ENST00000428149	ALKBH8	12.86	-2.44	0.72	-3.40	6.77E-04	1.73E-02	531.36	0.35	0.41	0.84	0.401	1.000
ENST00000512673	ALYREF	18.98	-7.38	1.65	-4.48	7.50E-06	3.07E-04	182.85	0.85	0.57	1.49	0.136	0.804
ENST00000382085	AMACR	30.07	-8.05	2.71	-2.97	2.99E-03	5.72E-02	631.62	-0.63	0.73	-0.87	0.387	1.000
ENST00000281471	AMN1	7.69	-2.71	0.87	-3.13	1.77E-03	3.77E-02	695.24	0.41	0.27	1.49	0.137	0.805
ENST00000354910	ANAPC13	30.50	-22.85	3.03	-7.55	4.41E-14	9.69E-12	537.35	-0.98	0.72	-1.36	0.173	0.861
ENST00000265140	ANKRD32	28.17	-1.22	0.33	-3.68	2.30E-04	6.90E-03	3214.92	-0.03	0.28	-0.12	0.907	1.000
ENST00000448801	ANKRD44	13.73	-6.92	1.20	-5.76	8.63E-09	4.42E-07	311.08	-0.18	0.28	-0.62	0.533	1.000
ENST00000493808	ANKRD44	2.30	-4.35	1.15	-3.79	1.50E-04	4.73E-03	54.69	0.49	0.59	0.82	0.412	1.000
ENST00000414522	ANO10	9.26	-6.34	1.08	-5.89	3.85E-09	2.00E-07	28.58	0.94	1.17	0.80	0.421	1.000
ENST00000425752	ANO6	18.64	-7.35	2.20	-3.35	8.08E-04	1.99E-02	43.30	0.96	2.75	0.35	0.728	1.000
ENST00000409628	ANP32A	38.70	-23.16	3.03	-7.65	1.97E-14	5.52E-12	4918.05	-0.19	0.13	-1.43	0.152	0.830
ENST00000495420	ANP32A	11.21	-6.62	2.11	-3.14	1.71E-03	3.67E-02	146.88	0.00	0.27	-0.02	0.986	1.000
ENST00000561430	ANP32A	13.90	-6.93	1.82	-3.80	1.42E-04	4.47E-03	59.72	-0.23	0.53	-0.44	0.663	1.000
ENST00000486769	ANP32B	8.60	-2.43	0.67	-3.61	3.01E-04	8.72E-03	98.84	0.04	0.37	0.12	0.906	1.000
ENST00000369119	ANP32E	31.39	-22.69	3.03	-7.49	6.64E-14	1.31E-11	856.27	-0.81	0.37	-2.19	0.029	0.391
ENST00000559350	ANXA2	28.95	-7.90	2.87	-2.75	5.90E-03	9.54E-02	552.97	1.87	1.00	1.88	0.060	0.575
ENST00000560165	ANXA2	4.84	-5.41	1.98	-2.73	6.39E-03	1.01E-01	409.55	0.12	0.43	0.28	0.778	1.000
ENST00000501272	ANXA5	59.87	-23.77	3.03	-7.85	4.09E-15	1.71E-12	3755.86	0.25	0.20	1.24	0.214	0.910
ENST00000517486	ANXA6	13.82	-6.92	1.15	-6.00	1.93E-09	1.02E-07	22.31	-0.97	0.81	-1.19	0.235	0.929
ENST00000372921	ANXA7	10.31	-21.35	3.03	-7.05	1.79E-12	1.51E-10	5053.42	-0.09	0.25	-0.34	0.735	1.000
ENST00000535178	ANXA7	36.44	-23.08	3.03	-7.63	2.42E-14	6.28E-12	302.33	-0.22	0.37	-0.58	0.563	1.000
ENST00000565412	AP1G1	4.77	-5.39	1.72	-3.13	1.77E-03	3.76E-02	17.50	0.43	0.66	0.65	0.513	1.000
ENST00000292807	AP2M1	14.32	-6.98	2.60	-2.68	7.34E-03	1.11E-01	15141.34	0.04	0.21	0.20	0.844	1.000
ENST00000439647	AP2M1	20.57	-7.50	1.99	-3.77	1.65E-04	5.13E-03	2090.18	0.49	0.15	3.35	0.001	0.031
ENST00000461733	AP2M1	20.95	-7.52	1.92	-3.92	8.91E-05	3.00E-03	377.03	-0.36	0.23	-1.58	0.115	0.762
ENST00000468048	AP2M1	5.88	-5.69	1.72	-3.32	9.10E-04	2.20E-02	92.99	-0.32	0.35	-0.91	0.361	0.999
ENST00000343267	APOD	210.46	-1.32	0.32	-4.15	3.34E-05	1.24E-03	6820.88	0.23	0.31	0.73	0.468	1.000
ENST00000348990	APP	150.35	-25.02	3.03	-8.27	1.36E-16	1.16E-13	9743.12	0.33	0.27	1.23	0.217	0.913
ENST00000354192	APP	168.29	-8.65	2.98	-2.91	3.64E-03	6.68E-02	9160.53	0.30	0.14	2.24	0.025	0.365
ENST00000439274	APP	94.80	-8.76	3.03	-2.89	3.79E-03	6.88E-02	735.88	0.01	0.27	0.03	0.976	1.000
ENST00000288266	APPL1	87.71	-1.28	0.33	-3.87	1.09E-04	3.55E-03	7558.82	-0.07	0.16	-0.42	0.677	1.000
ENST00000264028	ARCN1	26.85	-22.68	3.03	-7.49	6.77E-14	1.33E-11	17149.43	0.21	0.17	1.27	0.204	0.899
ENST00000000233	ARF5	48.66	-23.47	3.03	-7.76	8.83E-15	2.89E-12	3565.22	0.14	0.35	0.41	0.681	1.000
ENST00000532478	ARFGAP2	10.76	-2.69	0.93	-2.89	3.89E-03	7.00E-02	9.19	-0.67	1.40	-0.48	0.631	1.000
ENST00000400198	ARGLU1	71.05	-1.03	0.38	-2.71	6.69E-03	1.04E-01	2557.21	-0.08	0.15	-0.53	0.595	1.000
ENST00000529960	ARHGAP1	15.58	-7.10	2.66	-2.67	7.64E-03	1.14E-01	12.06	-0.40	0.78	-0.52	0.602	1.000
ENST00000303665	ARHGAP17	7.38	-20.93	3.03	-6.91	4.91E-12	3.20E-10	1732.30	-1.72	0.21	-7.99	0.000	0.000
ENST00000539826	ARHGAP5	5.63	-4.40	1.63	-2.70	6.91E-03	1.06E-01	305.41	0.87	0.96	0.91	0.361	0.999
ENST00000350026	ARID1B	7.15	-20.87	3.03	-6.89	5.59E-12	3.55E-10	12.85	-3.26	2.06	-1.58	0.113	0.757
ENST00000334344	ARID2	91.14	-0.81	0.28	-2.84	4.49E-03	7.78E-02	7610.28	0.16	0.26	0.62	0.536	1.000
ENST00000452385	ARIH2	6.84	-20.28	3.03	-6.69	2.19E-11	1.21E-09	1598.04	0.01	0.20	0.04	0.968	1.000
ENST00000492077	ARIH2	14.95	-7.04	2.66	-2.64	8.25E-03	1.20E-01	106.73	-0.19	0.42	-0.44	0.658	1.000
ENST00000549283	ARL1	7.47	-20.95	3.03	-6.91	4.70E-12	3.08E-10	8.80	0.05	1.35	0.04	0.972	1.000
ENST00000529254	ARL2	80.02	-3.82	1.46	-2.61	9.10E-03	1.29E-01	73.03	-0.60	0.78	-0.76	0.444	1.000

ENST00000455875	ARL6IP6	7.48	-3.11	1.14	-2.73	6.29E-03	1.00E-01	48.16	0.18	0.78	0.23	0.818	1.000
ENST00000441711	ARMC10	1.74	-3.91	1.22	-3.20	1.39E-03	3.10E-02	664.67	0.58	0.39	1.50	0.132	0.797
ENST00000470146	ARPC2	50.38	-7.64	1.59	-4.80	1.57E-06	6.92E-05	5951.30	0.27	0.74	0.36	0.721	1.000
ENST00000294742	ARPC5	14.56	-1.42	0.38	-3.70	2.19E-04	6.60E-03	1345.85	0.93	1.36	0.68	0.497	1.000
ENST00000539528	ASB8	11.76	-6.69	1.89	-3.54	3.99E-04	1.10E-02	271.06	0.52	0.65	0.79	0.428	1.000
ENST00000317168	ASCC1	5.78	-20.60	3.03	-6.80	1.06E-11	6.11E-10	103.19	-1.41	1.03	-1.37	0.170	0.857
ENST00000369162	ASCC3	140.04	-0.67	0.23	-2.95	3.22E-03	6.05E-02	4899.85	0.08	0.15	0.50	0.619	1.000
ENST00000229595	ASF1A	84.23	-0.68	0.22	-3.10	1.93E-03	4.04E-02	1479.34	-0.03	0.17	-0.21	0.836	1.000
ENST00000260952	ASNSD1	26.10	-1.29	0.45	-2.85	4.37E-03	7.64E-02	2199.80	-0.11	0.20	-0.56	0.578	1.000
ENST00000521499	ASPH	18.28	-3.61	1.31	-2.76	5.79E-03	9.40E-02	18.41	0.37	0.95	0.39	0.695	1.000
ENST00000261191	ASUN	72.59	-0.74	0.27	-2.69	7.05E-03	1.08E-01	1827.19	1.22	0.75	1.62	0.105	0.736
ENST00000328142	ATAD1	45.31	-3.61	0.93	-3.87	1.10E-04	3.58E-03	5790.84	0.34	0.25	1.38	0.167	0.851
ENST00000521903	ATAD2	4.28	-3.42	1.22	-2.81	4.91E-03	8.31E-02	684.58	0.51	0.91	0.56	0.575	1.000
ENST00000396680	ATF4	10.65	-21.43	3.03	-7.07	1.50E-12	1.33E-10	2878.80	-0.42	0.44	-0.95	0.343	0.991
ENST00000540793	ATF7IP	11.72	-21.55	3.03	-7.12	1.12E-12	1.05E-10	1418.21	1.64	1.03	1.59	0.111	0.754
ENST00000379594	ATG12	5.00	-4.84	1.87	-2.59	9.72E-03	1.35E-01	67.13	-0.43	0.45	-0.96	0.335	0.988
ENST00000496423	ATG3	17.52	-7.27	1.50	-4.85	1.25E-06	5.55E-05	47.04	-0.17	0.46	-0.38	0.707	1.000
ENST00000372246	ATG4A	4.01	-3.44	1.19	-2.88	3.97E-03	7.11E-02	199.12	1.24	0.89	1.40	0.163	0.845
ENST00000405546	ATG4B	9.35	-4.47	1.68	-2.66	7.77E-03	1.16E-01	285.09	-0.03	0.26	-0.11	0.911	1.000
ENST00000354449	ATG7	55.53	-0.90	0.33	-2.73	6.32E-03	1.00E-01	2732.18	0.02	0.21	0.12	0.908	1.000
ENST00000446622	ATIC	23.85	-3.35	0.85	-3.96	7.48E-05	2.57E-03	388.71	0.11	0.26	0.43	0.668	1.000
ENST00000402054	ATL2	32.24	-5.84	1.86	-3.13	1.72E-03	3.68E-02	48.49	-0.39	0.56	-0.70	0.484	1.000
ENST00000452699	ATP13A2	15.21	-1.02	0.37	-2.77	5.54E-03	9.10E-02	263.02	1.55	0.38	4.11	0.000	0.002
ENST00000484727	ATP1B3	6.64	-20.76	3.03	-6.85	7.37E-12	4.51E-10	373.83	-2.84	1.27	-2.24	0.025	0.365
ENST00000539276	ATP2A2	152.33	-25.04	3.03	-8.27	1.31E-16	1.14E-13	19945.57	0.26	0.17	1.55	0.120	0.775
ENST00000282050	ATP5A1	36.23	-23.08	3.03	-7.63	2.43E-14	6.28E-12	10449.92	6.66	1.98	3.37	0.001	0.029
ENST00000471790	ATP5A1	12.16	-2.84	0.89	-3.18	1.48E-03	3.26E-02	34.44	0.77	0.54	1.43	0.154	0.834
ENST00000484856	ATP5A1	69.47	-23.97	3.03	-7.92	2.38E-15	1.11E-12	163.45	-3.02	1.68	-1.80	0.071	0.623
ENST00000547808	ATP5B	18.81	-22.19	3.03	-7.33	2.30E-13	3.22E-11	637.51	4.59	1.25	3.66	0.000	0.012
ENST00000441945	ATP5C1P1	51.26	-1.85	0.40	-4.57	4.95E-06	2.07E-04	4.17	-1.14	2.27	-0.51	0.613	1.000
ENST00000395633	ATP5D	49.85	-8.77	1.03	-8.54	1.37E-17	1.65E-14	1917.09	-0.13	0.23	-0.55	0.585	1.000
ENST00000243997	ATP5E	269.93	-1.10	0.30	-3.73	1.94E-04	5.94E-03	30476.69	-0.05	0.11	-0.45	0.654	1.000
ENST00000468818	ATP5F1	101.35	-7.36	2.72	-2.71	6.79E-03	1.05E-01	6283.03	0.55	0.21	2.62	0.009	0.189
ENST00000483994	ATP5F1	108.81	-24.58	3.03	-8.12	4.58E-16	3.26E-13	39.24	0.36	0.54	0.66	0.509	1.000
ENST00000355938	ATP5G1	6.05	-2.83	1.08	-2.62	8.78E-03	1.26E-01	3372.38	-0.05	0.14	-0.38	0.704	1.000
ENST00000515116	ATP5I	109.66	-1.93	0.61	-3.16	1.59E-03	3.46E-02	40.99	0.03	0.45	0.07	0.941	1.000
ENST00000527186	ATP5L	30.36	-2.15	0.81	-2.67	7.60E-03	1.14E-01	257.97	0.29	0.23	1.24	0.216	0.912
ENST00000544975	ATP6AP2	63.56	-23.67	3.03	-7.82	5.27E-15	1.97E-12	606.75	0.17	0.41	0.42	0.672	1.000
ENST00000330398	ATP6V0C	25.37	-2.29	0.83	-2.77	5.57E-03	9.12E-02	3284.48	0.79	0.69	1.16	0.247	0.939
ENST00000566322	ATP6V0D1	13.98	-21.79	3.03	-7.20	6.21E-13	6.95E-11	13.20	-2.32	1.14	-2.03	0.042	0.482
ENST00000273398	ATP6V1A	196.94	-0.67	0.22	-3.02	2.52E-03	5.02E-02	4517.01	-0.51	0.54	-0.95	0.344	0.991
ENST00000557244	ATP6V1D	5.21	-5.52	1.30	-4.25	2.17E-05	8.44E-04	7.73	0.73	1.09	0.67	0.502	1.000
ENST00000460085	ATP6V1E1	92.97	-9.67	1.10	-8.75	2.06E-18	3.29E-15	4.31	-3.00	2.15	-1.39	0.163	0.846
ENST00000374050	ATP6V1G1	162.29	-1.08	0.22	-4.94	7.85E-07	3.55E-05	5942.45	0.25	0.14	1.85	0.065	0.596
ENST00000350721	ATR	61.79	-0.94	0.35	-2.69	7.22E-03	1.10E-01	5195.43	0.26	0.19	1.42	0.156	0.839
ENST00000446916	ATRN	29.56	-4.22	1.48	-2.86	4.23E-03	7.46E-02	19082.79	-0.26	0.25	-1.03	0.301	0.973
ENST00000359092	AXL	9.47	-21.27	3.03	-7.02	2.20E-12	1.77E-10	6827.72	-0.05	0.15	-0.34	0.737	1.000
ENST00000465740	B4GALT3	3.75	-2.88	1.07	-2.69	7.11E-03	1.09E-01	350.44	-0.08	0.29	-0.27	0.790	1.000
ENST00000464126	BAG6	15.95	-7.13	2.62	-2.72	6.45E-03	1.01E-01	24.25	0.17	0.70	0.24	0.811	1.000
ENST00000428708	BAIAP2	15.52	-21.94	3.03	-7.24	4.34E-13	5.32E-11	2479.30	-0.13	1.24	-0.11	0.915	1.000
ENST00000355163	BANP	3.32	-3.61	1.17	-3.07	2.11E-03	4.35E-02	153.00	0.51	2.46	0.21	0.834	1.000
ENST00000391871	BAX	7.84	-21.01	3.03	-6.94	4.05E-12	2.77E-10	66.84	-0.18	0.42	-0.43	0.666	1.000
ENST00000566210	BBS2	1.94	-4.08	1.44	-2.84	4.47E-03	7.74E-02	22.70	1.88	0.83	2.25	0.024	0.358
ENST00000506636	BBS7	13.65	-6.91	2.59	-2.66	7.77E-03	1.16E-01	1796.54	0.19	0.16	1.22	0.223	0.917
ENST00000491150	BCAP29	43.01	-3.47	0.77	-4.49	7.27E-06	2.99E-04	512.47	-0.01	0.28	-0.03	0.976	1.000
ENST00000368759	BCCIP	24.28	-3.51	1.29	-2.71	6.63E-03	1.03E-01	1213.21	-0.02	0.27	-0.08	0.935	1.000
ENST00000317582	BCL2L13	7.27	-3.03	1.06	-2.87	4.14E-03	7.33E-02	716.05	0.39	0.20	2.01	0.045	0.495
ENST00000543382	BECN1	65.79	-1.12	0.29	-3.93	8.39E-05	2.84E-03	5378.48	0.03	0.13	0.20	0.843	1.000

ENST00000563971	BFAR	13.88	-6.93	2.62	-2.64	8.21E-03	1.20E-01	33.09	-0.04	0.81	-0.05	0.959	1.000
ENST00000331595	BGN	32.55	-1.97	0.34	-5.75	8.72E-09	4.46E-07	7800.59	-0.87	0.17	-5.23	0.000	0.000
ENST00000355112	BLM	109.72	-0.60	0.21	-2.82	4.73E-03	8.08E-02	1278.41	-0.50	0.58	-0.85	0.394	1.000
ENST00000367807	BLZF1	12.19	-21.61	3.03	-7.14	9.65E-13	9.35E-11	381.97	0.00	0.29	0.00	0.998	1.000
ENST00000414869	BPNT1	16.91	-7.22	1.87	-3.86	1.14E-04	3.70E-03	375.82	-0.91	0.67	-1.36	0.172	0.860
ENST00000544455	BRCA2	38.51	-0.96	0.25	-3.83	1.29E-04	4.12E-03	6.96	-1.11	1.14	-0.98	0.329	0.985
ENST00000369462	BRCC3	3.94	-5.11	1.82	-2.80	5.04E-03	8.49E-02	286.55	-0.79	0.28	-2.86	0.004	0.114
ENST00000463620	BRD8	16.75	-7.20	2.64	-2.73	6.39E-03	1.01E-01	458.18	0.40	0.30	1.32	0.187	0.880
ENST00000259008	BRIP1	43.87	-1.02	0.34	-2.97	2.95E-03	5.67E-02	4871.28	-0.45	0.15	-3.07	0.002	0.067
ENST00000340934	BROX	157.54	-0.76	0.19	-3.89	1.00E-04	3.31E-03	5611.84	0.22	0.17	1.31	0.189	0.883
ENST00000367846	BRP44	42.38	-0.62	0.24	-2.59	9.52E-03	1.32E-01	1429.74	0.24	0.35	0.70	0.483	1.000
ENST00000353555	BSG	1151.34	-13.30	1.06	-12.60	2.02E-36	4.25E-32	55676.35	0.25	0.15	1.65	0.098	0.719
ENST00000252593	BST2	10.89	-6.57	1.06	-6.18	6.45E-10	3.40E-08	2761.31	-1.28	0.13	-10.19	0.000	0.000
ENST00000514360	BTF3	642.50	-1.06	0.27	-3.90	9.57E-05	3.18E-03	9952.02	0.26	0.52	0.51	0.608	1.000
ENST00000230340	BYSL	56.84	-1.38	0.46	-3.02	2.55E-03	5.08E-02	699.72	-0.07	0.19	-0.37	0.708	1.000
ENST00000359893	BZW1	48.15	-5.60	1.45	-3.86	1.13E-04	3.65E-03	88.88	-0.22	0.57	-0.39	0.697	1.000
ENST00000410110	BZW1	65.50	-23.75	3.03	-7.85	4.21E-15	1.73E-12	1758.09	-0.18	0.24	-0.75	0.452	1.000
ENST00000447069	BZW1	57.78	-8.99	2.79	-3.22	1.30E-03	2.94E-02	101.36	-0.24	0.35	-0.67	0.502	1.000
ENST00000452206	BZW1	55.80	-23.09	3.03	-7.63	2.36E-14	6.24E-12	617.69	-0.30	0.53	-0.56	0.575	1.000
ENST00000463310	BZW1	57.90	-23.71	3.03	-7.83	4.70E-15	1.84E-12	89.48	-0.46	0.31	-1.46	0.143	0.816
ENST00000415365	BZW2	4.72	-5.36	1.33	-4.03	5.63E-05	1.98E-03	1003.22	-0.75	0.74	-1.01	0.311	0.979
ENST00000419769	C10orf137	17.81	-7.29	2.17	-3.36	7.91E-04	1.96E-02	306.46	-0.28	1.02	-0.27	0.784	1.000
ENST00000540434	C11orf10	18.25	-3.63	0.73	-4.95	7.51E-07	3.40E-05	147.97	-0.17	0.42	-0.42	0.677	1.000
ENST00000525684	C11orf58	8.98	-21.19	3.03	-7.00	2.62E-12	2.03E-10	122.28	-0.02	0.58	-0.04	0.971	1.000
ENST00000529405	C11orf73	9.44	-6.37	1.77	-3.60	3.21E-04	9.18E-03	162.77	0.04	0.29	0.14	0.893	1.000
ENST00000542080	C12orf4	4.92	-4.19	1.61	-2.61	9.14E-03	1.29E-01	4.37	-5.64	2.52	-2.24	0.025	0.365
ENST00000552951	C12orf45	42.69	-0.90	0.30	-2.97	2.97E-03	5.69E-02	929.52	0.31	0.16	1.93	0.054	0.548
ENST00000550487	C12orf62	27.40	-2.60	0.96	-2.71	6.68E-03	1.04E-01	2884.23	0.13	0.18	0.76	0.450	1.000
ENST00000553888	C14orf133	20.11	-2.74	0.92	-2.99	2.79E-03	5.42E-02	386.30	0.75	0.29	2.57	0.010	0.208
ENST00000483571	C14orf135	2.80	-4.62	1.75	-2.64	8.19E-03	1.20E-01	342.98	0.58	0.62	0.95	0.344	0.991
ENST00000247194	C14orf149	34.64	-1.38	0.47	-2.93	3.36E-03	6.28E-02	914.61	-0.03	0.29	-0.12	0.907	1.000
ENST00000556760	C14orf166	10.42	-21.40	3.03	-7.07	1.60E-12	1.40E-10	3679.16	-0.08	0.42	-0.19	0.848	1.000
ENST00000416151	C15orf23	55.31	-4.80	1.46	-3.28	1.02E-03	2.42E-02	721.89	0.00	0.42	0.01	0.996	1.000
ENST00000560321	C15orf23	13.40	-21.74	3.03	-7.18	7.06E-13	7.46E-11	120.22	0.34	0.41	0.85	0.398	1.000
ENST00000416810	C15orf57	5.73	-20.59	3.03	-6.79	1.10E-11	6.29E-10	525.96	-0.38	0.64	-0.59	0.554	1.000
ENST00000497142	C15orf63	12.25	-21.61	3.03	-7.14	9.57E-13	9.32E-11	81.33	0.27	0.32	0.83	0.404	1.000
ENST00000327237	C16orf58	29.25	-2.62	0.77	-3.42	6.32E-04	1.63E-02	1822.30	-0.45	0.31	-1.47	0.143	0.816
ENST00000565844	C16orf88	19.47	-7.42	1.93	-3.84	1.25E-04	4.02E-03	560.51	-0.06	1.37	-0.04	0.966	1.000
ENST00000442039	C16orf91	12.37	-6.76	1.75	-3.87	1.09E-04	3.55E-03	1018.73	-1.49	0.16	-9.20	0.000	0.000
ENST00000354429	C17orf81	7.47	-20.95	3.03	-6.91	4.70E-12	3.08E-10	221.10	0.10	0.53	0.20	0.845	1.000
ENST00000269194	C18orf21	8.08	-6.15	1.84	-3.35	8.03E-04	1.98E-02	107.71	-0.06	0.36	-0.16	0.869	1.000
ENST00000461488	C19orf42	4.12	-5.17	1.24	-4.19	2.84E-05	1.08E-03	25.91	0.08	0.66	0.13	0.900	1.000
ENST00000535581	C19orf55	2.39	-4.37	1.28	-3.43	6.12E-04	1.59E-02	1465.68	-0.15	0.16	-0.97	0.334	0.987
ENST00000287859	C1orf27	47.18	-2.89	0.88	-3.27	1.06E-03	2.49E-02	3554.41	0.60	0.38	1.59	0.113	0.757
ENST00000372525	C1orf50	2.67	-4.55	1.65	-2.76	5.83E-03	9.46E-02	616.50	0.13	0.17	0.76	0.448	1.000
ENST00000372970	C20orf111	13.73	-19.64	3.03	-6.49	8.74E-11	4.73E-09	109.87	0.32	0.31	1.02	0.309	0.979
ENST00000379283	C20orf30	25.62	-2.20	0.44	-5.01	5.43E-07	2.49E-05	658.94	0.12	0.23	0.50	0.617	1.000
ENST00000377704	C20orf72	13.88	-6.93	2.67	-2.59	9.56E-03	1.33E-01	192.75	0.19	0.50	0.39	0.699	1.000
ENST00000491220	C2orf28	30.66	-1.01	0.34	-3.01	2.60E-03	5.14E-02	8.42	-0.41	0.97	-0.42	0.672	1.000
ENST00000295148	C2orf44	46.76	-0.65	0.24	-2.65	8.07E-03	1.19E-01	2268.36	-0.01	0.16	-0.09	0.927	1.000
ENST00000392290	C2orf47	12.56	-21.65	3.03	-7.15	8.72E-13	8.78E-11	1036.96	0.27	0.63	0.43	0.670	1.000
ENST00000342649	C3orf23	1.91	-4.07	1.25	-3.25	1.14E-03	2.64E-02	221.15	-0.15	0.64	-0.24	0.812	1.000
ENST00000537319	C5orf28	2.06	-4.16	1.49	-2.79	5.30E-03	8.82E-02	159.33	-1.29	0.69	-1.87	0.061	0.580
ENST00000510890	C5orf30	7.31	-3.36	1.14	-2.95	3.18E-03	6.00E-02	2096.47	-1.07	1.66	-0.64	0.522	1.000
ENST00000509253	C6orf108	4.45	-5.29	1.64	-3.23	1.22E-03	2.79E-02	16.39	-1.19	2.88	-0.41	0.680	1.000
ENST00000309930	C7orf10	4.16	-3.38	1.28	-2.64	8.23E-03	1.20E-01	336.92	-0.32	0.45	-0.70	0.481	1.000
ENST00000344417	C7orf26	26.86	-1.24	0.46	-2.71	6.77E-03	1.05E-01	3481.52	-0.15	0.25	-0.58	0.560	1.000
ENST00000297145	C7orf60	20.46	-1.43	0.50	-2.87	4.05E-03	7.20E-02	2202.45	0.05	0.15	0.36	0.720	1.000

ENST00000276704	C8orf76	17.64	-1.69	0.40	-4.22	2.39E-05	9.24E-04	804.24	0.25	0.16	1.57	0.117	0.767
ENST00000376834	C9orf41	12.20	-2.04	0.65	-3.14	1.67E-03	3.60E-02	2396.09	0.14	0.15	0.94	0.345	0.991
ENST00000223864	C9orf46	17.31	-5.59	1.50	-3.73	1.90E-04	5.84E-03	1320.00	0.05	0.14	0.34	0.731	1.000
ENST00000492991	C9orf78	8.83	-21.17	3.03	-6.99	2.75E-12	2.08E-10	24.99	-0.06	0.67	-0.09	0.930	1.000
ENST00000488630	C9orf89	13.48	-5.16	1.59	-3.24	1.18E-03	2.72E-02	612.24	0.04	0.30	0.13	0.893	1.000
ENST00000448105	CALCOCO2	21.52	-6.98	1.82	-3.83	1.29E-04	4.11E-03	138.45	0.56	0.28	2.01	0.044	0.494
ENST00000422748	CALD1	10.31	-21.35	3.03	-7.05	1.79E-12	1.51E-10	26.30	-1.02	0.63	-1.62	0.106	0.740
ENST00000430085	CALD1	64.62	-9.15	1.97	-4.64	3.54E-06	1.51E-04	92.64	-0.36	0.39	-0.93	0.351	0.994
ENST00000482470	CALD1	23.92	-7.72	2.74	-2.82	4.81E-03	8.18E-02	523.91	0.09	0.18	0.49	0.621	1.000
ENST00000260743	CALHM2	2.85	-4.64	1.78	-2.60	9.29E-03	1.31E-01	1780.53	0.11	0.20	0.53	0.595	1.000
ENST00000456319	CALM2	380.21	-3.38	0.98	-3.44	5.80E-04	1.52E-02	369.03	-0.30	0.63	-0.47	0.641	1.000
ENST00000535011	CALU	56.83	-23.69	3.03	-7.83	4.98E-15	1.90E-12	1750.79	-0.42	0.21	-2.04	0.041	0.475
ENST00000296402	CAMK2D	48.92	-8.75	1.17	-7.45	9.04E-14	1.64E-11	541.33	-0.06	0.27	-0.21	0.836	1.000
ENST00000394524	CAMK2D	7.47	-20.95	3.03	-6.91	4.70E-12	3.08E-10	2084.90	-0.52	0.39	-1.32	0.188	0.882
ENST00000503126	CANX	192.33	-1.65	0.59	-2.77	5.62E-03	9.18E-02	69.94	0.23	0.72	0.32	0.752	1.000
ENST00000479759	CAP1	151.81	-3.10	1.12	-2.78	5.51E-03	9.05E-02	254.26	0.26	0.39	0.67	0.504	1.000
ENST00000366869	CAPN2	33.92	-21.35	3.03	-7.05	1.77E-12	1.51E-10	381.94	1.72	0.92	1.87	0.062	0.581
ENST00000327064	CARM1	32.74	-1.90	0.73	-2.60	9.43E-03	1.32E-01	5127.35	0.06	0.21	0.26	0.794	1.000
ENST00000529772	CARS	12.00	-21.59	3.03	-7.13	1.02E-12	9.73E-11	1652.28	-0.50	0.56	-0.89	0.372	1.000
ENST00000524843	CASP4	5.26	-5.53	1.77	-3.13	1.74E-03	3.71E-02	53.02	-0.23	0.67	-0.34	0.736	1.000
ENST00000412916	CBFB	73.27	-9.33	1.00	-9.34	9.28E-21	1.95E-17	8467.26	0.06	0.16	0.38	0.706	1.000
ENST00000493361	CBLL1	13.16	-3.55	1.27	-2.78	5.38E-03	8.91E-02	71.41	0.06	0.51	0.11	0.909	1.000
ENST00000314367	CBWD1	2.10	-4.19	1.17	-3.59	3.34E-04	9.46E-03	193.15	0.74	0.39	1.90	0.058	0.565
ENST00000462165	CBX3	25.73	-7.82	3.01	-2.60	9.33E-03	1.31E-01	1225.01	0.01	0.27	0.03	0.977	1.000
ENST00000402326	CBX3P9	332.83	-0.91	0.22	-4.13	3.70E-05	1.36E-03	30.12	0.07	0.50	0.14	0.890	1.000
ENST00000492537	CBY1	3.91	-5.10	1.74	-2.92	3.46E-03	6.41E-02	107.35	-0.06	0.32	-0.20	0.842	1.000
ENST00000480627	CCAR1	12.70	-4.82	1.77	-2.73	6.41E-03	1.01E-01	431.81	-0.10	0.26	-0.38	0.701	1.000
ENST00000543225	CCAR1	27.93	-22.73	3.03	-7.51	5.94E-14	1.21E-11	3367.86	0.20	0.14	1.46	0.144	0.817
ENST00000446900	CCBL2	14.05	-21.80	3.03	-7.20	6.06E-13	6.91E-11	41.47	1.47	0.63	2.34	0.019	0.310
ENST00000339012	CCDC104	16.52	-5.37	1.05	-5.12	3.02E-07	1.41E-05	160.17	0.69	0.90	0.77	0.444	1.000
ENST00000249064	CCDC117	30.62	-1.91	0.48	-4.02	5.78E-05	2.03E-03	3972.14	-0.25	0.18	-1.42	0.156	0.839
ENST00000295124	CCDC138	22.69	-3.50	0.57	-6.14	8.01E-10	4.21E-08	1271.47	-0.23	0.32	-0.73	0.467	1.000
ENST00000487498	CCDC14	2.92	-4.05	1.46	-2.77	5.55E-03	9.10E-02	67.71	-0.26	0.62	-0.42	0.673	1.000
ENST00000504487	CCDC149	6.27	-2.59	0.76	-3.41	6.42E-04	1.65E-02	1788.07	-0.32	0.18	-1.77	0.077	0.646
ENST00000328697	CCDC34	8.06	-6.15	1.73	-3.54	3.94E-04	1.09E-02	775.59	-0.33	0.19	-1.75	0.080	0.656
ENST00000291458	CCDC58	17.27	-7.25	1.34	-5.39	7.01E-08	3.45E-06	246.74	-0.26	0.44	-0.60	0.551	1.000
ENST00000263102	CCDC6	133.92	-0.87	0.29	-2.98	2.87E-03	5.55E-02	6881.20	-0.17	0.13	-1.33	0.185	0.879
ENST00000206423	CCDC80	24.45	-1.29	0.40	-3.21	1.33E-03	2.99E-02	1849.77	-0.06	0.19	-0.29	0.772	1.000
ENST00000530106	CCDC82	24.48	-1.04	0.37	-2.80	5.06E-03	8.52E-02	461.43	0.32	0.25	1.28	0.199	0.894
ENST00000545580	CCDC86	33.80	-22.98	3.03	-7.59	3.12E-14	7.67E-12	21.51	-1.71	0.80	-2.14	0.032	0.416
ENST00000507232	CCDC99	14.71	-21.86	3.03	-7.22	5.24E-13	6.20E-11	522.05	0.95	0.98	0.97	0.332	0.987
ENST00000396268	CCHCR1	7.97	-6.12	1.16	-5.29	1.25E-07	6.08E-06	32.68	0.89	1.48	0.60	0.548	1.000
ENST00000559622	CCNB2	6.31	-20.72	3.03	-6.84	8.10E-12	4.88E-10	21.79	-1.25	1.38	-0.91	0.362	0.999
ENST00000536559	CCND1	4.84	-5.41	1.85	-2.93	3.41E-03	6.35E-02	103.19	0.01	0.49	0.03	0.979	1.000
ENST00000262643	CCNE1	6.50	-4.62	1.75	-2.64	8.31E-03	1.21E-01	1639.19	0.05	0.21	0.22	0.823	1.000
ENST00000256897	CCNH	30.88	-8.08	2.23	-3.63	2.87E-04	8.37E-03	684.21	0.01	0.23	0.05	0.957	1.000
ENST00000504115	CCNH	29.15	-8.00	2.71	-2.95	3.20E-03	6.03E-02	36.10	0.25	0.49	0.50	0.615	1.000
ENST00000505230	CCNH	5.78	-5.67	1.73	-3.27	1.07E-03	2.52E-02	214.95	-0.17	0.33	-0.52	0.603	1.000
ENST00000511207	CCNH	13.60	-6.90	2.62	-2.63	8.43E-03	1.22E-01	7.15	-1.22	1.09	-1.11	0.265	0.953
ENST00000310958	CCPG1	7.84	-2.25	0.74	-3.03	2.45E-03	4.91E-02	3608.61	0.29	0.30	0.98	0.325	0.985
ENST00000550010	CCT2	15.82	-21.93	3.03	-7.24	4.43E-13	5.40E-11	6554.80	-0.04	0.17	-0.23	0.819	1.000
ENST00000553169	CCT2	107.69	-9.89	0.97	-10.20	1.93E-24	6.73E-21	1733.34	-0.08	0.16	-0.53	0.599	1.000
ENST00000368259	CCT3	274.45	-25.84	3.03	-8.54	1.37E-17	1.65E-14	1803.00	0.58	1.32	0.44	0.660	1.000
ENST00000538252	CCT4	86.46	-2.49	0.87	-2.85	4.41E-03	7.68E-02	9322.84	-0.04	0.19	-0.23	0.822	1.000
ENST00000503026	CCT5	235.68	-25.64	3.03	-8.47	2.44E-17	2.62E-14	221.92	0.04	0.55	0.07	0.947	1.000
ENST00000515676	CCT5	407.23	-26.37	3.03	-8.71	2.91E-18	4.21E-15	16309.90	-0.20	0.78	-0.26	0.798	1.000
ENST00000482776	CCT6A	37.84	-7.22	1.76	-4.10	4.15E-05	1.50E-03	880.25	-0.09	0.32	-0.29	0.774	1.000
ENST00000258091	CCT7	403.78	-26.36	3.03	-8.71	3.06E-18	4.28E-15	3637.06	3.26	0.97	3.36	0.001	0.030

ENST00000398422	CCT7	33.25	-22.85	3.03	-7.55	4.41E-14	9.69E-12	303.05	-1.21	1.43	-0.84	0.399	1.000
ENST00000464397	CCT7	3.46	-4.93	1.37	-3.59	3.25E-04	9.27E-03	34.99	-0.75	0.80	-0.93	0.351	0.994
ENST00000469844	CCT7	50.32	-23.52	3.03	-7.77	7.70E-15	2.60E-12	1253.13	-1.15	1.27	-0.91	0.365	0.999
ENST00000480489	CCT7	12.29	-6.76	2.58	-2.62	8.89E-03	1.27E-01	8.54	-0.04	1.49	-0.03	0.978	1.000
ENST00000537131	CCT7	48.52	-22.54	3.03	-7.45	9.46E-14	1.64E-11	12480.86	0.11	0.62	0.17	0.861	1.000
ENST00000287097	CD109	292.53	-25.93	3.03	-8.57	1.06E-17	1.39E-14	38883.15	0.23	0.69	0.34	0.736	1.000
ENST00000530320	CD151	27.60	-22.71	3.03	-7.50	6.24E-14	1.25E-11	2866.33	0.18	0.19	0.93	0.353	0.995
ENST00000085219	CD22	4.93	-2.51	0.91	-2.76	5.83E-03	9.46E-02	1677.50	2.07	1.08	1.91	0.056	0.557
ENST00000359314	CD2AP	102.23	-0.78	0.27	-2.83	4.62E-03	7.93E-02	4055.68	-0.09	0.16	-0.56	0.573	1.000
ENST00000279452	CD44	132.26	-24.80	3.03	-8.19	2.54E-16	1.97E-13	196.75	0.13	0.25	0.53	0.595	1.000
ENST00000442151	CD44	22.37	-22.42	3.03	-7.41	1.29E-13	2.05E-11	3098.64	-0.08	0.39	-0.22	0.829	1.000
ENST00000527326	CD44	65.37	-2.68	0.88	-3.03	2.45E-03	4.90E-02	1554.33	0.12	0.35	0.34	0.731	1.000
ENST00000527926	CD59	205.30	-3.29	1.05	-3.12	1.78E-03	3.78E-02	858.41	0.62	0.51	1.20	0.231	0.927
ENST00000533403	CD59	28.14	-7.95	1.00	-7.93	2.16E-15	1.05E-12	134.02	0.10	0.36	0.28	0.777	1.000
ENST00000549117	CD63	51.72	-2.84	1.07	-2.66	7.88E-03	1.17E-01	3141.13	0.77	0.43	1.80	0.071	0.623
ENST00000550050	CD63	104.70	-24.36	3.03	-8.05	8.36E-16	5.17E-13	1495.51	-1.94	4.56	-0.43	0.671	1.000
ENST00000523208	CD74	31.53	-8.11	2.00	-4.06	5.00E-05	1.78E-03	580.42	0.40	0.46	0.88	0.379	1.000
ENST00000523813	CD74	32.23	-4.01	1.36	-2.96	3.09E-03	5.87E-02	166.26	0.86	0.32	2.72	0.007	0.153
ENST00000524315	CD74	12.18	-21.61	3.03	-7.13	9.70E-13	9.37E-11	604.33	0.48	0.27	1.78	0.076	0.640
ENST00000540891	CD9	24.50	-7.75	2.70	-2.87	4.07E-03	7.22E-02	30.21	0.41	0.52	0.78	0.438	1.000
ENST00000438817	CD96	10.77	-21.44	3.03	-7.08	1.46E-12	1.30E-10	312.61	-1.06	0.41	-2.56	0.010	0.211
ENST00000360383	CDC16	51.32	-1.66	0.56	-2.96	3.10E-03	5.89E-02	33.70	4.88	1.92	2.54	0.011	0.218
ENST00000439880	CDC25B	69.80	-23.98	3.03	-7.92	2.33E-15	1.10E-12	16714.59	-0.32	0.13	-2.50	0.013	0.237
ENST00000495915	CDC25B	2.91	-4.67	1.26	-3.72	2.00E-04	6.10E-03	65.60	-0.46	0.60	-0.77	0.442	1.000
ENST00000527547	CDC27	11.28	-21.51	3.03	-7.10	1.24E-12	1.15E-10	7735.46	0.18	0.29	0.62	0.533	1.000
ENST00000421089	CDC42	19.46	-22.25	3.03	-7.35	1.98E-13	2.90E-11	349.26	0.92	1.61	0.57	0.567	1.000
ENST00000470278	CDC42SE1	20.45	-22.31	3.03	-7.37	1.72E-13	2.58E-11	127.64	0.30	0.38	0.78	0.434	1.000
ENST00000371477	CDC5L	336.21	-0.74	0.23	-3.20	1.37E-03	3.08E-02	7368.87	-0.11	0.15	-0.75	0.455	1.000
ENST00000430031	CDC7	5.99	-20.65	3.03	-6.81	9.58E-12	5.60E-10	88.69	0.06	0.42	0.15	0.881	1.000
ENST00000523454	CDCA2	4.10	-5.16	1.25	-4.13	3.71E-05	1.36E-03	62.01	1.17	1.61	0.73	0.468	1.000
ENST00000479032	CDCA5	25.96	-5.35	1.46	-3.66	2.54E-04	7.52E-03	90.50	-0.13	0.32	-0.41	0.678	1.000
ENST00000431540	CDH13	10.96	-5.39	1.19	-4.52	6.13E-06	2.55E-04	157.16	-0.72	0.56	-1.29	0.197	0.893
ENST00000539548	CDH13	15.78	-21.94	3.03	-7.25	4.27E-13	5.27E-11	829.89	1.04	3.51	0.30	0.768	1.000
ENST00000565636	CDH13	3.12	-3.51	1.17	-3.00	2.74E-03	5.35E-02	90.26	-0.06	0.87	-0.06	0.948	1.000
ENST00000413878	CDH2	9.56	-2.90	0.97	-2.99	2.79E-03	5.42E-02	223.94	0.29	0.23	1.30	0.194	0.890
ENST00000563415	CDIPT	1.95	-3.47	1.22	-2.85	4.37E-03	7.64E-02	101.41	-0.20	0.30	-0.67	0.504	1.000
ENST00000276052	CDK16	5.77	-5.66	1.83	-3.09	2.02E-03	4.21E-02	467.43	-0.70	0.82	-0.86	0.391	1.000
ENST00000522234	CDK16	5.93	-20.64	3.03	-6.81	9.82E-12	5.71E-10	115.70	0.33	0.55	0.60	0.549	1.000
ENST00000261211	CDK17	57.39	-0.86	0.32	-2.72	6.50E-03	1.02E-01	2079.30	-0.02	0.27	-0.08	0.935	1.000
ENST00000312990	CDK4	47.45	-8.70	1.10	-7.93	2.12E-15	1.05E-12	2956.97	-0.13	0.17	-0.75	0.455	1.000
ENST00000552388	CDK4	6.73	-20.80	3.03	-6.86	6.68E-12	4.14E-10	2518.31	-0.06	0.87	-0.07	0.943	1.000
ENST00000373264	CDK9	9.02	-6.31	2.07	-3.05	2.29E-03	4.65E-02	292.95	-0.29	0.29	-0.99	0.322	0.984
ENST00000491521	CDK9	7.43	-6.03	1.77	-3.40	6.74E-04	1.72E-02	84.23	-0.09	0.59	-0.15	0.882	1.000
ENST00000503932	CDV3	8.20	-6.17	2.14	-2.88	3.92E-03	7.03E-02	55.39	2.15	0.90	2.37	0.018	0.294
ENST00000535982	CELF1	3.42	-4.90	1.08	-4.52	6.18E-06	2.57E-04	35.33	-0.33	0.55	-0.61	0.544	1.000
ENST00000510742	CENPH	18.43	-7.34	2.64	-2.78	5.45E-03	8.99E-02	55.65	-1.00	0.84	-1.20	0.229	0.925
ENST00000510768	CENPK	7.60	-6.06	1.94	-3.13	1.77E-03	3.76E-02	51.60	-0.68	0.43	-1.58	0.115	0.761
ENST00000562943	CENPN	4.74	-4.76	1.61	-2.96	3.10E-03	5.89E-02	33.84	-1.22	0.65	-1.86	0.063	0.588
ENST00000306467	CEP120	30.82	-1.75	0.63	-2.77	5.59E-03	9.15E-02	821.01	0.00	0.29	0.00	0.997	1.000
ENST00000445435	CEP55	3.15	-2.47	0.92	-2.69	7.05E-03	1.08E-01	22.58	0.28	1.68	0.17	0.867	1.000
ENST00000536374	CEP78	22.64	-7.64	1.88	-4.06	4.85E-05	1.74E-03	709.66	-0.09	0.23	-0.39	0.696	1.000
ENST00000422678	CFL2	8.47	-2.52	0.85	-2.97	2.99E-03	5.72E-02	1797.29	0.84	1.11	0.76	0.449	1.000
ENST00000341222	CFLAR	8.90	-4.01	1.25	-3.22	1.28E-03	2.90E-02	221.51	-0.61	1.13	-0.54	0.590	1.000
ENST00000314103	CHAF1B	37.33	-1.11	0.31	-3.55	3.82E-04	1.06E-02	1132.74	-0.19	0.15	-1.25	0.211	0.907
ENST00000512392	CHD1	11.52	-6.66	2.58	-2.58	9.90E-03	1.36E-01	20.29	-0.73	0.90	-0.81	0.417	1.000
ENST00000430771	CHD4	2.96	-4.06	1.55	-2.63	8.63E-03	1.24E-01	58.76	0.31	0.41	0.76	0.450	1.000
ENST00000429789	CHID1	5.78	-20.60	3.03	-6.80	1.06E-11	6.11E-10	784.63	-0.06	0.41	-0.15	0.881	1.000
ENST00000531859	CHID1	15.82	-7.12	1.91	-3.72	1.99E-04	6.07E-03	23.04	0.41	0.70	0.58	0.561	1.000

ENST00000256509	CHL1	31.05	-3.57	0.93	-3.82	1.33E-04	4.23E-03	1464.96	0.08	0.19	0.42	0.678	1.000
ENST00000263780	CHMP2B	117.93	-1.26	0.44	-2.85	4.33E-03	7.58E-02	3998.32	0.09	0.13	0.68	0.495	1.000
ENST00000295497	CHN1	81.86	-7.75	1.30	-5.96	2.45E-09	1.28E-07	6850.25	1.77	0.28	6.42	0.000	0.000
ENST00000529726	CHORDC1	7.43	-2.92	1.11	-2.64	8.32E-03	1.21E-01	481.01	-0.23	0.37	-0.61	0.539	1.000
ENST00000368476	CHRNB2	1.24	-3.42	1.31	-2.62	8.80E-03	1.26E-01	415.43	-0.38	0.22	-1.74	0.082	0.661
ENST00000565786	CIAPIN1	17.39	-7.26	2.64	-2.75	6.01E-03	9.67E-02	88.58	0.15	0.48	0.32	0.749	1.000
ENST00000342016	CIR1	11.41	-1.39	0.47	-2.93	3.37E-03	6.28E-02	1659.50	-0.23	1.22	-0.19	0.852	1.000
ENST00000564408	CIRH1A	3.90	-5.10	1.81	-2.81	4.97E-03	8.40E-02	218.37	0.13	0.33	0.38	0.705	1.000
ENST00000273986	CISD2	16.82	-1.06	0.38	-2.77	5.55E-03	9.10E-02	2546.63	-0.05	0.13	-0.36	0.720	1.000
ENST00000533413	CKAP5	28.39	-21.94	3.03	-7.25	4.22E-13	5.24E-11	376.08	-0.86	1.27	-0.68	0.500	1.000
ENST00000553878	CKB	7.35	-3.86	1.07	-3.59	3.25E-04	9.27E-03	175.78	1.46	1.42	1.03	0.305	0.977
ENST00000525064	CLNS1A	15.52	-21.94	3.03	-7.24	4.34E-13	5.32E-11	1212.93	0.26	0.15	1.80	0.072	0.627
ENST00000527299	CLNS1A	39.33	-3.33	0.52	-6.44	1.17E-10	6.32E-09	146.98	0.10	0.28	0.37	0.710	1.000
ENST00000517467	CLSPN	5.42	-4.34	1.32	-3.30	9.70E-04	2.32E-02	10.81	-0.28	0.98	-0.29	0.773	1.000
ENST00000477264	CLSTN1	147.04	-10.33	2.11	-4.90	9.51E-07	4.28E-05	247.49	0.16	0.51	0.32	0.751	1.000
ENST00000393043	CLTC	282.68	-8.30	2.94	-2.83	4.71E-03	8.06E-02	4257.81	0.40	0.19	2.10	0.036	0.444
ENST00000472129	CLTC	48.29	-8.73	2.79	-3.12	1.79E-03	3.79E-02	140.86	-0.47	0.64	-0.73	0.466	1.000
ENST00000538498	CMAS	7.35	-20.93	3.03	-6.91	4.96E-12	3.22E-10	51.18	-0.57	0.96	-0.60	0.551	1.000
ENST00000451728	CNBP	311.64	-1.44	0.31	-4.66	3.16E-06	1.36E-04	1978.82	-0.04	0.21	-0.20	0.844	1.000
ENST00000324301	CNDP2	15.24	-7.06	2.65	-2.66	7.77E-03	1.16E-01	376.35	-0.40	0.19	-2.07	0.039	0.458
ENST00000562075	CNN2	8.51	-21.12	3.03	-6.97	3.10E-12	2.28E-10	194.53	-0.36	0.23	-1.57	0.117	0.766
ENST00000328834	CNOT10	41.37	-1.61	0.33	-4.85	1.25E-06	5.56E-05	1024.53	-0.71	0.36	-1.98	0.048	0.516
ENST00000550194	CNOT2	3.65	-5.00	1.94	-2.58	9.80E-03	1.35E-01	14.34	-0.22	0.77	-0.29	0.775	1.000
ENST00000504123	CNOT6L	1.40	-3.64	1.28	-2.84	4.55E-03	7.85E-02	178.93	-0.87	4.56	-0.19	0.849	1.000
ENST00000403027	CNOT8	41.24	-3.61	1.35	-2.67	7.49E-03	1.13E-01	1261.90	-3.98	1.21	-3.29	0.001	0.037
ENST00000546937	CNPY2	118.54	-10.02	1.04	-9.68	3.56E-22	8.30E-19	1570.18	1.00	1.07	0.93	0.351	0.994
ENST00000551475	CNPY2	46.95	-8.69	2.78	-3.13	1.76E-03	3.76E-02	265.25	-0.57	0.27	-2.11	0.035	0.436
ENST00000366669	COG2	25.86	-7.83	1.81	-4.31	1.61E-05	6.36E-04	335.08	0.08	1.01	0.08	0.938	1.000
ENST00000567754	COG4	6.48	-4.60	1.39	-3.30	9.73E-04	2.32E-02	26.07	-0.17	0.55	-0.32	0.751	1.000
ENST00000347053	COG5	36.40	-23.09	3.03	-7.63	2.38E-14	6.24E-12	4694.23	0.14	0.18	0.81	0.420	1.000
ENST00000562081	COG8	8.17	-6.17	2.22	-2.78	5.45E-03	8.99E-02	1102.05	-1.97	0.92	-2.14	0.032	0.415
ENST00000512756	COL11A1	13.60	-21.75	3.03	-7.18	6.76E-13	7.31E-11	16013.60	0.96	0.84	1.14	0.255	0.947
ENST00000322507	COL12A1	291.91	-0.99	0.28	-3.59	3.37E-04	9.54E-03	20696.23	-1.03	0.73	-1.41	0.158	0.840
ENST00000375001	COL15A1	13.98	-4.83	0.91	-5.30	1.15E-07	5.60E-06	14409.85	-0.22	0.13	-1.67	0.096	0.714
ENST00000261415	COL4A3BP	119.27	-23.77	3.03	-7.85	4.00E-15	1.70E-12	1500.15	0.28	0.15	1.84	0.066	0.603
ENST00000357250	COL9A1	4.87	-3.06	1.11	-2.74	6.14E-03	9.82E-02	7830.80	-0.45	0.56	-0.79	0.429	1.000
ENST00000368069	COPA	180.95	-6.10	1.46	-4.19	2.77E-05	1.05E-03	27156.08	0.15	0.40	0.38	0.704	1.000
ENST00000349893	COPE	126.46	-10.12	1.99	-5.08	3.74E-07	1.74E-05	91.61	0.16	0.47	0.33	0.738	1.000
ENST00000538245	COPE	12.28	-21.62	3.03	-7.14	9.40E-13	9.17E-11	9531.57	0.00	0.18	-0.02	0.987	1.000
ENST00000445977	COPG2	47.13	-1.18	0.24	-4.82	1.47E-06	6.49E-05	30.71	-0.03	0.74	-0.04	0.970	1.000
ENST00000542928	COPS2	27.04	-4.41	1.61	-2.73	6.35E-03	1.01E-01	108.60	-0.08	0.41	-0.19	0.848	1.000
ENST00000558545	COPS2	36.73	-8.33	2.25	-3.71	2.06E-04	6.25E-03	23.53	0.07	0.84	0.08	0.938	1.000
ENST00000558843	COPS2	25.84	-7.83	2.68	-2.92	3.50E-03	6.48E-02	19.95	-0.34	0.61	-0.56	0.575	1.000
ENST00000511653	COPS4	6.82	-5.30	1.10	-4.82	1.41E-06	6.26E-05	849.41	0.11	0.26	0.42	0.675	1.000
ENST00000409629	COPS8	4.68	-5.35	1.39	-3.84	1.25E-04	4.01E-03	150.53	0.45	0.49	0.91	0.362	0.999
ENST00000341356	CORO1B	34.86	-23.03	3.03	-7.61	2.79E-14	7.06E-12	306.64	-0.45	0.83	-0.54	0.590	1.000
ENST00000562929	COX4I1	5.41	-20.49	3.03	-6.76	1.38E-11	7.68E-10	159.85	-0.28	0.31	-0.89	0.372	1.000
ENST00000566405	COX4I1	17.15	-5.57	1.92	-2.91	3.66E-03	6.69E-02	3239.45	-0.29	0.17	-1.75	0.081	0.659
ENST00000297564	COX6C	42.18	-0.87	0.32	-2.72	6.55E-03	1.02E-01	2163.51	0.38	0.17	2.28	0.023	0.344
ENST00000520468	COX6C	119.91	-10.04	1.10	-9.12	7.58E-20	1.51E-16	1093.97	0.25	1.32	0.19	0.849	1.000
ENST00000524245	COX6C	49.44	-1.78	0.61	-2.90	3.75E-03	6.82E-02	460.77	0.06	0.30	0.20	0.845	1.000
ENST00000460985	COX7A2	6.34	-5.80	1.74	-3.33	8.53E-04	2.09E-02	117.37	-0.17	0.39	-0.45	0.653	1.000
ENST00000234301	COX7A2L	28.71	-7.98	0.96	-8.31	9.36E-17	8.35E-14	3790.20	0.44	0.41	1.07	0.283	0.962
ENST00000247655	COX7C	66.15	-8.02	1.01	-7.96	1.72E-15	9.13E-13	6928.39	0.11	0.19	0.56	0.578	1.000
ENST00000402744	CPE	179.99	-1.36	0.43	-3.13	1.72E-03	3.68E-02	9470.46	-0.14	0.13	-1.09	0.277	0.960
ENST00000352393	CPNE1	31.10	-6.92	2.39	-2.89	3.84E-03	6.95E-02	65.11	1.41	1.02	1.38	0.169	0.854
ENST00000198765	CPNE3	33.37	-2.94	1.06	-2.78	5.46E-03	8.99E-02	11.29	-0.67	1.75	-0.39	0.700	1.000
ENST00000489629	CPSF3	19.11	-7.39	1.12	-6.58	4.70E-11	2.56E-09	491.33	1.19	0.62	1.93	0.054	0.546

ENST00000539743	CPT1A	9.89	-21.32	3.03	-7.04	1.92E-12	1.60E-10	28.05	0.49	0.77	0.63	0.529	1.000
ENST00000498700	CRBN	4.95	-5.44	2.03	-2.68	7.42E-03	1.12E-01	423.89	0.15	0.22	0.70	0.484	1.000
ENST00000344351	CREM	3.50	-4.94	1.74	-2.84	4.46E-03	7.74E-02	56.13	1.02	0.61	1.66	0.097	0.715
ENST00000329146	CRIP2	6.79	-5.30	1.45	-3.66	2.56E-04	7.56E-03	1044.88	0.35	0.24	1.46	0.143	0.817
ENST00000550577	CRIP2	10.70	-2.76	0.90	-3.06	2.19E-03	4.48E-02	29.52	2.29	2.13	1.08	0.282	0.962
ENST00000182096	CRYBG3	9.26	-1.46	0.55	-2.67	7.56E-03	1.14E-01	3634.43	0.00	0.19	0.01	0.992	1.000
ENST00000492102	CRYZ	4.81	-4.79	1.66	-2.89	3.86E-03	6.97E-02	214.39	0.14	0.42	0.33	0.744	1.000
ENST00000469700	CSE1L	29.58	-4.18	0.95	-4.39	1.11E-05	4.46E-04	1389.47	-0.22	0.14	-1.54	0.123	0.780
ENST00000344188	CSF1	8.85	-21.17	3.03	-6.99	2.75E-12	2.08E-10	41.06	-0.97	2.36	-0.41	0.682	1.000
ENST00000567135	CSK	19.25	-22.22	3.03	-7.34	2.14E-13	3.07E-11	856.50	-0.05	0.67	-0.08	0.940	1.000
ENST00000269361	CSNK1D	11.20	-21.49	3.03	-7.10	1.27E-12	1.18E-10	1247.11	-0.14	0.15	-0.96	0.338	0.989
ENST00000405675	CSNK1E	6.89	-3.84	1.48	-2.59	9.52E-03	1.32E-01	2277.06	0.33	0.17	1.97	0.048	0.518
ENST00000562367	CSNK2A2	9.19	-2.70	0.97	-2.80	5.15E-03	8.65E-02	52.60	0.00	0.62	0.00	0.999	1.000
ENST00000546966	CSRP2	44.65	-23.37	3.03	-7.72	1.15E-14	3.61E-12	215.28	0.31	0.24	1.29	0.197	0.893
ENST00000398411	CST3	41.30	-8.50	0.97	-8.72	2.67E-18	4.00E-15	1139.43	-0.22	0.15	-1.49	0.137	0.805
ENST00000490539	CSTF1	10.35	-5.31	1.97	-2.70	6.90E-03	1.06E-01	814.59	0.79	1.27	0.63	0.532	1.000
ENST00000478778	CTB-118N6.1.1	48.27	-1.63	0.46	-3.56	3.77E-04	1.05E-02	7.36	0.04	1.21	0.03	0.975	1.000
ENST00000477670	CTD-2284O10.1.1	200.81	-1.38	0.53	-2.60	9.26E-03	1.30E-01	30.72	-0.80	0.73	-1.10	0.272	0.958
ENST00000515712	CTD-2309M13.1.1	201.75	-1.81	0.64	-2.83	4.65E-03	7.96E-02	8.90	0.43	1.02	0.42	0.676	1.000
ENST00000534518	CTD-2589O24.1.1	123.23	-0.95	0.31	-3.02	2.56E-03	5.08E-02	21.05	-0.02	0.66	-0.03	0.977	1.000
ENST00000260327	CTDSPL2	54.36	-2.18	0.34	-6.43	1.29E-10	6.94E-09	4662.24	-0.18	0.24	-0.77	0.444	1.000
ENST00000396185	CTNNB1	511.85	-2.82	0.82	-3.42	6.30E-04	1.63E-02	1058.22	-0.32	0.60	-0.54	0.587	1.000
ENST00000361383	CTNNBL1	88.69	-9.61	2.83	-3.40	6.79E-04	1.73E-02	8013.28	0.12	0.15	0.79	0.430	1.000
ENST00000372616	CTPS	13.85	-21.78	3.03	-7.19	6.35E-13	7.03E-11	106.21	-0.94	1.57	-0.60	0.548	1.000
ENST00000372621	CTPS	342.60	-1.13	0.36	-3.11	1.84E-03	3.89E-02	12688.23	-0.06	0.13	-0.48	0.632	1.000
ENST00000372459	CTSA	8.38	-5.60	1.78	-3.15	1.61E-03	3.48E-02	1638.08	0.32	0.70	0.46	0.647	1.000
ENST00000367196	CTSD	8.89	-21.18	3.03	-6.99	2.73E-12	2.07E-10	201.94	0.46	0.39	1.17	0.242	0.935
ENST00000310325	CTSF	99.77	-1.73	0.64	-2.68	7.38E-03	1.12E-01	15972.78	0.09	0.21	0.41	0.679	1.000
ENST00000374748	CUL2	81.10	-1.18	0.36	-3.25	1.17E-03	2.69E-02	1840.37	-0.81	0.30	-2.68	0.007	0.167
ENST00000374754	CUL2	17.21	-7.24	1.64	-4.42	9.82E-06	3.98E-04	434.16	3.48	2.47	1.41	0.158	0.840
ENST00000264414	CUL3	202.65	-0.61	0.17	-3.49	4.89E-04	1.31E-02	1995.33	0.01	0.43	0.02	0.985	1.000
ENST00000375441	CUL4A	14.02	-20.67	3.03	-6.83	8.79E-12	5.21E-10	1586.70	-0.77	0.17	-4.52	0.000	0.000
ENST00000393094	CUL5	22.76	-1.03	0.29	-3.52	4.30E-04	1.18E-02	1772.44	0.02	0.17	0.13	0.893	1.000
ENST00000524608	CYB5R2	45.23	-3.65	1.05	-3.47	5.21E-04	1.39E-02	1214.92	0.11	0.14	0.73	0.464	1.000
ENST00000533444	CYC1	23.90	-3.39	1.04	-3.25	1.14E-03	2.64E-02	331.15	0.13	0.55	0.23	0.818	1.000
ENST00000558826	CYFIP1	22.29	-7.61	1.90	-4.00	6.38E-05	2.22E-03	56.71	-1.35	1.00	-1.34	0.179	0.870
ENST00000561020	CYFIP1	18.66	-6.77	1.83	-3.69	2.23E-04	6.71E-03	2563.28	2.15	0.83	2.60	0.009	0.198
ENST00000356079	CYP20A1	13.69	-6.32	1.62	-3.91	9.13E-05	3.06E-03	241.44	0.21	0.51	0.42	0.674	1.000
ENST00000360431	CYR61	68.77	-23.95	3.03	-7.91	2.48E-15	1.14E-12	19121.51	0.19	0.21	0.91	0.363	0.999
ENST00000479935	DAG1	23.79	-22.51	3.03	-7.44	1.03E-13	1.75E-11	119.78	0.22	0.48	0.46	0.644	1.000
ENST00000471523	DAP3	5.68	-5.64	1.74	-3.24	1.22E-03	2.78E-02	811.63	0.24	0.21	1.15	0.252	0.943
ENST00000535183	DAP3	21.80	-6.42	2.04	-3.15	1.64E-03	3.55E-02	405.49	-0.53	0.37	-1.43	0.153	0.832
ENST00000264161	DARS	481.56	-0.70	0.27	-2.61	9.16E-03	1.29E-01	3381.60	1.13	0.88	1.29	0.199	0.894
ENST00000361951	DARS2	135.07	-0.69	0.23	-3.03	2.48E-03	4.97E-02	1437.18	-0.01	0.16	-0.09	0.930	1.000
ENST00000311521	DBI	30.55	-8.03	3.03	-2.65	7.97E-03	1.18E-01	55.95	0.45	0.57	0.79	0.428	1.000
ENST00000409094	DBI	50.85	-7.65	1.41	-5.42	6.11E-08	3.01E-06	1390.36	0.10	0.16	0.65	0.513	1.000
ENST00000460901	DBI	41.96	-6.31	2.00	-3.16	1.56E-03	3.40E-02	525.05	-0.14	0.39	-0.35	0.727	1.000
ENST00000492375	DBI	10.48	-2.94	0.93	-3.17	1.51E-03	3.32E-02	1138.83	0.00	0.47	0.00	1.000	1.000
ENST00000535757	DBI	24.65	-2.61	1.01	-2.60	9.44E-03	1.32E-01	96.97	-0.74	0.55	-1.36	0.175	0.864
ENST00000370132	DBT	61.45	-1.70	0.47	-3.63	2.85E-04	8.30E-03	2485.40	0.10	0.13	0.77	0.439	1.000
ENST00000389063	DCP2	38.76	-1.58	0.44	-3.55	3.80E-04	1.06E-02	3874.16	1.29	0.76	1.68	0.092	0.701
ENST00000491465	DCTN1	18.70	-7.36	1.86	-3.95	7.91E-05	2.69E-03	237.29	-0.73	0.92	-0.79	0.427	1.000
ENST00000537120	DDB1	22.77	-21.85	3.03	-7.22	5.36E-13	6.28E-11	76.46	0.00	0.55	-0.01	0.993	1.000
ENST00000415136	DDOST	23.97	-22.52	3.03	-7.44	1.01E-13	1.72E-11	3561.85	0.70	0.92	0.76	0.448	1.000
ENST00000233084	DDX1	458.01	-11.97	2.11	-5.67	1.40E-08	7.09E-07	16750.11	-0.02	0.12	-0.20	0.838	1.000
ENST00000471144	DDX27	9.20	-4.56	1.47	-3.11	1.85E-03	3.91E-02	1115.92	1.51	0.27	5.53	0.000	0.000
ENST00000396172	DDX39B	7.85	-21.01	3.03	-6.94	4.04E-12	2.76E-10	4.09	2.12	1.42	1.49	0.136	0.803
ENST00000431908	DDX39B	64.80	-23.66	3.03	-7.82	5.44E-15	1.98E-12	1733.48	-0.16	0.29	-0.56	0.575	1.000

ENST00000453105	DDX39B	67.93	-6.08	1.35	-4.51	6.40E-06	2.66E-04	5339.07	-0.15	0.14	-1.07	0.286	0.963
ENST00000441189	DDX3X	46.00	-8.66	2.83	-3.06	2.23E-03	4.54E-02	185.12	-0.15	0.33	-0.45	0.650	1.000
ENST00000389924	DDX42	206.83	-0.55	0.17	-3.19	1.44E-03	3.20E-02	578.05	0.19	0.24	0.81	0.421	1.000
ENST00000373585	DDX50	74.25	-1.42	0.40	-3.53	4.19E-04	1.15E-02	5959.42	-0.28	0.22	-1.29	0.197	0.893
ENST00000460470	DDX50	5.89	-20.62	3.03	-6.81	1.01E-11	5.86E-10	510.03	0.68	0.88	0.78	0.437	1.000
ENST00000460080	DDX52	14.90	-5.84	1.82	-3.21	1.31E-03	2.95E-02	197.82	0.77	0.64	1.19	0.233	0.928
ENST00000244776	DEK	19.48	-22.21	3.03	-7.34	2.21E-13	3.13E-11	70.40	0.16	0.44	0.37	0.709	1.000
ENST00000512145	DEK	11.29	-6.63	1.91	-3.46	5.34E-04	1.42E-02	49.36	-1.20	0.46	-2.62	0.009	0.187
ENST00000280557	DENR	43.17	-1.18	0.39	-3.04	2.40E-03	4.84E-02	6118.33	-0.03	0.13	-0.21	0.831	1.000
ENST00000527316	DHCR7	11.55	-21.54	3.03	-7.11	1.15E-12	1.08E-10	84.52	-0.05	0.54	-0.09	0.925	1.000
ENST00000533800	DHCR7	31.12	-22.85	3.03	-7.55	4.37E-14	9.69E-12	38.54	-0.42	0.55	-0.75	0.454	1.000
ENST00000536410	DHRS7	18.56	-22.17	3.03	-7.32	2.43E-13	3.40E-11	878.62	0.54	1.13	0.48	0.630	1.000
ENST00000252011	DHX35	50.72	-1.57	0.43	-3.68	2.36E-04	7.04E-03	739.73	-0.72	0.35	-2.04	0.042	0.479
ENST00000477549	DHX36	12.66	-1.92	0.65	-2.94	3.33E-03	6.24E-02	138.34	0.56	0.69	0.81	0.416	1.000
ENST00000481941	DHX36	7.52	-2.23	0.67	-3.32	9.03E-04	2.19E-02	2479.53	0.19	0.21	0.88	0.376	1.000
ENST00000496811	DHX36	208.52	-1.09	0.38	-2.89	3.85E-03	6.95E-02	5431.37	-0.07	0.17	-0.44	0.658	1.000
ENST00000540306	DHX8	14.65	-21.85	3.03	-7.22	5.35E-13	6.28E-11	268.00	-0.03	0.32	-0.10	0.921	1.000
ENST00000485081	DHX9	14.95	-21.88	3.03	-7.23	4.92E-13	5.90E-11	1257.68	1.17	0.18	6.58	0.000	0.000
ENST00000509182	DIMT1	14.76	-21.87	3.03	-7.22	5.14E-13	6.09E-11	85.74	-0.09	0.58	-0.16	0.875	1.000
ENST00000319194	DIS3L	29.88	-1.11	0.33	-3.38	7.31E-04	1.84E-02	310.68	0.25	1.44	0.18	0.859	1.000
ENST00000484317	DKC1	18.17	-7.32	1.80	-4.07	4.80E-05	1.72E-03	864.64	-0.08	0.20	-0.43	0.668	1.000
ENST00000525493	DKK3	28.00	-22.74	3.03	-7.51	5.88E-14	1.21E-11	101.95	0.22	0.67	0.32	0.748	1.000
ENST00000494493	DLG3	3.15	-4.79	1.81	-2.64	8.26E-03	1.21E-01	4.70	-1.07	2.71	-0.40	0.692	1.000
ENST00000475894	DLGAP4	8.44	-21.11	3.03	-6.97	3.18E-12	2.32E-10	5408.81	0.14	0.18	0.75	0.454	1.000
ENST00000554007	DLGAP5	5.02	-5.47	1.10	-4.96	7.09E-07	3.21E-05	49.42	-0.07	0.80	-0.08	0.935	1.000
ENST00000372289	DMAP1	33.96	-1.74	0.65	-2.66	7.86E-03	1.17E-01	166.41	-0.12	0.42	-0.28	0.781	1.000
ENST00000412350	DMRT2	2.70	-4.56	1.77	-2.58	9.90E-03	1.36E-01	189.86	0.19	0.39	0.48	0.633	1.000
ENST00000507552	DMXL1	3.05	-4.76	1.17	-4.07	4.74E-05	1.70E-03	207.99	0.39	0.76	0.51	0.610	1.000
ENST00000298292	DNAAF2	65.05	-2.24	0.55	-4.09	4.22E-05	1.53E-03	1767.72	0.08	0.18	0.46	0.648	1.000
ENST00000544625	DNAJA1	17.53	-21.69	3.03	-7.17	7.76E-13	8.00E-11	629.89	-0.41	0.36	-1.14	0.253	0.945
ENST00000264065	DNAJC10	257.87	-1.03	0.39	-2.63	8.57E-03	1.24E-01	16744.03	-0.01	0.12	-0.06	0.950	1.000
ENST00000294401	DNAJC11	8.85	-21.17	3.03	-6.99	2.75E-12	2.08E-10	403.51	-1.58	0.80	-1.97	0.049	0.521
ENST00000485073	DNAJC11	24.85	-7.77	1.04	-7.46	8.89E-14	1.62E-11	138.07	-0.53	0.43	-1.22	0.221	0.917
ENST00000260818	DNAJC13	93.72	-1.05	0.32	-3.32	9.16E-04	2.21E-02	3854.99	0.14	0.18	0.75	0.454	1.000
ENST00000475090	DNAJC2	5.11	-3.30	1.21	-2.72	6.44E-03	1.01E-01	44.27	0.39	0.48	0.81	0.419	1.000
ENST00000483637	DNAJC2	7.52	-4.29	1.05	-4.09	4.36E-05	1.57E-03	90.63	0.03	0.55	0.05	0.960	1.000
ENST00000512136	DNAJC21	5.21	-5.52	1.98	-2.79	5.32E-03	8.83E-02	1243.81	0.04	0.16	0.21	0.831	1.000
ENST00000457167	DNAJC7	335.80	-0.65	0.16	-4.01	6.16E-05	2.15E-03	4719.65	0.04	0.42	0.09	0.929	1.000
ENST00000553031	DNM1L	19.76	-7.44	1.86	-4.00	6.46E-05	2.24E-03	202.72	-0.15	0.26	-0.56	0.574	1.000
ENST00000540357	DNMT1	83.58	-3.98	0.99	-4.01	6.01E-05	2.10E-03	11853.54	-0.49	0.22	-2.23	0.026	0.370
ENST00000462779	DNPEP	4.89	-5.42	1.76	-3.07	2.12E-03	4.36E-02	12.50	-0.31	1.22	-0.26	0.797	1.000
ENST00000409592	DOCK10	38.74	-1.99	0.64	-3.12	1.83E-03	3.87E-02	900.84	1.28	3.48	0.37	0.712	1.000
ENST00000251157	DOCK7	91.40	-1.96	0.63	-3.11	1.87E-03	3.94E-02	5055.61	-0.02	0.46	-0.04	0.967	1.000
ENST00000467758	DOCK7	7.11	-5.96	2.10	-2.84	4.58E-03	7.89E-02	48.71	0.39	0.56	0.70	0.485	1.000
ENST00000372546	DOLPP1	7.90	-3.32	1.03	-3.21	1.33E-03	3.00E-02	1562.96	0.84	0.26	3.25	0.001	0.041
ENST00000370148	DPCD	7.61	-20.94	3.03	-6.91	4.81E-12	3.14E-10	298.44	0.68	1.06	0.65	0.518	1.000
ENST00000495421	DPH2	14.65	-7.01	2.15	-3.26	1.11E-03	2.59E-02	665.53	0.38	0.62	0.62	0.534	1.000
ENST00000371584	DPM1	15.27	-19.92	3.03	-6.58	4.72E-11	2.56E-09	3.53	-0.22	1.60	-0.14	0.888	NA
ENST00000449701	DPM1	13.37	-6.87	2.11	-3.26	1.13E-03	2.63E-02	116.94	0.09	0.43	0.22	0.828	1.000
ENST00000530165	DPP3	8.75	-21.14	3.03	-6.98	2.95E-12	2.21E-10	799.02	0.42	0.83	0.50	0.614	1.000
ENST00000531863	DPP3	15.44	-21.93	3.03	-7.24	4.42E-13	5.40E-11	2487.99	-0.53	0.84	-0.64	0.524	1.000
ENST00000339244	DPP8	3.83	-5.07	1.72	-2.96	3.12E-03	5.91E-02	21.49	7.81	4.79	1.63	0.103	0.731
ENST00000342179	DPY19L3	43.17	-1.22	0.43	-2.82	4.84E-03	8.22E-02	431.04	-0.15	0.48	-0.32	0.753	1.000
ENST00000370192	DPYD	524.48	-0.94	0.24	-3.91	9.09E-05	3.05E-03	10839.56	0.00	0.12	0.03	0.978	1.000
ENST00000523027	DPYSL2	28.56	-5.78	2.14	-2.70	6.86E-03	1.06E-01	36.23	-1.56	0.68	-2.29	0.022	0.335
ENST00000525501	DRAP1	6.24	-20.70	3.03	-6.83	8.46E-12	5.06E-10	905.14	-0.61	0.28	-2.16	0.031	0.406
ENST00000540275	DSE	13.07	-21.70	3.03	-7.17	7.74E-13	7.99E-11	75.78	0.35	0.88	0.39	0.694	1.000
ENST00000246069	DSTN	3362.05	-1.09	0.13	-8.33	7.85E-17	7.32E-14	64943.48	0.45	0.12	3.89	0.000	0.005

ENST00000449141	DSTN	4.36	-5.26	1.82	-2.88	3.95E-03	7.07E-02	579.28	0.34	0.26	1.33	0.184	0.878
ENST00000338950	DTNBP1	16.57	-6.59	1.79	-3.69	2.22E-04	6.68E-03	228.66	0.00	0.24	0.00	0.999	1.000
ENST00000344537	DTNBP1	26.77	-7.88	2.72	-2.90	3.72E-03	6.77E-02	216.56	-1.29	1.53	-0.84	0.399	1.000
ENST00000296161	DTX3L	39.44	-1.02	0.31	-3.30	9.79E-04	2.34E-02	4403.06	-0.05	0.12	-0.40	0.689	1.000
ENST00000400770	DTYMK	8.60	-21.14	3.03	-6.98	3.01E-12	2.24E-10	305.71	-0.24	0.38	-0.62	0.536	1.000
ENST00000331200	DUT	19.64	-7.43	2.09	-3.55	3.78E-04	1.05E-02	2025.97	-1.29	1.00	-1.30	0.195	0.892
ENST00000559852	DUT	34.89	-1.40	0.45	-3.09	1.99E-03	4.14E-02	176.54	-0.02	0.36	-0.05	0.958	1.000
ENST00000273130	DYNC1LI1	28.60	-7.97	2.77	-2.88	3.94E-03	7.06E-02	957.47	0.67	0.70	0.96	0.338	0.990
ENST00000472985	DYNC1LI1	14.21	-6.96	2.62	-2.65	7.95E-03	1.18E-01	46.85	-0.29	0.94	-0.30	0.761	1.000
ENST00000527884	E2F8	69.51	-0.95	0.28	-3.38	7.26E-04	1.83E-02	295.67	-0.11	0.38	-0.28	0.782	1.000
ENST00000236051	EBNA1BP2	550.87	-12.24	2.12	-5.76	8.38E-09	4.30E-07	1926.15	-0.09	0.25	-0.38	0.707	1.000
ENST00000474240	ECHDC1	12.39	-3.83	1.41	-2.72	6.49E-03	1.02E-01	9.40	-0.10	0.97	-0.10	0.918	1.000
ENST00000464057	ECI2	11.48	-6.66	1.88	-3.53	4.10E-04	1.13E-02	332.67	0.55	0.33	1.65	0.098	0.719
ENST00000380138	EDIL3	53.84	-8.89	2.86	-3.10	1.92E-03	4.03E-02	32238.12	0.15	0.18	0.88	0.379	1.000
ENST00000322349	EEA1	156.56	-1.03	0.35	-2.94	3.33E-03	6.23E-02	7479.15	-0.08	0.20	-0.41	0.683	1.000
ENST00000358190	EEF1A1	36.41	-22.02	2.82	-7.82	5.47E-15	1.98E-12	829.96	-0.70	0.51	-1.36	0.174	0.863
ENST00000455150	EEF1B2	64.67	-9.15	1.39	-6.59	4.35E-11	2.37E-09	3971.21	-0.46	0.27	-1.73	0.084	0.671
ENST00000447718	EEF1B2P3	298.48	-1.01	0.33	-3.10	1.95E-03	4.08E-02	110.34	-0.54	0.29	-1.84	0.066	0.599
ENST00000529516	EEF1D	36.05	-22.48	3.03	-7.43	1.11E-13	1.86E-11	16.82	0.58	0.68	0.85	0.396	1.000
ENST00000534377	EEF1D	25.16	-22.59	3.03	-7.46	8.54E-14	1.59E-11	58.82	0.09	0.38	0.23	0.814	1.000
ENST00000379715	EEF1E1	23.29	-1.50	0.47	-3.17	1.55E-03	3.38E-02	1652.72	0.10	0.18	0.53	0.596	1.000
ENST00000329251	EEF1G	28.80	-4.88	1.86	-2.62	8.84E-03	1.26E-01	19781.46	-0.12	0.18	-0.68	0.499	1.000
ENST00000371088	EFCAB7	6.92	-5.33	1.63	-3.26	1.11E-03	2.59E-02	2196.26	0.02	0.14	0.17	0.865	1.000
ENST00000422786	EIF2AK1	12.14	-5.54	1.65	-3.35	7.98E-04	1.97E-02	82.03	0.50	0.38	1.33	0.185	0.878
ENST00000405334	EIF2AK2	29.55	-7.06	2.72	-2.60	9.41E-03	1.32E-01	67.72	9.46	4.58	2.07	0.039	0.460
ENST00000461070	EIF2S2P4	259.87	-1.42	0.41	-3.45	5.53E-04	1.46E-02	267.42	0.16	0.21	0.78	0.437	1.000
ENST00000253039	EIF2S3	358.46	-2.26	0.38	-5.97	2.36E-09	1.23E-07	29453.81	-0.03	0.12	-0.24	0.807	1.000
ENST00000423068	EIF2S3	93.81	-9.69	0.97	-9.99	1.70E-23	5.10E-20	71.39	-0.78	0.49	-1.62	0.106	0.740
ENST00000360876	EIF3B	65.36	-23.89	3.03	-7.89	2.96E-15	1.31E-12	3465.04	0.60	0.54	1.10	0.271	0.957
ENST00000380876	EIF3CL	503.96	-6.85	1.45	-4.73	2.20E-06	9.56E-05	8707.55	-0.14	0.33	-0.43	0.667	1.000
ENST00000432675	EIF3D	14.10	-6.95	2.13	-3.27	1.09E-03	2.56E-02	74.76	-0.91	0.38	-2.38	0.017	0.292
ENST00000457241	EIF3D	18.97	-20.75	3.03	-6.85	7.27E-12	4.47E-10	64.73	0.50	0.64	0.77	0.441	1.000
ENST00000519627	EIF3E	16.55	-22.02	3.03	-7.27	3.49E-13	4.52E-11	110.02	-0.49	0.37	-1.33	0.183	0.877
ENST00000558227	EIF3J	10.61	-1.83	0.71	-2.59	9.50E-03	1.32E-01	18.39	-0.55	0.68	-0.81	0.416	1.000
ENST00000426808	EIF4A2	15.88	-7.12	2.61	-2.73	6.31E-03	1.00E-01	180.13	-0.61	0.43	-1.42	0.157	0.839
ENST00000475653	EIF4A2	5.45	-4.35	1.57	-2.76	5.70E-03	9.27E-02	263.25	-0.59	0.21	-2.86	0.004	0.113
ENST00000424196	EIF4G1	50.29	-22.53	3.03	-7.44	9.87E-14	1.69E-11	1468.38	0.31	0.55	0.56	0.575	1.000
ENST00000525681	EIF4G2	7.76	-21.00	3.03	-6.93	4.18E-12	2.83E-10	10.57	1.44	0.87	1.65	0.099	0.720
ENST00000525972	EIF4G2	8.08	-21.05	3.03	-6.95	3.66E-12	2.58E-10	11.37	-1.00	0.93	-1.07	0.286	0.964
ENST00000531180	EIF4G2	28.78	-22.77	3.03	-7.52	5.46E-14	1.14E-11	32.05	-0.71	0.69	-1.04	0.301	0.973
ENST00000392715	EIF5	5.11	-5.48	1.80	-3.04	2.34E-03	4.73E-02	9.45	0.39	1.68	0.23	0.819	1.000
ENST00000394235	ELF2	57.57	-0.71	0.23	-3.04	2.33E-03	4.71E-02	44.32	-1.69	1.20	-1.41	0.160	0.843
ENST00000497412	ELMO2	3.76	-2.80	1.06	-2.65	8.15E-03	1.20E-01	75.67	0.13	0.46	0.29	0.773	1.000
ENST00000252445	ELOF1	118.64	-0.76	0.22	-3.40	6.65E-04	1.70E-02	5558.99	-0.02	0.16	-0.12	0.902	1.000
ENST00000304434	ELOVL5	129.44	-8.06	0.78	-10.37	3.47E-25	1.46E-21	135.43	-0.43	0.85	-0.50	0.615	1.000
ENST00000535488	ELP2	14.17	-6.37	2.22	-2.87	4.07E-03	7.22E-02	19.19	-0.35	0.68	-0.52	0.602	1.000
ENST00000536373	ELP2	5.54	-20.54	3.03	-6.78	1.22E-11	6.84E-10	68.98	-0.13	0.55	-0.23	0.818	1.000
ENST00000544274	ELP2	10.55	-1.90	0.65	-2.94	3.26E-03	6.12E-02	49.55	0.15	0.45	0.34	0.735	1.000
ENST00000518112	ELP3	7.08	-20.83	3.03	-6.88	6.19E-12	3.86E-10	79.44	0.27	3.87	0.07	0.945	1.000
ENST00000492448	EMD	10.29	-3.49	1.22	-2.87	4.11E-03	7.28E-02	61.24	0.14	0.38	0.37	0.712	1.000
ENST00000497899	ENAH	4.37	-4.66	1.11	-4.18	2.92E-05	1.10E-03	212.65	-0.05	0.59	-0.08	0.933	1.000
ENST00000373203	ENG	17.69	-7.28	1.28	-5.69	1.25E-08	6.34E-07	4202.82	0.16	0.19	0.80	0.424	1.000
ENST00000509635	ENOPH1	46.71	-3.30	1.05	-3.15	1.65E-03	3.57E-02	465.23	0.57	0.71	0.80	0.423	1.000
ENST00000261488	ENOX1	7.31	-3.39	0.92	-3.70	2.19E-04	6.59E-03	1594.91	-0.33	0.37	-0.91	0.365	0.999
ENST00000522167	ENPP2	35.63	-2.37	0.55	-4.31	1.63E-05	6.44E-04	4638.66	-0.19	0.17	-1.09	0.274	0.959
ENST00000412989	ENSAP3	8.45	-1.94	0.60	-3.25	1.15E-03	2.66E-02	1.43	-0.78	4.46	-0.17	0.861	NA
ENST00000491099	EPC2	9.10	-2.44	0.65	-3.73	1.90E-04	5.85E-03	22.64	-0.02	1.30	-0.02	0.987	1.000
ENST00000506744	ERBB2IP	13.20	-6.86	2.65	-2.59	9.71E-03	1.34E-01	18.89	-0.45	0.63	-0.71	0.476	1.000

ENST00000551242	ERBB3	50.32	-22.54	3.03	-7.45	9.46E-14	1.64E-11	294.08	-0.16	0.20	-0.81	0.419	1.000
ENST00000490062	ERCC3	9.28	-21.24	3.03	-7.01	2.34E-12	1.87E-10	202.83	0.23	0.24	0.95	0.341	0.990
ENST00000407654	ERLIN1	25.85	-3.94	1.36	-2.90	3.76E-03	6.84E-02	203.33	-0.38	0.23	-1.64	0.100	0.722
ENST00000521644	ERLIN2	10.84	-21.45	3.03	-7.08	1.43E-12	1.28E-10	42.15	-0.20	0.43	-0.46	0.646	1.000
ENST00000554251	ERO1L	5.41	-20.49	3.03	-6.76	1.38E-11	7.68E-10	224.28	0.55	0.32	1.71	0.087	0.683
ENST00000556769	ERO1L	5.93	-20.64	3.03	-6.81	9.82E-12	5.71E-10	24.47	-0.25	0.70	-0.35	0.725	1.000
ENST00000546477	ERP29	21.35	-7.55	2.69	-2.81	4.93E-03	8.34E-02	2970.59	-0.23	0.28	-0.83	0.404	1.000
ENST00000523910	ESCO2	12.37	-6.76	2.62	-2.58	9.89E-03	1.36E-01	3.81	-1.19	1.52	-0.79	0.432	1.000
ENST00000412582	ESD	34.46	-8.24	2.73	-3.02	2.53E-03	5.04E-02	601.47	-0.13	0.39	-0.32	0.748	1.000
ENST00000495654	ESD	26.64	-7.87	2.89	-2.72	6.50E-03	1.02E-01	761.63	-0.35	0.28	-1.25	0.210	0.905
ENST00000541590	ESYT1	49.90	-3.81	1.47	-2.59	9.50E-03	1.32E-01	332.06	-0.06	0.21	-0.27	0.791	1.000
ENST00000559973	ETFA	3.53	-4.95	1.36	-3.63	2.82E-04	8.26E-03	77.59	0.01	0.77	0.02	0.986	1.000
ENST00000266517	ETNK1	8.90	-21.18	3.03	-6.99	2.68E-12	2.06E-10	2418.77	0.54	0.13	4.13	0.000	0.002
ENST00000531611	ETS1	7.15	-20.88	3.03	-6.89	5.47E-12	3.50E-10	165.19	0.29	2.43	0.12	0.905	1.000
ENST00000455726	EWSR1	10.50	-21.40	3.03	-7.07	1.58E-12	1.39E-10	425.70	0.55	0.37	1.48	0.139	0.810
ENST00000366547	EXO1	20.35	-22.30	3.03	-7.37	1.75E-13	2.62E-11	450.36	0.03	0.35	0.08	0.935	1.000
ENST00000315013	EXOC3	20.46	-20.96	3.03	-6.92	4.46E-12	2.98E-10	1445.98	0.93	0.64	1.45	0.148	0.823
ENST00000260762	EXOC6	15.82	-21.96	3.03	-7.25	4.08E-13	5.11E-11	1647.12	-0.28	0.33	-0.86	0.389	1.000
ENST00000243498	EXOSC9	10.27	-21.38	3.03	-7.06	1.70E-12	1.45E-10	8.82	0.76	1.08	0.71	0.481	1.000
ENST00000508212	EXOSC9	7.07	-20.87	3.03	-6.89	5.69E-12	3.60E-10	76.46	-0.21	0.37	-0.56	0.575	1.000
ENST00000469631	EZH2	2.74	-3.25	1.13	-2.88	3.98E-03	7.13E-02	118.52	-0.06	0.36	-0.18	0.861	1.000
ENST00000368444	FABP7	103.54	-2.50	0.88	-2.84	4.51E-03	7.80E-02	22292.96	0.21	0.12	1.79	0.074	0.632
ENST00000370035	FAM102B	4.95	-2.41	0.91	-2.64	8.19E-03	1.20E-01	2440.47	0.17	0.17	1.00	0.317	0.981
ENST00000515037	FAM114A1	8.96	-21.19	3.03	-7.00	2.62E-12	2.03E-10	371.16	0.46	1.31	0.35	0.726	1.000
ENST00000487732	FAM118A	4.92	-3.06	1.10	-2.79	5.33E-03	8.86E-02	48.28	-0.92	0.83	-1.11	0.269	0.955
ENST00000409185	FAM168B	22.09	-2.26	0.48	-4.68	2.83E-06	1.22E-04	37876.53	-0.01	0.11	-0.08	0.936	1.000
ENST00000238961	FAM178A	66.55	-1.30	0.31	-4.20	2.63E-05	1.01E-03	7369.21	-0.05	0.12	-0.42	0.678	1.000
ENST00000566077	FAM192A	26.95	-7.30	2.60	-2.81	4.98E-03	8.41E-02	1.76	-2.55	4.32	-0.59	0.555	NA
ENST00000567439	FAM192A	16.51	-22.02	3.03	-7.27	3.54E-13	4.57E-11	834.25	2.57	0.91	2.84	0.004	0.118
ENST00000490716	FAM48A	24.86	-0.93	0.32	-2.89	3.86E-03	6.97E-02	250.07	-3.30	2.16	-1.53	0.125	0.784
ENST00000520887	FAM49B	27.48	-3.31	0.86	-3.84	1.25E-04	4.00E-03	87.92	0.21	0.34	0.60	0.551	1.000
ENST00000523288	FAM49B	80.58	-1.52	0.42	-3.60	3.16E-04	9.07E-03	717.43	-0.37	0.34	-1.08	0.280	0.961
ENST00000380290	FAM96A	17.02	-7.22	2.65	-2.72	6.48E-03	1.02E-01	2851.28	0.13	0.14	0.90	0.368	1.000
ENST00000561607	FAN1	9.12	-6.32	1.61	-3.91	9.06E-05	3.04E-03	189.80	0.29	0.42	0.70	0.486	1.000
ENST00000480909	FANCD2	9.04	-6.31	1.72	-3.68	2.38E-04	7.08E-03	38.59	-0.26	0.99	-0.26	0.792	1.000
ENST00000561894	FANCI	31.43	-22.89	3.03	-7.56	3.99E-14	9.06E-12	2311.30	-1.49	0.57	-2.62	0.009	0.187
ENST00000281828	FARSB	564.91	-0.90	0.22	-4.14	3.41E-05	1.26E-03	7513.30	-0.01	0.13	-0.06	0.956	1.000
ENST00000355740	FAS	7.84	-3.67	1.36	-2.71	6.79E-03	1.05E-01	926.81	-0.42	0.58	-0.73	0.468	1.000
ENST00000434523	FBXO11	7.35	-20.93	3.03	-6.91	4.96E-12	3.22E-10	48.92	-9.13	4.67	-1.96	0.050	0.528
ENST00000493962	FBXO11	11.47	-21.53	3.03	-7.11	1.18E-12	1.10E-10	2664.00	-0.23	0.15	-1.47	0.140	0.811
ENST00000427718	FBXO21	80.12	-1.36	0.35	-3.92	8.86E-05	2.99E-03	3441.32	-0.15	0.22	-0.66	0.512	1.000
ENST00000523990	FBXO28	48.68	-8.74	2.76	-3.16	1.56E-03	3.40E-02	92.72	0.75	0.74	1.01	0.311	0.979
ENST00000509134	FBXO4	22.78	-5.53	1.79	-3.09	2.02E-03	4.20E-02	292.77	0.05	0.39	0.13	0.899	1.000
ENST00000397426	FBXO7	35.55	-8.29	3.03	-2.74	6.19E-03	9.87E-02	19271.91	0.18	0.59	0.31	0.756	1.000
ENST00000420700	FBXO7	17.17	-7.24	2.64	-2.74	6.15E-03	9.82E-02	1738.36	-3.79	1.49	-2.55	0.011	0.217
ENST00000443614	FDFT1	27.62	-22.72	3.03	-7.50	6.17E-14	1.25E-11	1204.99	0.32	0.26	1.21	0.227	0.923
ENST00000274457	FEM1C	57.43	-0.71	0.26	-2.75	5.91E-03	9.54E-02	4492.35	-0.03	0.14	-0.22	0.825	1.000
ENST00000327214	FGFR1OP2	6.12	-20.68	3.03	-6.82	8.95E-12	5.28E-10	572.60	0.10	0.29	0.33	0.739	1.000
ENST00000546072	FGFR1OP2	7.00	-5.94	1.87	-3.18	1.49E-03	3.28E-02	574.76	0.24	0.23	1.04	0.301	0.973
ENST00000395556	FIGNL1	6.89	-20.84	3.03	-6.88	6.07E-12	3.81E-10	58.96	-0.58	0.40	-1.45	0.147	0.822
ENST00000433017	FIGNL1	8.59	-5.04	1.89	-2.67	7.68E-03	1.15E-01	810.65	-0.17	0.22	-0.78	0.433	1.000
ENST00000223136	FIS1	148.29	-1.36	0.50	-2.74	6.13E-03	9.80E-02	4487.92	-0.21	0.20	-1.05	0.293	0.968
ENST00000269598	FKBP10	62.60	-8.24	3.03	-2.72	6.49E-03	1.02E-01	388.46	0.25	0.19	1.30	0.193	0.889
ENST00000489591	FKBP10	44.69	-22.51	3.03	-7.44	1.04E-13	1.75E-11	3109.57	0.44	0.14	3.20	0.001	0.047
ENST00000396062	FKBP3	31.64	-22.90	3.03	-7.57	3.87E-14	8.97E-12	3129.32	0.02	1.01	0.02	0.981	1.000
ENST00000368431	FLAD1	4.44	-3.45	1.03	-3.36	7.91E-04	1.96E-02	744.26	0.16	0.30	0.52	0.602	1.000
ENST00000360319	FLNA	262.53	-25.78	3.03	-8.52	1.61E-17	1.82E-14	61191.69	-0.77	1.36	-0.56	0.573	1.000
ENST00000466319	FLNA	132.13	-0.84	0.25	-3.34	8.36E-04	2.05E-02	74.60	-0.30	0.58	-0.52	0.605	1.000

ENST00000474358	FLNA	28.88	-7.99	1.53	-5.23	1.72E-07	8.25E-06	483.88	0.13	0.35	0.38	0.703	1.000
ENST00000293590	FMNL3	21.45	-21.08	3.03	-6.96	3.30E-12	2.38E-10	84.21	-0.32	0.52	-0.61	0.545	1.000
ENST00000356005	FN1	31.65	-22.89	3.03	-7.56	3.93E-14	9.01E-12	229532.66	-0.18	0.17	-1.07	0.285	0.963
ENST00000370256	FNBP1L	5.22	-5.52	1.33	-4.15	3.37E-05	1.25E-03	3077.86	-0.06	0.15	-0.42	0.671	1.000
ENST00000344768	FRMD6	42.10	-8.53	1.10	-7.75	9.25E-15	3.01E-12	439.52	-0.11	0.28	-0.38	0.702	1.000
ENST00000405801	FSCN1	42.80	-23.31	3.03	-7.70	1.34E-14	4.09E-12	20.62	-0.41	0.84	-0.49	0.624	1.000
ENST00000256759	FST	66.47	-2.92	0.49	-5.94	2.80E-09	1.46E-07	16713.93	-0.07	0.11	-0.65	0.517	1.000
ENST00000396947	FST	12.40	-4.93	1.48	-3.32	9.04E-04	2.19E-02	522.24	-0.04	0.24	-0.19	0.853	1.000
ENST00000424703	FSTL1	21.82	-22.40	3.03	-7.40	1.39E-13	2.16E-11	230.34	3.11	1.28	2.43	0.015	0.266
ENST00000319725	FUBP3	96.03	-1.25	0.46	-2.71	6.64E-03	1.03E-01	10482.09	-0.20	0.16	-1.24	0.216	0.912
ENST00000358307	FUT8	4.08	-5.16	1.97	-2.61	8.94E-03	1.27E-01	34.11	-0.82	1.36	-0.60	0.548	1.000
ENST00000461044	FXR1	10.75	-2.98	0.69	-4.31	1.67E-05	6.55E-04	36.79	0.54	0.81	0.67	0.505	1.000
ENST00000473375	FXR1	8.86	-2.17	0.82	-2.65	7.95E-03	1.18E-01	34.88	-0.94	0.60	-1.57	0.117	0.766
ENST00000435734	FXYD3	7.18	-4.80	1.72	-2.78	5.38E-03	8.90E-02	524.77	-1.01	0.21	-4.79	0.000	0.000
ENST00000270310	FXYD7	17.95	-2.52	0.89	-2.82	4.78E-03	8.14E-02	1637.64	0.79	0.21	3.77	0.000	0.008
ENST00000393564	G6PD	205.87	-10.82	2.04	-5.30	1.17E-07	5.66E-06	108.91	-0.09	0.42	-0.22	0.824	1.000
ENST00000534779	GANAB	211.24	-8.46	2.96	-2.86	4.27E-03	7.49E-02	1315.27	-0.24	0.28	-0.87	0.385	1.000
ENST00000396859	GAPDH	131.91	-24.84	3.03	-8.21	2.25E-16	1.78E-13	895.92	-0.06	0.35	-0.17	0.864	1.000
ENST00000447061	GAPDHP25	12.03	-1.95	0.64	-3.06	2.18E-03	4.47E-02	7.16	-0.60	0.99	-0.60	0.548	1.000
ENST00000505218	GAPDHP40	654.10	-1.23	0.28	-4.42	9.84E-06	3.98E-04	6.23	1.09	1.52	0.72	0.473	1.000
ENST00000396115	GAS7	193.05	-1.73	0.61	-2.85	4.43E-03	7.70E-02	132037.02	0.06	0.13	0.49	0.622	1.000
ENST00000429563	GATAD2A	8.83	-3.67	1.20	-3.05	2.33E-03	4.71E-02	66.26	-0.05	0.38	-0.13	0.894	1.000
ENST00000481729	GCC2	2.49	-4.46	1.72	-2.60	9.40E-03	1.32E-01	60.66	0.31	0.43	0.73	0.465	1.000
ENST00000220822	GDAP1	40.54	-2.35	0.64	-3.65	2.66E-04	7.84E-03	2247.62	-0.18	0.18	-0.98	0.329	0.985
ENST00000465640	GDI1	84.83	-0.90	0.33	-2.78	5.44E-03	8.98E-02	161.49	0.48	0.46	1.06	0.291	0.966
ENST00000380181	GDI2	75.58	-24.09	3.03	-7.96	1.75E-15	9.17E-13	3915.31	0.69	0.27	2.51	0.012	0.233
ENST00000456041	GDI2	8.19	-19.03	3.03	-6.28	3.34E-10	1.77E-08	822.07	1.02	0.73	1.39	0.165	0.848
ENST00000284116	GDPD1	6.10	-2.65	0.94	-2.83	4.65E-03	7.96E-02	1632.77	0.00	0.13	-0.01	0.991	1.000
ENST00000250379	GEMIN2	4.94	-5.44	1.75	-3.11	1.84E-03	3.89E-02	190.65	0.54	0.48	1.14	0.254	0.946
ENST00000525153	GEMIN2	9.13	-6.33	1.10	-5.75	9.10E-09	4.65E-07	106.75	1.17	0.94	1.25	0.210	0.906
ENST00000285873	GEMIN5	123.28	-0.92	0.28	-3.31	9.39E-04	2.26E-02	5253.85	0.01	0.12	0.06	0.950	1.000
ENST00000264263	GFM1	67.47	-3.86	1.25	-3.09	2.02E-03	4.20E-02	6609.28	0.11	0.12	0.96	0.335	0.988
ENST00000483256	GGNBP2	27.42	-7.91	2.72	-2.91	3.66E-03	6.70E-02	2114.58	0.42	0.20	2.06	0.039	0.464
ENST00000409196	GIGYF2	16.71	-22.04	3.03	-7.28	3.38E-13	4.42E-11	551.11	0.84	0.90	0.93	0.350	0.994
ENST00000328514	GINS3	18.03	-7.31	1.06	-6.92	4.39E-12	2.95E-10	645.50	-0.23	0.21	-1.14	0.253	0.946
ENST00000553118	GIT2	8.81	-6.27	1.88	-3.34	8.36E-04	2.05E-02	69.48	0.39	0.45	0.87	0.382	1.000
ENST00000373365	GLO1	170.19	-0.79	0.29	-2.74	6.09E-03	9.76E-02	18263.87	0.00	0.11	-0.04	0.967	1.000
ENST00000264428	GLRB	6.72	-2.46	0.73	-3.37	7.50E-04	1.88E-02	1278.77	0.01	0.33	0.02	0.984	1.000
ENST00000338435	GLS	27.02	-7.89	3.00	-2.63	8.49E-03	1.23E-01	9407.32	-0.05	0.14	-0.35	0.725	1.000
ENST00000461965	GLS	8.12	-6.16	1.70	-3.63	2.81E-04	8.23E-03	28.66	-0.70	0.74	-0.95	0.343	0.991
ENST00000495444	GLS	8.04	-6.14	1.71	-3.60	3.24E-04	9.24E-03	74.43	0.11	0.44	0.24	0.809	1.000
ENST00000490206	GNAI1	4.24	-5.22	1.74	-3.00	2.68E-03	5.25E-02	14.46	0.25	0.95	0.26	0.792	1.000
ENST00000424361	GNB2	6.17	-20.69	3.03	-6.83	8.70E-12	5.19E-10	221.65	-1.03	0.41	-2.54	0.011	0.219
ENST00000503494	GNB2L1	18.03	-22.13	3.03	-7.31	2.67E-13	3.65E-11	19.21	0.10	0.75	0.14	0.889	1.000
ENST00000504325	GNB2L1	16.41	-22.01	3.03	-7.27	3.64E-13	4.66E-11	334.44	-0.73	0.91	-0.79	0.428	1.000
ENST00000508682	GNB2L1	10.76	-21.42	3.03	-7.07	1.53E-12	1.35E-10	1138.93	-0.10	0.42	-0.24	0.809	1.000
ENST00000511900	GNB2L1	62.81	-22.97	3.03	-7.59	3.25E-14	7.79E-12	142.01	0.21	1.08	0.20	0.844	1.000
ENST00000515417	GNB2L1	9.70	-21.30	3.03	-7.03	2.05E-12	1.67E-10	46286.36	-0.97	1.22	-0.80	0.425	1.000
ENST00000466899	GNB4	7.93	-21.03	3.03	-6.94	3.90E-12	2.68E-10	53.26	-0.26	0.59	-0.44	0.662	1.000
ENST00000358784	GNB5	8.73	-21.12	3.03	-6.97	3.16E-12	2.31E-10	481.46	-0.10	0.23	-0.43	0.669	1.000
ENST00000370982	GNG12	121.58	-1.39	0.27	-5.24	1.61E-07	7.70E-06	41955.00	0.11	0.16	0.73	0.467	1.000
ENST00000433809	GNL1	16.97	-4.44	1.59	-2.80	5.14E-03	8.63E-02	595.97	0.26	0.35	0.75	0.454	1.000
ENST00000373062	GNL2	259.57	-0.94	0.29	-3.21	1.31E-03	2.95E-02	15340.44	-0.01	0.12	-0.12	0.906	1.000
ENST00000451249	GOSR1	33.78	-2.79	0.52	-5.31	1.08E-07	5.24E-06	1125.40	-0.02	0.22	-0.07	0.943	1.000
ENST00000355105	GPBP1L1	22.85	-1.07	0.41	-2.60	9.43E-03	1.32E-01	5654.18	0.18	0.24	0.74	0.457	1.000
ENST00000407583	GPN1	35.98	-8.30	3.03	-2.74	6.09E-03	9.75E-02	46.99	2.86	2.16	1.32	0.186	0.880
ENST00000228827	GPN3	43.24	-8.57	1.95	-4.39	1.14E-05	4.60E-04	1439.82	0.30	0.31	0.96	0.338	0.989
ENST00000372406	GPR107	49.24	-0.83	0.24	-3.41	6.50E-04	1.67E-02	64.38	0.82	1.27	0.64	0.521	1.000

ENST00000361314	GPX7	4.04	-2.52	0.94	-2.67	7.61E-03	1.14E-01	2877.31	-0.05	0.19	-0.25	0.805	1.000
ENST00000316804	GRB2	30.93	-3.05	1.08	-2.81	4.91E-03	8.31E-02	3768.14	2.74	0.96	2.87	0.004	0.111
ENST00000460882	GRHPR	50.76	-2.62	0.87	-3.00	2.66E-03	5.23E-02	1048.40	0.17	0.26	0.66	0.509	1.000
ENST00000513661	GRPEL2	22.82	-22.46	3.03	-7.42	1.19E-13	1.95E-11	63.91	0.54	0.45	1.20	0.228	0.924
ENST00000439371	GRSF1	28.03	-22.68	3.03	-7.49	6.84E-14	1.33E-11	3329.33	-0.06	4.56	-0.01	0.990	1.000
ENST00000341272	GSN	46.52	-23.42	3.03	-7.74	1.02E-14	3.28E-12	2622.00	0.38	0.29	1.31	0.190	0.884
ENST00000373806	GSN	39.96	-5.44	1.35	-4.04	5.35E-05	1.89E-03	83.81	0.51	0.41	1.25	0.213	0.909
ENST00000373807	GSN	31.17	-22.77	3.03	-7.52	5.38E-14	1.13E-11	12029.20	-0.05	0.13	-0.41	0.679	1.000
ENST00000432226	GSN	6.89	-20.84	3.03	-6.88	6.07E-12	3.81E-10	12.97	-4.06	2.18	-1.86	0.062	0.584
ENST00000485767	GSN	6.50	-5.83	1.90	-3.07	2.11E-03	4.36E-02	398.85	0.12	0.28	0.42	0.677	1.000
ENST00000326729	GSTM4	11.76	-6.69	2.58	-2.59	9.51E-03	1.32E-01	1761.76	-0.08	0.23	-0.36	0.715	1.000
ENST00000432659	GSTO1	21.61	-22.38	3.03	-7.39	1.44E-13	2.22E-11	631.08	0.09	0.17	0.49	0.625	1.000
ENST00000484743	GTF2A2	5.83	-20.61	3.03	-6.80	1.04E-11	5.99E-10	24.25	0.38	0.79	0.47	0.636	1.000
ENST00000330280	GTF2H2	14.00	-3.60	1.20	-3.00	2.69E-03	5.27E-02	562.91	0.00	0.27	-0.02	0.988	1.000
ENST00000521942	GTF2H2	7.76	-6.09	1.36	-4.48	7.35E-06	3.02E-04	492.58	0.09	0.27	0.34	0.735	1.000
ENST00000324896	GTF2I	67.32	-1.84	0.32	-5.68	1.34E-08	6.79E-07	1325.31	-0.20	0.22	-0.92	0.355	0.996
ENST00000470115	GTF3C2	7.23	-20.88	3.03	-6.89	5.56E-12	3.54E-10	118.05	-0.07	0.35	-0.19	0.848	1.000
ENST00000372146	GTF3C4	128.79	-0.86	0.28	-3.13	1.78E-03	3.78E-02	10460.96	0.05	0.11	0.42	0.677	1.000
ENST00000372097	GTF3C5	46.79	-1.95	0.52	-3.73	1.92E-04	5.89E-03	1540.66	0.05	0.14	0.34	0.730	1.000
ENST00000512507	H2AFY	132.27	-2.15	0.65	-3.29	1.01E-03	2.40E-02	10862.13	0.10	0.12	0.80	0.422	1.000
ENST00000511319	H2AFZ	83.21	-24.22	3.03	-8.00	1.23E-15	7.19E-13	2743.42	-0.78	0.50	-1.56	0.119	0.772
ENST00000466976	HABP4	3.26	-4.85	1.63	-2.98	2.89E-03	5.57E-02	114.35	0.00	0.31	0.00	0.996	1.000
ENST00000309522	HADH	44.41	-23.23	3.03	-7.68	1.65E-14	4.77E-12	2489.97	-0.06	0.29	-0.22	0.826	1.000
ENST00000507260	HADH	5.79	-3.17	1.21	-2.63	8.54E-03	1.23E-01	863.20	-0.06	0.20	-0.33	0.741	1.000
ENST00000405867	HADHB	8.55	-21.12	3.03	-6.97	3.11E-12	2.29E-10	2634.89	0.05	0.32	0.16	0.873	1.000
ENST00000545822	HADHB	13.01	-21.69	3.03	-7.16	7.84E-13	8.05E-11	2507.90	0.23	0.75	0.31	0.755	1.000
ENST00000415192	HARS	53.70	-8.88	1.99	-4.46	8.31E-06	3.38E-04	94.49	-0.51	0.59	-0.86	0.388	1.000
ENST00000570178	HAUS2	11.83	-21.57	3.03	-7.12	1.06E-12	1.01E-10	13.10	-0.11	3.93	-0.03	0.977	1.000
ENST00000380502	HAUS6	103.60	-0.51	0.16	-3.16	1.60E-03	3.48E-02	8536.30	0.03	0.14	0.22	0.825	1.000
ENST00000476391	HDAC1	24.19	-6.55	2.38	-2.75	5.99E-03	9.65E-02	2113.74	0.04	0.70	0.05	0.960	1.000
ENST00000520746	HDAC2	13.55	-6.90	1.85	-3.73	1.90E-04	5.84E-03	116.56	-0.59	0.96	-0.62	0.537	1.000
ENST00000486618	HDAC3	9.56	-4.29	1.17	-3.68	2.35E-04	7.02E-03	135.92	0.73	0.45	1.62	0.106	0.740
ENST00000491581	HDAC3	5.58	-5.61	2.05	-2.73	6.31E-03	1.00E-01	61.93	0.23	0.37	0.63	0.532	1.000
ENST00000456922	HDAC4	2.55	-4.49	1.41	-3.18	1.47E-03	3.25E-02	81.73	0.17	0.56	0.31	0.760	1.000
ENST00000405943	HDGFP1	17.52	-2.17	0.65	-3.33	8.61E-04	2.10E-02	16.08	0.84	0.97	0.86	0.388	1.000
ENST00000555843	HECTD1	5.22	-5.52	1.83	-3.01	2.60E-03	5.14E-02	5499.13	0.04	0.14	0.33	0.742	1.000
ENST00000247815	HELB	12.56	-6.79	1.82	-3.72	1.96E-04	6.01E-03	127.24	-1.24	0.97	-1.28	0.200	0.895
ENST00000466552	HELLS	4.83	-4.19	1.26	-3.33	8.54E-04	2.09E-02	331.72	0.10	1.07	0.09	0.928	1.000
ENST00000427635	HERC4	49.70	-1.26	0.23	-5.38	7.47E-08	3.66E-06	1249.44	1.36	0.49	2.79	0.005	0.131
ENST00000380265	HERC6	2.71	-2.72	1.00	-2.73	6.42E-03	1.01E-01	146.30	-0.43	2.85	-0.15	0.880	1.000
ENST00000344114	HERPUD1	2.74	-4.59	1.70	-2.70	6.96E-03	1.07E-01	48.11	-0.72	0.59	-1.23	0.219	0.914
ENST00000439977	HERPUD1	3.64	-5.00	1.12	-4.48	7.40E-06	3.04E-04	151.97	-0.64	0.58	-1.10	0.273	0.958
ENST00000504459	HEXB	25.58	-5.42	1.86	-2.90	3.68E-03	6.71E-02	491.17	0.17	0.27	0.64	0.525	1.000
ENST00000513079	HEXB	16.87	-7.21	2.67	-2.70	6.98E-03	1.07E-01	1311.85	-0.04	0.27	-0.13	0.893	1.000
ENST00000354724	HEY1	8.40	-6.21	1.27	-4.88	1.08E-06	4.84E-05	193.44	0.55	0.75	0.74	0.462	1.000
ENST00000359678	HIBCH	38.75	-8.41	2.76	-3.05	2.31E-03	4.68E-02	723.09	-0.33	0.51	-0.65	0.518	1.000
ENST00000418900	HIGD1A	9.60	-21.28	3.03	-7.03	2.11E-12	1.71E-10	431.37	0.29	2.13	0.14	0.892	1.000
ENST00000377791	HIST1H2AC	445.16	-2.31	0.25	-9.08	1.11E-19	2.11E-16	81.17	1.11	1.92	0.58	0.564	1.000
ENST00000359985	HIST1H2BF	325.63	-0.82	0.27	-3.04	2.35E-03	4.73E-02	26.37	1.71	0.78	2.20	0.028	0.388
ENST00000356350	HIST1H2BH	183.53	-1.26	0.26	-4.91	9.03E-07	4.06E-05	42.71	-0.05	0.50	-0.09	0.927	1.000
ENST00000339812	HIST1H2BJ	495.10	-1.04	0.27	-3.86	1.11E-04	3.62E-03	41.50	0.20	0.59	0.35	0.730	1.000
ENST00000377831	HIST1H3D	40.31	-1.51	0.32	-4.75	2.04E-06	8.91E-05	6.52	-2.55	1.22	-2.08	0.037	0.450
ENST00000354348	HIST1H4I	295.72	-0.82	0.24	-3.43	5.94E-04	1.55E-02	178.29	0.99	0.32	3.10	0.002	0.061
ENST00000355981	HIST1H4L	109.68	-1.11	0.33	-3.32	9.00E-04	2.18E-02	3.46	-5.31	2.75	-1.93	0.054	NA
ENST00000369155	HIST2H2BE	317.67	-1.39	0.23	-5.92	3.14E-09	1.63E-07	5975.88	1.35	0.21	6.41	0.000	0.000
ENST00000392939	HIST2H4A	346.34	-2.66	0.84	-3.18	1.48E-03	3.26E-02	819.46	0.10	0.16	0.64	0.524	1.000
ENST00000412585	HLA-B	71.97	-2.78	0.82	-3.41	6.51E-04	1.67E-02	3391.99	-0.10	0.12	-0.79	0.430	1.000
ENST00000422592	HLA-DPB1	41.82	-5.92	1.45	-4.10	4.16E-05	1.51E-03	6241.82	0.07	0.26	0.25	0.800	1.000

ENST00000310053	HLTF	148.38	-1.20	0.18	-6.78	1.20E-11	6.80E-10	120.67	0.14	0.41	0.33	0.738	1.000
ENST00000340852	HM13	20.42	-7.49	2.73	-2.74	6.16E-03	9.83E-02	147.53	-0.54	0.31	-1.74	0.082	0.662
ENST00000494153	HM13	17.68	-7.28	1.89	-3.85	1.17E-04	3.79E-03	16.13	-0.40	0.70	-0.57	0.566	1.000
ENST00000416526	HMG20B	7.91	-21.03	3.03	-6.94	3.91E-12	2.68E-10	57.20	-0.02	1.92	-0.01	0.991	1.000
ENST00000405805	HMGB1	96.47	-1.91	0.74	-2.60	9.40E-03	1.32E-01	8941.19	0.89	0.26	3.42	0.001	0.026
ENST00000513148	HMGCL	4.87	-4.22	1.29	-3.28	1.05E-03	2.47E-02	337.02	0.11	0.73	0.15	0.880	1.000
ENST00000358715	HMMR	42.86	-8.56	2.80	-3.05	2.28E-03	4.64E-02	1887.80	-1.04	0.65	-1.60	0.110	0.750
ENST00000562569	HN1L	31.00	-22.87	3.03	-7.56	4.14E-14	9.34E-12	19.20	-0.74	2.70	-0.27	0.784	1.000
ENST00000556897	HNRNPC	27.08	-22.13	3.03	-7.31	2.65E-13	3.64E-11	8065.28	0.12	0.22	0.55	0.580	1.000
ENST00000515432	HNRNPD	18.11	-22.14	3.03	-7.31	2.62E-13	3.60E-11	115.44	0.18	0.36	0.52	0.606	1.000
ENST00000481819	HNRNPH3	12.68	-21.66	3.03	-7.15	8.42E-13	8.51E-11	1693.63	0.25	0.34	0.73	0.463	1.000
ENST00000376281	HNRNPK	70.84	-24.00	3.03	-7.93	2.22E-15	1.07E-12	7.37	-1.19	1.32	-0.90	0.369	1.000
ENST00000470941	HNRNPR	44.09	-8.60	1.91	-4.51	6.53E-06	2.70E-04	103.02	-0.43	0.38	-1.13	0.259	0.950
ENST00000283179	HNRNPU	58.72	-5.59	2.01	-2.78	5.50E-03	9.04E-02	45003.75	-0.27	0.25	-1.10	0.269	0.955
ENST00000349655	HNRPDL	99.73	-24.46	3.03	-8.08	6.30E-16	4.07E-13	1074.53	-0.36	0.36	-0.98	0.326	0.985
ENST00000378915	HNRPLL	23.16	-7.67	2.72	-2.82	4.84E-03	8.22E-02	79.80	-1.62	2.25	-0.72	0.470	1.000
ENST00000424732	HP1BP3	58.84	-1.00	0.35	-2.84	4.51E-03	7.80E-02	1838.44	-0.14	0.22	-0.62	0.532	1.000
ENST00000513628	HSD17B4	25.80	-22.62	3.03	-7.47	7.79E-14	1.47E-11	4490.43	0.81	3.80	0.21	0.830	1.000
ENST00000549334	HSP90B1	24.08	-22.53	3.03	-7.44	9.89E-14	1.69E-11	44.52	-0.31	0.42	-0.73	0.466	1.000
ENST00000532167	HSPA8	13.47	-21.74	3.03	-7.18	7.06E-13	7.46E-11	781.42	-0.60	0.28	-2.15	0.032	0.412
ENST00000425843	HSPA8P1	1450.14	-1.09	0.27	-4.06	4.81E-05	1.72E-03	63.46	-0.65	0.44	-1.47	0.141	0.813
ENST00000504902	HSPA9	75.19	-3.08	0.82	-3.75	1.74E-04	5.38E-03	3665.87	-0.42	0.26	-1.62	0.105	0.739
ENST00000524109	HSPA9	9.54	-21.28	3.03	-7.02	2.15E-12	1.74E-10	1428.55	-0.22	0.27	-0.81	0.417	1.000
ENST00000418022	HSPD1	7.22	-2.77	1.06	-2.63	8.59E-03	1.24E-01	50.57	-0.21	0.68	-0.30	0.762	1.000
ENST00000537078	HSPD1P1	21.13	-1.80	0.60	-3.00	2.74E-03	5.34E-02	14.74	-2.09	1.46	-1.43	0.152	0.829
ENST00000218364	HTATSF1	40.92	-8.49	3.02	-2.81	5.00E-03	8.43E-02	636.28	1.12	1.12	1.00	0.317	0.981
ENST00000496603	IAH1	6.00	-5.72	1.90	-3.00	2.69E-03	5.26E-02	24.83	0.47	0.57	0.81	0.418	1.000
ENST00000473915	IARS	6.75	-5.89	1.24	-4.76	1.98E-06	8.65E-05	48.48	-0.34	0.48	-0.72	0.471	1.000
ENST00000265986	IDE	191.13	-0.78	0.22	-3.58	3.42E-04	9.66E-03	6438.21	-0.01	0.16	-0.09	0.931	1.000
ENST00000484575	IDH1	20.00	-7.46	2.64	-2.82	4.73E-03	8.07E-02	344.16	-1.01	0.65	-1.55	0.121	0.776
ENST00000558535	IDH3A	12.77	-6.81	2.04	-3.34	8.42E-04	2.06E-02	2827.71	0.00	0.13	0.01	0.995	1.000
ENST00000479376	IDH3B	9.36	-2.38	0.68	-3.49	4.85E-04	1.31E-02	144.84	0.42	0.37	1.13	0.259	0.950
ENST00000370092	IDH3G	10.95	-4.87	1.25	-3.90	9.49E-05	3.16E-03	272.07	-0.47	0.30	-1.56	0.118	0.769
ENST00000295809	IFI16	29.29	-7.42	2.42	-3.07	2.12E-03	4.36E-02	3659.38	0.04	0.18	0.25	0.803	1.000
ENST00000448393	IFI16	67.93	-9.22	2.81	-3.28	1.04E-03	2.47E-02	9097.69	-0.11	0.13	-0.81	0.417	1.000
ENST00000562225	IFI16	26.63	-7.87	1.99	-3.95	7.74E-05	2.64E-03	59.90	-0.17	0.40	-0.42	0.673	1.000
ENST00000438486	IFI44	6.51	-20.73	3.03	-6.84	7.93E-12	4.80E-10	27.64	-0.10	0.83	-0.12	0.903	1.000
ENST00000263642	IFIH1	4.52	-3.05	1.09	-2.79	5.29E-03	8.80E-02	2237.80	0.17	0.13	1.33	0.182	0.875
ENST00000408968	IFITM1	27.81	-1.71	0.52	-3.28	1.03E-03	2.44E-02	380.29	-3.08	1.05	-2.94	0.003	0.094
ENST00000399815	IFITM2	12.49	-1.86	0.70	-2.66	7.84E-03	1.17E-01	115.80	-0.39	0.48	-0.81	0.418	1.000
ENST00000346192	IGF2BP2	10.85	-2.63	0.81	-3.25	1.14E-03	2.64E-02	1014.96	0.01	0.29	0.04	0.969	1.000
ENST00000496495	IGF2BP2	6.12	-3.19	0.97	-3.29	9.96E-04	2.37E-02	167.63	0.14	0.25	0.57	0.566	1.000
ENST00000417621	IGFBP3	5.73	-5.66	1.73	-3.26	1.11E-03	2.59E-02	127.34	-1.17	0.62	-1.90	0.058	0.565
ENST00000374647	IKBKAP	128.60	-0.67	0.24	-2.80	5.11E-03	8.59E-02	9161.92	0.09	0.13	0.66	0.508	1.000
ENST00000422485	IL1RAP	9.37	-21.23	3.03	-7.01	2.41E-12	1.90E-10	211.90	-0.10	1.57	-0.06	0.950	1.000
ENST00000250241	ILF3	17.28	-7.24	1.48	-4.88	1.06E-06	4.74E-05	9573.43	-0.35	0.17	-2.08	0.038	0.454
ENST00000520065	IMPA1	5.38	-5.56	1.70	-3.26	1.10E-03	2.56E-02	72.98	0.04	0.42	0.09	0.930	1.000
ENST00000326739	IMPDH2	403.03	-1.48	0.44	-3.34	8.34E-04	2.05E-02	12761.91	-0.16	0.15	-1.05	0.292	0.967
ENST00000442157	IMPDH2	18.18	-22.15	3.03	-7.32	2.57E-13	3.56E-11	79.02	0.74	0.35	2.13	0.034	0.426
ENST00000462980	IMPDH2	2.93	-4.68	1.09	-4.30	1.68E-05	6.61E-04	947.27	-0.35	0.23	-1.49	0.136	0.803
ENST00000339121	ING3	13.43	-3.69	1.13	-3.27	1.09E-03	2.56E-02	302.60	-0.21	0.28	-0.73	0.462	1.000
ENST00000283410	INO80C	5.78	-20.60	3.03	-6.80	1.06E-11	6.11E-10	42.49	-5.74	3.50	-1.64	0.101	0.725
ENST00000368670	INTS3	6.79	-20.82	3.03	-6.87	6.37E-12	3.96E-10	196.19	-0.30	1.36	-0.22	0.825	1.000
ENST00000366994	INTS7	21.62	-21.58	3.03	-7.13	1.02E-12	9.73E-11	1227.55	0.75	0.41	1.84	0.066	0.600
ENST00000403772	IPO5	14.45	-21.84	3.03	-7.21	5.53E-13	6.41E-11	30.49	0.23	0.79	0.30	0.766	1.000
ENST00000490680	IPO5	193.63	-25.36	3.03	-8.38	5.32E-17	5.07E-14	1355.88	0.05	0.61	0.08	0.937	1.000
ENST00000491555	IPO5	89.48	-9.62	2.89	-3.33	8.65E-04	2.11E-02	7275.36	-0.13	0.16	-0.78	0.433	1.000
ENST00000542464	IPO8	8.02	-21.04	3.03	-6.95	3.74E-12	2.61E-10	14.08	-0.54	0.83	-0.64	0.519	1.000

ENST00000544829	IPO8	23.00	-22.44	3.03	-7.41	1.23E-13	1.97E-11	104.48	-0.21	0.66	-0.32	0.752	1.000
ENST00000310864	IQCB1	7.18	-3.13	0.82	-3.84	1.24E-04	3.98E-03	2162.56	-0.06	0.21	-0.28	0.777	1.000
ENST00000561461	IQGAP1	21.51	-1.33	0.50	-2.67	7.49E-03	1.13E-01	152.46	-0.36	0.40	-0.91	0.363	0.999
ENST00000369940	IRAK1BP1	2.80	-3.30	1.21	-2.72	6.52E-03	1.02E-01	623.17	-0.05	0.22	-0.22	0.825	1.000
ENST00000258886	IREB2	98.59	-1.40	0.42	-3.32	9.05E-04	2.19E-02	12745.02	0.03	0.13	0.20	0.842	1.000
ENST00000538850	IST1	54.79	-8.91	1.92	-4.65	3.29E-06	1.41E-04	32.86	-8.56	4.76	-1.80	0.072	0.626
ENST00000544001	ITFG1	14.46	-21.84	3.03	-7.21	5.52E-13	6.41E-11	1003.44	-0.12	0.39	-0.31	0.754	1.000
ENST00000228799	ITFG2	3.80	-2.42	0.91	-2.67	7.62E-03	1.14E-01	967.19	0.01	0.22	0.05	0.956	1.000
ENST00000296585	ITGA2	85.22	-0.91	0.34	-2.65	8.02E-03	1.18E-01	9613.48	-0.24	0.53	-0.45	0.652	1.000
ENST00000264107	ITGA6	29.90	-22.81	3.03	-7.53	4.89E-14	1.06E-11	2093.76	-0.84	1.36	-0.61	0.540	1.000
ENST00000409080	ITGA6	8.57	-21.12	3.03	-6.97	3.16E-12	2.31E-10	3098.59	-1.70	0.31	-5.51	0.000	0.000
ENST00000430709	ITGAV	19.00	-7.38	1.19	-6.21	5.41E-10	2.86E-08	167.14	0.05	0.27	0.18	0.855	1.000
ENST00000474571	ITGAV	13.26	-6.87	1.20	-5.73	1.01E-08	5.16E-07	14.51	0.14	0.75	0.19	0.848	1.000
ENST00000496477	ITGAV	12.22	-6.75	1.26	-5.37	7.74E-08	3.79E-06	36.77	0.00	0.63	0.01	0.995	1.000
ENST00000360635	ITGB1BP1	5.46	-4.38	1.28	-3.42	6.17E-04	1.60E-02	830.63	-0.46	0.31	-1.50	0.133	0.799
ENST00000460394	ITGB3BP	17.16	-2.63	1.01	-2.61	9.08E-03	1.29E-01	166.62	-0.04	0.28	-0.16	0.875	1.000
ENST00000218436	ITIH6	46.04	-2.02	0.46	-4.41	1.01E-05	4.10E-04	20057.93	-0.27	0.12	-2.31	0.021	0.328
ENST00000492029	ITM2C	8.94	-21.19	3.03	-6.99	2.66E-12	2.05E-10	70.96	0.48	0.38	1.28	0.199	0.894
ENST00000553695	ITPK1	8.12	-21.05	3.03	-6.95	3.67E-12	2.58E-10	14.33	0.20	0.79	0.25	0.800	1.000
ENST00000429204	ITPKB	59.44	-1.02	0.31	-3.26	1.13E-03	2.63E-02	309.25	0.08	1.40	0.06	0.954	1.000
ENST00000406921	ITSN2	9.87	-21.32	3.03	-7.04	1.92E-12	1.60E-10	50.48	2.29	1.53	1.50	0.134	0.801
ENST00000487418	IVD	5.27	-20.45	3.03	-6.75	1.50E-11	8.36E-10	1112.68	-0.82	0.40	-2.07	0.038	0.456
ENST00000494880	IVNS1ABP	26.50	-7.86	2.74	-2.87	4.06E-03	7.21E-02	949.44	-0.15	0.25	-0.58	0.564	1.000
ENST00000387429	J01415.15	17.84	-2.81	0.62	-4.52	6.29E-06	2.61E-04	9378.99	0.47	0.15	3.23	0.001	0.044
ENST00000387449	J01415.18	387.92	-0.94	0.24	-3.87	1.07E-04	3.50E-03	16085.72	0.30	0.15	2.03	0.042	0.480
ENST00000387456	J01415.19	37.98	-2.60	0.51	-5.12	3.10E-07	1.45E-05	20242.63	0.31	0.17	1.86	0.063	0.586
ENST00000387460	J01415.21	21.95	-1.68	0.38	-4.47	7.80E-06	3.19E-04	4408.77	0.30	0.17	1.76	0.079	0.654
ENST00000361851	J01415.24	61.85	-1.52	0.43	-3.55	3.79E-04	1.06E-02	123212.11	0.14	0.12	1.15	0.251	0.942
ENST00000387342	J01415.3	103.78	-3.17	0.50	-6.39	1.62E-10	8.71E-09	2660.54	0.20	0.18	1.11	0.265	0.953
ENST00000441717	JAM3	16.82	-22.04	3.03	-7.28	3.32E-13	4.35E-11	194.45	-0.24	0.48	-0.49	0.627	1.000
ENST00000565258	JMJD8	3.30	-4.85	1.80	-2.70	7.03E-03	1.08E-01	32.02	0.14	0.66	0.21	0.835	1.000
ENST00000482442	JOSD1	5.57	-5.60	1.11	-5.06	4.15E-07	1.92E-05	65.28	0.16	0.62	0.26	0.797	1.000
ENST00000570215	KARS	6.01	-3.95	1.49	-2.65	7.95E-03	1.18E-01	104.62	-0.50	0.42	-1.18	0.236	0.930
ENST00000377046	KAT5	7.30	-20.91	3.03	-6.90	5.12E-12	3.32E-10	129.18	-0.19	0.51	-0.37	0.713	1.000
ENST00000510829	KCTD7	3.02	-4.72	1.63	-2.90	3.77E-03	6.85E-02	286.26	-0.05	0.53	-0.10	0.919	1.000
ENST00000400181	KDM1A	100.12	-0.95	0.31	-3.03	2.44E-03	4.90E-02	230.30	1.61	1.01	1.60	0.109	0.749
ENST00000406396	KDSR	28.12	-1.40	0.43	-3.29	9.92E-04	2.36E-02	9418.94	0.06	0.13	0.49	0.625	1.000
ENST00000518728	KHDRBS3	9.73	-21.30	3.03	-7.03	2.02E-12	1.65E-10	1.54	0.33	4.29	0.08	0.939	NA
ENST00000397885	KIAA0020	212.25	-0.68	0.17	-4.03	5.52E-05	1.95E-03	8569.42	0.03	0.16	0.16	0.873	1.000
ENST00000558043	KIAA0101	14.64	-1.83	0.69	-2.65	7.99E-03	1.18E-01	242.25	0.72	0.45	1.60	0.110	0.750
ENST00000194118	KIAA0141	4.65	-5.35	1.06	-5.03	4.81E-07	2.21E-05	912.49	-0.22	0.35	-0.64	0.524	1.000
ENST00000259335	KIAA0368	263.08	-0.56	0.19	-2.97	2.94E-03	5.66E-02	877.94	-1.29	1.30	-1.00	0.318	0.982
ENST00000465499	KIAA0368	17.80	-22.12	3.03	-7.31	2.75E-13	3.74E-11	623.01	-0.25	0.68	-0.37	0.709	1.000
ENST00000557565	KIAA0391	132.15	-1.32	0.41	-3.22	1.30E-03	2.94E-02	1525.69	-0.27	0.28	-0.98	0.329	0.985
ENST00000546967	KIAA0430	3.95	-3.89	1.05	-3.72	1.98E-04	6.06E-03	8.77	-1.48	0.99	-1.50	0.135	0.801
ENST00000548534	KIAA1033	3.94	-3.25	1.12	-2.91	3.58E-03	6.60E-02	190.69	0.24	0.27	0.89	0.372	1.000
ENST00000295746	KIAA1524	227.97	-0.74	0.20	-3.67	2.42E-04	7.18E-03	2077.81	-0.07	0.27	-0.27	0.787	1.000
ENST00000378814	KIF13A	48.09	-6.05	0.79	-7.68	1.63E-14	4.76E-12	5206.55	0.09	0.23	0.39	0.698	1.000
ENST00000407071	KIF26B	4.91	-5.43	1.75	-3.10	1.94E-03	4.05E-02	1066.88	-0.37	0.18	-2.04	0.042	0.479
ENST00000401507	KIF2A	35.07	-23.04	3.03	-7.61	2.73E-14	6.95E-12	2508.44	-0.74	1.29	-0.58	0.565	1.000
ENST00000455186	KIF2C	28.32	-7.96	2.72	-2.93	3.41E-03	6.35E-02	70.94	-0.16	2.45	-0.06	0.949	1.000
ENST00000302418	KIF5B	594.86	-0.73	0.16	-4.62	3.87E-06	1.64E-04	26634.74	-0.01	0.14	-0.09	0.931	1.000
ENST00000543003	KIN	16.32	-2.91	1.06	-2.75	5.91E-03	9.54E-02	353.18	-0.03	0.32	-0.09	0.925	1.000
ENST00000392647	KLHL23	29.20	-1.08	0.30	-3.54	3.97E-04	1.10E-02	2553.60	0.76	0.80	0.95	0.343	0.991
ENST00000545252	KLHL8	8.02	-21.04	3.03	-6.95	3.74E-12	2.61E-10	7.45	-6.41	2.19	-2.93	0.003	0.096
ENST00000333479	KNTC1	54.93	-1.33	0.46	-2.90	3.77E-03	6.85E-02	12161.65	-0.35	0.12	-3.00	0.003	0.081
ENST00000330459	KPNA2	202.90	-25.20	3.03	-8.33	8.25E-17	7.52E-14	26104.19	-0.07	0.11	-0.62	0.537	1.000
ENST00000446515	KPNA6	12.32	-21.62	3.03	-7.14	9.31E-13	9.16E-11	23.86	-0.10	1.15	-0.09	0.932	1.000

ENST00000537679	KPNB1	482.23	-26.61	3.03	-8.79	1.44E-18	2.51E-15	58.53	-9.39	4.61	-2.04	0.042	0.479
ENST00000551070	KRR1	14.48	-6.99	1.80	-3.89	1.02E-04	3.34E-03	118.04	-0.90	0.85	-1.06	0.288	0.965
ENST00000276590	LACTB2	33.44	-1.52	0.55	-2.77	5.55E-03	9.10E-02	672.82	0.04	0.24	0.15	0.882	1.000
ENST00000389463	LAMA4	48.13	-22.56	3.03	-7.45	9.17E-14	1.64E-11	17732.81	0.12	0.12	1.05	0.294	0.969
ENST00000521693	LAMA4	17.31	-22.08	3.03	-7.29	3.05E-13	4.09E-11	135.99	0.26	0.33	0.79	0.430	1.000
ENST00000521732	LAMA4	4.19	-3.44	1.04	-3.32	8.90E-04	2.16E-02	26.25	0.40	0.95	0.42	0.674	1.000
ENST00000515100	LAMTOR3	7.44	-5.43	1.52	-3.58	3.38E-04	9.57E-03	194.19	-0.07	0.31	-0.24	0.812	1.000
ENST00000412863	LANCL1	13.71	-6.91	2.68	-2.58	9.99E-03	1.37E-01	31.75	-0.86	0.81	-1.06	0.288	0.965
ENST00000326639	LARP1B	8.18	-2.65	0.68	-3.87	1.07E-04	3.50E-03	166.13	-0.64	0.87	-0.74	0.461	1.000
ENST00000539715	LARS	93.82	-9.69	2.87	-3.37	7.38E-04	1.85E-02	1099.41	1.09	1.39	0.79	0.431	1.000
ENST00000379509	LCLAT1	13.86	-21.77	3.03	-7.19	6.58E-13	7.15E-11	1599.96	-0.16	0.47	-0.33	0.739	1.000
ENST00000396222	LDHA	83.57	-9.52	1.16	-8.24	1.78E-16	1.46E-13	3605.04	-1.31	0.23	-5.74	0.000	0.000
ENST00000537296	LDHA	55.79	-1.30	0.49	-2.63	8.55E-03	1.23E-01	675.57	-1.51	0.37	-4.10	0.000	0.002
ENST00000350669	LDHB	152.23	-10.38	2.02	-5.15	2.58E-07	1.21E-05	16201.49	1.23	1.11	1.11	0.269	0.955
ENST00000470280	LDHB	2.64	-4.53	1.70	-2.67	7.49E-03	1.13E-01	77.08	-0.20	0.33	-0.60	0.545	1.000
ENST00000505328	LEF1	2.07	-4.19	1.32	-3.17	1.50E-03	3.30E-02	10.58	-0.35	0.85	-0.42	0.677	1.000
ENST00000308330	LEMD3	61.11	-0.72	0.24	-3.06	2.18E-03	4.46E-02	5515.52	-0.01	0.12	-0.08	0.935	1.000
ENST00000296388	LEPRE1	11.41	-21.52	3.03	-7.10	1.21E-12	1.13E-10	4044.24	0.48	0.29	1.64	0.102	0.727
ENST00000557725	LGMN	4.27	-5.23	1.75	-2.98	2.84E-03	5.49E-02	31.57	-0.22	0.53	-0.41	0.681	1.000
ENST00000340417	LIN54	23.32	-1.78	0.53	-3.36	7.89E-04	1.96E-02	132.14	0.57	0.61	0.95	0.342	0.990
ENST00000336233	LIPA	135.43	-2.39	0.87	-2.75	5.88E-03	9.53E-02	2959.75	0.32	0.49	0.65	0.519	1.000
ENST00000261366	LMNB1	44.96	-23.38	3.03	-7.72	1.13E-14	3.56E-12	602.51	-2.99	0.95	-3.14	0.002	0.056
ENST00000409549	LOXL3	202.60	-4.01	0.99	-4.03	5.64E-05	1.98E-03	11946.10	0.35	0.24	1.47	0.143	0.815
ENST00000470907	LOXL3	57.73	-7.12	2.53	-2.82	4.82E-03	8.20E-02	1784.27	0.74	0.28	2.68	0.007	0.168
ENST00000283415	LPCAT1	17.23	-7.24	2.62	-2.76	5.73E-03	9.31E-02	9670.06	-0.89	0.84	-1.07	0.286	0.963
ENST00000261596	LPIN2	13.01	-1.61	0.51	-3.19	1.44E-03	3.20E-02	4509.46	-0.10	0.12	-0.87	0.384	1.000
ENST00000311322	LPL	85.22	-0.74	0.26	-2.82	4.83E-03	8.21E-02	3043.47	-0.64	0.40	-1.59	0.112	0.756
ENST00000389798	LRCH1	19.01	-2.21	0.45	-4.88	1.07E-06	4.80E-05	291.34	0.16	2.56	0.06	0.952	1.000
ENST00000460646	LRRFIP2	3.29	-4.87	1.44	-3.38	7.29E-04	1.84E-02	1107.20	-0.11	0.19	-0.60	0.549	1.000
ENST00000433627	LSM14A	82.02	-0.92	0.30	-3.08	2.08E-03	4.29E-02	276.18	3.43	1.43	2.40	0.016	0.279
ENST00000470086	LSM2	2.29	-3.68	1.27	-2.89	3.89E-03	7.01E-02	172.78	0.32	0.56	0.57	0.568	1.000
ENST00000409987	LSM5	5.31	-5.54	1.85	-2.99	2.78E-03	5.40E-02	21.71	-0.91	0.72	-1.27	0.203	0.898
ENST00000468732	LUC7L	3.33	-4.87	1.76	-2.77	5.62E-03	9.17E-02	91.76	0.02	0.48	0.04	0.965	1.000
ENST00000498518	LUC7L2	28.51	-22.73	3.03	-7.51	5.90E-14	1.21E-11	351.62	-0.23	0.49	-0.48	0.631	1.000
ENST00000522528	LY6E	24.28	-7.15	1.72	-4.16	3.24E-05	1.21E-03	16.61	-0.17	0.65	-0.27	0.790	1.000
ENST00000343470	LYAR	70.70	-1.43	0.43	-3.32	8.89E-04	2.16E-02	2964.76	-0.17	0.17	-1.02	0.306	0.978
ENST00000374502	LYPLA2	15.05	-4.53	1.43	-3.16	1.56E-03	3.39E-02	1810.99	0.24	0.22	1.06	0.289	0.966
ENST00000463964	LYPLAL1	6.16	-20.68	3.03	-6.83	8.79E-12	5.21E-10	49.56	0.33	0.48	0.70	0.485	1.000
ENST00000400903	LZIC	23.62	-7.70	2.11	-3.64	2.72E-04	8.00E-03	30.08	0.11	0.58	0.20	0.845	1.000
ENST00000488540	LZIC	16.57	-7.18	2.76	-2.60	9.28E-03	1.31E-01	52.10	0.00	0.72	0.00	0.997	1.000
ENST00000440576	LZTFL1	16.14	-7.15	1.81	-3.95	7.82E-05	2.66E-03	100.72	0.91	0.72	1.25	0.211	0.907
ENST00000543834	M6PR	23.07	-2.47	0.86	-2.86	4.19E-03	7.41E-02	150.01	0.48	0.50	0.96	0.335	0.988
ENST00000538595	MACROD1	6.39	-20.73	3.03	-6.84	7.83E-12	4.75E-10	375.91	0.12	0.33	0.36	0.716	1.000
ENST00000296509	MAD2L1	335.44	-1.08	0.27	-4.04	5.44E-05	1.92E-03	4065.74	-0.23	0.14	-1.70	0.089	0.690
ENST00000322428	MAF1	15.12	-7.05	2.67	-2.64	8.22E-03	1.20E-01	3015.01	-0.13	0.41	-0.33	0.740	1.000
ENST00000396224	MAGED2	13.20	-21.72	3.03	-7.17	7.45E-13	7.81E-11	246.16	-0.07	1.65	-0.04	0.967	1.000
ENST00000518389	MAK16	19.63	-22.25	3.03	-7.35	1.98E-13	2.90E-11	74.05	-0.48	0.50	-0.96	0.336	0.989
ENST00000261483	MAN2A1	78.09	-1.09	0.30	-3.69	2.27E-04	6.80E-03	4221.88	0.10	0.13	0.82	0.412	1.000
ENST00000397150	MANBAL	7.05	-1.56	0.59	-2.63	8.64E-03	1.24E-01	198.26	0.19	0.28	0.70	0.482	1.000
ENST00000447185	MAP2	37.34	-23.12	3.03	-7.64	2.22E-14	6.05E-12	2755.69	-0.68	0.41	-1.66	0.097	0.717
ENST00000425818	MAP2K1	7.76	-21.00	3.03	-6.93	4.18E-12	2.83E-10	59.28	-1.09	1.11	-0.98	0.326	0.985
ENST00000415385	MAP2K4	63.35	-0.59	0.22	-2.69	7.14E-03	1.09E-01	1.13	-3.68	4.11	-0.90	0.370	NA
ENST00000392142	MAP3K4	46.13	-0.93	0.28	-3.25	1.14E-03	2.65E-02	281.88	0.27	0.37	0.73	0.467	1.000
ENST00000369327	MAP3K7	16.33	-7.16	2.66	-2.69	7.08E-03	1.08E-01	2757.48	0.03	0.13	0.25	0.806	1.000
ENST00000369332	MAP3K7	46.06	-3.39	1.07	-3.17	1.53E-03	3.36E-02	578.50	2.13	0.92	2.30	0.021	0.330
ENST00000479630	MAP3K7	19.57	-5.87	1.87	-3.14	1.68E-03	3.63E-02	786.42	0.92	0.67	1.36	0.173	0.862
ENST00000360240	MAP4	25.94	-22.28	3.03	-7.36	1.84E-13	2.74E-11	1461.55	1.15	0.96	1.19	0.233	0.928
ENST00000498066	MAP4K4	37.12	-1.22	0.38	-3.24	1.18E-03	2.72E-02	582.65	0.18	0.35	0.52	0.604	1.000

ENST00000496250	MAPK14	23.34	-7.68	2.66	-2.88	3.92E-03	7.04E-02	74.67	0.41	1.19	0.34	0.732	1.000
ENST00000420397	MARCH7	10.54	-6.53	1.75	-3.74	1.83E-04	5.66E-03	26.08	-2.61	1.96	-1.33	0.184	0.877
ENST00000262027	MARS	42.28	-7.14	2.77	-2.58	9.95E-03	1.37E-01	3406.45	-0.05	0.59	-0.09	0.929	1.000
ENST00000547665	MARS	3.63	-3.72	1.31	-2.84	4.52E-03	7.80E-02	302.12	-0.21	0.26	-0.83	0.405	1.000
ENST00000318473	MBIP	11.52	-6.66	1.91	-3.49	4.78E-04	1.29E-02	134.87	0.05	0.42	0.11	0.914	1.000
ENST00000553298	MBIP	7.63	-20.97	3.03	-6.92	4.47E-12	2.98E-10	35.22	-0.10	0.88	-0.11	0.912	1.000
ENST00000370839	MBNL3	1.55	-3.75	1.30	-2.89	3.83E-03	6.94E-02	10.12	-1.34	2.23	-0.60	0.550	1.000
ENST00000394311	MBNL3	4.44	-5.29	1.36	-3.90	9.45E-05	3.15E-03	3220.39	-4.67	1.88	-2.49	0.013	0.240
ENST00000340941	MCCC2	54.22	-1.87	0.65	-2.88	4.03E-03	7.19E-02	5062.90	-0.97	1.60	-0.61	0.544	1.000
ENST00000361282	MCM10	123.88	-10.09	1.01	-9.95	2.59E-23	6.80E-20	1753.89	-1.28	0.27	-4.73	0.000	0.000
ENST00000419835	MCM3	90.86	-2.50	0.86	-2.92	3.50E-03	6.48E-02	5734.00	0.24	0.19	1.28	0.200	0.895
ENST00000519138	MCM4	216.27	-10.89	1.06	-10.26	1.03E-24	3.93E-21	110.63	-0.41	0.35	-1.17	0.240	0.934
ENST00000519170	MCM4	54.66	-8.91	2.80	-3.19	1.44E-03	3.20E-02	48.26	-0.46	0.51	-0.90	0.369	1.000
ENST00000264156	MCM6	1042.96	-0.78	0.25	-3.09	2.01E-03	4.20E-02	21407.56	-0.14	0.13	-1.10	0.271	0.957
ENST00000343023	MCM7	14.00	-21.79	3.03	-7.20	6.13E-13	6.95E-11	737.26	-0.40	0.35	-1.14	0.253	0.945
ENST00000360003	MCMBP	283.42	-0.68	0.21	-3.21	1.31E-03	2.96E-02	9016.12	-0.17	0.18	-0.96	0.339	0.990
ENST00000548334	MCRS1	7.61	-20.94	3.03	-6.91	4.81E-12	3.14E-10	126.32	0.05	0.48	0.10	0.921	1.000
ENST00000371315	MCTS1	20.26	-3.49	1.33	-2.63	8.52E-03	1.23E-01	651.80	1.69	0.95	1.78	0.075	0.638
ENST00000371317	MCTS1	63.00	-1.31	0.41	-3.21	1.32E-03	2.98E-02	3525.42	-0.11	0.39	-0.28	0.783	1.000
ENST00000490105	MDH2	20.83	-4.99	0.95	-5.27	1.37E-07	6.62E-06	107.12	0.09	0.34	0.26	0.793	1.000
ENST00000367182	MDM4	13.74	-1.28	0.39	-3.32	9.13E-04	2.20E-02	15748.96	-0.11	0.18	-0.59	0.558	1.000
ENST00000462012	MDM4	2.19	-4.25	1.13	-3.77	1.65E-04	5.13E-03	262.96	-0.06	0.22	-0.26	0.795	1.000
ENST00000369393	MDN1	176.54	-0.63	0.18	-3.44	5.90E-04	1.54E-02	33026.81	-0.05	0.12	-0.38	0.706	1.000
ENST00000373073	MEAF6	5.61	-20.56	3.03	-6.78	1.18E-11	6.66E-10	23.38	-0.77	0.66	-1.16	0.245	0.937
ENST00000464511	MECR	2.89	-4.65	1.17	-3.97	7.07E-05	2.44E-03	77.80	-0.27	0.53	-0.50	0.618	1.000
ENST00000255764	MED10	81.46	-0.80	0.21	-3.89	1.02E-04	3.34E-03	2026.09	-0.08	0.16	-0.54	0.589	1.000
ENST00000479574	MED18	8.18	-21.07	3.03	-6.96	3.51E-12	2.51E-10	82.80	0.75	0.35	2.14	0.032	0.417
ENST00000534677	MED19	3.26	-4.84	1.43	-3.37	7.49E-04	1.87E-02	33.63	-0.55	0.49	-1.12	0.261	0.951
ENST00000378594	MED4	13.57	-6.90	2.10	-3.29	1.02E-03	2.41E-02	2103.80	-0.12	0.43	-0.27	0.786	1.000
ENST00000417167	MED4	16.62	-4.27	1.61	-2.65	8.08E-03	1.19E-01	84.52	-0.21	0.37	-0.56	0.577	1.000
ENST00000411432	METTL21A	2.42	-3.09	1.19	-2.60	9.36E-03	1.31E-01	94.25	0.58	0.38	1.54	0.124	0.782
ENST00000392640	METTL5	7.46	-20.91	3.03	-6.90	5.16E-12	3.34E-10	338.00	0.39	0.35	1.11	0.266	0.953
ENST00000383789	METTL6	7.05	-20.84	3.03	-6.88	6.11E-12	3.82E-10	469.08	-0.48	0.53	-0.92	0.358	0.998
ENST00000490857	MFF	13.46	-6.88	1.86	-3.70	2.13E-04	6.42E-03	1142.44	-0.32	0.15	-2.12	0.034	0.429
ENST00000566497	MFGE8	17.88	-22.11	3.03	-7.30	2.81E-13	3.81E-11	6215.91	0.47	0.28	1.67	0.095	0.711
ENST00000539261	MGP	36.33	-1.63	0.40	-4.06	4.87E-05	1.74E-03	2570.97	-0.58	0.26	-2.18	0.029	0.394
ENST00000344507	MIA3	11.62	-6.67	2.02	-3.30	9.62E-04	2.30E-02	1212.49	0.74	0.41	1.81	0.071	0.621
ENST00000467029	MINOS1	18.10	-2.33	0.70	-3.33	8.57E-04	2.09E-02	84.18	0.02	0.51	0.04	0.968	1.000
ENST00000368653	MKI67	33.86	-22.39	3.03	-7.40	1.40E-13	2.18E-11	439.54	-0.59	0.67	-0.89	0.375	1.000
ENST00000537609	MKI67	7.28	-20.89	3.03	-6.89	5.42E-12	3.49E-10	17019.98	-0.06	0.30	-0.20	0.845	1.000
ENST00000447132	MKI67IP	14.05	-21.80	3.03	-7.20	6.06E-13	6.91E-11	921.31	-0.55	0.19	-2.89	0.004	0.106
ENST00000399054	MKKS	122.84	-0.64	0.20	-3.17	1.53E-03	3.35E-02	8.40	0.17	0.95	0.17	0.862	1.000
ENST00000341243	MKL2	8.40	-3.45	1.10	-3.13	1.73E-03	3.71E-02	784.78	-0.05	0.25	-0.18	0.856	1.000
ENST00000486716	MKNK1	7.85	-3.76	1.03	-3.65	2.67E-04	7.86E-03	9.23	-0.16	1.01	-0.16	0.873	1.000
ENST00000411987	MKRN2	32.49	-3.44	0.77	-4.49	7.22E-06	2.97E-04	38.99	0.32	1.12	0.28	0.776	1.000
ENST00000508095	MLF1IP	13.16	-6.85	1.03	-6.65	2.93E-11	1.60E-09	42.38	0.23	0.72	0.32	0.753	1.000
ENST00000305963	MMGT1	39.13	-1.06	0.29	-3.62	2.89E-04	8.41E-03	2848.30	0.12	0.32	0.37	0.710	1.000
ENST00000520568	MMP16	8.12	-6.16	2.06	-2.99	2.78E-03	5.40E-02	71.67	-0.47	0.37	-1.28	0.201	0.897
ENST00000543485	MMP2	88.53	-7.26	2.00	-3.64	2.76E-04	8.08E-03	84317.61	0.25	0.25	0.99	0.320	0.983
ENST00000275053	MMS22L	54.51	-0.92	0.32	-2.91	3.57E-03	6.58E-02	1842.26	-0.15	0.20	-0.75	0.452	1.000
ENST00000409916	MOB4	6.28	-20.71	3.03	-6.83	8.22E-12	4.94E-10	29.14	-0.26	0.56	-0.46	0.645	1.000
ENST00000380492	MOSPD2	33.71	-1.14	0.44	-2.59	9.51E-03	1.32E-01	2646.27	0.17	0.14	1.17	0.243	0.936
ENST00000319217	MPDZ	78.50	-1.05	0.40	-2.61	8.98E-03	1.28E-01	87.07	0.34	0.45	0.75	0.453	1.000
ENST00000498451	MPHOSPH10	32.08	-8.14	1.92	-4.23	2.30E-05	8.89E-04	1213.96	0.04	0.16	0.23	0.817	1.000
ENST00000541076	MPHOSPH9	37.70	-1.00	0.33	-2.98	2.89E-03	5.58E-02	3852.21	0.21	0.15	1.45	0.147	0.822
ENST00000567570	MPI	9.56	-5.22	1.93	-2.70	6.95E-03	1.07E-01	38.25	-0.05	0.55	-0.10	0.921	1.000
ENST00000491955	MPP1	5.55	-1.94	0.72	-2.71	6.81E-03	1.05E-01	625.89	-0.37	0.94	-0.39	0.695	1.000
ENST00000414263	MPRIP	5.22	-20.44	3.03	-6.74	1.53E-11	8.53E-10	1362.22	-0.21	0.23	-0.91	0.363	0.999

ENST00000233545	MPV17	19.59	-2.55	0.50	-5.14	2.75E-07	1.29E-05	110.67	0.65	0.75	0.86	0.389	1.000
ENST00000380044	MPV17	15.15	-6.47	1.28	-5.05	4.31E-07	1.99E-05	845.89	0.53	1.01	0.52	0.601	1.000
ENST00000430991	MPV17	4.78	-3.55	1.31	-2.70	6.84E-03	1.06E-01	23.28	-0.95	0.60	-1.57	0.117	0.766
ENST00000491222	MPZ	6.88	-20.79	3.03	-6.86	6.81E-12	4.20E-10	749.48	0.18	0.53	0.34	0.730	1.000
ENST00000315567	MRPL1	48.58	-0.79	0.26	-3.07	2.11E-03	4.36E-02	1194.14	0.13	0.38	0.34	0.736	1.000
ENST00000332396	MRPL12	88.74	-9.61	2.90	-3.31	9.22E-04	2.22E-02	12172.18	-0.18	0.16	-1.13	0.258	0.949
ENST00000545862	MRPL12	16.59	-7.19	2.73	-2.63	8.42E-03	1.22E-01	989.21	0.97	1.29	0.75	0.451	1.000
ENST00000522717	MRPL13	16.93	-7.22	2.67	-2.70	6.91E-03	1.06E-01	2.32	1.76	2.09	0.84	0.400	NA
ENST00000529958	MRPL17	24.77	-1.39	0.48	-2.92	3.53E-03	6.53E-02	458.74	0.12	0.32	0.37	0.712	1.000
ENST00000362034	MRPL21	140.20	-1.66	0.40	-4.14	3.54E-05	1.31E-03	2478.23	-0.10	0.36	-0.29	0.775	1.000
ENST00000361531	MRPL24	149.42	-0.94	0.29	-3.22	1.28E-03	2.91E-02	3632.25	0.15	0.14	1.09	0.275	0.959
ENST00000505059	MRPL36	59.62	-9.03	2.87	-3.15	1.64E-03	3.55E-02	2746.95	0.36	0.30	1.19	0.236	0.929
ENST00000352957	MRPL39	9.76	-21.31	3.03	-7.03	2.00E-12	1.64E-10	1127.08	-0.33	0.25	-1.34	0.181	0.873
ENST00000558660	MRPL46.1	49.87	-1.58	0.49	-3.24	1.18E-03	2.72E-02	322.35	0.05	0.25	0.21	0.838	1.000
ENST00000553965	MRPL52	51.94	-1.90	0.44	-4.27	1.97E-05	7.70E-04	253.44	0.22	0.21	1.08	0.281	0.962
ENST00000053468	MRPS10	68.35	-1.17	0.29	-3.98	6.79E-05	2.35E-03	3877.58	0.04	0.18	0.24	0.807	1.000
ENST00000259873	MRPS18B	21.61	-7.57	2.66	-2.84	4.49E-03	7.78E-02	4600.69	0.33	0.18	1.82	0.069	0.612
ENST00000492644	MRPS22	6.80	-5.30	1.58	-3.36	7.75E-04	1.93E-02	127.12	0.23	0.38	0.62	0.537	1.000
ENST00000261413	MRPS27	84.44	-2.80	1.00	-2.80	5.19E-03	8.70E-02	2501.20	-0.09	0.24	-0.36	0.720	1.000
ENST00000323563	MRPS31	24.17	-1.31	0.49	-2.66	7.87E-03	1.17E-01	1918.44	-0.03	0.20	-0.15	0.884	1.000
ENST00000081029	MRPS35	113.94	-1.08	0.22	-4.98	6.46E-07	2.95E-05	1920.09	0.11	0.15	0.78	0.437	1.000
ENST00000538315	MRPS35	7.00	-20.83	3.03	-6.87	6.22E-12	3.88E-10	91.15	1.11	1.27	0.87	0.382	1.000
ENST00000489572	MRRF	5.41	-20.49	3.03	-6.76	1.38E-11	7.68E-10	1191.27	0.27	0.20	1.36	0.175	0.864
ENST00000406134	MSH2	320.18	-0.58	0.14	-4.05	5.07E-05	1.80E-03	150.35	-0.98	0.67	-1.47	0.142	0.815
ENST00000437063	MTAPP2	31.01	-0.70	0.27	-2.61	9.06E-03	1.29E-01	19.68	0.11	0.86	0.12	0.901	1.000
ENST00000305949	MTBP	35.59	-0.99	0.30	-3.31	9.41E-04	2.26E-02	2226.13	-0.13	0.18	-0.73	0.465	1.000
ENST00000523373	MTBP	4.14	-5.19	1.79	-2.89	3.81E-03	6.90E-02	3.58	2.56	2.08	1.23	0.219	NA
ENST00000542981	MTCH2	19.68	-7.43	2.70	-2.75	5.90E-03	9.54E-02	97.63	-0.10	0.46	-0.21	0.832	1.000
ENST00000361739	MT-CO2	1373.16	-0.61	0.19	-3.27	1.08E-03	2.53E-02	1082086.96	0.06	0.14	0.42	0.675	1.000
ENST00000362079	MT-CO3	1560.29	-0.57	0.15	-3.77	1.65E-04	5.13E-03	747419.66	0.14	0.11	1.25	0.213	0.909
ENST00000555709	MTHFD1	876.24	-0.78	0.19	-4.12	3.86E-05	1.41E-03	5191.69	-0.53	0.40	-1.33	0.183	0.877
ENST00000462026	MTHFD2	10.71	-21.43	3.03	-7.08	1.48E-12	1.32E-10	44.12	-0.26	0.87	-0.30	0.764	1.000
ENST00000370396	MTM1	28.07	-1.73	0.37	-4.63	3.59E-06	1.53E-04	684.78	-0.12	0.27	-0.43	0.667	1.000
ENST00000457540	MTND2P28	13.10	-2.48	0.69	-3.62	2.93E-04	8.51E-03	58137.85	0.20	0.12	1.69	0.091	0.697
ENST00000358107	MTPAP	50.48	-1.70	0.46	-3.66	2.53E-04	7.49E-03	633.77	-0.31	0.18	-1.73	0.083	0.666
ENST00000308110	MUS81	19.35	-2.32	0.88	-2.63	8.59E-03	1.24E-01	2648.17	0.00	0.21	-0.02	0.986	1.000
ENST00000450313	MUTYH	3.83	-3.34	1.20	-2.79	5.25E-03	8.77E-02	9.35	-0.64	1.02	-0.63	0.530	1.000
ENST00000237500	MYL12B	150.59	-4.77	1.82	-2.62	8.91E-03	1.27E-01	12859.91	0.10	0.15	0.67	0.500	1.000
ENST00000293422	MYL6	7.15	-20.88	3.03	-6.89	5.47E-12	3.50E-10	90.48	0.16	0.36	0.44	0.659	1.000
ENST00000536128	MYL6	26.61	-1.79	0.69	-2.58	9.83E-03	1.36E-01	35.85	0.39	0.55	0.71	0.479	1.000
ENST00000552297	MYL6	8.16	-4.98	1.55	-3.21	1.32E-03	2.97E-02	22.66	-0.37	1.07	-0.34	0.731	1.000
ENST00000356716	MYNN	26.76	-1.31	0.46	-2.83	4.59E-03	7.90E-02	1412.28	0.10	0.14	0.75	0.456	1.000
ENST00000428345	MYO6	92.43	-0.72	0.20	-3.56	3.71E-04	1.04E-02	482.48	2.01	1.89	1.06	0.289	0.966
ENST00000359263	MYOF	53.74	-3.52	1.16	-3.04	2.33E-03	4.71E-02	2531.46	0.97	0.62	1.56	0.119	0.773
ENST00000472487	MYSM1	16.63	-1.55	0.46	-3.39	7.10E-04	1.80E-02	702.67	0.19	0.26	0.74	0.461	1.000
ENST00000493821	MYSM1	28.27	-0.99	0.34	-2.94	3.24E-03	6.09E-02	15657.32	0.05	0.14	0.34	0.731	1.000
ENST00000262384	N4BP1	26.92	-0.84	0.32	-2.62	8.74E-03	1.25E-01	5071.19	0.00	0.13	-0.02	0.981	1.000
ENST00000511480	N4BP2	27.63	-1.19	0.31	-3.81	1.37E-04	4.34E-03	1350.84	-0.17	0.71	-0.24	0.808	1.000
ENST00000267068	N4BP2L2	10.01	-4.24	1.60	-2.65	7.96E-03	1.18E-01	934.39	-1.28	0.78	-1.65	0.099	0.720
ENST00000512755	N4BP2L2	22.17	-1.26	0.48	-2.61	8.98E-03	1.28E-01	2027.68	0.08	0.30	0.28	0.782	1.000
ENST00000480277	NAA15	14.17	-21.81	3.03	-7.20	5.87E-13	6.73E-11	450.28	0.24	1.89	0.13	0.898	1.000
ENST00000361671	NAA35	40.08	-2.00	0.65	-3.08	2.05E-03	4.25E-02	3333.57	0.13	0.13	1.06	0.290	0.966
ENST00000547914	NACA	405.36	-0.84	0.19	-4.54	5.54E-06	2.32E-04	2117.77	-1.15	0.57	-2.02	0.043	0.487
ENST00000552055	NACA	227.19	-1.34	0.48	-2.77	5.65E-03	9.21E-02	1140.78	-0.74	0.40	-1.87	0.062	0.583
ENST00000359087	NAE1	19.62	-7.43	2.88	-2.58	9.85E-03	1.36E-01	27.27	-0.53	1.25	-0.43	0.671	1.000
ENST00000463871	NAMPT	2.84	-4.64	1.68	-2.77	5.67E-03	9.24E-02	120.13	-0.26	0.34	-0.76	0.449	1.000
ENST00000431879	NAP1L1	12.91	-5.63	1.66	-3.40	6.75E-04	1.72E-02	6.80	0.53	1.33	0.40	0.689	1.000
ENST00000552056	NAP1L1	50.08	-23.52	3.03	-7.77	7.75E-15	2.60E-12	2153.79	-0.49	0.36	-1.36	0.175	0.864

ENST00000529361	NAP1L4	13.14	-21.71	3.03	-7.17	7.54E-13	7.87E-11	21.20	1.11	1.12	0.99	0.322	0.983
ENST00000390006	NARF	13.32	-6.87	1.80	-3.81	1.40E-04	4.42E-03	3451.45	0.75	1.79	0.42	0.677	1.000
ENST00000437901	NASP	16.37	-22.01	3.03	-7.27	3.61E-13	4.63E-11	593.99	-0.05	0.58	-0.08	0.934	1.000
ENST00000481782	NASP	38.96	-3.35	0.86	-3.89	9.82E-05	3.26E-03	7236.36	0.08	0.26	0.30	0.768	1.000
ENST00000530073	NASP	48.76	-4.00	0.99	-4.04	5.26E-05	1.87E-03	4296.89	-0.09	0.18	-0.49	0.627	1.000
ENST00000396252	NBN	159.97	-1.04	0.27	-3.89	1.00E-04	3.31E-03	41.84	2.10	1.32	1.59	0.112	0.757
ENST00000542492	NCAPD2	29.16	-8.00	2.76	-2.90	3.78E-03	6.85E-02	778.60	0.95	0.64	1.49	0.137	0.805
ENST00000525432	NCAPD3	4.93	-5.44	1.71	-3.18	1.48E-03	3.26E-02	76.50	0.34	0.39	0.86	0.391	1.000
ENST00000477409	NCAPH	44.08	-23.35	3.03	-7.71	1.22E-14	3.80E-12	232.50	-0.22	0.54	-0.41	0.681	1.000
ENST00000417652	NCL	8.96	-21.19	3.03	-7.00	2.62E-12	2.03E-10	47.54	0.14	0.56	0.24	0.807	1.000
ENST00000374087	NCOA4	21.99	-22.32	3.03	-7.37	1.67E-13	2.53E-11	489.76	0.05	0.26	0.17	0.862	1.000
ENST00000290231	NCOA5	150.87	-0.71	0.22	-3.16	1.55E-03	3.39E-02	4798.38	-0.12	0.12	-0.97	0.334	0.987
ENST00000555026	NDRG2	2.64	-4.54	1.74	-2.61	8.93E-03	1.27E-01	2.97	-0.22	2.43	-0.09	0.929	NA
ENST00000339600	NDUFA4	245.00	-8.62	1.27	-6.81	9.73E-12	5.68E-10	19726.06	0.09	0.13	0.68	0.497	1.000
ENST00000482299	NDUFA4	71.01	-6.57	1.18	-5.55	2.81E-08	1.40E-06	2921.49	-0.09	0.23	-0.40	0.687	1.000
ENST00000567761	NDUFAB1	29.93	-22.81	3.03	-7.54	4.81E-14	1.05E-11	84.90	-0.16	0.45	-0.36	0.716	1.000
ENST00000569148	NDUFB10	5.73	-20.59	3.03	-6.79	1.10E-11	6.29E-10	157.77	-0.54	0.73	-0.73	0.464	1.000
ENST00000259037	NDUFB5	27.00	-7.89	1.86	-4.25	2.12E-05	8.23E-04	2055.79	0.11	0.14	0.77	0.444	1.000
ENST00000522532	NDUFB9	9.20	-21.23	3.03	-7.01	2.41E-12	1.90E-10	287.06	-0.05	0.88	-0.06	0.955	1.000
ENST00000423725	NDUFS1	72.54	-2.71	0.91	-2.99	2.83E-03	5.47E-02	219.45	-0.09	0.81	-0.11	0.910	1.000
ENST00000372967	NDUFS5	353.32	-1.36	0.38	-3.53	4.08E-04	1.12E-02	6732.09	0.07	0.14	0.50	0.616	1.000
ENST00000529867	NDUFV1	10.03	-21.34	3.03	-7.05	1.84E-12	1.54E-10	17.25	0.14	0.70	0.21	0.836	1.000
ENST00000529927	NDUFV1	10.76	-21.42	3.03	-7.07	1.53E-12	1.35E-10	104.19	-0.02	0.47	-0.05	0.960	1.000
ENST00000266742	NEDD1	31.47	-3.10	0.59	-5.22	1.83E-07	8.73E-06	305.22	-0.66	0.54	-1.23	0.219	0.915
ENST00000530579	NEDD8-MDP1	79.81	-9.45	2.84	-3.33	8.83E-04	2.15E-02	26.53	1.38	2.51	0.55	0.583	1.000
ENST00000366998	NEK2	36.37	-23.09	3.03	-7.63	2.37E-14	6.24E-12	2287.83	0.09	0.35	0.26	0.795	1.000
ENST00000367385	NEK7	25.68	-1.83	0.36	-5.05	4.43E-07	2.04E-05	5671.46	0.14	0.16	0.90	0.366	1.000
ENST00000238616	NEK9	25.47	-0.99	0.37	-2.65	7.99E-03	1.18E-01	1570.74	-0.26	0.33	-0.78	0.438	1.000
ENST00000479589	NENF	40.82	-8.49	1.94	-4.38	1.21E-05	4.85E-04	694.63	-0.27	0.25	-1.09	0.275	0.959
ENST00000361676	NF2	8.94	-21.19	3.03	-6.99	2.66E-12	2.05E-10	40.50	-0.38	2.34	-0.16	0.872	1.000
ENST00000396009	NFATC2	16.20	-3.75	1.09	-3.44	5.76E-04	1.51E-02	41250.10	-0.05	0.18	-0.26	0.798	1.000
ENST00000504044	NFKB1	7.52	-6.04	1.78	-3.39	6.93E-04	1.76E-02	51.89	-0.46	0.44	-1.04	0.300	0.972
ENST00000462320	NFU1	39.10	-8.43	0.98	-8.60	7.87E-18	1.06E-14	200.82	-0.08	1.04	-0.08	0.940	1.000
ENST00000555128	NGDN	11.55	-6.67	2.36	-2.82	4.74E-03	8.08E-02	47.90	-1.14	1.17	-0.97	0.332	0.987
ENST00000556022	NGDN	16.65	-7.20	1.06	-6.82	9.39E-12	5.51E-10	699.60	-0.17	0.22	-0.75	0.453	1.000
ENST00000280700	NGLY1	6.92	-4.09	1.04	-3.93	8.50E-05	2.87E-03	52.50	-0.55	0.93	-0.59	0.554	1.000
ENST00000402458	NHP2L1	21.74	-7.58	1.81	-4.18	2.96E-05	1.11E-03	71.13	0.44	0.47	0.93	0.351	0.994
ENST00000366595	NID1	32.18	-22.92	3.03	-7.57	3.65E-14	8.62E-12	7431.56	-0.11	0.43	-0.25	0.805	1.000
ENST00000409588	NIF3L1	8.14	-21.06	3.03	-6.95	3.57E-12	2.54E-10	22.57	0.27	0.68	0.40	0.692	1.000
ENST00000368009	NIT1	11.05	-21.47	3.03	-7.09	1.35E-12	1.23E-10	673.97	-0.01	0.24	-0.06	0.954	1.000
ENST00000472258	NKTR	2.61	-4.52	1.08	-4.19	2.81E-05	1.06E-03	20.71	0.89	0.81	1.11	0.268	0.955
ENST00000514264	NME2	29.55	-4.05	1.38	-2.93	3.40E-03	6.34E-02	333.90	1.62	0.99	1.64	0.101	0.727
ENST00000344920	NNT	14.41	-21.82	3.03	-7.21	5.73E-13	6.60E-11	9630.22	0.37	0.47	0.79	0.427	1.000
ENST00000564620	NOB1	15.03	-6.45	1.89	-3.41	6.57E-04	1.69E-02	498.68	1.16	0.60	1.95	0.051	0.533
ENST00000543788	NOC3L	7.76	-21.00	3.03	-6.93	4.18E-12	2.83E-10	34.78	-0.63	0.86	-0.73	0.467	1.000
ENST00000345985	NOL10	140.66	-0.93	0.25	-3.70	2.16E-04	6.52E-03	2333.19	1.20	0.20	5.91	0.000	0.000
ENST00000253247	NOL11	302.25	-1.38	0.15	-9.44	3.74E-21	8.26E-18	8453.88	-0.02	0.14	-0.13	0.897	1.000
ENST00000535137	NOL11	5.67	-20.57	3.03	-6.79	1.14E-11	6.50E-10	121.66	-0.69	3.92	-0.17	0.861	1.000
ENST00000451315	NOL7	100.98	-1.09	0.37	-2.96	3.10E-03	5.89E-02	3317.08	-0.06	0.15	-0.39	0.693	1.000
ENST00000569526	NOMO2	60.27	-1.92	0.53	-3.60	3.17E-04	9.08E-03	54.15	0.67	0.42	1.61	0.108	0.744
ENST00000373841	NONO	21.25	-21.56	3.03	-7.12	1.07E-12	1.02E-10	4434.95	-0.43	0.21	-2.07	0.038	0.457
ENST00000382421	NOP2	47.06	-6.96	1.95	-3.57	3.56E-04	1.00E-02	1268.42	0.33	0.18	1.87	0.062	0.582
ENST00000542919	NOP2	19.06	-7.39	2.70	-2.74	6.24E-03	9.93E-02	254.83	0.28	0.31	0.90	0.369	1.000
ENST00000381169	NOP56	94.93	-9.70	2.04	-4.77	1.88E-06	8.27E-05	45.47	0.83	1.23	0.67	0.500	1.000
ENST00000264279	NOP58	281.39	-0.71	0.14	-5.19	2.11E-07	1.00E-05	12099.82	-0.06	0.17	-0.37	0.713	1.000
ENST00000362074	NOTCH2NL	6.11	-20.68	3.03	-6.82	8.95E-12	5.28E-10	219.05	0.05	0.28	0.20	0.845	1.000
ENST00000335681	NPAS2	9.03	-4.04	1.46	-2.76	5.82E-03	9.45E-02	8963.94	0.07	0.20	0.34	0.735	1.000
ENST00000544660	NPEPPS	18.17	-22.14	3.03	-7.31	2.59E-13	3.58E-11	140.14	0.08	0.38	0.20	0.838	1.000

ENST00000351986	NPM1	544.64	-1.18	0.41	-2.86	4.20E-03	7.42E-02	17225.33	0.04	0.13	0.29	0.769		1.000
ENST00000463178	NPRL3	2.55	-4.49	1.65	-2.72	6.50E-03	1.02E-01	469.10	-0.10	0.27	-0.37	0.709		1.000
ENST00000379046	NQO1	67.48	-9.21	1.94	-4.76	1.97E-06	8.61E-05	290.96	1.13	0.63	1.80	0.072		0.624
ENST00000544883	NR2C2AP	7.17	-5.98	1.73	-3.46	5.48E-04	1.45E-02	322.65	-0.26	0.26	-0.99	0.324		0.984
ENST00000369535	NRAS	209.69	-0.72	0.19	-3.87	1.09E-04	3.57E-03	12265.15	0.06	0.14	0.40	0.686		1.000
ENST00000371665	NRD1	8.38	-21.10	3.03	-6.97	3.27E-12	2.37E-10	579.57	1.66	1.18	1.40	0.160		0.843
ENST00000355117	NRP2	9.47	-21.27	3.03	-7.02	2.20E-12	1.77E-10	95.01	-0.18	0.50	-0.36	0.720		1.000
ENST00000216879	NSFL1C	32.84	-7.00	1.78	-3.93	8.63E-05	2.91E-03	127.15	-0.26	0.52	-0.49	0.625		1.000
ENST00000361439	NSMCE1	12.41	-21.63	3.03	-7.14	9.10E-13	9.07E-11	1051.35	0.03	0.38	0.08	0.935		1.000
ENST00000522563	NSMCE2	21.38	-7.55	1.56	-4.84	1.29E-06	5.73E-05	521.85	0.70	0.51	1.36	0.174		0.863
ENST00000552185	NT5C2	5.26	-5.53	1.99	-2.77	5.55E-03	9.10E-02	71.82	0.21	0.43	0.48	0.632		1.000
ENST00000456458	NT5C3	15.51	-2.14	0.74	-2.89	3.87E-03	6.99E-02	667.85	-0.48	0.27	-1.77	0.076		0.642
ENST00000319550	NT5DC1	70.89	-0.78	0.28	-2.81	4.92E-03	8.32E-02	1634.92	0.14	0.26	0.56	0.573		1.000
ENST00000369651	NT5E	101.47	-4.68	1.79	-2.61	8.99E-03	1.28E-01	362.00	0.14	0.27	0.52	0.605		1.000
ENST00000483358	NUB1	7.68	-20.99	3.03	-6.93	4.30E-12	2.89E-10	19.26	3.14	1.24	2.53	0.011		0.223
ENST00000493588	NUB1	6.69	-20.76	3.03	-6.85	7.41E-12	4.52E-10	14.90	-0.60	0.70	-0.86	0.392		1.000
ENST00000458064	NUCB2	50.75	-23.54	3.03	-7.78	7.42E-15	2.55E-12	2584.06	0.08	0.12	0.63	0.529		1.000
ENST00000531242	NUCB2	32.36	-3.37	0.93	-3.63	2.83E-04	8.26E-03	33.93	-0.15	0.58	-0.25	0.799		1.000
ENST00000239690	NUDCD1	106.54	-0.61	0.20	-3.13	1.73E-03	3.69E-02	4241.08	-0.21	0.34	-0.61	0.539		1.000
ENST00000378927	NUDT5	28.61	-7.97	2.47	-3.22	1.26E-03	2.87E-02	609.38	0.39	0.36	1.07	0.283		0.962
ENST00000540453	NUP107	3.73	-5.04	1.64	-3.08	2.05E-03	4.25E-02	43.61	-0.61	0.85	-0.72	0.473		1.000
ENST00000403890	NUP43	14.24	-6.97	2.02	-3.44	5.78E-04	1.51E-02	56.31	0.42	0.95	0.44	0.661		1.000
ENST00000508465	NUP54	5.22	-5.52	1.82	-3.03	2.41E-03	4.84E-02	24.88	-0.06	0.81	-0.07	0.942		1.000
ENST00000560898	NUSAP1	3.04	-4.73	1.76	-2.69	7.22E-03	1.10E-01	45.18	0.23	0.58	0.41	0.684		1.000
ENST00000294172	NXF1	9.87	-21.32	3.03	-7.04	1.93E-12	1.60E-10	8.75	-0.36	1.70	-0.21	0.831		1.000
ENST00000439713	NXF1	8.37	-21.10	3.03	-6.97	3.28E-12	2.37E-10	14.16	-0.17	0.80	-0.21	0.832		1.000
ENST00000486557	ODF2L	3.62	-3.77	1.11	-3.41	6.55E-04	1.68E-02	11.05	-0.16	0.83	-0.19	0.849		1.000
ENST00000443864	OGDH	29.83	-4.41	1.69	-2.61	9.12E-03	1.29E-01	498.64	0.28	0.44	0.64	0.524		1.000
ENST00000444676	OGDH	8.70	-20.19	3.03	-6.67	2.63E-11	1.44E-09	6650.44	-0.86	2.33	-0.37	0.712		1.000
ENST00000373701	OGT	9.98	-21.34	3.03	-7.05	1.84E-12	1.54E-10	10759.84	0.24	0.22	1.07	0.284		0.962
ENST00000220514	OIP5	6.42	-2.00	0.77	-2.59	9.55E-03	1.33E-01	964.11	-0.20	0.15	-1.34	0.181		0.873
ENST00000234296	ORC2	24.18	-1.43	0.44	-3.20	1.35E-03	3.04E-02	5200.22	0.12	0.12	0.98	0.328		0.985
ENST00000440042	ORC4	6.13	-5.75	1.66	-3.46	5.49E-04	1.45E-02	7.17	0.09	2.04	0.04	0.966		1.000
ENST00000219097	ORC6	6.73	-20.80	3.03	-6.86	6.68E-12	4.14E-10	3.38	-0.09	1.38	-0.07	0.947	NA	
ENST00000315970	OS9	108.81	-2.04	0.69	-2.96	3.09E-03	5.88E-02	3584.59	0.43	0.26	1.66	0.097		0.715
ENST00000389142	OS9	10.18	-21.34	3.03	-7.05	1.83E-12	1.54E-10	141.12	0.29	0.87	0.34	0.737		1.000
ENST00000552285	OS9	16.69	-21.04	3.03	-6.95	3.70E-12	2.60E-10	11201.13	-0.15	0.57	-0.27	0.788		1.000
ENST00000513831	OSMR	12.43	-6.77	2.05	-3.30	9.57E-04	2.29E-02	323.95	0.19	0.26	0.74	0.458		1.000
ENST00000346234	OSTF1	29.05	-8.00	1.19	-6.70	2.10E-11	1.16E-09	1375.77	0.18	0.13	1.34	0.180		0.871
ENST00000543004	OTUB1	24.50	-22.55	3.03	-7.45	9.43E-14	1.64E-11	177.53	-0.07	0.52	-0.13	0.898		1.000
ENST00000375120	OTUD3	3.32	-4.24	1.57	-2.70	7.00E-03	1.07E-01	5108.61	-0.23	0.16	-1.43	0.152		0.828
ENST00000440381	P4HA1	51.28	-8.81	2.81	-3.14	1.72E-03	3.68E-02	1576.16	0.43	0.16	2.72	0.007		0.153
ENST00000550166	PA2G4	43.02	-2.11	0.61	-3.45	5.62E-04	1.48E-02	174.12	0.36	0.25	1.45	0.148		0.823
ENST00000310571	PAAF1	6.19	-20.69	3.03	-6.83	8.65E-12	5.16E-10	412.90	0.08	0.27	0.30	0.764		1.000
ENST00000520868	PABPC1	7.05	-5.95	1.69	-3.52	4.33E-04	1.18E-02	1836.83	-0.04	0.60	-0.07	0.943		1.000
ENST00000522387	PABPC1	47.79	-8.71	2.97	-2.93	3.34E-03	6.25E-02	28456.97	-0.26	0.28	-0.90	0.369		1.000
ENST00000523555	PABPC1	22.92	-22.46	3.03	-7.42	1.17E-13	1.91E-11	2426.11	0.93	1.34	0.70	0.487		1.000
ENST00000419197	PAFAH1B2	72.87	-24.03	3.03	-7.94	2.01E-15	1.03E-12	341.66	-0.10	0.24	-0.42	0.673		1.000
ENST00000338972	PAIP1	22.90	-7.65	2.83	-2.70	6.91E-03	1.06E-01	141.12	-1.52	0.30	-5.09	0.000		0.000
ENST00000436644	PAIP1	7.80	-21.01	3.03	-6.93	4.10E-12	2.79E-10	111.48	1.52	0.80	1.91	0.056		0.556
ENST00000510409	PAIP2	5.86	-20.61	3.03	-6.80	1.03E-11	5.98E-10	2884.01	0.56	0.24	2.33	0.020		0.314
ENST00000379568	PAK1IP1	127.43	-0.61	0.18	-3.39	6.93E-04	1.76E-02	3846.22	0.03	0.13	0.19	0.846		1.000
ENST00000379799	PAM	132.07	-3.65	0.76	-4.84	1.33E-06	5.90E-05	3649.70	0.21	0.15	1.46	0.143		0.816
ENST00000504691	PAM	7.07	-20.87	3.03	-6.89	5.69E-12	3.60E-10	1167.90	-0.05	0.18	-0.30	0.762		1.000
ENST00000238714	PAPOLG	22.97	-1.78	0.60	-2.97	2.97E-03	5.69E-02	353.33	1.47	0.66	2.23	0.026		0.371
ENST00000497113	PARK7	13.77	-21.77	3.03	-7.19	6.45E-13	7.12E-11	2456.42	0.00	0.21	0.00	0.997		1.000
ENST00000490921	PARP1	71.49	-2.98	1.07	-2.79	5.22E-03	8.72E-02	175.16	-0.06	0.27	-0.21	0.830		1.000
ENST00000397192	PAXIP1	6.88	-5.92	1.05	-5.63	1.81E-08	9.08E-07	186.69	0.15	0.82	0.19	0.851		1.000

ENST00000521226	PBK	6.45	-20.71	3.03	-6.83	8.29E-12	4.98E-10	81.79	0.00	0.34	-0.01	0.993	1.000
ENST00000524266	PBK	29.83	-22.82	3.03	-7.54	4.72E-14	1.03E-11	38.73	0.20	0.47	0.43	0.669	1.000
ENST00000462207	PBRM1	15.06	-7.05	1.87	-3.76	1.71E-04	5.29E-03	85.85	-0.32	0.45	-0.71	0.476	1.000
ENST00000549863	PCBP2	8.76	-4.49	1.53	-2.94	3.29E-03	6.18E-02	1654.04	0.05	0.29	0.17	0.866	1.000
ENST00000294192	PCBP4	7.61	-20.94	3.03	-6.91	4.81E-12	3.14E-10	29.94	-0.06	0.61	-0.09	0.925	1.000
ENST00000355852	PCBP4	6.21	-5.77	1.95	-2.95	3.16E-03	5.97E-02	50.50	-1.26	0.90	-1.40	0.162	0.845
ENST00000468777	PCCB	5.78	-20.60	3.03	-6.80	1.06E-11	6.11E-10	46.22	0.61	1.28	0.48	0.633	1.000
ENST00000336126	PCGF5	24.48	-4.03	1.28	-3.15	1.61E-03	3.50E-02	4780.17	0.14	0.19	0.74	0.459	1.000
ENST00000498274	PCNP	8.42	-2.11	0.74	-2.87	4.15E-03	7.34E-02	1203.26	0.31	0.26	1.19	0.235	0.929
ENST00000223061	PCOLCE	250.31	-0.75	0.27	-2.79	5.25E-03	8.76E-02	26155.27	-0.43	0.18	-2.33	0.020	0.316
ENST00000472348	PCOLCE	22.99	-3.76	1.21	-3.09	1.98E-03	4.14E-02	15723.42	-0.31	0.17	-1.85	0.064	0.593
ENST00000268896	PCTP	45.36	-1.29	0.48	-2.67	7.62E-03	1.14E-01	3891.74	0.04	0.45	0.09	0.931	1.000
ENST00000430755	PCYT1A	3.45	-3.67	1.14	-3.21	1.34E-03	3.00E-02	80.36	0.26	0.50	0.52	0.601	1.000
ENST00000473645	PDCD10	78.42	-8.85	2.11	-4.19	2.75E-05	1.05E-03	79.68	0.07	0.35	0.20	0.840	1.000
ENST00000280154	PDCD4	85.14	-24.16	3.03	-7.98	1.45E-15	8.09E-13	2741.74	0.00	0.98	0.00	0.999	1.000
ENST00000481353	PDCD4	25.92	-3.64	1.23	-2.96	3.10E-03	5.89E-02	1076.00	-0.02	0.60	-0.03	0.978	1.000
ENST00000511482	PDCD6	12.54	-5.59	1.30	-4.30	1.74E-05	6.82E-04	1495.83	0.05	0.18	0.27	0.784	1.000
ENST00000487821	PDCD6IP	7.27	-3.18	1.18	-2.70	6.98E-03	1.07E-01	341.64	0.22	0.27	0.84	0.400	1.000
ENST00000396182	PDE1C	33.81	-22.99	3.03	-7.60	3.06E-14	7.60E-12	348.52	-0.10	0.37	-0.26	0.796	1.000
ENST00000369351	PDE4DIP	8.90	-21.18	3.03	-6.99	2.69E-12	2.06E-10	454.63	-3.08	1.76	-1.74	0.081	0.661
ENST00000402802	PDGFA	6.52	-2.74	1.00	-2.74	6.15E-03	9.83E-02	3547.22	0.09	0.13	0.68	0.497	1.000
ENST00000479146	PDHA1	7.17	-3.31	1.05	-3.15	1.62E-03	3.50E-02	324.00	0.34	0.31	1.08	0.280	0.961
ENST00000430469	PDHX	5.54	-20.54	3.03	-6.78	1.22E-11	6.84E-10	49.68	1.12	1.05	1.07	0.285	0.963
ENST00000446523	PDIA3	56.11	-1.36	0.44	-3.10	1.92E-03	4.02E-02	1222.21	-0.58	0.85	-0.69	0.492	1.000
ENST00000379018	PDLIM4	27.66	-7.92	2.74	-2.89	3.85E-03	6.95E-02	9192.22	0.04	0.23	0.19	0.853	1.000
ENST00000297598	PDP1	34.64	-8.25	2.76	-2.99	2.82E-03	5.47E-02	1928.32	-0.72	1.01	-0.71	0.476	1.000
ENST00000517764	PDP1	35.85	-8.30	2.72	-3.05	2.30E-03	4.67E-02	2213.83	-0.26	0.16	-1.60	0.109	0.748
ENST00000376215	PDSS1	23.30	-5.08	1.22	-4.17	3.08E-05	1.15E-03	808.68	0.15	0.21	0.73	0.463	1.000
ENST00000468392	PDXK	7.99	-21.03	3.03	-6.94	3.88E-12	2.67E-10	114.20	-0.15	0.37	-0.39	0.693	1.000
ENST00000478502	PEF1	14.02	-21.79	3.03	-7.20	6.16E-13	6.95E-11	71.57	-0.48	0.56	-0.85	0.396	1.000
ENST00000482108	PEG10	123.71	-6.51	1.54	-4.22	2.44E-05	9.39E-04	22373.10	-0.05	0.15	-0.31	0.754	1.000
ENST00000335214	PES1	62.08	-22.92	3.03	-7.57	3.68E-14	8.62E-12	4924.41	-0.14	0.15	-0.93	0.352	0.994
ENST00000354694	PES1	39.69	-23.21	3.03	-7.67	1.76E-14	5.06E-12	2064.31	0.17	0.22	0.77	0.440	1.000
ENST00000514611	PFDN1	3.67	-5.01	1.72	-2.91	3.59E-03	6.61E-02	55.74	0.13	0.41	0.31	0.754	1.000
ENST00000549759	PFDN5	33.48	-22.21	3.03	-7.34	2.16E-13	3.08E-11	109.35	0.21	0.45	0.46	0.645	1.000
ENST00000550513	PFDN5	9.08	-3.76	1.30	-2.89	3.89E-03	7.00E-02	184.23	-0.08	0.31	-0.28	0.782	1.000
ENST00000546964	PFKM	27.95	-22.58	3.03	-7.46	8.63E-14	1.60E-11	2324.26	-0.17	1.60	-0.11	0.914	1.000
ENST00000381125	PFKP	105.37	-24.54	3.03	-8.11	5.17E-16	3.56E-13	10091.65	-0.16	0.15	-1.02	0.307	0.978
ENST00000421751	PFKP	11.72	-21.55	3.03	-7.12	1.12E-12	1.05E-10	151.44	-0.26	0.29	-0.89	0.375	1.000
ENST00000423691	PFN2	68.35	-2.51	0.55	-4.58	4.76E-06	2.00E-04	107.35	-0.22	0.37	-0.59	0.553	1.000
ENST00000409475	PGAP1	3.00	-4.73	1.40	-3.38	7.26E-04	1.83E-02	256.62	0.56	0.24	2.39	0.017	0.287
ENST00000371084	PGM1	208.42	-10.84	0.98	-11.11	1.08E-28	6.49E-25	6038.74	0.05	0.16	0.29	0.769	1.000
ENST00000507554	PGM3	38.06	-8.38	2.19	-3.84	1.25E-04	4.01E-03	10.26	-0.37	0.84	-0.44	0.662	1.000
ENST00000493669	PHACTR4	35.25	-3.58	1.21	-2.97	3.00E-03	5.73E-02	613.32	-0.77	0.60	-1.29	0.196	0.892
ENST00000399433	PHB2	20.87	-21.45	3.03	-7.09	1.38E-12	1.25E-10	39.60	0.01	0.59	0.02	0.988	1.000
ENST00000546217	PHB2	8.82	-5.68	1.64	-3.45	5.50E-04	1.46E-02	136.12	-0.07	0.34	-0.21	0.831	1.000
ENST00000312189	PHF19	22.97	-7.65	0.98	-7.80	5.99E-15	2.11E-12	1452.91	0.51	0.20	2.61	0.009	0.194
ENST00000339089	PHF20	5.61	-20.56	3.03	-6.78	1.18E-11	6.66E-10	382.45	0.59	1.03	0.57	0.570	1.000
ENST00000509876	PHF3	12.34	-21.63	3.03	-7.14	9.26E-13	9.16E-11	6883.84	-0.91	1.90	-0.48	0.631	1.000
ENST00000416404	PHF6	23.52	-22.45	3.03	-7.42	1.21E-13	1.95E-11	879.77	0.21	0.41	0.51	0.607	1.000
ENST00000369409	PHGDH	25.85	-22.63	3.03	-7.48	7.71E-14	1.46E-11	3341.63	0.18	0.72	0.24	0.807	1.000
ENST00000537497	PHGDH	5.83	-5.67	1.85	-3.06	2.20E-03	4.49E-02	115.79	1.39	1.10	1.27	0.205	0.900
ENST00000379942	PHKA2	8.74	-3.09	0.74	-4.18	2.89E-05	1.09E-03	3140.58	0.00	0.18	0.01	0.995	1.000
ENST00000326291	PIBF1	44.45	-0.88	0.29	-3.01	2.63E-03	5.19E-02	3380.92	0.05	0.14	0.35	0.724	1.000
ENST00000530692	PICALM	4.98	-4.83	1.53	-3.15	1.64E-03	3.55E-02	186.35	0.18	0.74	0.25	0.805	1.000
ENST00000533350	PICALM	3.31	-4.86	1.74	-2.79	5.31E-03	8.83E-02	22.53	0.14	0.83	0.17	0.865	1.000
ENST00000306465	PIGF	6.58	-3.68	0.97	-3.80	1.43E-04	4.50E-03	205.67	-0.19	0.38	-0.49	0.624	1.000
ENST00000341555	PIGT	14.81	-21.87	3.03	-7.22	5.10E-13	6.06E-11	17332.75	-0.10	0.18	-0.52	0.600	1.000

ENST00000486866	PIP5K1A	6.82	-3.25	1.09	-2.99	2.82E-03	5.47E-02	92.42	-0.46	0.43	-1.07	0.283	0.962
ENST00000436663	PITPNB	5.15	-5.50	2.02	-2.73	6.43E-03	1.01E-01	24.28	-1.04	0.65	-1.61	0.107	0.741
ENST00000361557	PJA2	72.16	-24.02	3.03	-7.94	2.08E-15	1.05E-12	4728.87	0.16	0.86	0.18	0.855	1.000
ENST00000237596	PKD2	50.49	-1.37	0.50	-2.73	6.39E-03	1.01E-01	9994.69	0.03	0.11	0.23	0.821	1.000
ENST00000418336	PKNOX1	20.05	-7.46	1.82	-4.10	4.13E-05	1.50E-03	3.00	0.53	1.92	0.28	0.783	NA
ENST00000303908	PLEKHB2	14.25	-21.82	3.03	-7.21	5.74E-13	6.60E-11	0.91	-3.39	4.85	-0.70	0.485	NA
ENST00000427836	PLEKHM3	7.56	-6.06	1.01	-5.99	2.07E-09	1.09E-07	1249.54	-0.01	0.17	-0.05	0.962	1.000
ENST00000503914	PLK4	3.10	-4.75	1.14	-4.15	3.26E-05	1.21E-03	18.78	-1.24	0.76	-1.64	0.100	0.722
ENST00000376369	PLOD1	13.29	-21.72	3.03	-7.17	7.27E-13	7.66E-11	17847.68	0.23	0.17	1.38	0.167	0.851
ENST00000469350	PLOD2	8.52	-21.12	3.03	-6.97	3.10E-12	2.28E-10	178.52	-7.60	2.09	-3.63	0.000	0.013
ENST00000421736	PLOD3	8.36	-21.10	3.03	-6.96	3.29E-12	2.38E-10	188.75	0.12	0.42	0.28	0.778	1.000
ENST00000465975	PLP1	42.47	-8.54	1.12	-7.59	3.08E-14	7.60E-12	1439.91	0.24	1.54	0.15	0.879	1.000
ENST00000457734	PLS1	6.12	-20.68	3.03	-6.82	8.95E-12	5.28E-10	507.36	-1.55	1.03	-1.50	0.133	0.799
ENST00000462666	PLSCR1	5.80	-4.47	1.13	-3.96	7.57E-05	2.59E-03	35.69	0.00	0.58	0.00	0.997	1.000
ENST00000275072	PM20D2	44.27	-1.61	0.49	-3.31	9.33E-04	2.24E-02	3462.73	-0.29	0.15	-1.91	0.056	0.556
ENST00000268261	PMM2	8.96	-6.29	1.51	-4.18	2.97E-05	1.11E-03	155.10	0.27	0.51	0.52	0.602	1.000
ENST00000395936	PMP22	6.06	-3.59	1.39	-2.59	9.65E-03	1.34E-01	628.84	1.99	1.05	1.89	0.059	0.568
ENST00000444457	PMPCB	49.06	-8.17	2.12	-3.85	1.20E-04	3.89E-03	888.54	-0.59	0.20	-2.93	0.003	0.096
ENST00000498530	PMPCB	88.77	-9.61	2.86	-3.36	7.83E-04	1.95E-02	1658.93	-0.04	0.38	-0.09	0.925	1.000
ENST00000361505	PNP	16.12	-7.14	1.10	-6.47	9.57E-11	5.17E-09	142.58	0.20	0.30	0.66	0.511	1.000
ENST00000534893	PNPO	10.03	-21.34	3.03	-7.05	1.84E-12	1.54E-10	48.45	0.13	0.52	0.24	0.810	1.000
ENST00000486717	POFUT1	26.94	-22.24	3.03	-7.35	2.03E-13	2.93E-11	106.27	-0.11	0.32	-0.35	0.730	1.000
ENST00000471540	POFUT2	3.63	-4.99	1.21	-4.14	3.49E-05	1.29E-03	1014.34	-1.50	1.23	-1.22	0.222	0.917
ENST00000527618	POLA2	40.66	-2.40	0.71	-3.39	7.03E-04	1.79E-02	284.29	0.08	0.34	0.24	0.811	1.000
ENST00000539111	POLG2	22.92	-0.99	0.32	-3.11	1.89E-03	3.98E-02	3951.99	0.00	0.15	0.00	0.996	1.000
ENST00000562953	POLR2C	10.38	-6.51	1.83	-3.55	3.82E-04	1.06E-02	101.03	-0.53	1.17	-0.46	0.648	1.000
ENST00000567982	POLR2C	5.41	-5.56	1.59	-3.51	4.54E-04	1.23E-02	159.30	0.04	0.39	0.11	0.913	1.000
ENST00000306433	POLR3D	13.92	-21.79	3.03	-7.20	6.24E-13	6.95E-11	167.59	-0.16	0.49	-0.33	0.742	1.000
ENST00000409832	POTEG	48.59	-2.00	0.67	-2.99	2.76E-03	5.38E-02	31.63	0.01	0.52	0.02	0.986	1.000
ENST00000499847	PPA2	8.64	-21.14	3.03	-6.98	2.96E-12	2.21E-10	188.55	-0.25	0.29	-0.86	0.387	1.000
ENST00000256678	PPHLN1	18.57	-3.45	1.17	-2.94	3.25E-03	6.10E-02	308.99	0.17	0.49	0.36	0.721	1.000
ENST00000551723	PPHLN1	3.83	-5.07	1.76	-2.88	3.93E-03	7.05E-02	19.93	-0.15	1.17	-0.13	0.899	1.000
ENST00000372835	PPIE	7.31	-6.00	1.91	-3.14	1.70E-03	3.65E-02	228.67	-0.16	0.31	-0.50	0.614	1.000
ENST00000448165	PPIF	16.25	-4.43	1.43	-3.10	1.90E-03	4.00E-02	683.10	-0.04	0.24	-0.19	0.852	1.000
ENST00000433207	PPIG	5.37	-5.56	1.88	-2.96	3.13E-03	5.92E-02	87.84	-0.67	0.52	-1.28	0.200	0.895
ENST00000321521	PPIP5K2	18.07	-6.73	1.58	-4.25	2.10E-05	8.16E-04	1273.80	-0.34	0.15	-2.29	0.022	0.339
ENST00000358506	PPP1CB	78.22	-24.13	3.03	-7.97	1.55E-15	8.55E-13	8460.89	0.35	0.74	0.48	0.634	1.000
ENST00000551191	PPP1R12A	6.17	-5.76	1.85	-3.12	1.82E-03	3.86E-02	9.80	0.06	0.89	0.07	0.943	1.000
ENST00000202556	PPP1R13B	12.33	-1.14	0.40	-2.83	4.60E-03	7.91E-02	362.21	-1.74	1.12	-1.55	0.121	0.777
ENST00000557217	PPP2R3C	60.54	-2.42	0.39	-6.19	6.03E-10	3.18E-08	1216.18	0.54	0.34	1.58	0.113	0.757
ENST00000455292	PPP2R4	12.50	-6.78	1.80	-3.77	1.62E-04	5.07E-03	218.80	0.44	0.42	1.05	0.292	0.967
ENST00000394853	PPP3CA	6.51	-20.72	3.03	-6.84	7.98E-12	4.81E-10	2316.30	0.18	1.16	0.15	0.880	1.000
ENST00000527193	PPP5C	16.56	-7.18	2.65	-2.71	6.77E-03	1.05E-01	31.90	1.94	0.64	3.02	0.003	0.077
ENST00000529172	PPP6R3	12.31	-21.62	3.03	-7.14	9.37E-13	9.16E-11	14.48	-0.20	0.80	-0.25	0.805	1.000
ENST00000531432	PPP6R3	17.40	-1.54	0.38	-4.06	4.92E-05	1.76E-03	171.53	-0.18	0.25	-0.74	0.457	1.000
ENST00000265462	PRDX5	813.95	-0.55	0.18	-3.04	2.35E-03	4.73E-02	25577.18	0.00	0.18	0.00	1.000	1.000
ENST00000425263	PREPL	22.94	-22.44	3.03	-7.41	1.25E-13	2.00E-11	90.62	-3.18	3.73	-0.85	0.394	1.000
ENST00000316299	PRKAG1	25.11	-6.61	2.43	-2.72	6.51E-03	1.02E-01	11.93	6.96	1.89	3.68	0.000	0.011
ENST00000395170	PRKAG1	31.38	-8.11	1.65	-4.92	8.53E-07	3.85E-05	3.58	2.54	1.93	1.31	0.190	NA
ENST00000392710	PRKAR1A	122.44	-3.65	1.28	-2.86	4.24E-03	7.46E-02	32697.23	-0.04	0.12	-0.35	0.724	1.000
ENST00000330452	PRKCD	25.92	-22.55	3.03	-7.45	9.43E-14	1.64E-11	17395.66	-0.19	0.39	-0.50	0.618	1.000
ENST00000303927	PRKCDBP	55.00	-2.64	0.89	-2.97	3.00E-03	5.73E-02	3401.25	-0.41	0.17	-2.38	0.017	0.291
ENST00000331968	PRKD1	58.76	-0.98	0.30	-3.32	9.09E-04	2.19E-02	3810.16	0.08	0.26	0.29	0.768	1.000
ENST00000331079	PRMT3	50.22	-0.76	0.25	-3.08	2.08E-03	4.30E-02	3104.73	-0.13	0.31	-0.43	0.669	1.000
ENST00000541371	PRPF19	14.85	-7.03	2.15	-3.26	1.11E-03	2.59E-02	110.99	-0.11	0.36	-0.31	0.757	1.000
ENST00000372435	PRPS1	90.86	-1.26	0.41	-3.08	2.10E-03	4.34E-02	6101.78	-0.24	0.18	-1.31	0.192	0.887
ENST00000472686	PRPSAP1	5.55	-5.60	1.76	-3.18	1.48E-03	3.26E-02	164.89	0.36	0.27	1.33	0.185	0.879
ENST00000488339	PRPSAP1	12.48	-4.58	1.48	-3.09	1.98E-03	4.14E-02	19.88	1.13	1.05	1.08	0.282	0.962

ENST00000481741	PRR3	37.81	-8.38	1.98	-4.24	2.24E-05	8.69E-04	89.80	0.39	0.33	1.18	0.239	0.934
ENST00000467601	PRRC2C	7.68	-20.99	3.03	-6.93	4.30E-12	2.89E-10	31.14	-0.45	0.53	-0.86	0.388	1.000
ENST00000397519	PSIP1	6.12	-20.68	3.03	-6.82	8.95E-12	5.28E-10	5186.70	-0.20	0.30	-0.65	0.513	1.000
ENST00000445517	PSMA2	34.98	-8.26	1.08	-7.62	2.51E-14	6.45E-12	674.83	-0.11	0.34	-0.33	0.740	1.000
ENST00000559146	PSMA4	15.06	-21.89	3.03	-7.23	4.80E-13	5.77E-11	208.95	0.34	0.39	0.87	0.386	1.000
ENST00000370861	PSMA7	98.06	-9.66	3.03	-3.19	1.41E-03	3.14E-02	1048.06	1.10	0.80	1.37	0.171	0.858
ENST00000555895	PSMB5	13.04	-21.70	3.03	-7.17	7.71E-13	7.99E-11	4.08	5.41	4.80	1.13	0.259	0.950
ENST00000292644	PSMC2	246.04	-11.08	2.99	-3.70	2.12E-04	6.41E-03	2629.43	3.95	1.19	3.31	0.001	0.034
ENST00000544811	PSMC2	33.44	-22.98	3.03	-7.59	3.18E-14	7.75E-12	466.02	-0.45	0.49	-0.92	0.359	0.999
ENST00000524447	PSMC3	14.45	-21.84	3.03	-7.21	5.51E-13	6.41E-11	266.20	-0.05	0.39	-0.13	0.899	1.000
ENST00000409643	PSMD1	27.39	-22.56	3.03	-7.45	9.23E-14	1.64E-11	3796.16	-0.25	0.16	-1.54	0.125	0.783
ENST00000382671	PSMD13	9.20	-21.23	3.03	-7.01	2.41E-12	1.90E-10	20.91	0.32	3.92	0.08	0.935	1.000
ENST00000532097	PSMD13	233.42	-0.97	0.32	-2.98	2.87E-03	5.53E-02	3589.90	0.09	0.16	0.54	0.587	1.000
ENST00000417952	PSMD2	19.54	-7.42	1.03	-7.23	4.74E-13	5.71E-11	17.01	1.13	1.02	1.11	0.267	0.954
ENST00000435761	PSMD2	20.51	-22.31	3.03	-7.37	1.72E-13	2.58E-11	252.59	0.05	0.58	0.09	0.928	1.000
ENST00000445776	PSMD4	13.56	-21.74	3.03	-7.18	6.93E-13	7.38E-11	57.25	-0.18	0.63	-0.28	0.782	1.000
ENST00000476949	PSMD5	3.35	-4.88	1.70	-2.88	4.01E-03	7.16E-02	17.00	1.55	2.12	0.73	0.464	1.000
ENST00000543699	PSMD9	9.09	-21.21	3.03	-7.00	2.51E-12	1.97E-10	97.74	-0.58	0.62	-0.93	0.352	0.994
ENST00000544254	PSMD9	10.39	-6.51	1.16	-5.62	1.90E-08	9.51E-07	204.74	-0.62	0.47	-1.32	0.188	0.881
ENST00000559056	PSME2	20.57	-7.50	2.68	-2.80	5.11E-03	8.59E-02	104.98	0.52	0.41	1.27	0.203	0.898
ENST00000478731	PSME4	9.17	-1.74	0.66	-2.63	8.60E-03	1.24E-01	27.12	0.48	0.64	0.76	0.450	1.000
ENST00000438768	PSMF1	29.99	-3.88	1.49	-2.61	9.15E-03	1.29E-01	342.09	-1.95	0.94	-2.09	0.037	0.449
ENST00000236228	PTBP2	20.21	-1.90	0.51	-3.70	2.13E-04	6.44E-03	494.09	-0.49	0.33	-1.50	0.134	0.801
ENST00000483925	PTCD3	8.11	-20.64	3.03	-6.81	9.64E-12	5.63E-10	22.80	0.41	0.57	0.72	0.471	1.000
ENST00000338961	PTGES2	44.48	-1.04	0.36	-2.85	4.42E-03	7.69E-02	517.00	-0.12	0.17	-0.71	0.477	1.000
ENST00000407693	PTGR1	15.08	-7.05	2.60	-2.71	6.77E-03	1.05E-01	96.55	0.79	2.33	0.34	0.735	1.000
ENST00000393083	PTN	29.77	-8.03	1.59	-5.04	4.54E-07	2.09E-05	4088.75	-0.16	0.22	-0.73	0.463	1.000
ENST00000372413	PTPRF	9.91	-6.44	1.85	-3.47	5.14E-04	1.37E-02	1176.80	1.58	1.74	0.91	0.364	0.999
ENST00000527197	PUF60	30.77	-4.56	1.52	-3.01	2.63E-03	5.19E-02	6396.34	0.07	0.50	0.14	0.885	1.000
ENST00000356362	PUS7	57.45	-1.20	0.41	-2.93	3.34E-03	6.24E-02	2520.70	-0.03	0.14	-0.21	0.834	1.000
ENST00000478208	PUS7	1.89	-4.05	1.27	-3.20	1.38E-03	3.09E-02	29.65	0.29	0.56	0.51	0.609	1.000
ENST00000319792	PVRL3	9.93	-21.33	3.03	-7.04	1.90E-12	1.59E-10	44.20	0.16	0.66	0.25	0.803	1.000
ENST00000541166	PWP1	21.67	-5.46	1.90	-2.87	4.07E-03	7.22E-02	3648.82	0.00	0.13	-0.03	0.977	1.000
ENST00000486126	PWP2	4.19	-5.20	1.66	-3.14	1.69E-03	3.63E-02	19.04	0.09	0.68	0.14	0.891	1.000
ENST00000425171	PXDN	12.55	-6.79	1.05	-6.49	8.70E-11	4.72E-09	322.80	0.60	0.23	2.58	0.010	0.206
ENST00000530336	PYGL	26.57	-4.40	1.29	-3.40	6.69E-04	1.71E-02	1557.79	0.06	0.41	0.14	0.891	1.000
ENST00000531889	PYGL	54.80	-23.65	3.03	-7.81	5.60E-15	2.01E-12	1537.07	0.43	0.26	1.64	0.100	0.722
ENST00000532107	PYGL	20.74	-6.36	2.33	-2.72	6.43E-03	1.01E-01	106.58	0.03	0.43	0.07	0.945	1.000
ENST00000553872	PYGL	10.04	-6.47	1.38	-4.69	2.79E-06	1.21E-04	771.72	0.14	0.24	0.60	0.551	1.000
ENST00000567671	RAB11A	44.07	-7.43	1.75	-4.26	2.08E-05	8.12E-04	37.92	-1.06	0.71	-1.48	0.138	0.806
ENST00000462680	RAB13	26.15	-1.82	0.65	-2.78	5.47E-03	9.00E-02	703.47	0.41	0.42	0.97	0.332	0.987
ENST00000484297	RAB13	18.32	-1.42	0.39	-3.64	2.76E-04	8.08E-03	172.04	-0.04	0.31	-0.13	0.901	1.000
ENST00000261263	RAB21	127.93	-1.37	0.38	-3.62	2.93E-04	8.52E-03	4550.23	-0.07	0.15	-0.45	0.654	1.000
ENST00000396696	RAB2A	3.67	-4.37	1.08	-4.03	5.58E-05	1.97E-03	14.62	-1.17	1.11	-1.05	0.293	0.967
ENST00000415040	RAB34	10.18	-6.48	1.75	-3.71	2.08E-04	6.30E-03	603.08	0.34	0.48	0.69	0.489	1.000
ENST00000366690	RAB4A	10.17	-3.86	1.05	-3.68	2.36E-04	7.04E-03	3590.10	0.00	0.13	-0.02	0.983	1.000
ENST00000481981	RAB4A	3.69	-5.01	1.52	-3.29	1.02E-03	2.41E-02	3.64	-1.42	1.52	-0.93	0.351	NA
ENST00000346213	RAB5C	40.73	-7.31	2.42	-3.02	2.53E-03	5.05E-02	4418.57	0.08	0.34	0.23	0.816	1.000
ENST00000336083	RAB6A	13.36	-2.08	0.76	-2.75	5.91E-03	9.54E-02	5842.89	-0.39	0.44	-0.90	0.367	1.000
ENST00000410061	RAB6C	40.49	-0.93	0.33	-2.83	4.72E-03	8.07E-02	128.01	-0.73	0.29	-2.54	0.011	0.220
ENST00000493186	RAB7A	377.81	-1.82	0.51	-3.60	3.23E-04	9.24E-03	48.23	0.24	0.51	0.46	0.645	1.000
ENST00000559006	RAB8B	5.85	-5.68	1.78	-3.18	1.45E-03	3.21E-02	14.36	-0.23	0.91	-0.25	0.800	1.000
ENST00000485090	RABGAP1	6.67	-4.11	1.03	-3.97	7.10E-05	2.44E-03	2.70	-1.45	1.70	-0.85	0.394	NA
ENST00000465063	RABL2B	6.41	-1.86	0.72	-2.60	9.21E-03	1.30E-01	409.10	-0.01	0.29	-0.03	0.973	1.000
ENST00000495499	RAC1	108.10	-2.28	0.36	-6.33	2.48E-10	1.33E-08	302.40	-0.08	0.20	-0.41	0.682	1.000
ENST00000546786	RACGAP1	4.25	-2.98	0.86	-3.47	5.13E-04	1.37E-02	37.56	-0.60	0.46	-1.29	0.196	0.892
ENST00000550149	RACGAP1	36.00	-3.78	1.08	-3.48	4.92E-04	1.32E-02	29.98	-0.04	0.76	-0.05	0.957	1.000
ENST00000520992	RAD21	44.64	-5.56	1.71	-3.25	1.15E-03	2.65E-02	37.71	0.12	0.52	0.23	0.820	1.000

ENST00000536346	RAD51AP1	13.88	-4.03	1.30	-3.10	1.96E-03	4.09E-02	60.35	-0.09	0.75	-0.12	0.907	1.000
ENST00000487270	RAD51B	1.83	-3.33	1.15	-2.90	3.72E-03	6.77E-02	179.91	0.72	0.71	1.01	0.314	0.980
ENST00000371975	RAD54L	45.07	-0.93	0.33	-2.77	5.68E-03	9.26E-02	3673.52	0.22	0.47	0.47	0.641	1.000
ENST00000005257	RALA	337.23	-1.17	0.41	-2.85	4.35E-03	7.62E-02	5861.06	0.13	0.13	1.05	0.293	0.967
ENST00000392367	RAN	7.52	-20.96	3.03	-6.92	4.60E-12	3.05E-10	260.41	0.24	0.47	0.51	0.610	1.000
ENST00000541630	RAN	137.42	-24.88	3.03	-8.22	2.03E-16	1.64E-13	46.70	-0.19	2.41	-0.08	0.935	1.000
ENST00000331821	RANBP1	91.56	-2.22	0.74	-3.00	2.72E-03	5.32E-02	3667.62	-0.03	0.14	-0.21	0.838	1.000
ENST00000423859	RANBP1	6.16	-20.68	3.03	-6.83	8.79E-12	5.21E-10	60.20	-1.43	1.16	-1.23	0.220	0.916
ENST00000259569	RANBP6	66.79	-0.68	0.21	-3.25	1.14E-03	2.65E-02	5828.08	0.00	0.12	0.03	0.973	1.000
ENST00000413361	RANP4	9.62	-3.35	0.65	-5.12	2.99E-07	1.40E-05	28.64	-1.02	0.77	-1.33	0.183	0.876
ENST00000250559	RAP1B	5.67	-4.41	1.07	-4.14	3.46E-05	1.28E-03	958.16	0.39	0.45	0.87	0.387	1.000
ENST00000544639	RAP1B	10.10	-5.87	1.07	-5.49	3.98E-08	1.97E-06	51.17	-0.24	0.58	-0.41	0.683	1.000
ENST00000380158	RAP1GDS1	8.44	-21.11	3.03	-6.97	3.18E-12	2.32E-10	20.15	-1.02	1.36	-0.75	0.455	1.000
ENST00000505026	RAPGEF2	4.52	-2.97	0.93	-3.20	1.38E-03	3.09E-02	78.12	-0.58	0.36	-1.61	0.107	0.742
ENST00000518757	RARS	13.23	-6.27	1.46	-4.30	1.74E-05	6.82E-04	385.79	0.14	0.30	0.47	0.640	1.000
ENST00000521939	RARS	26.87	-22.65	3.03	-7.48	7.32E-14	1.41E-11	39.90	-0.16	0.56	-0.28	0.780	1.000
ENST00000369536	RARS2	7.54	-20.96	3.03	-6.92	4.56E-12	3.03E-10	4741.31	0.02	0.17	0.09	0.930	1.000
ENST00000334062	RASA3	72.25	-0.96	0.34	-2.84	4.50E-03	7.79E-02	14690.10	0.03	0.15	0.23	0.821	1.000
ENST00000405154	RASSF8	23.81	-6.53	1.95	-3.35	8.00E-04	1.98E-02	2788.14	3.71	1.63	2.28	0.023	0.343
ENST00000267163	RB1	254.22	-0.81	0.22	-3.68	2.33E-04	6.96E-03	9444.87	0.02	0.17	0.10	0.920	1.000
ENST00000458695	RBBP4	6.77	-20.78	3.03	-6.86	6.93E-12	4.26E-10	10654.68	-0.08	0.14	-0.54	0.592	1.000
ENST00000367164	RBBP5	17.70	-7.28	2.66	-2.73	6.26E-03	9.96E-02	2273.36	-0.07	0.27	-0.27	0.787	1.000
ENST00000373664	RBL1	118.28	-0.72	0.17	-4.19	2.76E-05	1.05E-03	4020.04	-0.29	0.23	-1.28	0.201	0.896
ENST00000496012	RBM10	6.39	-20.73	3.03	-6.84	7.83E-12	4.75E-10	38.09	0.15	0.56	0.27	0.791	1.000
ENST00000514361	RBM14-RBM4	17.80	-7.29	1.91	-3.82	1.33E-04	4.25E-03	9.95	-0.18	0.88	-0.21	0.838	1.000
ENST00000554256	RBM23	7.49	-20.95	3.03	-6.92	4.67E-12	3.08E-10	5.43	0.05	1.63	0.03	0.975	1.000
ENST00000449987	RBM26	20.72	-2.56	0.85	-3.00	2.69E-03	5.26E-02	4082.23	0.05	0.15	0.31	0.756	1.000
ENST00000403542	RBM39	64.22	-3.48	0.89	-3.89	1.01E-04	3.33E-03	767.84	-0.51	0.58	-0.87	0.383	1.000
ENST00000310092	RBM4	20.66	-4.35	1.63	-2.67	7.66E-03	1.15E-01	326.56	0.28	0.21	1.33	0.183	0.877
ENST00000408993	RBM4	22.73	-7.64	1.88	-4.06	4.84E-05	1.73E-03	966.81	0.31	0.22	1.39	0.164	0.847
ENST00000360475	RBM42	12.32	-21.62	3.03	-7.14	9.31E-13	9.16E-11	1189.95	-0.42	0.21	-1.97	0.049	0.520
ENST00000412742	RCAN3	15.96	-7.13	2.61	-2.73	6.31E-03	1.00E-01	9.00	2.46	2.01	1.22	0.221	0.917
ENST00000533898	RCN1	40.52	-8.47	2.12	-3.99	6.48E-05	2.25E-03	2844.14	0.10	0.26	0.39	0.696	1.000
ENST00000529774	RDX	16.55	-7.18	1.12	-6.39	1.71E-10	9.19E-09	13.18	0.27	1.05	0.26	0.795	1.000
ENST00000444129	RECQL	251.35	-0.78	0.20	-3.89	1.02E-04	3.34E-03	5903.88	0.04	0.12	0.35	0.724	1.000
ENST00000513339	REEP5	8.26	-21.07	3.03	-6.96	3.49E-12	2.50E-10	64.70	0.88	0.60	1.48	0.138	0.807
ENST00000201979	REM1	13.39	-1.24	0.47	-2.65	8.05E-03	1.19E-01	6171.35	-0.21	0.18	-1.17	0.242	0.936
ENST00000422377	REV3L	26.61	-1.13	0.35	-3.28	1.05E-03	2.47E-02	139.36	-3.13	1.32	-2.37	0.018	0.296
ENST00000434425	RFC3	15.67	-7.10	2.69	-2.64	8.38E-03	1.22E-01	390.95	0.79	0.56	1.40	0.160	0.843
ENST00000454402	RFC5	50.43	-8.79	2.09	-4.21	2.51E-05	9.65E-04	1884.26	0.13	0.22	0.59	0.558	1.000
ENST00000464302	RGS2	12.43	-6.77	1.82	-3.72	2.01E-04	6.11E-03	57.58	0.25	0.87	0.29	0.771	1.000
ENST00000367906	RGS4	18.26	-5.75	2.09	-2.75	6.01E-03	9.67E-02	254.24	-0.20	0.31	-0.63	0.526	1.000
ENST00000527809	RGS4	14.41	-21.82	3.03	-7.21	5.73E-13	6.60E-11	16.55	-7.57	4.79	-1.58	0.114	0.760
ENST00000392837	RIC8B	28.48	-1.53	0.54	-2.81	4.91E-03	8.32E-02	1306.94	-0.49	0.39	-1.27	0.204	0.899
ENST00000535839	RLF	31.74	-8.12	2.70	-3.01	2.64E-03	5.20E-02	830.66	-0.58	0.83	-0.70	0.484	1.000
ENST00000283632	RMND5A	35.32	-1.23	0.44	-2.79	5.26E-03	8.78E-02	4292.24	1.00	0.67	1.50	0.133	0.799
ENST00000336617	RNASEH2B	24.82	-2.11	0.72	-2.95	3.18E-03	6.00E-02	356.22	0.15	0.21	0.71	0.476	1.000
ENST00000528220	RNASEH2C	76.05	-4.09	1.50	-2.72	6.54E-03	1.02E-01	606.42	0.00	0.34	0.01	0.993	1.000
ENST00000263895	RND3	10.62	-21.42	3.03	-7.07	1.53E-12	1.35E-10	6067.44	-0.09	0.24	-0.39	0.694	1.000
ENST00000526549	RNF121	6.47	-20.75	3.03	-6.85	7.54E-12	4.60E-10	93.85	0.08	0.33	0.25	0.803	1.000
ENST00000493238	RNF13	13.95	-6.94	1.84	-3.77	1.61E-04	5.04E-03	47.49	0.31	0.48	0.66	0.510	1.000
ENST00000261593	RNF138	29.68	-1.17	0.31	-3.71	2.04E-04	6.19E-03	4381.29	0.00	0.18	0.02	0.983	1.000
ENST00000367510	RNF2	90.54	-1.32	0.48	-2.74	6.16E-03	9.83E-02	3412.81	-0.03	0.15	-0.21	0.834	1.000
ENST00000389120	RNF20	331.62	-0.55	0.21	-2.64	8.41E-03	1.22E-01	9657.51	0.14	0.12	1.13	0.260	0.950
ENST00000282003	RNF219	70.52	-1.53	0.32	-4.78	1.76E-06	7.73E-05	2865.63	0.02	0.13	0.19	0.853	1.000
ENST00000563909	RNF40	8.92	-21.19	3.03	-6.99	2.65E-12	2.05E-10	67.84	0.09	0.43	0.22	0.824	1.000
ENST00000492849	RNPEP	7.40	-20.94	3.03	-6.91	4.82E-12	3.15E-10	44.08	0.05	0.58	0.08	0.936	1.000
ENST00000570051	RNPS1	18.71	-7.36	2.80	-2.63	8.49E-03	1.23E-01	174.36	-0.32	0.26	-1.27	0.205	0.900

ENST00000362512	RNU12	65.29	-1.49	0.30	-4.97	6.83E-07	3.10E-05	5.05	-0.32	1.41	-0.23	0.819	1.000
ENST00000399089	ROCK2	28.70	-21.46	3.03	-7.09	1.35E-12	1.23E-10	177.02	0.58	0.66	0.87	0.385	1.000
ENST00000567078	RP11-1035H13.3.1	28.82	-22.78	3.03	-7.52	5.32E-14	1.13E-11	312.22	-0.17	0.54	-0.31	0.759	1.000
ENST00000436697	RP11-139H15.1.1	5.98	-5.10	1.24	-4.12	3.75E-05	1.38E-03	576.86	0.22	0.21	1.09	0.278	0.961
ENST00000413059	RP11-1A15.2.1	91.18	-0.77	0.27	-2.89	3.80E-03	6.88E-02	10.09	-1.00	0.89	-1.12	0.262	0.952
ENST00000552100	RP11-202G11.1.1	63.81	-0.89	0.31	-2.83	4.60E-03	7.91E-02	1.39	0.40	4.48	0.09	0.929	NA
ENST00000569473	RP11-217B1.2.1	47.10	-2.69	0.91	-2.97	2.95E-03	5.67E-02	32.41	0.79	0.69	1.14	0.255	0.947
ENST00000496294	RP11-234A1.1.1	977.69	-1.09	0.30	-3.63	2.84E-04	8.27E-03	562.84	-0.01	0.19	-0.06	0.950	1.000
ENST00000558778	RP11-323I15.2.1	84.76	-1.33	0.30	-4.49	7.12E-06	2.94E-04	12.95	-0.43	0.93	-0.46	0.645	1.000
ENST00000510543	RP11-323K3.1.1	11.90	-1.29	0.47	-2.75	6.05E-03	9.70E-02	3.14	1.36	2.42	0.56	0.573	NA
ENST00000445455	RP11-325E14.2.1	11.04	-1.26	0.42	-2.97	2.97E-03	5.69E-02	2.44	-0.85	2.33	-0.36	0.716	NA
ENST00000438469	RP11-325P15.2.1	32.82	-0.98	0.29	-3.38	7.13E-04	1.80E-02	43.29	0.22	0.43	0.51	0.610	1.000
ENST00000562949	RP11-343C2.8.1	7.78	-3.24	1.18	-2.74	6.20E-03	9.88E-02	36.49	-0.13	0.83	-0.15	0.878	1.000
ENST00000521717	RP11-359B20.1.1	3.99	-2.54	0.93	-2.74	6.14E-03	9.82E-02	8.41	-1.61	1.17	-1.38	0.169	0.854
ENST00000309865	RP11-368J21.1.1	110.61	-0.82	0.20	-4.05	5.15E-05	1.83E-03	792.30	-0.34	0.16	-2.20	0.028	0.388
ENST00000441206	RP11-36D19.5.1	85.57	-1.26	0.24	-5.27	1.37E-07	6.62E-06	17.86	0.80	0.81	0.98	0.325	0.985
ENST00000446071	RP11-373A9.1.1	60.14	-1.33	0.33	-4.10	4.22E-05	1.53E-03	21.10	0.10	0.61	0.16	0.875	1.000
ENST00000299633	RP11-382A20.3.1	14.09	-2.12	0.73	-2.91	3.67E-03	6.71E-02	7680.38	-0.02	0.12	-0.21	0.831	1.000
ENST00000487533	RP11-385J23.1.1	12.81	-1.37	0.42	-3.28	1.04E-03	2.46E-02	11.91	-0.13	0.98	-0.13	0.895	1.000
ENST00000452964	RP11-3J10.7.1	21.96	-1.14	0.35	-3.23	1.25E-03	2.86E-02	41.75	0.67	0.46	1.45	0.148	0.823
ENST00000567372	RP11-421N8.1.1	78.22	-0.97	0.33	-2.94	3.31E-03	6.21E-02	43.11	0.26	0.48	0.53	0.598	1.000
ENST00000486001	RP11-4K3__A.5.1	72.30	-1.97	0.50	-3.92	9.00E-05	3.03E-03	182.39	-0.12	0.26	-0.44	0.657	1.000
ENST00000489251	RP11-507E23.1.1	105.74	-1.32	0.50	-2.61	8.96E-03	1.28E-01	8.46	-0.06	1.55	-0.04	0.968	1.000
ENST00000466718	RP11-510I6.2.1	148.71	-0.65	0.24	-2.66	7.79E-03	1.16E-01	7.49	-1.88	1.51	-1.24	0.214	0.910
ENST00000548656	RP11-536C10.12.1	9.03	-1.27	0.44	-2.86	4.21E-03	7.42E-02	4.90	0.48	1.15	0.42	0.677	1.000
ENST00000546580	RP11-620J15.3.1	3.91	-3.86	1.41	-2.75	6.02E-03	9.67E-02	1270.82	-0.19	0.19	-1.00	0.316	0.980
ENST00000443998	RP11-632C17__A.1.1	61.89	-1.44	0.49	-2.92	3.54E-03	6.53E-02	2880.80	-0.12	0.18	-0.70	0.486	1.000
ENST00000407054	RP11-63K6.4.1	33.43	-0.91	0.31	-2.88	4.01E-03	7.15E-02	0.64	2.76	4.86	0.57	0.571	NA
ENST00000502784	RP11-65F13.1.1	69.53	-1.41	0.27	-5.19	2.08E-07	9.86E-06	79.42	-0.19	0.42	-0.44	0.659	1.000
ENST00000426243	RP11-753C18.7.1	52.20	-2.12	0.55	-3.86	1.13E-04	3.65E-03	8.22	0.11	1.04	0.11	0.916	1.000
ENST00000569028	RP11-80F22.9.1	2.81	-4.63	1.77	-2.61	8.94E-03	1.27E-01	204.20	0.32	1.11	0.29	0.772	1.000
ENST00000405720	RP1-292B18.1.1	86.39	-0.99	0.30	-3.33	8.68E-04	2.11E-02	80.76	-0.68	0.53	-1.27	0.204	0.899
ENST00000396021	RP13-178D16.2.1	1942.31	-0.68	0.16	-4.31	1.61E-05	6.37E-04	49.55	0.26	0.56	0.46	0.643	1.000
ENST00000408001	RP3-375P9.2.1	16.47	-1.51	0.58	-2.63	8.61E-03	1.24E-01	15.80	0.03	0.68	0.05	0.961	1.000
ENST00000400299	RP3-412A9.11.1	13.76	-6.92	2.61	-2.65	7.98E-03	1.18E-01	1387.72	0.11	0.39	0.28	0.780	1.000
ENST00000546846	RP3-432I18.1.1	7.80	-2.39	0.75	-3.18	1.47E-03	3.25E-02	1.53	1.23	4.27	0.29	0.774	NA
ENST00000565336	RP4-561L24.3.1	291.23	-2.62	0.97	-2.72	6.57E-03	1.03E-01	81.29	-0.13	0.60	-0.21	0.831	1.000
ENST00000469566	RP4-604K5.1.1	20.75	-22.33	3.03	-7.38	1.63E-13	2.48E-11	60.69	-0.53	0.79	-0.67	0.503	1.000
ENST00000434910	RP4-775C13.1.1	126.19	-1.11	0.18	-6.15	7.58E-10	3.99E-08	87.23	-0.32	0.40	-0.80	0.424	1.000
ENST00000414855	RP5-1166A24.1.1	12.87	-1.12	0.43	-2.64	8.29E-03	1.21E-01	7.58	-0.61	1.02	-0.60	0.546	1.000
ENST00000422958	RP5-827C21.1.1	19.66	-2.01	0.57	-3.54	4.07E-04	1.12E-02	9.43	-0.80	1.00	-0.80	0.422	1.000
ENST00000396682	RPA3	19.63	-22.25	3.03	-7.35	1.98E-13	2.90E-11	13.39	7.12	4.79	1.49	0.137	0.806
ENST00000406109	RPA3	33.35	-22.97	3.03	-7.59	3.25E-14	7.79E-12	19.24	-0.65	0.64	-1.02	0.308	0.979
ENST00000547706	RPAP3	6.78	-2.86	0.73	-3.90	9.70E-05	3.22E-03	159.05	-0.48	0.46	-1.05	0.296	0.970
ENST00000490335	RPL10A	6.01	-20.65	3.03	-6.82	9.42E-12	5.52E-10	601.00	-0.16	0.24	-0.68	0.498	1.000
ENST00000472730	RPL10AP6	44.09	-0.79	0.30	-2.64	8.32E-03	1.21E-01	101.37	-0.05	0.30	-0.18	0.859	1.000
ENST00000391857	RPL13A	18.41	-20.52	3.03	-6.78	1.22E-11	6.88E-10	106268.30	-0.28	0.26	-1.07	0.283	0.962
ENST00000467825	RPL13A	14.74	-7.02	1.78	-3.94	8.22E-05	2.79E-03	13071.77	0.11	0.35	0.30	0.761	1.000
ENST00000461368	RPL14	131.68	-1.09	0.42	-2.58	9.89E-03	1.36E-01	99.55	0.07	0.32	0.21	0.833	1.000
ENST00000418495	RPL17	81.19	-0.71	0.27	-2.62	8.75E-03	1.25E-01	35889.99	-0.19	0.13	-1.42	0.157	0.839
ENST00000546623	RPL18	5.61	-20.56	3.03	-6.78	1.18E-11	6.66E-10	149.85	0.35	0.58	0.59	0.552	1.000
ENST00000295830	RPL22L1	68.14	-4.46	1.48	-3.02	2.53E-03	5.04E-02	3355.44	-0.75	0.16	-4.74	0.000	0.000
ENST00000463836	RPL22L1	40.84	-23.24	3.03	-7.68	1.61E-14	4.73E-12	2348.92	-0.61	0.16	-3.76	0.000	0.008
ENST00000245857	RPL23	442.06	-11.35	1.67	-6.77	1.25E-11	7.00E-10	898.83	0.28	3.89	0.07	0.942	1.000
ENST00000419125	RPL26P30	10.87	-3.60	1.31	-2.75	5.89E-03	9.54E-02	80.10	-0.66	1.40	-0.47	0.638	1.000
ENST00000216146	RPL3	104.43	-5.36	1.00	-5.36	8.27E-08	4.04E-06	123634.89	-0.26	0.33	-0.80	0.426	1.000
ENST00000517489	RPL30	7.15	-20.87	3.03	-6.89	5.59E-12	3.55E-10	273.48	0.15	0.28	0.52	0.604	1.000
ENST00000518850	RPL30	8.02	-21.04	3.03	-6.95	3.74E-12	2.61E-10	2923.33	1.83	0.77	2.38	0.017	0.289

ENST00000441435	RPL31	12.69	-21.54	3.03	-7.11	1.15E-12	1.08E-10	394.73	0.23	0.34	0.67	0.506	1.000
ENST00000434963	RPL32	6.56	-20.74	3.03	-6.84	7.66E-12	4.67E-10	57.63	-0.06	0.58	-0.11	0.915	1.000
ENST00000394667	RPL34	65.40	-9.17	2.80	-3.28	1.05E-03	2.47E-02	368.63	0.41	0.41	1.00	0.318	0.982
ENST00000429437	RPL35A	10.10	-21.35	3.03	-7.05	1.79E-12	1.52E-10	7549.43	-0.12	0.30	-0.41	0.682	1.000
ENST00000439255	RPL35A	4.07	-5.17	1.78	-2.91	3.64E-03	6.68E-02	3034.01	0.00	0.14	-0.01	0.994	1.000
ENST00000420712	RPL37A	22.78	-4.70	1.67	-2.82	4.82E-03	8.19E-02	108.30	0.26	0.34	0.77	0.440	1.000
ENST00000448289	RPL39P6	17.01	-2.22	0.62	-3.55	3.86E-04	1.07E-02	1.36	0.95	2.60	0.37	0.715	NA
ENST00000315741	RPL5	62.22	-23.82	3.03	-7.87	3.54E-15	1.53E-12	811.92	-0.80	0.27	-2.93	0.003	0.096
ENST00000546368	RPL6	15.71	-1.87	0.60	-3.14	1.67E-03	3.60E-02	2736.09	-0.19	0.22	-0.87	0.385	1.000
ENST00000550238	RPL6	14.40	-1.47	0.48	-3.09	2.02E-03	4.20E-02	522.47	0.13	0.40	0.33	0.738	1.000
ENST00000315731	RPL7A	290.96	-4.38	1.46	-3.00	2.72E-03	5.31E-02	979.92	-0.39	0.22	-1.76	0.078	0.650
ENST00000462348	RPL7L1	17.66	-4.12	1.05	-3.92	9.01E-05	3.03E-03	174.92	0.50	0.34	1.48	0.140	0.810
ENST00000527914	RPL8	8.82	-19.13	3.03	-6.32	2.69E-10	1.44E-08	1617.41	-0.42	0.19	-2.28	0.023	0.344
ENST00000528957	RPL8	570.93	-2.57	0.96	-2.67	7.56E-03	1.14E-01	1844.38	-0.81	0.73	-1.11	0.269	0.955
ENST00000529920	RPL8	7.85	-21.02	3.03	-6.94	4.00E-12	2.74E-10	1831.12	-0.02	0.22	-0.11	0.916	1.000
ENST00000550296	RPLP0	26.32	-22.07	3.03	-7.29	3.12E-13	4.15E-11	29.15	0.46	0.61	0.75	0.451	1.000
ENST00000373632	RPN2	35.72	-3.00	0.99	-3.02	2.49E-03	4.98E-02	681.29	0.82	0.91	0.90	0.369	1.000
ENST00000445193	RPP14	8.81	-2.60	0.72	-3.62	2.98E-04	8.62E-03	175.63	-1.02	0.25	-4.15	0.000	0.002
ENST00000474973	RPS18	8.29	-21.09	3.03	-6.96	3.38E-12	2.43E-10	1066.15	-0.08	0.21	-0.40	0.691	1.000
ENST00000479802	RPS18	539.18	-1.52	0.40	-3.81	1.36E-04	4.33E-03	794.07	-0.38	0.32	-1.19	0.235	0.929
ENST00000526586	RPS2	7.48	-6.04	1.39	-4.35	1.34E-05	5.30E-04	2387.01	-0.29	0.17	-1.70	0.089	0.691
ENST00000504293	RPS23	249.10	-2.37	0.61	-3.89	9.87E-05	3.27E-03	6733.98	-0.22	0.86	-0.25	0.803	1.000
ENST00000440692	RPS24	48.44	-8.73	1.49	-5.88	4.21E-09	2.18E-07	564.10	-0.88	2.54	-0.34	0.730	1.000
ENST00000532567	RPS25	122.03	-2.31	0.78	-2.97	3.00E-03	5.73E-02	2993.69	-0.50	0.32	-1.57	0.118	0.767
ENST00000477151	RPS27	1.70	-3.89	1.38	-2.83	4.71E-03	8.06E-02	432.98	-0.30	0.22	-1.32	0.187	0.880
ENST00000557367	RPS29	29.22	-1.50	0.57	-2.64	8.19E-03	1.20E-01	298.71	-0.48	0.31	-1.57	0.117	0.766
ENST00000528439	RPS3	224.49	-1.53	0.55	-2.79	5.27E-03	8.79E-02	985.32	-0.53	0.32	-1.69	0.092	0.700
ENST00000534440	RPS3	39.79	-8.45	1.88	-4.50	6.95E-06	2.87E-04	15674.40	0.36	0.29	1.23	0.217	0.913
ENST00000398541	RPS4L.1	71.27	-2.24	0.55	-4.08	4.50E-05	1.62E-03	22.92	-0.45	0.57	-0.79	0.427	1.000
ENST00000315377	RPS6	121.64	-24.73	3.03	-8.17	3.04E-16	2.32E-13	68154.04	-0.46	0.26	-1.77	0.076	0.643
ENST00000379565	RPS6KA3	79.22	-1.25	0.40	-3.11	1.86E-03	3.92E-02	11260.09	-0.14	0.20	-0.72	0.472	1.000
ENST00000304921	RPS7	16.66	-22.02	3.03	-7.27	3.49E-13	4.52E-11	71895.56	-0.20	0.15	-1.32	0.186	0.880
ENST00000396651	RPS8	38.88	-8.42	2.27	-3.70	2.15E-04	6.49E-03	77806.61	-0.20	0.20	-1.01	0.313	0.980
ENST00000474582	RPS8	29.94	-1.72	0.47	-3.69	2.27E-04	6.80E-03	1965.07	-1.09	0.54	-2.01	0.045	0.496
ENST00000391752	RPS9	69.10	-23.67	3.03	-7.82	5.20E-15	1.97E-12	466.02	-0.29	0.19	-1.51	0.131	0.795
ENST00000246043	RRBP1	84.22	-24.23	3.03	-8.01	1.19E-15	7.03E-13	38852.53	-0.01	0.20	-0.07	0.944	1.000
ENST00000300738	RRM1	85.57	-8.97	2.46	-3.65	2.58E-04	7.60E-03	7225.34	-0.19	0.37	-0.51	0.612	1.000
ENST00000485717	RRM2	41.19	-4.26	1.28	-3.34	8.52E-04	2.09E-02	22.23	-0.76	0.62	-1.21	0.225	0.920
ENST00000526324	RSF1	5.67	-20.57	3.03	-6.79	1.14E-11	6.50E-10	229.14	0.64	0.46	1.38	0.168	0.853
ENST00000394420	RSPRY1	15.98	-7.13	2.69	-2.65	8.06E-03	1.19E-01	460.57	-0.66	0.46	-1.44	0.151	0.827
ENST00000370128	RTCD1	62.83	-0.76	0.29	-2.60	9.38E-03	1.31E-01	2909.48	-0.27	0.24	-1.09	0.274	0.959
ENST00000255674	RTTN	22.85	-1.30	0.45	-2.87	4.06E-03	7.21E-02	6980.19	-0.26	0.12	-2.26	0.024	0.353
ENST00000464873	RUVBL1	45.55	-6.92	1.20	-5.76	8.34E-09	4.29E-07	559.13	0.16	0.19	0.85	0.397	1.000
ENST00000221413	RUVBL2	110.52	-9.92	2.87	-3.45	5.56E-04	1.47E-02	4601.23	-0.07	0.15	-0.47	0.639	1.000
ENST00000368809	S100A10	156.02	-9.45	2.25	-4.19	2.74E-05	1.05E-03	964.16	4.27	1.73	2.47	0.014	0.248
ENST00000368704	S100A16	8.84	-5.67	1.98	-2.87	4.16E-03	7.35E-02	141.48	-0.73	0.39	-1.88	0.060	0.572
ENST00000354332	S100A4	15.19	-2.82	0.93	-3.05	2.28E-03	4.63E-02	20.22	-0.95	0.63	-1.52	0.129	0.790
ENST00000382298	SACS	181.30	-0.66	0.23	-2.92	3.50E-03	6.48E-02	1251.09	-0.13	0.75	-0.17	0.865	1.000
ENST00000382533	SAP18	158.21	-0.95	0.36	-2.64	8.41E-03	1.22E-01	5141.93	0.02	0.13	0.16	0.872	1.000
ENST00000450573	SAP18	20.10	-1.86	0.47	-3.98	7.01E-05	2.42E-03	13.90	-0.28	0.97	-0.29	0.771	1.000
ENST00000467636	SAP18	10.96	-6.59	1.02	-6.44	1.18E-10	6.37E-09	5.33	-0.90	1.20	-0.75	0.452	1.000
ENST00000471009	SAP18	16.94	-2.57	0.50	-5.13	2.83E-07	1.33E-05	253.64	-0.20	0.31	-0.64	0.525	1.000
ENST00000296504	SAP30	31.03	-2.06	0.76	-2.71	6.77E-03	1.05E-01	1869.23	0.27	0.26	1.04	0.299	0.972
ENST00000373241	SAR1A	50.38	-8.79	2.76	-3.18	1.47E-03	3.25E-02	546.40	0.31	0.45	0.69	0.493	1.000
ENST00000402673	SAR1B	87.12	-1.85	0.69	-2.69	7.11E-03	1.09E-01	4650.77	0.05	0.13	0.36	0.717	1.000
ENST00000369923	SARS	8.36	-6.20	1.51	-4.12	3.85E-05	1.41E-03	440.05	-0.04	0.51	-0.09	0.930	1.000
ENST00000547397	SART3	27.84	-7.93	1.95	-4.06	4.91E-05	1.75E-03	337.45	0.34	0.28	1.22	0.223	0.917
ENST00000462159	SASS6	31.30	-2.06	0.76	-2.71	6.70E-03	1.04E-01	1320.27	0.02	0.29	0.07	0.948	1.000

ENST00000269298	SAT2	4.86	-3.24	0.96	-3.36	7.78E-04	1.93E-02	1892.48	0.64	0.38	1.71	0.088	0.686
ENST00000246868	SBDS	324.00	-0.95	0.20	-4.69	2.71E-06	1.17E-04	3750.38	0.10	0.13	0.81	0.416	1.000
ENST00000490953	SBDS	2.50	-4.46	1.69	-2.64	8.19E-03	1.20E-01	28.92	0.53	0.93	0.57	0.566	1.000
ENST00000423610	SC22CB-1E7.1.1	63.69	-1.55	0.53	-2.90	3.72E-03	6.77E-02	701.76	-0.22	0.19	-1.20	0.231	0.926
ENST00000538629	SCAMP1	66.10	-1.02	0.27	-3.76	1.70E-04	5.28E-03	1087.45	0.32	0.18	1.82	0.069	0.614
ENST00000261693	SCARB1	58.74	-4.98	1.56	-3.19	1.42E-03	3.16E-02	770.79	-0.20	0.41	-0.48	0.632	1.000
ENST00000546215	SCARB1	15.37	-21.77	3.03	-7.19	6.56E-13	7.15E-11	1399.67	0.07	0.16	0.42	0.675	1.000
ENST00000370355	SCD	445.07	-2.03	0.77	-2.64	8.24E-03	1.20E-01	60909.09	-0.05	0.12	-0.44	0.660	1.000
ENST00000487197	SCLY	2.20	-3.59	1.15	-3.12	1.81E-03	3.83E-02	39.11	-1.09	0.47	-2.33	0.020	0.318
ENST00000402904	SCMH1	19.58	-2.12	0.41	-5.13	2.90E-07	1.36E-05	485.01	-0.71	0.50	-1.43	0.152	0.830
ENST00000255390	SCO1	63.46	-0.87	0.28	-3.14	1.70E-03	3.65E-02	3999.24	0.18	0.21	0.83	0.408	1.000
ENST00000502535	SCOC	8.20	-21.06	3.03	-6.95	3.62E-12	2.56E-10	148.40	1.54	0.89	1.73	0.083	0.669
ENST00000409497	SCRN1	58.08	-8.99	2.37	-3.79	1.51E-04	4.74E-03	29.06	0.09	0.80	0.11	0.912	1.000
ENST00000416113	SCRN1	29.24	-22.78	3.03	-7.52	5.32E-14	1.13E-11	67.10	0.44	0.77	0.57	0.566	1.000
ENST00000395710	SDAD1	107.05	-0.85	0.29	-2.93	3.41E-03	6.35E-02	141.84	-0.16	0.46	-0.35	0.725	1.000
ENST00000504824	SDHA	6.69	-20.76	3.03	-6.85	7.41E-12	4.52E-10	309.17	-0.64	0.44	-1.46	0.145	0.819
ENST00000558217	SEC11A	83.14	-9.51	2.82	-3.37	7.44E-04	1.86E-02	477.75	-0.16	0.33	-0.47	0.636	1.000
ENST00000350697	SEC13	73.86	-9.34	1.97	-4.73	2.23E-06	9.69E-05	1884.73	-0.35	0.40	-0.89	0.373	1.000
ENST00000397101	SEC13	14.76	-7.02	2.20	-3.19	1.42E-03	3.15E-02	243.99	0.24	0.28	0.86	0.389	1.000
ENST00000262544	SEC23B	115.93	-9.99	2.91	-3.43	5.93E-04	1.54E-02	3412.55	0.22	0.17	1.27	0.204	0.899
ENST00000369075	SEC23IP	129.54	-0.64	0.25	-2.58	9.94E-03	1.37E-01	8031.13	0.12	0.13	0.89	0.372	1.000
ENST00000339365	SEC24C	51.32	-4.30	0.72	-5.95	2.73E-09	1.42E-07	1839.05	-0.14	0.18	-0.76	0.447	1.000
ENST00000540668	SEC24C	9.05	-20.22	3.03	-6.68	2.45E-11	1.35E-09	71.61	-0.14	0.34	-0.42	0.672	1.000
ENST00000379735	SEC24D	91.86	-0.66	0.21	-3.19	1.40E-03	3.12E-02	3437.81	-0.22	0.26	-0.82	0.413	1.000
ENST00000503226	SEC31A	11.76	-4.98	1.31	-3.81	1.40E-04	4.43E-03	34.82	0.38	0.56	0.68	0.495	1.000
ENST00000554744	SEL1L	6.44	-5.82	1.72	-3.39	6.87E-04	1.75E-02	23.69	-0.54	0.57	-0.95	0.344	0.991
ENST00000557372	SEL1L	42.90	-1.92	0.51	-3.74	1.86E-04	5.74E-03	26.84	-0.14	0.65	-0.21	0.832	1.000
ENST00000526049	SELS.1	85.44	-9.55	2.87	-3.33	8.62E-04	2.10E-02	1370.20	0.36	0.28	1.29	0.197	0.893
ENST00000448372	SENP1	8.12	-6.16	1.93	-3.20	1.40E-03	3.12E-02	626.56	0.15	0.52	0.28	0.777	1.000
ENST00000478753	SEPHS2	25.60	-1.53	0.57	-2.68	7.47E-03	1.13E-01	1877.19	1.77	2.39	0.74	0.457	1.000
ENST00000502584	SEPT11	67.74	-3.55	1.05	-3.38	7.14E-04	1.81E-02	3790.87	-0.04	0.16	-0.26	0.798	1.000
ENST00000510515	SEPT11	16.24	-21.99	3.03	-7.26	3.78E-13	4.77E-11	53.77	-0.17	0.69	-0.25	0.803	1.000
ENST00000360051	SEPT2	37.89	-23.15	3.03	-7.65	2.06E-14	5.71E-12	535.54	0.06	1.45	0.04	0.965	1.000
ENST00000428282	SEPT2	6.51	-20.72	3.03	-6.84	7.98E-12	4.81E-10	18.82	-0.32	0.69	-0.46	0.648	1.000
ENST00000361871	SEPX1	17.84	-2.51	0.94	-2.67	7.67E-03	1.15E-01	1338.60	-0.09	0.19	-0.46	0.645	1.000
ENST00000367102	SERAC1	1.53	-3.76	1.24	-3.05	2.32E-03	4.70E-02	38.29	3.68	1.85	1.99	0.047	0.507
ENST00000462814	SERBP1	191.42	-0.73	0.26	-2.81	4.99E-03	8.43E-02	247.79	-0.60	0.21	-2.84	0.005	0.118
ENST00000445816	SERF2	7.91	-21.03	3.03	-6.94	3.91E-12	2.68E-10	11976.12	0.22	0.14	1.53	0.126	0.785
ENST00000339697	SERINC1	51.87	-22.55	3.03	-7.45	9.23E-14	1.64E-11	10686.05	0.23	0.13	1.80	0.072	0.625
ENST00000479209	SERP1	30.06	-5.18	1.52	-3.40	6.78E-04	1.73E-02	4155.62	-0.78	0.53	-1.47	0.142	0.815
ENST00000467132	SERPINA3	19.37	-3.17	1.08	-2.93	3.44E-03	6.38E-02	31143.46	-0.05	0.15	-0.34	0.730	1.000
ENST00000422298	SF1	13.66	-21.76	3.03	-7.19	6.64E-13	7.20E-11	55.22	-0.28	0.68	-0.41	0.684	1.000
ENST00000489544	SF1	246.98	-0.75	0.28	-2.68	7.41E-03	1.12E-01	678.29	-0.37	0.23	-1.63	0.102	0.730
ENST00000215793	SF3A1	78.52	-8.85	2.30	-3.85	1.20E-04	3.87E-03	2587.29	-0.02	0.30	-0.07	0.942	1.000
ENST00000462258	SF3A3	21.33	-21.57	3.03	-7.13	1.04E-12	9.89E-11	25.15	-0.22	1.12	-0.20	0.841	1.000
ENST00000414963	SF3B1	8.16	-21.04	3.03	-6.95	3.78E-12	2.63E-10	29.93	-0.16	0.60	-0.27	0.790	1.000
ENST00000568291	SF3B3	10.62	-2.51	0.67	-3.74	1.83E-04	5.66E-03	101.40	-0.75	0.30	-2.52	0.012	0.225
ENST00000321442	SFXN1	80.85	-2.18	0.71	-3.07	2.13E-03	4.39E-02	10801.34	0.02	0.11	0.19	0.852	1.000
ENST00000502865	SFXN1	13.92	-21.79	3.03	-7.20	6.24E-13	6.95E-11	44.40	-0.18	0.65	-0.28	0.781	1.000
ENST00000224807	SFXN3	7.68	-20.99	3.03	-6.93	4.30E-12	2.89E-10	10164.33	0.15	0.19	0.80	0.422	1.000
ENST00000521435	SGK3	6.50	-3.74	1.05	-3.57	3.50E-04	9.86E-03	54.74	-0.45	0.52	-0.87	0.386	1.000
ENST00000557348	SHMT2	38.53	-8.40	2.80	-3.00	2.67E-03	5.24E-02	9083.32	-0.04	0.14	-0.31	0.755	1.000
ENST00000212015	SIRT1	72.30	-0.86	0.23	-3.66	2.55E-04	7.53E-03	920.13	-0.13	0.21	-0.60	0.550	1.000
ENST00000536038	SIRT7	11.64	-2.78	0.87	-3.21	1.32E-03	2.98E-02	223.22	0.85	0.81	1.05	0.292	0.967
ENST00000398452	SKA1	56.25	-1.84	0.68	-2.70	6.84E-03	1.06E-01	281.80	-0.12	0.46	-0.26	0.791	1.000
ENST00000494518	SKA1	35.62	-2.72	0.83	-3.27	1.07E-03	2.52E-02	64.22	0.14	0.52	0.26	0.793	1.000
ENST00000426052	SKIL	10.89	-21.45	3.03	-7.08	1.41E-12	1.26E-10	59.25	-0.15	0.44	-0.34	0.735	1.000
ENST00000504997	SKIV2L2	26.25	-7.85	2.06	-3.81	1.37E-04	4.34E-03	49.20	-1.28	0.82	-1.57	0.116	0.765

ENST00000505565	SKIV2L2	13.12	-21.71	3.03	-7.17	7.58E-13	7.89E-11	28.52	0.29	0.63	0.47	0.640	1.000
ENST00000550061	SLC11A2	5.85	-3.90	0.99	-3.96	7.50E-05	2.57E-03	80.56	0.18	0.74	0.24	0.809	1.000
ENST00000547379	SLC16A7	12.47	-6.78	1.74	-3.89	1.02E-04	3.34E-03	442.98	-0.78	0.53	-1.48	0.138	0.807
ENST00000262352	SLC1A1	41.95	-1.13	0.43	-2.63	8.47E-03	1.22E-01	4338.91	0.09	0.21	0.42	0.678	1.000
ENST00000542575	SLC1A5	40.87	-8.49	2.78	-3.06	2.23E-03	4.54E-02	361.86	-4.23	1.50	-2.81	0.005	0.125
ENST00000521645	SLC25A32	33.86	-7.63	2.18	-3.50	4.72E-04	1.27E-02	1381.36	0.14	0.14	0.98	0.329	0.985
ENST00000341119	SLC25A40	23.49	-4.01	1.34	-2.98	2.86E-03	5.53E-02	4305.15	-0.09	0.12	-0.73	0.467	1.000
ENST00000483810	SLC25A40	2.26	-4.30	1.35	-3.20	1.40E-03	3.12E-02	30.28	-0.66	0.55	-1.19	0.235	0.929
ENST00000371731	SLC29A1	8.65	-21.14	3.03	-6.98	2.95E-12	2.21E-10	1063.89	-0.70	0.37	-1.89	0.059	0.568
ENST00000359271	SLC2A10	6.01	-1.51	0.57	-2.65	7.99E-03	1.18E-01	2101.40	-0.37	0.21	-1.78	0.074	0.636
ENST00000357650	SLC30A7	19.01	-21.78	3.03	-7.20	6.24E-13	6.95E-11	212.90	0.11	0.25	0.44	0.658	1.000
ENST00000374212	SLC31A1	66.99	-0.80	0.26	-3.14	1.69E-03	3.64E-02	5277.09	-0.02	0.12	-0.16	0.874	1.000
ENST00000529113	SLC43A3	8.52	-21.12	3.03	-6.97	3.10E-12	2.28E-10	38.89	-0.28	0.53	-0.53	0.593	1.000
ENST00000419036	SLC4A7	23.21	-7.67	2.68	-2.86	4.27E-03	7.50E-02	14941.55	-0.12	0.59	-0.20	0.838	1.000
ENST00000428179	SLC4A7	18.84	-5.25	1.62	-3.25	1.16E-03	2.68E-02	31.14	-0.57	0.76	-0.74	0.458	1.000
ENST00000238688	SLIRP	15.79	-21.96	3.03	-7.25	4.11E-13	5.13E-11	309.69	0.65	0.47	1.39	0.163	0.846
ENST00000432750	SLTM	13.69	-21.77	3.03	-7.19	6.56E-13	7.15E-11	33.15	0.00	0.72	0.01	0.995	1.000
ENST00000497088	SLTM	5.83	-5.68	1.75	-3.25	1.16E-03	2.68E-02	54.13	0.78	0.59	1.31	0.189	0.883
ENST00000545279	SMAD5	64.43	-0.62	0.23	-2.66	7.77E-03	1.16E-01	1337.57	1.80	0.25	7.32	0.000	0.000
ENST00000371123	SMARCA1	187.17	-10.68	2.94	-3.64	2.73E-04	8.03E-03	4990.29	-0.15	1.13	-0.14	0.892	1.000
ENST00000349721	SMARCA2	13.08	-21.58	3.03	-7.13	1.04E-12	9.89E-11	2359.01	-0.01	0.15	-0.09	0.927	1.000
ENST00000359052	SMARCAD1	101.20	-1.24	0.45	-2.76	5.78E-03	9.40E-02	1739.22	0.55	0.65	0.84	0.398	1.000
ENST00000407422	SMARCB1	29.34	-1.93	0.57	-3.37	7.62E-04	1.90E-02	5552.79	-0.55	0.49	-1.13	0.260	0.950
ENST00000491967	SMARCB1	12.19	-21.61	3.03	-7.14	9.65E-13	9.35E-11	219.90	0.00	0.46	0.00	0.998	1.000
ENST00000483847	SMARCC1	18.20	-7.32	2.68	-2.73	6.37E-03	1.01E-01	41.81	-0.18	0.43	-0.42	0.673	1.000
ENST00000347471	SMARCC2	23.44	-22.48	3.03	-7.42	1.13E-13	1.88E-11	302.12	-1.40	0.56	-2.51	0.012	0.229
ENST00000412207	SMARCE1P5	20.93	-2.00	0.39	-5.07	3.95E-07	1.83E-05	2.90	-1.76	2.37	-0.74	0.457	NA
ENST00000374793	SMC2	59.59	-22.51	3.03	-7.44	1.02E-13	1.74E-11	635.35	-0.04	0.50	-0.08	0.940	1.000
ENST00000465563	SMC4	20.22	-7.47	1.00	-7.46	8.89E-14	1.62E-11	25.20	-0.35	1.01	-0.34	0.731	1.000
ENST00000472282	SMC4	2.67	-4.54	1.74	-2.62	8.85E-03	1.26E-01	56.57	0.00	0.59	0.00	0.998	1.000
ENST00000497203	SMC4	14.06	-2.73	0.56	-4.88	1.05E-06	4.70E-05	107.22	-0.16	0.47	-0.34	0.737	1.000
ENST00000361138	SMC5	53.03	-1.43	0.40	-3.61	3.12E-04	8.97E-03	11901.38	-0.02	0.15	-0.12	0.907	1.000
ENST00000351948	SMC6	6.02	-5.73	1.13	-5.05	4.41E-07	2.04E-05	1132.03	0.18	0.69	0.26	0.794	1.000
ENST00000448223	SMC6	206.41	-1.00	0.38	-2.68	7.40E-03	1.12E-01	2120.00	-0.11	0.35	-0.33	0.745	1.000
ENST00000320876	SMCHD1	114.56	-1.07	0.29	-3.71	2.10E-04	6.35E-03	12297.54	-0.14	0.14	-0.99	0.320	0.983
ENST00000433117	SMCR7L	18.65	-7.35	2.70	-2.73	6.39E-03	1.01E-01	283.96	0.15	0.84	0.17	0.862	1.000
ENST00000345102	SMEK2	17.06	-7.23	2.09	-3.46	5.48E-04	1.45E-02	5383.92	0.05	0.14	0.35	0.729	1.000
ENST00000543872	SMG8	8.41	-21.09	3.03	-6.96	3.38E-12	2.43E-10	5086.57	-0.03	0.12	-0.22	0.823	1.000
ENST00000563830	SNAP23	48.55	-23.48	3.03	-7.76	8.69E-15	2.87E-12	31.25	-0.03	0.53	-0.05	0.957	1.000
ENST00000490458	SNAP29	12.17	-21.17	3.03	-6.99	2.72E-12	2.07E-10	66.53	-1.77	0.93	-1.89	0.059	0.569
ENST00000465900	SND1	28.72	-3.13	1.18	-2.65	7.96E-03	1.18E-01	142.68	0.13	0.78	0.17	0.864	1.000
ENST00000486037	SND1	5.38	-5.56	2.05	-2.72	6.54E-03	1.02E-01	48.20	0.41	0.41	1.01	0.315	0.980
ENST00000492840	SND1	4.41	-3.46	1.24	-2.78	5.42E-03	8.96E-02	15.52	-0.04	0.74	-0.06	0.955	1.000
ENST00000521399	SNHG6	7.99	-1.84	0.70	-2.63	8.42E-03	1.22E-01	1462.76	-0.37	0.23	-1.60	0.111	0.752
ENST00000384550	SNORD20	54.77	-1.66	0.50	-3.35	7.98E-04	1.97E-02	114.59	-0.43	0.38	-1.12	0.261	0.952
ENST00000384048	SNORD37	7.04	-2.25	0.58	-3.86	1.15E-04	3.72E-03	21.60	0.13	0.66	0.20	0.839	1.000
ENST00000390833	SNORD67	21.85	-2.73	0.85	-3.20	1.38E-03	3.09E-02	13.83	-0.94	0.73	-1.30	0.195	0.891
ENST00000386747	SNORD83A	21.36	-1.72	0.56	-3.06	2.19E-03	4.48E-02	270.35	-0.03	0.22	-0.14	0.889	1.000
ENST00000263694	SNRNP40	42.36	-23.30	3.03	-7.70	1.40E-14	4.22E-12	497.56	-0.20	0.27	-0.75	0.455	1.000
ENST00000254193	SNRPA1	24.02	-4.14	1.00	-4.12	3.71E-05	1.36E-03	1268.15	0.52	0.45	1.15	0.250	0.941
ENST00000339610	SNRPB	52.84	-7.70	1.86	-4.13	3.59E-05	1.32E-03	5525.13	-0.45	0.23	-1.95	0.051	0.532
ENST00000391932	SNRPD2	18.71	-4.63	1.06	-4.38	1.18E-05	4.73E-04	331.93	-0.25	0.20	-1.25	0.212	0.908
ENST00000468770	SNRPD3	21.81	-3.41	0.95	-3.60	3.21E-04	9.17E-03	31.39	-0.67	0.73	-0.92	0.359	0.999
ENST00000266735	SNRPF	338.07	-0.84	0.19	-4.38	1.17E-05	4.69E-04	4116.61	-0.19	0.19	-0.99	0.321	0.983
ENST00000456025	SNRPGP9	246.21	-2.43	0.32	-7.63	2.29E-14	6.15E-12	2.36	0.48	1.65	0.29	0.772	NA
ENST00000567437	SNUPN	4.03	-3.38	1.27	-2.66	7.75E-03	1.16E-01	15.41	0.42	0.78	0.54	0.590	1.000
ENST00000554324	SNW1	12.78	-6.81	2.30	-2.97	3.02E-03	5.75E-02	41.09	0.47	0.74	0.64	0.524	1.000
ENST00000554775	SNW1	9.67	-21.29	3.03	-7.03	2.06E-12	1.68E-10	1465.08	-1.00	3.64	-0.28	0.783	1.000

ENST00000559844	SNX1	253.98	-11.12	3.01	-3.70	2.19E-04	6.60E-03	547.56	-0.24	0.27	-0.91	0.365	0.999
ENST00000428135	SNX13	18.89	-4.85	1.38	-3.50	4.63E-04	1.25E-02	2994.64	0.55	0.67	0.81	0.416	1.000
ENST00000314673	SNX14	13.86	-21.77	3.03	-7.19	6.58E-13	7.15E-11	932.33	0.01	0.52	0.01	0.991	1.000
ENST00000511365	SNX2	16.28	-1.50	0.42	-3.55	3.92E-04	1.09E-02	65.53	-0.41	0.36	-1.14	0.255	0.947
ENST00000368838	SNX27	9.26	-21.24	3.03	-7.01	2.37E-12	1.88E-10	52.64	-0.29	0.47	-0.60	0.548	1.000
ENST00000426155	SNX3	12.49	-6.78	2.62	-2.59	9.57E-03	1.33E-01	1096.03	-0.44	0.49	-0.90	0.367	1.000
ENST00000536067	SNX4	24.45	-22.55	3.03	-7.45	9.40E-14	1.64E-11	78.85	-0.36	0.33	-1.10	0.272	0.957
ENST00000377759	SNX5	42.30	-23.29	3.03	-7.69	1.42E-14	4.23E-12	75.98	-0.20	0.66	-0.30	0.761	1.000
ENST00000381692	SON	7.96	-21.04	3.03	-6.94	3.82E-12	2.65E-10	4590.33	0.37	0.17	2.26	0.024	0.354
ENST00000426016	SOS1	45.44	-1.11	0.30	-3.66	2.48E-04	7.35E-03	262.32	5.07	1.88	2.70	0.007	0.159
ENST00000528252	SOX6	15.92	-5.94	1.32	-4.52	6.26E-06	2.60E-04	50.88	0.11	2.30	0.05	0.961	1.000
ENST00000327443	SP1	10.95	-21.46	3.03	-7.09	1.38E-12	1.25E-10	375.60	0.25	0.37	0.67	0.501	1.000
ENST00000415673	SP140L	5.55	-20.54	3.03	-6.78	1.22E-11	6.84E-10	850.89	0.22	0.35	0.63	0.531	1.000
ENST00000506500	SPAG9	6.21	-5.77	1.70	-3.39	7.05E-04	1.79E-02	393.78	0.16	0.19	0.87	0.384	1.000
ENST00000510283	SPAG9	75.44	-7.71	1.57	-4.93	8.43E-07	3.81E-05	4354.25	0.58	0.48	1.21	0.227	0.923
ENST00000358677	SPATS2L	26.14	-22.15	3.03	-7.32	2.53E-13	3.51E-11	262.41	3.24	2.45	1.32	0.186	0.880
ENST00000530257	SPCS2	52.64	-8.85	2.00	-4.42	9.80E-06	3.97E-04	105.46	0.27	0.60	0.45	0.656	1.000
ENST00000437398	SPECC1L	237.24	-0.66	0.25	-2.60	9.30E-03	1.31E-01	384.48	-0.15	0.20	-0.72	0.472	1.000
ENST00000216484	SPTLC2	158.52	-1.94	0.57	-3.40	6.70E-04	1.71E-02	6617.59	0.05	0.12	0.43	0.668	1.000
ENST00000557566	SPTLC2	29.41	-0.99	0.28	-3.59	3.28E-04	9.34E-03	11.79	-0.61	1.13	-0.54	0.587	1.000
ENST00000536336	SPTY2D1	10.42	-21.39	3.03	-7.06	1.63E-12	1.41E-10	114.67	-1.38	0.87	-1.57	0.116	0.764
ENST00000360718	SQSTM1	7.15	-20.88	3.03	-6.89	5.47E-12	3.50E-10	2245.73	-2.39	1.08	-2.20	0.028	0.386
ENST00000445403	SRC	4.25	-4.60	1.41	-3.27	1.09E-03	2.54E-02	9.43	6.62	2.07	3.20	0.001	0.047
ENST00000265729	SRI	22.46	-7.04	1.05	-6.69	2.21E-11	1.22E-09	7532.86	-0.01	0.17	-0.06	0.956	1.000
ENST00000486860	SRI	31.51	-8.11	1.02	-7.96	1.70E-15	9.13E-13	479.28	0.17	0.21	0.79	0.431	1.000
ENST00000542536	SRP68	14.02	-20.67	3.03	-6.83	8.79E-12	5.21E-10	1154.86	-0.01	0.41	-0.01	0.989	1.000
ENST00000342756	SRP72	204.84	-0.79	0.19	-4.11	3.99E-05	1.46E-03	12959.72	0.04	0.12	0.35	0.724	1.000
ENST00000477925	SRPK2	5.39	-4.93	1.25	-3.95	7.69E-05	2.63E-03	414.69	-0.30	0.95	-0.32	0.750	1.000
ENST00000490543	SRRM1	6.23	-20.70	3.03	-6.83	8.46E-12	5.06E-10	191.54	-0.41	0.49	-0.83	0.405	1.000
ENST00000426911	SRRM1P3	15.84	-1.13	0.38	-2.97	2.97E-03	5.69E-02	63.28	-0.17	0.40	-0.43	0.666	1.000
ENST00000405432	SRSF11	15.22	-21.91	3.03	-7.23	4.66E-13	5.63E-11	29.09	-0.13	0.56	-0.23	0.816	1.000
ENST00000339436	SRSF3	70.36	-7.65	2.21	-3.47	5.29E-04	1.41E-02	2536.63	-0.16	0.18	-0.91	0.365	0.999
ENST00000546138	SRSF4	9.13	-21.21	3.03	-7.00	2.51E-12	1.97E-10	197.54	0.31	0.73	0.42	0.675	1.000
ENST00000244020	SRSF6	135.82	-10.22	2.91	-3.51	4.54E-04	1.23E-02	39288.02	-0.34	0.13	-2.69	0.007	0.164
ENST00000409276	SRSF7	41.53	-8.51	1.01	-8.45	2.83E-17	2.90E-14	184.36	0.36	0.35	1.01	0.310	0.979
ENST00000446327	SRSF7	44.97	-22.78	3.03	-7.53	5.17E-14	1.11E-11	1103.29	-0.02	0.20	-0.11	0.909	1.000
ENST00000548792	SRSF9	25.82	-1.59	0.46	-3.43	5.99E-04	1.56E-02	6628.26	0.16	0.12	1.39	0.166	0.848
ENST00000260956	SSB	110.26	-24.60	3.03	-8.13	4.38E-16	3.17E-13	4259.88	-0.47	0.40	-1.16	0.246	0.938
ENST00000461708	SSB	13.43	-6.88	2.61	-2.63	8.49E-03	1.23E-01	887.63	0.79	0.91	0.87	0.385	1.000
ENST00000473783	SSBP1	21.51	-2.25	0.85	-2.64	8.19E-03	1.20E-01	36.56	0.06	0.85	0.06	0.949	1.000
ENST00000474597	SSR1	68.12	-23.39	3.03	-7.73	1.09E-14	3.48E-12	48.22	-0.01	0.92	-0.01	0.992	1.000
ENST00000480176	SSR2	7.28	-20.89	3.03	-6.89	5.42E-12	3.49E-10	259.52	-0.46	0.23	-2.00	0.046	0.502
ENST00000486204	SSR4	9.14	-21.22	3.03	-7.01	2.47E-12	1.94E-10	131.76	-0.27	0.34	-0.78	0.436	1.000
ENST00000522430	ST13P6	29.43	-0.96	0.31	-3.08	2.04E-03	4.24E-02	12.02	0.55	1.05	0.52	0.600	1.000
ENST00000470519	ST7L	6.85	-5.31	1.92	-2.77	5.59E-03	9.15E-02	21.88	0.66	0.70	0.94	0.346	0.992
ENST00000236698	STAG1	9.26	-21.24	3.03	-7.01	2.37E-12	1.88E-10	2290.23	0.82	0.45	1.84	0.066	0.600
ENST00000371145	STAG2	33.43	-22.20	3.03	-7.33	2.23E-13	3.15E-11	649.50	0.56	0.74	0.76	0.448	1.000
ENST00000456374	STAG3L1	3.51	-4.95	1.68	-2.95	3.23E-03	6.07E-02	1283.15	-0.12	0.15	-0.81	0.416	1.000
ENST00000463854	STAM2	8.59	-6.23	2.03	-3.08	2.09E-03	4.31E-02	39.94	-0.55	0.47	-1.17	0.243	0.936
ENST00000409465	STAT1	117.65	-9.43	2.41	-3.91	9.22E-05	3.08E-03	809.96	0.22	0.40	0.55	0.585	1.000
ENST00000404369	STEAP1B	1.45	-3.65	1.21	-3.03	2.46E-03	4.94E-02	24.04	-3.04	2.02	-1.51	0.132	0.796
ENST00000360380	STIL	65.46	-1.03	0.34	-3.06	2.21E-03	4.51E-02	377.14	1.27	0.81	1.56	0.119	0.770
ENST00000357865	STMN1	69.85	-23.98	3.03	-7.92	2.33E-15	1.10E-12	11871.48	-0.12	0.13	-0.93	0.352	0.994
ENST00000399728	STMN1	112.45	-8.80	2.54	-3.47	5.28E-04	1.41E-02	5434.38	-0.12	0.14	-0.83	0.409	1.000
ENST00000446334	STMN1	69.81	-8.32	3.03	-2.75	5.99E-03	9.65E-02	231.53	-0.15	0.26	-0.58	0.561	1.000
ENST00000538352	STRAP	17.30	-7.25	2.74	-2.64	8.25E-03	1.20E-01	4.26	2.09	1.54	1.35	0.176	0.866
ENST00000486660	STX4	4.35	-2.22	0.86	-2.58	9.95E-03	1.37E-01	42.63	-0.12	0.52	-0.23	0.818	1.000
ENST00000481942	STXBP1	10.35	-21.39	3.03	-7.06	1.66E-12	1.42E-10	24.05	-1.08	0.59	-1.84	0.066	0.602

ENST00000370008	STXBP3	117.35	-1.14	0.43	-2.66	7.89E-03	1.17E-01	3895.62	0.09	0.14	0.64	0.525	1.000
ENST00000511175	SUB1	104.89	-9.85	1.96	-5.03	5.02E-07	2.30E-05	111.00	-0.81	0.75	-1.08	0.279	0.961
ENST00000393868	SUCLG1	102.58	-0.58	0.23	-2.58	9.87E-03	1.36E-01	6434.29	0.01	0.17	0.04	0.965	1.000
ENST00000371460	SUPT3H	5.08	-4.27	1.09	-3.93	8.60E-05	2.90E-03	46.65	-0.08	0.45	-0.17	0.866	1.000
ENST00000225504	SUPT4H1	202.11	-1.08	0.20	-5.32	1.07E-07	5.19E-06	9494.93	0.04	0.11	0.32	0.753	1.000
ENST00000402194	SUPT5H	13.07	-21.70	3.03	-7.17	7.74E-13	7.99E-11	408.40	1.61	0.29	5.63	0.000	0.000
ENST00000432763	SUPT5H	23.22	-22.48	3.03	-7.43	1.12E-13	1.87E-11	17944.30	0.03	0.19	0.15	0.884	1.000
ENST00000354361	SYF2	6.90	-20.84	3.03	-6.88	6.10E-12	3.82E-10	132.72	0.45	0.39	1.15	0.249	0.939
ENST00000355238	SYNCRIP	192.33	-10.72	1.57	-6.82	8.99E-12	5.29E-10	25391.47	-0.06	0.15	-0.43	0.665	1.000
ENST00000318801	SYNGR1	20.59	-3.03	0.81	-3.73	1.95E-04	5.98E-03	1504.47	-0.27	0.32	-0.85	0.394	1.000
ENST00000464029	SYPL1	18.37	-6.75	2.31	-2.92	3.52E-03	6.50E-02	73.51	-0.26	0.69	-0.38	0.702	1.000
ENST00000484651	TACC3	26.90	-6.10	1.95	-3.13	1.78E-03	3.78E-02	3524.03	-0.08	0.17	-0.46	0.648	1.000
ENST00000467021	TADA1	7.36	-6.02	1.74	-3.46	5.47E-04	1.45E-02	1279.67	0.31	0.25	1.23	0.219	0.914
ENST00000338366	TAF13	95.59	-0.82	0.24	-3.46	5.35E-04	1.42E-02	2312.12	0.29	0.16	1.86	0.063	0.586
ENST00000352967	TAF1A	5.61	-20.56	3.03	-6.78	1.18E-11	6.66E-10	12.01	1.88	0.84	2.23	0.026	0.369
ENST00000369839	TAF5	13.70	-1.38	0.53	-2.62	8.83E-03	1.26E-01	953.88	-0.18	0.15	-1.23	0.220	0.915
ENST00000380818	TAF9	39.91	-8.45	1.23	-6.89	5.42E-12	3.49E-10	776.77	-0.03	0.26	-0.14	0.893	1.000
ENST00000532870	TAGLN	12.90	-6.23	2.08	-3.00	2.68E-03	5.25E-02	305.02	1.94	0.79	2.46	0.014	0.251
ENST00000368096	TAGLN2	228.99	-25.60	3.03	-8.46	2.71E-17	2.84E-14	26974.07	-1.00	1.30	-0.77	0.443	1.000
ENST00000530119	TALDO1	5.82	-5.67	1.88	-3.02	2.52E-03	5.02E-02	713.70	0.09	0.26	0.34	0.731	1.000
ENST00000532685	TALDO1	6.11	-4.50	1.42	-3.17	1.55E-03	3.38E-02	151.57	2.48	1.15	2.15	0.032	0.414
ENST00000273037	TAMM41	10.14	-5.89	1.57	-3.75	1.80E-04	5.56E-03	79.65	0.85	1.37	0.62	0.536	1.000
ENST00000508361	TARS	36.78	-3.81	1.27	-3.00	2.68E-03	5.25E-02	595.07	-0.61	0.41	-1.49	0.137	0.806
ENST00000541634	TARS	46.63	-22.59	3.03	-7.46	8.52E-14	1.59E-11	11.48	0.68	1.41	0.48	0.629	1.000
ENST00000369054	TARS2	15.78	-21.94	3.03	-7.25	4.27E-13	5.27E-11	76.20	-1.24	1.64	-0.75	0.451	1.000
ENST00000225525	TAX1BP3	9.86	-21.32	3.03	-7.04	1.94E-12	1.60E-10	4347.79	0.08	0.14	0.58	0.561	1.000
ENST00000319106	TBC1D15	49.88	-23.52	3.03	-7.77	7.80E-15	2.60E-12	31.47	-0.65	0.50	-1.30	0.194	0.891
ENST00000546450	TBC1D15	2.92	-4.68	1.68	-2.78	5.40E-03	8.93E-02	2282.52	0.06	0.13	0.50	0.616	1.000
ENST00000486274	TBC1D23	18.94	-2.27	0.64	-3.55	3.88E-04	1.08E-02	288.98	2.02	0.92	2.19	0.029	0.390
ENST00000253692	TBC1D5	69.70	-1.31	0.41	-3.16	1.56E-03	3.40E-02	9318.06	0.33	0.25	1.35	0.178	0.868
ENST00000306388	TBCA	31.82	-2.48	0.91	-2.73	6.40E-03	1.01E-01	537.33	0.15	0.19	0.76	0.446	1.000
ENST00000522370	TBCA	57.54	-23.71	3.03	-7.83	4.69E-15	1.84E-12	65.12	0.99	0.66	1.49	0.135	0.803
ENST00000436520	TBCAP1	101.07	-1.27	0.31	-4.11	3.91E-05	1.43E-03	228.49	0.05	0.29	0.16	0.875	1.000
ENST00000331710	TBK1	47.40	-1.49	0.54	-2.73	6.32E-03	1.00E-01	5631.42	0.09	0.12	0.76	0.449	1.000
ENST00000430069	TBL1XR1	167.12	-1.15	0.42	-2.73	6.25E-03	9.95E-02	4186.76	-0.10	0.21	-0.49	0.621	1.000
ENST00000237264	TBPL1	4.88	-3.21	0.92	-3.49	4.81E-04	1.30E-02	1130.88	0.37	0.16	2.36	0.018	0.302
ENST00000521604	TCEA1	31.93	-8.13	1.97	-4.12	3.74E-05	1.37E-03	4482.45	-0.19	0.75	-0.25	0.800	1.000
ENST00000478245	TCTN3	26.16	-7.84	1.57	-4.99	6.01E-07	2.75E-05	35.13	0.09	0.79	0.12	0.906	1.000
ENST00000402696	TF	183.76	-1.61	0.51	-3.16	1.60E-03	3.48E-02	45659.52	-0.10	0.24	-0.44	0.662	1.000
ENST00000467842	TF	30.73	-5.71	1.31	-4.37	1.24E-05	4.95E-04	3227.84	0.29	0.20	1.45	0.147	0.822
ENST00000482271	TF	6.88	-20.79	3.03	-6.86	6.81E-12	4.20E-10	8409.39	-0.14	0.12	-1.12	0.263	0.952
ENST00000373895	TFAM	44.13	-3.68	1.13	-3.27	1.09E-03	2.55E-02	98.29	-0.31	0.37	-0.84	0.400	1.000
ENST00000544618	TFB2M	28.38	-7.96	1.84	-4.33	1.51E-05	5.99E-04	102.30	0.47	0.37	1.29	0.198	0.893
ENST00000495310	TFDP2	3.12	-4.78	1.62	-2.95	3.17E-03	5.98E-02	58.10	-0.36	0.40	-0.90	0.368	1.000
ENST00000481389	TFPI	14.77	-7.02	0.99	-7.07	1.50E-12	1.33E-10	11.71	-3.16	1.02	-3.09	0.002	0.063
ENST00000392396	TFRC	177.00	-8.25	2.01	-4.10	4.07E-05	1.48E-03	14482.73	-0.85	0.66	-1.29	0.198	0.893
ENST00000535031	TFRC	69.76	-23.73	3.03	-7.84	4.44E-15	1.79E-12	48.19	-0.97	2.33	-0.42	0.676	1.000
ENST00000536701	TGM2	21.14	-5.16	1.85	-2.80	5.18E-03	8.69E-02	86.84	-1.28	0.61	-2.11	0.035	0.437
ENST00000398263	TGOLN2	27.73	-22.72	3.03	-7.50	6.16E-14	1.25E-11	14371.29	0.36	1.23	0.29	0.770	1.000
ENST00000245838	THOC2	105.91	-0.72	0.23	-3.12	1.80E-03	3.81E-02	5996.48	-0.08	0.13	-0.64	0.519	1.000
ENST00000469141	THRAP3	37.08	-23.11	3.03	-7.64	2.25E-14	6.08E-12	312.78	0.85	0.66	1.29	0.197	0.893
ENST00000369087	TIAL1	5.36	-5.56	1.68	-3.32	9.05E-04	2.19E-02	1636.45	0.02	0.32	0.08	0.940	1.000
ENST00000472645	TIMM17B	4.58	-4.71	1.77	-2.66	7.88E-03	1.17E-01	61.28	-0.46	1.66	-0.28	0.783	1.000
ENST00000264244	TIMMDC1	50.84	-22.55	3.03	-7.45	9.36E-14	1.64E-11	426.27	-0.60	1.58	-0.38	0.702	1.000
ENST00000456754	TIMP1	14.82	-3.71	1.05	-3.53	4.19E-04	1.15E-02	14977.84	0.11	0.11	0.95	0.341	0.990
ENST00000536189	TIMP2	5.61	-20.56	3.03	-6.78	1.18E-11	6.66E-10	52.72	0.05	0.44	0.11	0.913	1.000
ENST00000356107	TJP1	13.14	-21.71	3.03	-7.17	7.53E-13	7.87E-11	1704.49	0.39	0.37	1.06	0.290	0.966
ENST00000400007	TJP1	29.30	-8.01	2.74	-2.93	3.43E-03	6.38E-02	6961.24	-0.13	0.16	-0.83	0.408	1.000

ENST00000561307	TJP1	12.30	-6.76	2.59	-2.60	9.19E-03	1.30E-01	14.87	0.08	0.70	0.11	0.914	1.000
ENST00000535702	TJP2	13.58	-21.75	3.03	-7.18	6.79E-13	7.32E-11	2720.66	-0.17	0.32	-0.53	0.593	1.000
ENST00000423525	TKT	59.67	-22.89	3.03	-7.56	3.93E-14	9.01E-12	14820.32	-1.50	0.69	-2.19	0.029	0.392
ENST00000450814	TKT	67.76	-9.22	2.87	-3.22	1.30E-03	2.95E-02	2276.18	0.17	1.07	0.16	0.874	1.000
ENST00000376499	TLE1	23.42	-1.12	0.37	-2.98	2.86E-03	5.52E-02	2921.44	-0.04	0.16	-0.23	0.819	1.000
ENST00000443638	TM9SF3	7.96	-21.04	3.03	-6.94	3.82E-12	2.65E-10	8.99	-1.98	1.70	-1.17	0.243	0.936
ENST00000547798	TMBIM6	20.36	-5.47	1.65	-3.31	9.43E-04	2.26E-02	56197.78	0.11	0.13	0.83	0.408	1.000
ENST00000464650	TMCO1	199.81	-10.78	2.09	-5.17	2.36E-07	1.11E-05	2370.69	-3.42	1.46	-2.35	0.019	0.306
ENST00000555036	TMED10	9.03	-21.20	3.03	-7.00	2.56E-12	2.00E-10	267.96	0.13	0.38	0.34	0.736	1.000
ENST00000557670	TMED10	102.06	-24.49	3.03	-8.09	5.89E-16	3.92E-13	91.13	0.34	0.31	1.10	0.270	0.955
ENST00000370280	TMED5	17.63	-1.81	0.65	-2.78	5.46E-03	8.99E-02	106.94	-0.03	0.30	-0.10	0.922	1.000
ENST00000503010	TMED7	26.02	-22.63	3.03	-7.48	7.69E-14	1.46E-11	49.34	-1.14	0.47	-2.42	0.015	0.270
ENST00000282382	TMED7-TICAM2	23.00	-22.42	3.03	-7.41	1.30E-13	2.05E-11	78.18	5.77	2.28	2.53	0.012	0.225
ENST00000333314	TMED7-TICAM2	16.87	-22.04	3.03	-7.28	3.32E-13	4.35E-11	1144.46	-0.24	0.22	-1.06	0.288	0.965
ENST00000216468	TMED8	36.92	-0.69	0.26	-2.60	9.25E-03	1.30E-01	11410.60	-0.07	0.11	-0.58	0.559	1.000
ENST00000396668	TMEM106B	10.40	-21.39	3.03	-7.06	1.63E-12	1.41E-10	375.39	-0.07	0.41	-0.16	0.871	1.000
ENST00000420833	TMEM106B	10.30	-5.31	1.84	-2.88	3.98E-03	7.12E-02	223.08	-0.40	0.87	-0.46	0.648	1.000
ENST00000245046	TMEM111	217.77	-0.63	0.21	-3.04	2.37E-03	4.77E-02	5484.50	0.14	0.12	1.24	0.216	0.912
ENST00000340353	TMEM135	16.46	-3.47	1.20	-2.89	3.79E-03	6.87E-02	2588.66	0.09	0.15	0.58	0.563	1.000
ENST00000381787	TMEM138	8.59	-6.24	1.82	-3.43	5.98E-04	1.56E-02	1123.30	0.17	0.21	0.81	0.415	1.000
ENST00000511118	TMEM167A	7.60	-2.95	0.96	-3.06	2.23E-03	4.54E-02	16.11	-0.03	0.69	-0.05	0.962	1.000
ENST00000338272	TMEM167B	26.13	-1.08	0.35	-3.05	2.29E-03	4.65E-02	5729.26	0.17	0.11	1.46	0.143	0.816
ENST00000306480	TMEM192	24.94	-1.41	0.41	-3.41	6.51E-04	1.67E-02	4379.54	-0.02	0.13	-0.15	0.883	1.000
ENST00000377066	TMEM2	11.30	-3.67	1.09	-3.38	7.33E-04	1.84E-02	3560.40	0.90	0.30	2.96	0.003	0.087
ENST00000533723	TMEM41B	8.25	-6.18	1.11	-5.56	2.71E-08	1.35E-06	24.25	-1.85	1.01	-1.82	0.068	0.609
ENST00000537333	TMEM48	12.50	-20.52	3.03	-6.77	1.25E-11	6.98E-10	402.50	1.90	1.13	1.68	0.093	0.705
ENST00000480937	TMEM50A	14.78	-7.02	1.96	-3.58	3.38E-04	9.57E-03	120.63	0.45	0.31	1.46	0.144	0.817
ENST00000371341	TMEM59	103.01	-2.32	0.66	-3.52	4.37E-04	1.19E-02	1554.46	0.42	0.35	1.21	0.226	0.922
ENST00000257663	TMEM60	24.53	-1.26	0.33	-3.82	1.33E-04	4.25E-03	1421.14	-0.02	0.16	-0.13	0.896	1.000
ENST00000518174	TMEM66	9.05	-20.22	3.03	-6.68	2.45E-11	1.35E-09	1129.51	0.33	0.22	1.52	0.128	0.789
ENST00000523127	TMEM66	10.65	-21.43	3.03	-7.07	1.50E-12	1.33E-10	96.72	-0.68	0.60	-1.13	0.256	0.948
ENST00000545648	TMEM66	21.81	-6.40	2.39	-2.67	7.49E-03	1.13E-01	327.57	-0.12	0.26	-0.45	0.655	1.000
ENST00000452614	TMEM87B	13.27	-6.87	2.59	-2.65	8.03E-03	1.18E-01	41.02	-0.39	0.48	-0.82	0.415	1.000
ENST00000367330	TMEM9	11.40	-3.19	1.22	-2.62	8.74E-03	1.25E-01	644.44	0.89	0.68	1.31	0.190	0.884
ENST00000394642	TMEM98	20.09	-7.46	1.89	-3.95	7.94E-05	2.70E-03	521.34	0.23	0.34	0.68	0.499	1.000
ENST00000548223	TMPO	15.90	-5.06	1.76	-2.88	3.99E-03	7.13E-02	48.61	-0.02	0.53	-0.04	0.968	1.000
ENST00000233143	TMSB10	3352.94	-1.18	0.20	-5.75	9.01E-09	4.60E-07	44877.28	0.04	0.17	0.25	0.803	1.000
ENST00000533602	TMX2	7.14	-4.76	1.84	-2.59	9.55E-03	1.33E-01	10.11	-0.04	0.82	-0.05	0.957	1.000
ENST00000299608	TMX3	313.29	-0.66	0.25	-2.69	7.18E-03	1.10E-01	14637.24	0.17	0.17	0.95	0.342	0.990
ENST00000341037	TNC	303.00	-4.76	1.33	-3.57	3.61E-04	1.01E-02	45007.45	0.64	0.12	5.27	0.000	0.000
ENST00000537320	TNC	54.70	-23.64	3.03	-7.81	5.65E-15	2.01E-12	15685.21	0.41	0.14	2.85	0.004	0.116
ENST00000274456	TNFAIP8	8.31	-19.05	3.03	-6.29	3.18E-10	1.69E-08	177.48	-0.13	0.35	-0.37	0.714	1.000
ENST00000539372	TNFRSF1A	6.09	-20.67	3.03	-6.82	9.08E-12	5.34E-10	51.81	0.57	0.80	0.71	0.475	1.000
ENST00000517989	TNKS	6.88	-5.92	1.71	-3.45	5.51E-04	1.46E-02	12.08	-0.88	0.77	-1.13	0.257	0.949
ENST00000371627	TNKS2	66.07	-1.14	0.36	-3.16	1.55E-03	3.39E-02	11391.83	0.05	0.15	0.32	0.751	1.000
ENST00000508762	TNPO1	16.22	-7.15	1.99	-3.59	3.30E-04	9.37E-03	20.78	-0.99	0.73	-1.36	0.173	0.860
ENST00000509030	TNPO1	106.53	-9.87	1.98	-4.99	5.98E-07	2.74E-05	94.16	0.70	0.52	1.35	0.178	0.867
ENST00000366607	TOMM20	551.02	-0.97	0.18	-5.23	1.69E-07	8.08E-06	53548.86	-0.11	0.11	-1.01	0.312	0.980
ENST00000321301	TOMM5	42.16	-23.29	3.03	-7.69	1.42E-14	4.23E-12	3356.85	0.01	0.14	0.06	0.949	1.000
ENST00000544379	TOMM5	16.27	-21.79	3.03	-7.20	6.18E-13	6.95E-11	26.23	-0.23	1.11	-0.21	0.837	1.000
ENST00000284320	TOMM70A	187.02	-0.60	0.22	-2.77	5.63E-03	9.19E-02	9147.59	-0.11	0.12	-0.88	0.381	1.000
ENST00000357601	TOP2A	42.38	-0.93	0.35	-2.69	7.25E-03	1.10E-01	48.94	-1.15	0.79	-1.45	0.146	0.821
ENST00000379858	TOPORS	7.23	-20.88	3.03	-6.89	5.56E-12	3.54E-10	354.46	-0.80	0.57	-1.40	0.162	0.845
ENST00000504937	TP53	6.79	-20.82	3.03	-6.87	6.37E-12	3.96E-10	14.39	0.87	1.55	0.56	0.576	1.000
ENST00000391878	TP53BP2	112.92	-0.63	0.20	-3.07	2.14E-03	4.41E-02	5322.61	-0.64	0.94	-0.68	0.497	1.000
ENST00000378292	TPM2	31.23	-1.48	0.47	-3.13	1.75E-03	3.74E-02	4263.34	0.62	0.13	4.59	0.000	0.000
ENST00000368533	TPM3	55.54	-23.66	3.03	-7.82	5.35E-15	1.98E-12	4045.07	1.23	0.23	5.28	0.000	0.000
ENST00000505010	TPM3	19.52	-22.25	3.03	-7.35	2.01E-13	2.92E-11	16.08	0.14	0.76	0.19	0.853	1.000

ENST00000309983	TPMT	40.25	-1.22	0.31	-3.96	7.55E-05	2.59E-03	5617.13	0.18	0.17	1.05	0.293	0.967
ENST00000299427	TPP1	11.05	-21.47	3.03	-7.09	1.34E-12	1.22E-10	84.44	0.98	1.64	0.60	0.551	1.000
ENST00000376065	TPP2	11.76	-21.56	3.03	-7.12	1.09E-12	1.03E-10	1595.85	0.01	0.31	0.04	0.971	1.000
ENST00000367478	TPR	976.28	-0.57	0.22	-2.58	9.74E-03	1.35E-01	46756.99	-0.10	0.14	-0.71	0.476	1.000
ENST00000379055	TPT1	151.61	-1.54	0.33	-4.67	3.04E-06	1.31E-04	1400.52	1.23	0.92	1.33	0.183	0.876
ENST00000300403	TPX2	28.01	-22.73	3.03	-7.51	5.90E-14	1.21E-11	28135.97	-0.23	0.12	-2.02	0.044	0.490
ENST00000297071	TRA2A	156.51	-0.85	0.23	-3.76	1.69E-04	5.23E-03	1904.22	-0.03	0.16	-0.20	0.844	1.000
ENST00000414862	TRA2B	14.63	-7.01	2.68	-2.61	9.06E-03	1.29E-01	129.87	-0.21	0.40	-0.53	0.595	1.000
ENST00000521425	TRAM1	192.41	-0.58	0.22	-2.59	9.61E-03	1.33E-01	4608.18	0.19	0.30	0.65	0.513	1.000
ENST00000434101	TRAPPC4	5.54	-20.54	3.03	-6.78	1.22E-11	6.84E-10	29.07	-0.29	0.84	-0.34	0.732	1.000
ENST00000528230	TRAPPC4	111.28	-24.61	3.03	-8.13	4.24E-16	3.12E-13	114.30	0.93	0.86	1.08	0.281	0.961
ENST00000354631	TRDMT1	13.03	-1.27	0.48	-2.65	8.09E-03	1.19E-01	1297.93	-0.40	0.53	-0.76	0.445	1.000
ENST00000343526	TRIM24	69.29	-1.23	0.41	-3.00	2.66E-03	5.23E-02	1466.94	0.10	0.49	0.20	0.841	1.000
ENST00000377194	TRIM27	15.89	-2.45	0.72	-3.39	6.89E-04	1.75E-02	371.99	1.44	0.72	2.01	0.045	0.497
ENST00000358465	TRIM33	66.66	-1.21	0.21	-5.84	5.09E-09	2.63E-07	7154.76	-0.18	0.14	-1.29	0.196	0.892
ENST00000267622	TRIP11	137.52	-0.80	0.30	-2.65	8.00E-03	1.18E-01	5771.59	0.33	0.25	1.32	0.186	0.880
ENST00000389045	TRIP12	21.03	-0.98	0.35	-2.84	4.46E-03	7.74E-02	15977.47	0.09	0.20	0.44	0.658	1.000
ENST00000261884	TRIP4	73.60	-0.65	0.23	-2.79	5.20E-03	8.72E-02	1673.32	-0.08	0.14	-0.59	0.558	1.000
ENST00000203001	TRMT6	74.55	-9.35	2.82	-3.31	9.26E-04	2.23E-02	430.32	-0.03	0.38	-0.07	0.942	1.000
ENST00000251607	TRNT1	79.52	-0.86	0.33	-2.58	9.82E-03	1.36E-01	27.57	0.60	0.84	0.72	0.472	1.000
ENST00000367441	TROVE2	47.45	-8.70	1.28	-6.80	1.04E-11	5.99E-10	340.35	0.04	0.21	0.19	0.846	1.000
ENST00000539834	TRPC4AP	26.46	-7.86	2.86	-2.75	6.02E-03	9.67E-02	336.33	-0.03	0.30	-0.10	0.917	1.000
ENST00000361136	TSC22D2	26.02	-7.84	1.82	-4.31	1.66E-05	6.54E-04	1874.38	0.22	0.27	0.82	0.411	1.000
ENST00000543727	TSFM	5.58	-5.62	1.74	-3.22	1.26E-03	2.88E-02	204.05	-0.19	0.43	-0.44	0.662	1.000
ENST00000476913	TSNAX	18.68	-2.27	0.78	-2.91	3.66E-03	6.69E-02	184.26	0.19	0.35	0.56	0.573	1.000
ENST00000423920	TSPAN3	12.84	-21.68	3.03	-7.16	8.11E-13	8.26E-11	54.41	-3.40	1.96	-1.73	0.083	0.667
ENST00000243344	TTC21B	20.46	-1.45	0.47	-3.06	2.21E-03	4.51E-02	5053.31	-0.02	0.17	-0.12	0.906	1.000
ENST00000397906	TTC28	45.00	-0.94	0.25	-3.72	2.01E-04	6.13E-03	7605.64	-0.19	0.12	-1.63	0.103	0.731
ENST00000317571	TTC39C	46.74	-0.87	0.31	-2.78	5.39E-03	8.92E-02	3380.75	-0.25	0.34	-0.72	0.474	1.000
ENST00000301071	TUBA1A	592.55	-4.93	1.83	-2.69	7.09E-03	1.08E-01	1435.94	0.33	0.25	1.34	0.181	0.873
ENST00000548363	TUBA1A	174.21	-4.57	0.66	-6.95	3.60E-12	2.56E-10	56.80	-0.57	0.53	-1.07	0.286	0.963
ENST00000336023	TUBA1B	4316.56	-2.17	0.58	-3.74	1.87E-04	5.78E-03	1055.07	-1.06	0.44	-2.44	0.015	0.260
ENST00000333628	TUBB2A	104.88	-1.21	0.29	-4.14	3.53E-05	1.30E-03	5625.72	0.19	0.18	1.05	0.294	0.969
ENST00000510836	TUSC3	44.19	-23.33	3.03	-7.71	1.27E-14	3.90E-12	74.46	-0.04	0.62	-0.06	0.951	1.000
ENST00000547459	TWF1	10.49	-21.40	3.03	-7.07	1.59E-12	1.39E-10	3848.39	-1.47	0.88	-1.67	0.095	0.712
ENST00000374517	TXN	691.61	-1.56	0.37	-4.18	2.91E-05	1.10E-03	12208.84	-0.08	0.17	-0.45	0.651	1.000
ENST00000379757	TXNDC5	194.42	-3.01	1.07	-2.83	4.66E-03	7.98E-02	9576.94	0.45	0.70	0.64	0.520	1.000
ENST00000425134	TXNIP	13.15	-5.67	1.16	-4.89	1.01E-06	4.53E-05	57.57	0.22	0.69	0.31	0.754	1.000
ENST00000526580	TXNRD1	20.06	-7.46	1.10	-6.79	1.10E-11	6.30E-10	170.53	0.38	0.37	1.03	0.305	0.977
ENST00000540716	TXNRD1	14.42	-6.98	2.63	-2.65	7.95E-03	1.18E-01	52.05	0.12	0.66	0.19	0.852	1.000
ENST00000410344	U2	63.85	-1.48	0.45	-3.29	1.01E-03	2.39E-02	3.66	-0.41	1.57	-0.26	0.793	NA
ENST00000398137	U2AF1	25.52	-6.65	1.62	-4.11	4.02E-05	1.46E-03	124.22	0.37	0.32	1.16	0.246	0.937
ENST00000464750	U2AF1	29.79	-8.03	1.92	-4.19	2.78E-05	1.05E-03	51.34	-0.67	1.82	-0.37	0.713	1.000
ENST00000322244	UBA6	103.25	-0.85	0.31	-2.75	6.00E-03	9.66E-02	7852.21	-0.06	0.16	-0.41	0.685	1.000
ENST00000271877	UBAP2L	21.68	-4.92	1.73	-2.84	4.50E-03	7.79E-02	11945.38	0.66	0.61	1.08	0.279	0.961
ENST00000395837	UBB	51.28	-3.74	1.21	-3.10	1.94E-03	4.05E-02	806.45	-0.27	0.33	-0.83	0.406	1.000
ENST00000506787	UBE2B	10.36	-2.70	0.90	-2.99	2.80E-03	5.44E-02	47.10	0.87	0.56	1.56	0.119	0.771
ENST00000445950	UBE2K	6.23	-20.70	3.03	-6.83	8.46E-12	5.06E-10	1926.99	0.20	0.22	0.90	0.366	1.000
ENST00000487227	UBE2T	8.58	-21.14	3.03	-6.98	3.00E-12	2.24E-10	2164.82	-0.68	0.63	-1.08	0.280	0.961
ENST00000371674	UBE2V1	37.30	-23.13	3.03	-7.64	2.17E-14	5.96E-12	3026.33	-0.17	1.11	-0.15	0.878	1.000
ENST00000483534	UBE2V1	12.31	-21.62	3.03	-7.14	9.37E-13	9.16E-11	70.45	0.07	0.44	0.16	0.875	1.000
ENST00000490555	UBE2V1	75.38	-24.05	3.03	-7.95	1.94E-15	1.00E-12	246.05	-0.10	0.26	-0.39	0.699	1.000
ENST00000565634	UBFD1	8.16	-21.04	3.03	-6.95	3.78E-12	2.63E-10	101.86	0.68	0.36	1.88	0.060	0.573
ENST00000565494	UBL7	8.55	-21.11	3.03	-6.97	3.16E-12	2.31E-10	122.76	0.42	0.31	1.36	0.175	0.864
ENST00000553674	UBR7	56.74	-8.96	1.11	-8.09	6.05E-16	3.97E-13	87.78	1.77	1.23	1.44	0.151	0.828
ENST00000272638	UBXN4	153.10	-0.84	0.25	-3.42	6.26E-04	1.62E-02	14301.76	-0.30	0.27	-1.13	0.259	0.950
ENST00000519246	UBXN8	11.76	-3.31	0.69	-4.81	1.52E-06	6.71E-05	739.68	0.11	0.16	0.72	0.474	1.000
ENST00000505232	UCHL1	29.53	-22.79	3.03	-7.53	5.15E-14	1.11E-11	84.45	-0.19	0.64	-0.30	0.765	1.000

ENST00000508768	UCHL1	43.71	-23.34	3.03	-7.71	1.25E-14	3.84E-12	2053.37	-0.19	0.14	-1.38	0.168	0.854
ENST00000510566	UCHL1	7.15	-2.13	0.72	-2.98	2.92E-03	5.63E-02	211.98	-0.48	0.22	-2.17	0.030	0.403
ENST00000420791	UCHL5	33.32	-6.51	1.74	-3.73	1.88E-04	5.80E-03	207.94	0.18	0.64	0.27	0.784	1.000
ENST00000367879	UCK2	224.95	-1.13	0.27	-4.25	2.18E-05	8.45E-04	5367.39	-0.20	0.17	-1.22	0.221	0.917
ENST00000372212	UCK2	6.39	-20.73	3.03	-6.84	7.83E-12	4.75E-10	56.23	-0.79	0.40	-1.97	0.048	0.517
ENST00000368003	UFC1	77.81	-1.57	0.49	-3.19	1.40E-03	3.13E-02	9158.42	0.07	0.12	0.58	0.560	1.000
ENST00000421968	UFD1L	16.32	-22.00	3.03	-7.27	3.67E-13	4.69E-11	15.60	-0.26	0.87	-0.30	0.762	1.000
ENST00000369278	UFL1	74.90	-1.34	0.37	-3.65	2.64E-04	7.78E-03	4403.84	0.01	0.14	0.10	0.917	1.000
ENST00000239878	UFM1	15.40	-7.08	1.79	-3.96	7.46E-05	2.56E-03	881.55	-0.39	0.15	-2.64	0.008	0.182
ENST00000262952	UHRF1	40.99	-21.62	3.03	-7.14	9.18E-13	9.12E-11	5917.83	-0.01	0.32	-0.03	0.973	1.000
ENST00000455180	UHRF1	7.99	-21.03	3.03	-6.94	3.88E-12	2.67E-10	843.16	-2.41	1.13	-2.14	0.033	0.420
ENST00000279907	UHRF1BP1L	39.68	-1.02	0.34	-3.02	2.50E-03	5.01E-02	2694.74	-1.07	1.50	-0.71	0.477	1.000
ENST00000242576	UNG	12.61	-6.79	2.60	-2.61	9.12E-03	1.29E-01	750.82	-0.06	0.81	-0.07	0.940	1.000
ENST00000356352	UPF2	75.62	-0.80	0.22	-3.57	3.51E-04	9.87E-03	962.26	-3.04	2.37	-1.28	0.200	0.895
ENST00000417464	UPP1	7.76	-20.99	3.03	-6.93	4.21E-12	2.85E-10	31.89	-0.49	0.52	-0.95	0.344	0.991
ENST00000429491	UPP1	8.67	-21.15	3.03	-6.98	2.92E-12	2.20E-10	1849.79	0.20	0.20	1.02	0.309	0.979
ENST00000304863	UQCRFS1	252.20	-0.74	0.25	-2.93	3.42E-03	6.36E-02	5908.79	-0.01	0.13	-0.09	0.928	1.000
ENST00000496429	UQCRQ	28.44	-7.96	2.73	-2.92	3.53E-03	6.52E-02	289.80	0.23	0.26	0.88	0.380	1.000
ENST00000369815	USMG5	36.37	-7.74	2.39	-3.24	1.19E-03	2.74E-02	680.19	0.31	0.20	1.55	0.122	0.777
ENST00000218348	USP11	54.93	-8.91	2.58	-3.46	5.44E-04	1.44E-02	888.88	-0.03	0.67	-0.04	0.969	1.000
ENST00000261601	USP14	327.11	-1.76	0.44	-3.97	7.28E-05	2.50E-03	3516.18	0.47	0.41	1.14	0.253	0.945
ENST00000547317	USP15	12.17	-21.17	3.03	-6.99	2.72E-12	2.07E-10	41.11	-0.23	0.45	-0.51	0.610	1.000
ENST00000434032	USP19	12.48	-21.64	3.03	-7.15	8.94E-13	8.95E-11	5361.58	0.79	0.33	2.38	0.017	0.290
ENST00000261497	USP22	33.44	-8.20	2.74	-2.99	2.76E-03	5.38E-02	632.22	-1.28	0.27	-4.77	0.000	0.000
ENST00000559192	USP3	53.78	-2.35	0.54	-4.36	1.30E-05	5.18E-04	2044.74	0.25	0.30	0.83	0.408	1.000
ENST00000527733	USP47	6.77	-20.78	3.03	-6.86	6.93E-12	4.26E-10	262.07	0.41	0.98	0.42	0.677	1.000
ENST00000567329	USP7	2.83	-4.63	1.37	-3.37	7.52E-04	1.88E-02	39.67	-0.83	0.56	-1.48	0.138	0.807
ENST00000307179	USP8	8.99	-21.19	3.03	-7.00	2.62E-12	2.03E-10	252.23	-0.11	0.24	-0.48	0.633	1.000
ENST00000378308	USP9X	6.51	-5.84	1.02	-5.70	1.18E-08	6.01E-07	14753.68	0.05	0.14	0.33	0.744	1.000
ENST00000296792	UTP15	47.92	-2.02	0.38	-5.27	1.39E-07	6.70E-06	1289.87	0.56	0.56	1.00	0.315	0.980
ENST00000508506	UTP18	26.31	-22.65	3.03	-7.48	7.35E-14	1.41E-11	4216.22	-0.07	0.12	-0.59	0.552	1.000
ENST00000261637	UTP20	217.29	-1.01	0.17	-5.83	5.66E-09	2.92E-07	10208.78	-0.07	0.14	-0.50	0.618	1.000
ENST00000484661	UTP6	32.03	-22.91	3.03	-7.57	3.75E-14	8.75E-12	213.01	-0.37	0.28	-1.35	0.176	0.865
ENST00000409501	UXS1	31.74	-5.14	1.85	-2.78	5.36E-03	8.88E-02	3433.02	0.15	0.20	0.74	0.457	1.000
ENST00000262640	VAMP7	7.67	-20.95	3.03	-6.92	4.64E-12	3.07E-10	1897.70	1.44	0.68	2.13	0.033	0.424
ENST00000265619	VAPB	36.08	-3.34	1.26	-2.65	8.10E-03	1.19E-01	288.86	0.26	0.37	0.71	0.479	1.000
ENST00000474643	VARS	18.72	-7.36	1.83	-4.03	5.52E-05	1.95E-03	87.73	0.11	0.66	0.17	0.866	1.000
ENST00000286428	VBP1	258.23	-1.95	0.57	-3.45	5.53E-04	1.46E-02	3121.84	-0.07	0.13	-0.51	0.608	1.000
ENST00000459836	VBP1	3.63	-4.35	1.48	-2.95	3.19E-03	6.00E-02	329.13	0.40	0.38	1.05	0.293	0.968
ENST00000439229	VDAC1P1	468.30	-0.74	0.19	-3.84	1.23E-04	3.97E-03	125.35	-0.23	0.35	-0.65	0.515	1.000
ENST00000258963	VEZF1	71.68	-1.28	0.47	-2.71	6.73E-03	1.04E-01	18434.75	0.06	0.11	0.51	0.609	1.000
ENST00000469543	VIM	4353.04	-3.84	0.92	-4.16	3.15E-05	1.18E-03	28482.58	-0.38	0.51	-0.73	0.466	1.000
ENST00000487938	VIM	446.08	-26.51	3.03	-8.76	1.97E-18	3.29E-15	16904.68	3.43	1.43	2.41	0.016	0.277
ENST00000489794	VPS26A	26.16	-21.56	3.03	-7.12	1.07E-12	1.02E-10	14.20	-0.34	0.82	-0.41	0.680	1.000
ENST00000546041	VPS26A	65.41	-9.17	1.57	-5.85	4.80E-09	2.48E-07	78.15	-0.57	0.34	-1.64	0.100	0.723
ENST00000531032	VPS28	6.73	-20.80	3.03	-6.86	6.68E-12	4.14E-10	19.35	-0.26	0.62	-0.43	0.669	1.000
ENST00000533806	VPS28	11.34	-6.64	1.81	-3.67	2.45E-04	7.28E-03	259.21	0.07	0.39	0.17	0.867	1.000
ENST00000419023	VPS45	7.63	-20.97	3.03	-6.92	4.47E-12	2.98E-10	225.75	-0.40	0.55	-0.72	0.474	1.000
ENST00000555351	VRK1	7.99	-21.04	3.03	-6.94	3.80E-12	2.64E-10	264.91	-0.96	0.72	-1.34	0.180	0.872
ENST00000427932	VTA1	25.39	-22.60	3.03	-7.47	8.21E-14	1.54E-11	3117.10	0.43	0.30	1.45	0.147	0.822
ENST00000480474	WAC	3.19	-4.81	1.66	-2.90	3.73E-03	6.80E-02	139.05	0.35	0.41	0.84	0.400	1.000
ENST00000392882	WARS	42.83	-7.40	2.17	-3.41	6.54E-04	1.68E-02	707.34	0.66	0.40	1.66	0.096	0.714
ENST00000261167	WBP11	121.97	-0.64	0.20	-3.25	1.15E-03	2.66E-02	13173.74	-0.10	0.11	-0.88	0.376	1.000
ENST00000372661	WBP5	65.39	-2.87	0.55	-5.21	1.84E-07	8.77E-06	4057.37	0.09	0.14	0.69	0.491	1.000
ENST00000421744	WBSCR22	6.51	-20.72	3.03	-6.84	7.98E-12	4.81E-10	72.04	-1.77	1.02	-1.73	0.083	0.668
ENST00000487006	WBSCR22	16.51	-21.66	3.03	-7.15	8.37E-13	8.49E-11	626.30	-1.14	0.74	-1.53	0.126	0.785
ENST00000502702	WDR1	32.24	-22.92	3.03	-7.57	3.67E-14	8.62E-12	231.93	-0.06	0.56	-0.10	0.920	1.000
ENST00000508079	WDR1	112.71	-5.95	2.17	-2.74	6.10E-03	9.77E-02	48.70	-0.01	0.54	-0.01	0.991	1.000

ENST00000261015	WDR12	225.39	-0.78	0.26	-3.04	2.33E-03	4.71E-02	8748.48	-0.06	0.12	-0.47	0.635	1.000
ENST00000471680	WDR3	5.93	-20.63	3.03	-6.81	9.84E-12	5.72E-10	605.79	0.18	0.43	0.43	0.671	1.000
ENST00000505303	WDR36	57.10	-8.97	2.81	-3.19	1.41E-03	3.14E-02	65.89	0.17	0.50	0.33	0.743	1.000
ENST00000506474	WDR41	3.99	-5.13	1.85	-2.78	5.44E-03	8.97E-02	11.01	-0.82	1.12	-0.73	0.462	1.000
ENST00000371822	WDR44	17.53	-7.27	2.67	-2.72	6.45E-03	1.01E-01	93.37	-2.73	0.95	-2.88	0.004	0.106
ENST00000392325	WDR45L	82.40	-1.40	0.31	-4.46	8.29E-06	3.38E-04	16502.75	-0.10	0.12	-0.86	0.389	1.000
ENST00000563381	WDR59	3.09	-4.77	1.08	-4.42	9.76E-06	3.96E-04	4.36	-1.18	1.90	-0.62	0.537	1.000
ENST00000471162	WDR6	60.07	-22.97	3.03	-7.59	3.21E-14	7.78E-12	101.39	0.13	0.31	0.41	0.684	1.000
ENST00000436347	WDR75	18.33	-22.16	3.03	-7.32	2.50E-13	3.48E-11	87.71	-0.91	0.43	-2.09	0.037	0.448
ENST00000472286	WDR75	16.26	-7.16	1.81	-3.96	7.50E-05	2.57E-03	571.92	-0.35	0.32	-1.09	0.276	0.959
ENST00000411751	WDR77	8.35	-21.08	3.03	-6.96	3.43E-12	2.46E-10	270.62	4.88	1.70	2.86	0.004	0.112
ENST00000449340	WDR77	61.48	-9.08	2.82	-3.22	1.29E-03	2.93E-02	63.65	0.49	0.83	0.60	0.551	1.000
ENST00000549091	WDR90	3.56	-3.12	1.14	-2.73	6.36E-03	1.01E-01	539.38	-5.54	1.74	-3.18	0.001	0.051
ENST00000474411	WDR91	3.44	-4.91	1.10	-4.48	7.61E-06	3.11E-04	39.09	0.02	0.62	0.04	0.971	1.000
ENST00000523984	WDYHV1	4.93	-5.44	1.75	-3.11	1.88E-03	3.95E-02	156.67	0.13	0.27	0.46	0.644	1.000
ENST00000508803	WHSC1	46.75	-8.68	2.75	-3.15	1.61E-03	3.49E-02	238.75	-0.94	0.31	-3.00	0.003	0.079
ENST00000380764	WRNIP1	9.24	-21.23	3.03	-7.01	2.40E-12	1.90E-10	5769.16	0.07	0.13	0.54	0.591	1.000
ENST00000540129	WSB2	6.08	-5.13	1.42	-3.61	3.06E-04	8.83E-03	156.75	0.13	0.28	0.47	0.642	1.000
ENST00000484256	XBP1	20.09	-1.01	0.35	-2.85	4.31E-03	7.56E-02	4308.68	-0.42	0.24	-1.76	0.078	0.648
ENST00000369658	XPNPEP1	22.14	-7.60	1.82	-4.18	2.88E-05	1.09E-03	1443.89	0.05	0.19	0.26	0.793	1.000
ENST00000443240	XPO1	6.18	-5.76	2.02	-2.85	4.36E-03	7.63E-02	172.71	-1.76	1.18	-1.48	0.138	0.806
ENST00000400602	XPO4	40.14	-0.96	0.36	-2.63	8.53E-03	1.23E-01	158.89	0.21	0.41	0.50	0.619	1.000
ENST00000332707	XPOT	196.08	-0.89	0.34	-2.63	8.52E-03	1.23E-01	28065.37	-0.06	0.11	-0.56	0.572	1.000
ENST00000392132	XRCC5	501.39	-26.67	3.03	-8.81	1.24E-18	2.25E-15	45007.31	0.22	0.18	1.24	0.214	0.910
ENST00000460284	XRCC5	27.27	-7.90	2.85	-2.77	5.56E-03	9.11E-02	7828.55	-0.95	0.45	-2.13	0.033	0.425
ENST00000360079	XRCC6	412.04	-11.82	2.14	-5.54	3.08E-08	1.53E-06	28348.08	0.01	0.25	0.02	0.983	1.000
ENST00000405506	XRCC6	59.36	-23.75	3.03	-7.85	4.24E-15	1.73E-12	1274.34	0.03	0.44	0.08	0.939	1.000
ENST00000428575	XRCC6	56.75	-23.69	3.03	-7.83	4.93E-15	1.90E-12	2417.95	0.11	0.27	0.42	0.673	1.000
ENST00000464116	XRCC6	4.84	-5.41	1.69	-3.21	1.33E-03	2.99E-02	14.87	-0.56	1.15	-0.48	0.631	1.000
ENST00000300145	XRCC6BP1	5.07	-5.47	1.04	-5.26	1.47E-07	7.05E-06	806.58	0.17	0.64	0.27	0.787	1.000
ENST00000377191	XRN2	715.07	-0.52	0.18	-2.95	3.13E-03	5.93E-02	13752.69	-0.35	0.12	-2.87	0.004	0.110
ENST00000207870	XYLB	4.00	-5.13	1.80	-2.86	4.23E-03	7.46E-02	1060.23	0.24	0.49	0.48	0.629	1.000
ENST00000469100	YARS	26.67	-7.87	2.48	-3.18	1.48E-03	3.26E-02	938.83	-0.09	0.20	-0.44	0.660	1.000
ENST00000470377	YARS	6.76	-3.11	1.04	-3.00	2.67E-03	5.24E-02	270.05	-0.29	0.26	-1.10	0.271	0.956
ENST00000478828	YARS	24.87	-22.57	3.03	-7.46	8.84E-14	1.62E-11	848.30	0.11	0.25	0.42	0.672	1.000
ENST00000487404	YARS	14.78	-4.21	1.55	-2.72	6.57E-03	1.03E-01	2342.88	-0.22	0.23	-0.93	0.353	0.995
ENST00000332220	YBX1	14.91	-21.88	3.03	-7.23	4.96E-13	5.93E-11	980.52	-0.62	0.24	-2.51	0.012	0.230
ENST00000305135	YEATS2	16.53	-7.18	1.13	-6.33	2.38E-10	1.28E-08	7681.48	-0.03	0.14	-0.21	0.830	1.000
ENST00000416380	YIPF3	31.34	-8.11	0.96	-8.41	4.25E-17	4.15E-14	25.34	-0.12	0.60	-0.21	0.836	1.000
ENST00000437765	YIPF4	7.50	-6.04	1.71	-3.53	4.22E-04	1.16E-02	20.50	2.00	2.00	1.00	0.319	0.983
ENST00000376016	YME1L1	97.48	-24.43	3.03	-8.07	6.90E-16	4.38E-13	10194.82	-0.28	0.22	-1.29	0.198	0.894
ENST00000355665	YTHDC1	4.44	-5.28	1.71	-3.09	2.00E-03	4.18E-02	1440.30	0.56	0.66	0.86	0.392	1.000
ENST00000477896	YWHAB	105.84	-2.51	0.61	-4.09	4.29E-05	1.55E-03	215.60	-0.35	0.27	-1.28	0.200	0.895
ENST00000486241	YWHAE	30.56	-4.76	1.58	-3.02	2.56E-03	5.09E-02	38.40	-0.04	0.51	-0.08	0.935	1.000
ENST00000373659	ZBTB6	46.81	-0.85	0.26	-3.28	1.03E-03	2.43E-02	2888.82	0.00	0.16	-0.03	0.976	1.000
ENST00000473294	ZBTB8OS	29.14	-3.86	1.28	-3.02	2.57E-03	5.09E-02	162.64	0.38	0.25	1.49	0.137	0.806
ENST00000483138	ZBTB8OS	7.12	-20.85	3.03	-6.88	5.97E-12	3.76E-10	104.52	0.89	0.49	1.83	0.068	0.608
ENST00000555799	ZC3H14	6.90	-5.92	1.84	-3.22	1.30E-03	2.94E-02	59.61	-2.69	1.64	-1.64	0.102	0.727
ENST00000337859	ZC3H15	288.60	-0.78	0.20	-3.95	7.70E-05	2.63E-03	5659.62	0.11	0.18	0.65	0.514	1.000
ENST00000498757	ZC3H15	13.13	-6.85	2.60	-2.63	8.53E-03	1.23E-01	687.19	0.15	0.26	0.57	0.566	1.000
ENST00000409573	ZC3H8	5.77	-5.66	1.72	-3.29	1.00E-03	2.38E-02	1066.84	0.02	0.41	0.04	0.970	1.000
ENST00000460845	ZC3HAV1	15.46	-7.09	2.60	-2.73	6.34E-03	1.00E-01	121.19	1.28	0.38	3.34	0.001	0.032
ENST00000531722	ZCCHC11	8.68	-6.25	1.11	-5.65	1.64E-08	8.26E-07	828.86	0.05	0.27	0.17	0.861	1.000
ENST00000269499	ZCCHC2	31.12	-0.70	0.27	-2.61	9.18E-03	1.30E-01	5146.56	-0.12	0.12	-1.04	0.297	0.970
ENST00000542645	ZDHHC20	6.64	-20.76	3.03	-6.85	7.37E-12	4.51E-10	187.96	0.15	0.42	0.36	0.715	1.000
ENST00000558170	ZEB2	13.52	-21.75	3.03	-7.18	6.84E-13	7.33E-11	7005.04	0.15	0.18	0.81	0.415	1.000
ENST00000521895	ZFAND1	8.02	-6.14	2.11	-2.91	3.59E-03	6.61E-02	82.40	0.10	0.95	0.10	0.917	1.000
ENST00000261749	ZFAND6	11.50	-6.66	1.90	-3.50	4.72E-04	1.27E-02	308.68	1.27	0.22	5.76	0.000	0.000

ENST00000558688	ZFAND6	6.79	-5.30	2.03	-2.62	8.82E-03	1.26E-01	67.37	0.71	0.63	1.12	0.263	0.952
ENST00000561017	ZFAND6	11.83	-6.70	2.60	-2.58	9.90E-03	1.36E-01	86.24	0.46	0.61	0.75	0.451	1.000
ENST00000507465	ZFR	8.53	-21.13	3.03	-6.97	3.08E-12	2.28E-10	473.76	-0.31	0.28	-1.12	0.261	0.952
ENST00000357540	ZFYVE27	6.63	-5.86	1.69	-3.47	5.25E-04	1.40E-02	77.61	-0.56	3.86	-0.14	0.885	1.000
ENST00000493328	ZMYM6	18.92	-1.64	0.55	-2.98	2.92E-03	5.63E-02	633.97	-0.09	0.23	-0.39	0.697	1.000
ENST00000320451	ZNF121	45.77	-4.14	1.33	-3.12	1.82E-03	3.86E-02	2373.46	-0.51	0.15	-3.46	0.001	0.023
ENST00000360647	ZNF148	89.81	-0.65	0.24	-2.72	6.47E-03	1.02E-01	3825.66	0.01	0.23	0.03	0.976	1.000
ENST00000523638	ZNF260	8.88	-1.40	0.53	-2.62	8.72E-03	1.25E-01	4490.86	0.10	0.13	0.75	0.451	1.000
ENST00000334197	ZNF347	2.81	-3.31	1.17	-2.84	4.52E-03	7.82E-02	1997.65	0.23	0.20	1.15	0.250	0.941
ENST00000397148	ZNF43	6.13	-1.68	0.64	-2.64	8.41E-03	1.22E-01	390.53	0.19	0.64	0.29	0.771	1.000
ENST00000425708	ZNF445	19.53	-1.03	0.32	-3.19	1.44E-03	3.20E-02	3991.11	-0.86	0.85	-1.01	0.313	0.980
ENST00000294753	ZNF496	4.11	-5.17	1.83	-2.83	4.61E-03	7.92E-02	12365.44	-0.21	0.16	-1.28	0.201	0.896
ENST00000482709	ZNF644	4.74	-5.38	1.23	-4.38	1.21E-05	4.84E-04	23.69	0.01	0.66	0.01	0.989	1.000
ENST00000366503	ZNF670	18.59	-1.16	0.34	-3.41	6.48E-04	1.67E-02	1496.61	0.12	0.15	0.80	0.422	1.000
ENST00000418100	ZNF724P	21.70	-1.41	0.34	-4.18	2.96E-05	1.11E-03	895.77	-0.32	0.18	-1.78	0.075	0.637
ENST00000552593	ZNF740	5.77	-5.67	1.12	-5.08	3.70E-07	1.72E-05	736.98	-0.03	0.17	-0.17	0.867	1.000
ENST00000356321	ZNF770	66.06	-0.92	0.34	-2.71	6.66E-03	1.04E-01	8709.84	0.03	0.14	0.20	0.841	1.000
ENST00000508784	ZNF827	30.04	-0.97	0.30	-3.22	1.27E-03	2.89E-02	12928.16	-0.16	0.19	-0.84	0.402	1.000
ENST00000446801	ZWILCH	14.15	-21.79	3.03	-7.20	6.13E-13	6.95E-11	1496.57	-1.21	0.84	-1.44	0.150	0.825
ENST00000535141	ZWILCH	21.39	-7.55	2.65	-2.85	4.37E-03	7.64E-02	589.16	0.57	2.62	0.22	0.829	1.000
ENST00000569489	ZWILCH	24.12	-0.89	0.31	-2.85	4.41E-03	7.68E-02	18.42	0.13	1.27	0.10	0.920	1.000
ENST00000336332	ZXDC	12.45	-6.77	2.61	-2.60	9.42E-03	1.32E-01	1461.72	-0.19	0.68	-0.28	0.779	1.000
ENST00000449423	ZYX	8.75	-20.25	3.03	-6.68	2.34E-11	1.29E-09	3380.21	-1.76	0.88	-2.00	0.046	0.501

B. Ribo-seq and RNA-seq fold-change and p-values for RNAs with higher translational efficiency in LN229 NSUN5 deficient cells in the presence of H_2O_2 stress

		Ribo-Seq						RNA-Seq					
**EnsemblID**	**GeneName**	**Base Mean**	**log2Fold Change**	**Lfc SE**	**stat**	**pvalue**	**padj**	**Base Mean**	**log2Fold Change**	**Lfc SE**	**stat**	**pvalue**	**padj**
ENST00000516388	AC091047.1	72.59	-3.50	1.13	-3.10	1.94E-03	3.89E-01	76.64	-0.33	0.25	-1.29	0.20	1.00
ENST00000480301	ACTB	22.88	-3.57	1.27	-2.81	4.93E-03	8.07E-01	6.35	-0.35	0.77	-0.46	0.65	1.00
ENST00000481423	AHCYL1	7.15	-21.32	3.03	-7.03	2.02E-12	1.07E-09	24.45	-0.58	0.44	-1.33	0.18	0.99
ENST00000529701	APLP2	29.16	-8.46	1.73	-4.88	1.09E-06	2.91E-04	12.10	-2.22	1.49	-1.49	0.14	0.93
ENST00000368233	APOA1BP	2.50	-4.87	1.65	-2.95	3.13E-03	5.73E-01	4035.29	0.30	0.18	1.69	0.09	0.85
ENST00000522919	ASPH	7.45	-21.37	3.03	-7.05	1.79E-12	1.05E-09	220.04	0.61	0.42	1.48	0.14	0.94
ENST00000339159	ATP1A1	65.20	-24.33	3.03	-8.04	9.15E-16	5.52E-12	8828.89	-0.78	0.47	-1.68	0.09	0.85
ENST00000537345	ATP1A1	49.76	-23.85	3.03	-7.88	3.29E-15	1.21E-11	73750.46	-0.11	0.36	-0.31	0.75	1.00
ENST00000367815	ATP1B1	6.12	-21.09	3.03	-6.95	3.56E-12	1.50E-09	7530.34	0.20	1.07	0.18	0.85	1.00
ENST00000460085	ATP6V1E1	12.57	-22.10	3.03	-7.29	3.01E-13	2.56E-10	6.06	3.47	1.02	3.42	0.00	0.04
ENST00000404251	BAZ1B	12.68	-22.11	3.03	-7.30	2.93E-13	2.56E-10	274.78	0.97	0.42	2.32	0.02	0.44
ENST00000301633	BIRC5	11.20	-3.55	1.14	-3.13	1.78E-03	3.65E-01	839.94	-0.22	0.19	-1.14	0.25	1.00
ENST00000353555	BSG	80.03	-24.60	3.03	-8.13	4.32E-16	3.12E-12	43682.70	0.10	0.19	0.55	0.58	1.00
ENST00000379286	C20orf30	19.79	-6.73	2.15	-3.14	1.71E-03	3.54E-01	252.70	-11.45	3.46	-3.31	0.00	0.06
ENST00000472428	C7orf49	16.45	-22.34	3.03	-7.38	1.63E-13	1.60E-10	7.08	-0.64	0.80	-0.80	0.42	1.00
ENST00000463087	CABYR	2.56	-3.72	1.33	-2.79	5.33E-03	8.63E-01	171.74	1.41	0.42	3.39	0.00	0.05
ENST00000367681	CACYBP	30.64	-8.53	2.47	-3.45	5.55E-04	1.29E-01	109.84	0.01	0.51	0.02	0.98	1.00
ENST00000506298	CANX	500.35	-26.91	3.03	-8.89	5.99E-19	7.22E-15	16.11	-0.02	0.59	-0.03	0.98	1.00
ENST00000461993	CAP1	94.02	-10.14	1.85	-5.50	3.83E-08	1.04E-05	68.45	-0.35	0.30	-1.16	0.24	1.00
ENST00000222125	CAPS	5.96	-21.09	3.03	-6.95	3.57E-12	1.50E-09	1443.89	0.59	0.88	0.67	0.50	1.00
ENST00000209875	CBX5	14.08	-1.93	0.67	-2.87	4.14E-03	7.06E-01	45541.74	-0.01	0.10	-0.08	0.93	1.00
ENST00000472524	CCDC88B	2.44	-4.86	1.76	-2.77	5.65E-03	9.00E-01	517.41	-0.03	0.14	-0.19	0.85	1.00
ENST00000493482	CETN2	9.73	-6.28	2.11	-2.98	2.87E-03	5.43E-01	37.81	-0.09	0.34	-0.27	0.79	1.00
ENST00000308162	CFL1	258.07	-2.30	0.80	-2.87	4.17E-03	7.08E-01	38961.45	0.00	0.17	0.03	0.98	1.00
ENST00000535571	CLDN12	4.44	-5.73	1.76	-3.26	1.10E-03	2.39E-01	0.85	-0.65	1.99	-0.33	0.74	NA
ENST00000541297	CLPTM1	6.34	-21.17	3.03	-6.98	2.91E-12	1.35E-09	766.66	0.73	1.54	0.47	0.64	1.00
ENST00000273308	CNPY2	7.45	-4.06	1.46	-2.78	5.46E-03	8.78E-01	488.05	-0.23	0.29	-0.80	0.42	1.00
ENST00000273342	COL8A1	15.82	-22.40	3.03	-7.40	1.40E-13	1.45E-10	13203.28	0.59	1.47	0.40	0.69	1.00

ENST00000449653	COMT	5.99	-6.18	2.20	-2.81	4.93E-03	8.07E-01	2341.49	0.69	0.42	1.65	0.10	0.87
ENST00000462432	CPSF3L	2.52	-4.94	1.74	-2.85	4.42E-03	7.47E-01	91.47	0.12	0.30	0.38	0.70	1.00
ENST00000468255	CSNK2B	24.12	-8.18	3.03	-2.70	6.89E-03	1.00E+00	4408.57	0.14	0.17	0.84	0.40	1.00
ENST00000548783	CSRP2	7.53	-5.91	2.00	-2.96	3.11E-03	5.73E-01	4318.75	0.22	0.31	0.72	0.47	1.00
ENST00000568342	CYB5B	14.83	-2.24	0.67	-3.36	7.80E-04	1.77E-01	10.85	-0.37	0.57	-0.66	0.51	1.00
ENST00000466152	DPM1	18.79	-22.64	3.03	-7.48	7.71E-14	1.14E-10	1420.43	-0.53	1.09	-0.49	0.63	1.00
ENST00000495333	EEF1A1	20.39	-7.97	3.03	-2.63	8.47E-03	1.00E+00	2360.14	-0.26	0.24	-1.06	0.29	1.00
ENST00000256383	EIF2S1	6.56	-5.13	1.85	-2.77	5.64E-03	9.00E-01	15787.39	-0.02	0.12	-0.14	0.89	1.00
ENST00000522352	EIF3E	16.91	-22.49	3.03	-7.43	1.11E-13	1.38E-10	160.41	-0.13	0.29	-0.47	0.64	1.00
ENST00000293831	EIF4A1	50.22	-7.58	2.48	-3.06	2.23E-03	4.37E-01	139472.90	-0.05	0.16	-0.28	0.78	1.00
ENST00000424196	EIF4G1	25.62	-7.11	2.23	-3.19	1.44E-03	3.02E-01	359.17	0.70	0.92	0.76	0.45	1.00
ENST00000525681	EIF4G2	17.62	-22.44	3.03	-7.41	1.25E-13	1.45E-10	35.25	3.51	1.01	3.49	0.00	0.04
ENST00000467800	ELMO2	4.45	-3.27	1.10	-2.98	2.91E-03	5.45E-01	1016.21	-1.51	0.98	-1.54	0.12	0.91
ENST00000539440	EMG1	15.36	-7.53	2.62	-2.88	4.03E-03	6.95E-01	433.45	-0.64	0.50	-1.29	0.20	1.00
ENST00000546220	EMG1	4.52	-5.77	1.60	-3.60	3.15E-04	7.50E-02	224.34	-0.48	1.50	-0.32	0.75	1.00
ENST00000281821	EPHA4	15.49	-1.16	0.45	-2.60	9.38E-03	1.00E+00	859.32	-0.05	1.06	-0.05	0.96	1.00
ENST00000431842	FAM208A	4.05	-5.59	1.67	-3.35	8.18E-04	1.84E-01	8538.92	-0.27	0.15	-1.89	0.06	0.74
ENST00000459993	FAM208A	2.33	-4.82	1.63	-2.95	3.21E-03	5.83E-01	1481.13	-0.10	0.14	-0.76	0.45	1.00
ENST00000505125	FIP1L1	27.56	-3.20	0.86	-3.72	1.95E-04	4.87E-02	170.39	-1.46	1.00	-1.46	0.14	0.94
ENST00000487376	FLOT1	6.44	-21.18	3.03	-6.99	2.82E-12	1.33E-09	2121.85	0.10	1.01	0.10	0.92	1.00
ENST00000541882	FLRT3	6.12	-21.09	3.03	-6.95	3.56E-12	1.50E-09	5861.97	-0.13	0.22	-0.60	0.55	1.00
ENST00000356005	FN1	38.84	-23.62	3.03	-7.80	6.05E-15	1.82E-11	243480.92	-0.02	0.16	-0.16	0.88	1.00
ENST00000443816	FN1	34.24	-23.23	3.03	-7.67	1.68E-14	3.79E-11	2380.49	0.29	0.30	0.97	0.33	1.00
ENST00000568685	FUS	10.20	-21.81	3.03	-7.20	6.10E-13	4.32E-10	509.92	0.22	0.29	0.76	0.45	1.00
ENST00000254108	FUS	20.50	-6.34	2.32	-2.73	6.28E-03	9.68E-01	2544.32	0.11	0.71	0.15	0.88	1.00
ENST00000396861	GAPDH	26.68	-8.33	2.70	-3.08	2.05E-03	4.08E-01	20161.15	-0.37	0.10	-3.90	0.00	0.01
ENST00000380181	GDI2	18.14	-22.59	3.03	-7.46	8.69E-14	1.16E-10	4054.20	-0.07	0.64	-0.10	0.92	1.00
ENST00000492383	GNAI2	10.63	-7.00	2.30	-3.05	2.30E-03	4.50E-01	570.12	-0.09	0.29	-0.31	0.76	1.00
ENST00000482112	GNAS	6.44	-21.18	3.03	-6.99	2.82E-12	1.33E-09	345.96	-0.13	0.97	-0.14	0.89	1.00
ENST00000515417	GNB2L1	10.36	-21.83	3.03	-7.21	5.77E-13	4.26E-10	10512.00	1.09	1.12	0.97	0.33	1.00
ENST00000476321	GSTM3	6.97	-21.30	3.03	-7.03	2.12E-12	1.08E-09	22.39	-0.12	0.64	-0.18	0.86	1.00
ENST00000281543	GUF1	1.72	-4.41	1.70	-2.59	9.72E-03	1.00E+00	589.93	-0.03	0.25	-0.11	0.91	1.00
ENST00000425835	HDAC2	6.95	-6.39	2.25	-2.84	4.56E-03	7.63E-01	25.91	-0.08	0.81	-0.10	0.92	1.00
ENST00000374982	HLA-DRA	24.65	-22.85	3.03	-7.55	4.40E-14	7.66E-11	26057.55	-0.01	0.21	-0.03	0.97	1.00
ENST00000255320	HMGB1P5	5.36	-6.01	2.24	-2.69	7.21E-03	1.00E+00	12352.63	-0.10	0.13	-0.80	0.43	1.00
ENST00000446922	HMGB2	10.06	-21.79	3.03	-7.19	6.43E-13	4.47E-10	1820.92	-0.54	1.21	-0.45	0.66	1.00
ENST00000376256	HNRNPK	8.51	-21.57	3.03	-7.12	1.11E-12	7.19E-10	1999.52	-0.07	0.32	-0.24	0.81	1.00
ENST00000299767	HSP90B1	146.75	-10.79	2.60	-4.15	3.34E-05	8.51E-03	85791.58	0.02	0.24	0.10	0.92	1.00
ENST00000479376	IDH3B	5.12	-5.95	1.64	-3.64	2.77E-04	6.67E-02	3.45	1.29	0.93	1.39	0.16	0.97
ENST00000340979	IFI16	7.59	-6.51	2.40	-2.71	6.65E-03	1.00E+00	691.42	-0.27	0.26	-1.03	0.30	1.00
ENST00000368131	IFI16	7.43	-6.48	2.37	-2.73	6.29E-03	9.68E-01	1664.29	-0.18	0.20	-0.88	0.38	1.00
ENST00000559676	IREB2	2.15	-4.68	1.73	-2.71	6.69E-03	1.00E+00	107.46	0.10	0.56	0.17	0.86	1.00
ENST00000394636	IRX5	3.05	-3.93	1.26	-3.11	1.86E-03	3.78E-01	104.45	0.59	1.00	0.58	0.56	1.00
ENST00000381340	ITPR2	46.59	-1.11	0.32	-3.50	4.60E-04	1.07E-01	14928.61	-1.67	0.15	-11.08	0.00	0.00
ENST00000261247	JKAMP	6.18	-21.11	3.03	-6.96	3.37E-12	1.47E-09	379.83	-0.13	0.38	-0.34	0.73	1.00
ENST00000554271	JKAMP	13.19	-4.34	1.00	-4.34	1.41E-05	3.62E-03	361.28	0.11	0.21	0.56	0.58	1.00
ENST00000330459	KPNA2	30.47	-23.29	3.03	-7.69	1.44E-14	3.48E-11	84177.91	0.02	0.24	0.08	0.93	1.00
ENST00000371335	LAMP2	13.04	-7.29	2.38	-3.07	2.17E-03	4.28E-01	23361.33	0.16	0.17	0.93	0.35	1.00
ENST00000556263	LGALS3	2.55	-4.26	1.26	-3.39	7.05E-04	1.61E-01	14.95	0.33	0.84	0.39	0.70	1.00
ENST00000347559	LMNA	20.47	-22.75	3.03	-7.51	5.78E-14	9.09E-11	5294.09	0.00	1.21	0.00	1.00	1.00
ENST00000480956	LSM5	3.50	-5.42	1.82	-2.97	2.95E-03	5.50E-01	795.87	0.09	0.21	0.42	0.68	1.00
ENST00000523944	MCM4	7.24	-21.35	3.03	-7.04	1.88E-12	1.06E-09	956.70	-0.01	0.49	-0.01	0.99	1.00
ENST00000495083	MDH1	11.40	-1.30	0.49	-2.66	7.81E-03	1.00E+00	296.30	0.79	0.22	3.61	0.00	0.03
ENST00000546753	METAP2	5.11	-20.86	3.03	-6.88	6.12E-12	2.19E-09	289.72	-0.37	0.66	-0.57	0.57	1.00
ENST00000564864	MMP2	14.04	-22.22	3.03	-7.33	2.23E-13	2.12E-10	149.18	-0.16	0.41	-0.40	0.69	1.00
ENST00000535726	NDUFA9	6.27	-6.24	2.26	-2.76	5.79E-03	9.11E-01	147.05	0.52	0.32	1.61	0.11	0.89
ENST00000476279	NDUFB2	7.06	-21.31	3.03	-7.03	2.07E-12	1.08E-09	87.97	1.42	1.69	0.84	0.40	1.00
ENST00000468210	NDUFB5	6.60	-21.21	3.03	-7.00	2.62E-12	1.26E-09	84.68	-1.14	0.80	-1.41	0.16	0.96

ENST00000525440	NME7	4.10	-5.63	1.58	-3.56	3.70E-04	8.74E-02	690.26	-0.60	1.69	-0.35	0.72	1.00
ENST00000517671	NPM1	27.52	-23.15	3.03	-7.65	2.06E-14	4.14E-11	16381.64	-9.42	3.73	-2.53	NA	NA
ENST00000379046	NQO1	12.60	-22.10	3.03	-7.30	2.98E-13	2.56E-10	433.32	0.82	1.03	0.79	0.43	1.00
ENST00000569118	NQO1	64.01	-1.79	0.69	-2.58	9.85E-03	1.00E+00	421.01	-0.14	0.48	-0.30	0.76	1.00
ENST00000569236	NSMCE1	5.67	-6.10	2.36	-2.58	9.86E-03	1.00E+00	138.19	-1.01	1.06	-0.95	0.34	1.00
ENST00000563405	NUP93	7.89	-3.97	1.45	-2.74	6.18E-03	9.60E-01	383.67	0.39	0.35	1.13	0.26	1.00
ENST00000551035	OS9	5.11	-20.86	3.03	-6.88	6.12E-12	2.19E-09	66.16	0.06	0.35	0.17	0.86	1.00
ENST00000354694	PES1	18.40	-4.83	1.46	-3.31	9.22E-04	2.02E-01	3900.34	-0.17	0.39	-0.43	0.67	1.00
ENST00000355899	PLS3	16.24	-22.44	3.03	-7.41	1.28E-13	1.45E-10	11229.32	-0.38	0.94	-0.40	0.69	1.00
ENST00000450367	PPP1R7	5.43	-20.96	3.03	-6.91	4.81E-12	1.88E-09	46.77	-0.08	0.40	-0.21	0.83	1.00
ENST00000412601	PRKCSH	15.66	-6.39	2.26	-2.83	4.63E-03	7.72E-01	3937.77	-2.25	0.85	-2.64	0.01	0.26
ENST00000376033	PRRC2A	6.66	-21.23	3.03	-7.00	2.50E-12	1.22E-09	2542.42	-0.39	0.76	-0.51	0.61	1.00
ENST00000554812	PSMA3	9.48	-6.83	2.34	-2.92	3.52E-03	6.23E-01	3134.88	-0.09	0.29	-0.30	0.76	1.00
ENST00000474100	PSMB4	21.94	-8.05	3.03	-2.66	7.88E-03	1.00E+00	2536.67	-0.40	0.42	-0.95	0.34	1.00
ENST00000395330	PSMB9	2.68	-5.00	1.36	-3.68	2.34E-04	5.76E-02	6.68	0.52	2.96	0.18	0.86	1.00
ENST00000295901	PSMD6	8.31	-21.54	3.03	-7.11	1.20E-12	7.59E-10	7225.36	0.18	0.44	0.40	0.69	1.00
ENST00000544823	QKI	5.48	-20.93	3.03	-6.90	5.16E-12	1.96E-09	6.15	-0.44	1.09	-0.40	0.69	1.00
ENST00000358546	RAB21	5.22	-20.89	3.03	-6.89	5.66E-12	2.09E-09	26.76	0.39	1.04	0.38	0.71	1.00
ENST00000482525	RAB7A	5.06	-20.85	3.03	-6.87	6.33E-12	2.22E-09	11063.39	-0.17	0.23	-0.75	0.45	1.00
ENST00000392369	RAN	45.82	-23.85	3.03	-7.88	3.34E-15	1.21E-11	507.14	0.40	0.34	1.19	0.24	1.00
ENST00000550265	RARG	2.74	-5.06	1.74	-2.90	3.67E-03	6.45E-01	2.77	4.91	3.92	1.25	0.21	1.00
ENST00000488353	RER1	2.49	-4.24	1.20	-3.53	4.12E-04	9.67E-02	7382.61	0.06	0.09	0.63	0.53	1.00
ENST00000458349	RPL28	12.83	-22.12	3.03	-7.30	2.81E-13	2.56E-10	2943.85	0.12	0.33	0.36	0.72	1.00
ENST00000527273	RPS3	5.65	-6.10	2.01	-3.04	2.37E-03	4.61E-01	1768.64	-0.22	0.18	-1.22	0.22	1.00
ENST00000337526	RTN4	30.49	-5.06	1.67	-3.04	2.40E-03	4.62E-01	542.80	-1.97	2.71	-0.73	NA	NA
ENST00000496817	S100A6	183.15	-9.97	3.03	-3.29	9.91E-04	2.16E-01	45061.65	0.02	0.14	0.18	0.86	1.00
ENST00000369923	SARS	49.26	-9.21	2.06	-4.48	7.57E-06	1.96E-03	357.10	-0.06	0.44	-0.13	0.89	1.00
ENST00000514362	SEC31A	16.56	-22.47	3.03	-7.42	1.18E-13	1.43E-10	64.13	-0.57	0.50	-1.13	0.26	1.00
ENST00000223641	SEC61B	7.26	-6.45	2.31	-2.79	5.35E-03	8.63E-01	8918.72	-0.06	0.12	-0.52	0.60	1.00
ENST00000360051	SEPT2	17.30	-22.53	3.03	-7.44	1.02E-13	1.31E-10	517.66	-0.02	2.12	-0.01	0.99	1.00
ENST00000358171	SERPINH1	46.26	-23.86	3.03	-7.88	3.24E-15	1.21E-11	166.16	0.46	0.65	0.71	0.48	1.00
ENST00000522552	SKP1	6.74	-21.24	3.03	-7.00	2.47E-12	1.22E-09	98.14	0.24	0.33	0.74	0.46	1.00
ENST00000552981	SLC25A3	19.01	-7.84	2.42	-3.24	1.20E-03	2.58E-01	1132.46	-0.56	0.44	-1.27	0.20	1.00
ENST00000551374	SLC38A2	15.79	-6.40	1.91	-3.35	8.20E-04	1.84E-01	22614.25	-0.19	0.15	-1.28	0.20	1.00
ENST00000483847	SMARCC1	5.54	-20.96	3.03	-6.91	4.84E-12	1.88E-09	20.05	0.12	0.60	0.20	0.84	1.00
ENST00000360804	SPP1	19.89	-7.90	3.03	-2.61	9.06E-03	1.00E+00	959.24	0.16	0.10	1.65	0.10	0.87
ENST00000488179	SSR2	33.51	-8.70	3.03	-2.88	4.04E-03	6.95E-01	6123.01	0.02	0.44	0.05	0.96	1.00
ENST00000480567	SSR2	24.29	-4.77	1.69	-2.83	4.68E-03	7.76E-01	5442.19	-0.84	0.91	-0.92	0.36	1.00
ENST00000541731	STRAP	11.66	-21.99	3.03	-7.26	3.88E-13	3.17E-10	839.12	0.16	0.33	0.48	0.63	1.00
ENST00000367874	TADA1	19.15	-3.96	1.36	-2.92	3.56E-03	6.27E-01	3422.89	0.06	0.10	0.57	0.57	1.00
ENST00000377636	TBC1D4	3.26	-5.32	1.66	-3.20	1.39E-03	2.93E-01	0.32	-1.84	4.04	-0.45	0.65	NA
ENST00000400011	TJP1	6.18	-21.11	3.03	-6.96	3.37E-12	1.47E-09	5.91	-3.48	3.83	-0.91	0.36	1.00
ENST00000552370	TMBIM6	5.28	-6.00	2.20	-2.73	6.40E-03	9.81E-01	66.21	0.47	0.50	0.95	0.34	1.00
ENST00000464650	TMCO1	15.09	-6.92	2.20	-3.14	1.67E-03	3.47E-01	8879.77	-0.08	1.54	-0.05	0.96	1.00
ENST00000423613	TNC	21.67	-8.07	3.03	-2.67	7.67E-03	1.00E+00	1589.13	0.35	0.28	1.26	0.21	1.00
ENST00000405021	TOMM7	8.67	-6.11	2.01	-3.04	2.40E-03	4.62E-01	213.82	0.25	0.47	0.52	0.60	1.00
ENST00000300403	TPX2	18.37	-22.61	3.03	-7.47	8.31E-14	1.16E-10	79634.58	0.06	0.21	0.30	0.76	1.00
ENST00000494255	TRA2A	3.58	-4.79	1.84	-2.60	9.32E-03	1.00E+00	6.47	0.03	0.69	0.05	0.96	1.00
ENST00000541364	TUBA1C	98.99	-24.90	3.03	-8.23	1.94E-16	1.75E-12	882.06	-0.15	0.26	-0.57	0.57	1.00
ENST00000552448	TUBA1C	35.97	-23.52	3.03	-7.77	7.98E-15	2.22E-11	87.92	-0.43	0.69	-0.62	0.54	1.00
ENST00000425134	TXNIP	9.38	-6.82	2.29	-2.98	2.90E-03	5.45E-01	175.11	-0.59	0.47	-1.26	0.21	1.00
ENST00000246548	UBA2	10.17	-21.10	3.03	-6.96	3.34E-12	1.47E-09	15746.70	0.01	0.57	0.02	0.99	1.00
ENST00000339647	UBC	21.80	-2.92	1.08	-2.69	7.08E-03	1.00E+00	1133.45	-1.75	1.25	-1.40	0.16	0.97
ENST00000462569	UBE2G2	5.83	-5.54	1.98	-2.80	5.03E-03	8.20E-01	7.15	0.01	0.93	0.02	0.99	1.00
ENST00000523920	UQCRB	11.93	-6.58	2.07	-3.18	1.47E-03	3.07E-01	40.77	-0.70	0.50	-1.41	0.16	0.96
ENST00000369825	USMG5	19.74	-4.62	1.39	-3.32	9.06E-04	2.00E-01	616.85	-0.05	1.86	-0.03	0.98	1.00
ENST00000518563	VDAC3	9.00	-21.64	3.03	-7.14	9.22E-13	6.06E-10	111.25	-0.22	0.51	-0.43	0.67	1.00
ENST00000467957	YBX1	35.14	-23.48	3.03	-7.76	8.77E-15	2.27E-11	807.84	-0.91	0.57	-1.58	0.11	0.90

ENST00000299927	ZNF592	69.60	-4.88	1.20	-4.08	4.58E-05	1.16E-02	298.40	0.50	0.40	1.26	0.21	1.00

Table S2. Proteins with different expression in the SILAC approach upon NSUN5 restoration in LN229 cells. Related to Figure 5.

A. Upregulated upon restoration of NSUN5 (p-value ≤ 0.05)

Accession	Description
Q96P11	Probable 28S rRNA (cytosine-C(5))-methyltransferase OS=Homo sapiens GN=NSUN5 PE=1 SV=2
O14949	Cytochrome b-c1 complex subunit 8 OS=Homo sapiens GN=UQCRQ PE=1 SV=4
Q15785	Mitochondrial import receptor subunit TOM34 OS=Homo sapiens GN=TOMM34 PE=1 SV=2
Q9BV57	1,2-dihydroxy-3-keto-5-methylthiopentene dioxygenase OS=Homo sapiens GN=ADI1 PE=1 SV=1
P07942	Laminin subunit beta-1 OS=Homo sapiens GN=LAMB1 PE=1 SV=2
Q7Z5H4	Vomeronasal type-1 receptor 5 OS=Homo sapiens GN=VN1R5 PE=2 SV=2
Q15165	Serum paraoxonase/arylesterase 2 OS=Homo sapiens GN=PON2 PE=1 SV=3
Q9Y5K8	V-type proton ATPase subunit D OS=Homo sapiens GN=ATP6V1D PE=1 SV=1
P26358	DNA (cytosine-5)-methyltransferase 1 OS=Homo sapiens GN=DNMT1 PE=1 SV=2
P21399	Cytoplasmic aconitate hydratase OS=Homo sapiens GN=ACO1 PE=1 SV=3
Q6BCY4	NADH-cytochrome b5 reductase 2 OS=Homo sapiens GN=CYB5R2 PE=1 SV=1
Q8TBC4	NEDD8-activating enzyme E1 catalytic subunit OS=Homo sapiens GN=UBA3 PE=1 SV=2
O00625	Pirin OS=Homo sapiens GN=PIR PE=1 SV=1
P35249	Replication factor C subunit 4 OS=Homo sapiens GN=RFC4 PE=1 SV=2
Q9UHX1	Poly(U)-binding-splicing factor PUF60 OS=Homo sapiens GN=PUF60 PE=1 SV=1
P19404	NADH dehydrogenase [ubiquinone] flavoprotein 2, mitochondrial OS=Homo sapiens GN=NDUFV2 PE=1 SV=2
Q53GS9	U4/U6.U5 tri-snRNP-associated protein 2 OS=Homo sapiens GN=USP39 PE=1 SV=2
Q96DG6	Carboxymethylenebutenolidase homolog OS=Homo sapiens GN=CMBL PE=1 SV=1
P48147	Prolyl endopeptidase OS=Homo sapiens GN=PREP PE=1 SV=2
P13473	Lysosome-associated membrane glycoprotein 2 OS=Homo sapiens GN=LAMP2 PE=1 SV=2
O95562	Vesicle transport protein SFT2B OS=Homo sapiens GN=SFT2D2 PE=1 SV=1
Q16543	Hsp90 co-chaperone Cdc37 OS=Homo sapiens GN=CDC37 PE=1 SV=1
Q13144	Translation initiation factor eIF-2B subunit epsilon OS=Homo sapiens GN=EIF2B5 PE=1 SV=3
O75369	Filamin-B OS=Homo sapiens GN=FLNB PE=1 SV=2
Q8TC12	Retinol dehydrogenase 11 OS=Homo sapiens GN=RDH11 PE=1 SV=2
P82979	SAP domain-containing ribonucleoprotein OS=Homo sapiens GN=SARNP PE=1 SV=3
P19022	Cadherin-2 OS=Homo sapiens GN=CDH2 PE=1 SV=4
Q9H8Y8	Golgi reassembly-stacking protein 2 OS=Homo sapiens GN=GORASP2 PE=1 SV=3
P53007	Tricarboxylate transport protein, mitochondrial OS=Homo sapiens GN=SLC25A1 PE=1 SV=2
P16152	Carbonyl reductase [NADPH] 1 OS=Homo sapiens GN=CBR1 PE=1 SV=3
Q92544	Transmembrane 9 superfamily member 4 OS=Homo sapiens GN=TM9SF4 PE=1 SV=2
O60664	Perilipin-3 OS=Homo sapiens GN=PLIN3 PE=1 SV=3
P43246	DNA mismatch repair protein Msh2 OS=Homo sapiens GN=MSH2 PE=1 SV=1
O43493	Trans-Golgi network integral membrane protein 2 OS=Homo sapiens GN=TGOLN2 PE=1 SV=2
P09936	Ubiquitin carboxyl-terminal hydrolase isozyme L1 OS=Homo sapiens GN=UCHL1 PE=1 SV=2
O00469	Procollagen-lysine,2-oxoglutarate 5-dioxygenase 2 OS=Homo sapiens GN=PLOD2 PE=1

	SV=2
Q86Y56	Dynein assembly factor 5, axonemal OS=Homo sapiens GN=DNAAF5 PE=1 SV=4
Q32P28	Prolyl 3-hydroxylase 1 OS=Homo sapiens GN=P3H1 PE=1 SV=2
O43676	NADH dehydrogenase [ubiquinone] 1 beta subcomplex subunit 3 OS=Homo sapiens GN=NDUFB3 PE=1 SV=3
P54709	Sodium/potassium-transporting ATPase subunit beta-3 OS=Homo sapiens GN=ATP1B3 PE=1 SV=1
P61163	Alpha-centractin OS=Homo sapiens GN=ACTR1A PE=1 SV=1
P04264	Keratin, type II cytoskeletal 1 OS=Homo sapiens GN=KRT1 PE=1 SV=6
Q9Y3E5	Peptidyl-tRNA hydrolase 2, mitochondrial OS=Homo sapiens GN=PTRH2 PE=1 SV=1
Q9UH62	Armadillo repeat-containing X-linked protein 3 OS=Homo sapiens GN=ARMCX3 PE=1 SV=1
O43719	HIV Tat-specific factor 1 OS=Homo sapiens GN=HTATSF1 PE=1 SV=1
Q9UNH7	Sorting nexin-6 OS=Homo sapiens GN=SNX6 PE=1 SV=1
P42330	Aldo-keto reductase family 1 member C3 OS=Homo sapiens GN=AKR1C3 PE=1 SV=4
Q9Y520	Protein PRRC2C OS=Homo sapiens GN=PRRC2C PE=1 SV=4
O75152	Zinc finger CCCH domain-containing protein 11A OS=Homo sapiens GN=ZC3H11A PE=1 SV=3
Q92925	SWI/SNF-related matrix-associated actin-dependent regulator of chromatin subfamily D member 2 OS=Homo sapiens GN=SMARCD2 PE=1 SV=3
Q99614	Tetratricopeptide repeat protein 1 OS=Homo sapiens GN=TTC1 PE=1 SV=1
Q92597	Protein NDRG1 OS=Homo sapiens GN=NDRG1 PE=1 SV=1
Q96FZ7	Charged multivesicular body protein 6 OS=Homo sapiens GN=CHMP6 PE=1 SV=3
O95169	NADH dehydrogenase [ubiquinone] 1 beta subcomplex subunit 8, mitochondrial OS=Homo sapiens GN=NDUFB8 PE=1 SV=1
P53701	Cytochrome c-type heme lyase OS=Homo sapiens GN=HCCS PE=1 SV=1
O95817	BAG family molecular chaperone regulator 3 OS=Homo sapiens GN=BAG3 PE=1 SV=3
Q9UI12	V-type proton ATPase subunit H OS=Homo sapiens GN=ATP6V1H PE=1 SV=1
Q13895	Bystin OS=Homo sapiens GN=BYSL PE=1 SV=3
O60613	15 kDa selenoprotein OS=Homo sapiens GN=SEP15 PE=1 SV=3
Q99943	1-acyl-sn-glycerol-3-phosphate acyltransferase alpha OS=Homo sapiens GN=AGPAT1 PE=1 SV=2
Q9GZT8	NIF3-like protein 1 OS=Homo sapiens GN=NIF3L1 PE=1 SV=2
Q08209	Serine/threonine-protein phosphatase 2B catalytic subunit alpha isoform OS=Homo sapiens GN=PPP3CA PE=1 SV=1
P13995	Bifunctional methylenetetrahydrofolate dehydrogenase/cyclohydrolase, mitochondrial OS=Homo sapiens GN=MTHFD2 PE=1 SV=2
P11908	Ribose-phosphate pyrophosphokinase 2 OS=Homo sapiens GN=PRPS2 PE=1 SV=2
Q3YEC7	Rab-like protein 6 OS=Homo sapiens GN=RABL6 PE=1 SV=2
P30043	Flavin reductase (NADPH) OS=Homo sapiens GN=BLVRB PE=1 SV=3
Q9Y6W5	Wiskott-Aldrich syndrome protein family member 2 OS=Homo sapiens GN=WASF2 PE=1 SV=3
Q7Z417	Nuclear fragile X mental retardation-interacting protein 2 OS=Homo sapiens GN=NUFIP2 PE=1 SV=1
Q9NW13	RNA-binding protein 28 OS=Homo sapiens GN=RBM28 PE=1 SV=3
Q9H098	Protein FAM107B OS=Homo sapiens GN=FAM107B PE=1 SV=1
Q96IZ0	PRKC apoptosis WT1 regulator protein OS=Homo sapiens GN=PAWR PE=1 SV=1
Q12999	Tetraspanin-31 OS=Homo sapiens GN=TSPAN31 PE=2 SV=1

O15269	Serine palmitoyltransferase 1 OS=Homo sapiens GN=SPTLC1 PE=1 SV=1
P43355	Melanoma-associated antigen 1 OS=Homo sapiens GN=MAGEA1 PE=1 SV=1
P78362	SRSF protein kinase 2 OS=Homo sapiens GN=SRPK2 PE=1 SV=3
O15160	DNA-directed RNA polymerases I and III subunit RPAC1 OS=Homo sapiens GN=POLR1C PE=1 SV=1
Q96DI7	U5 small nuclear ribonucleoprotein 40 kDa protein OS=Homo sapiens GN=SNRNP40 PE=1 SV=1
Q9Y4I1	Unconventional myosin-Va OS=Homo sapiens GN=MYO5A PE=1 SV=2
Q9BVM2	Protein DPCD OS=Homo sapiens GN=DPCD PE=1 SV=2
O00186	Syntaxin-binding protein 3 OS=Homo sapiens GN=STXBP3 PE=1 SV=2
Q9Y605	MORF4 family-associated protein 1 OS=Homo sapiens GN=MRFAP1 PE=1 SV=1
Q9BVJ6	U3 small nucleolar RNA-associated protein 14 homolog A OS=Homo sapiens GN=UTP14A PE=1 SV=1
Q8N3F8	MICAL-like protein 1 OS=Homo sapiens GN=MICALL1 PE=1 SV=2
P56182	Ribosomal RNA processing protein 1 homolog A OS=Homo sapiens GN=RRP1 PE=1 SV=1
P23258	Tubulin gamma-1 chain OS=Homo sapiens GN=TUBG1 PE=1 SV=2
P07311	Acylphosphatase-1 OS=Homo sapiens GN=ACYP1 PE=1 SV=2
Q8TF72	Protein Shroom3 OS=Homo sapiens GN=SHROOM3 PE=1 SV=2
Q8N122	Regulatory-associated protein of mTOR OS=Homo sapiens GN=RPTOR PE=1 SV=1
P13716	Delta-aminolevulinic acid dehydratase OS=Homo sapiens GN=ALAD PE=1 SV=1
Q9H9G7	Protein argonaute-3 OS=Homo sapiens GN=AGO3 PE=1 SV=2
Q00653	Nuclear factor NF-kappa-B p100 subunit OS=Homo sapiens GN=NFKB2 PE=1 SV=4
P08559	Pyruvate dehydrogenase E1 component subunit alpha, somatic form, mitochondrial OS=Homo sapiens GN=PDHA1 PE=1 SV=3
Q9H910	Hematological and neurological expressed 1-like protein OS=Homo sapiens GN=HN1L PE=1 SV=1
Q9BUN8	Derlin-1 OS=Homo sapiens GN=DERL1 PE=1 SV=1

B. Downregulated upon restoration of NSUN5 (p-value ≤ 0.05)

Accession	Description
P36551	Oxygen-dependent coproporphyrinogen-III oxidase, mitochondrial OS=Homo sapiens GN=CPOX PE=1 SV=3
Q9UHV9	Prefoldin subunit 2 OS=Homo sapiens GN=PFDN2 PE=1 SV=1
Q96TA2	ATP-dependent zinc metalloprotease YME1L1 OS=Homo sapiens GN=YME1L1 PE=1 SV=2
O75044	SLIT-ROBO Rho GTPase-activating protein 2 OS=Homo sapiens GN=SRGAP2 PE=1 SV=2
P20962	Parathymosin OS=Homo sapiens GN=PTMS PE=1 SV=2
P20933	N(4)-(beta-N-acetylglucosaminyl)-L-asparaginase OS=Homo sapiens GN=AGA PE=1 SV=2
P53992	Protein transport protein Sec24C OS=Homo sapiens GN=SEC24C PE=1 SV=3
Q99798	Aconitate hydratase, mitochondrial OS=Homo sapiens GN=ACO2 PE=1 SV=2
Q9UJU6	Drebrin-like protein OS=Homo sapiens GN=DBNL PE=1 SV=1
Q9NXR7	BRCA1-A complex subunit BRE OS=Homo sapiens GN=BRE PE=1 SV=2
Q9Y376	Calcium-binding protein 39 OS=Homo sapiens GN=CAB39 PE=1 SV=1
Q8WYA6	Beta-catenin-like protein 1 OS=Homo sapiens GN=CTNNBL1 PE=1 SV=1

Q5T1J5	Putative coiled-coil-helix-coiled-coil-helix domain-containing protein CHCHD2P9, mitochondrial OS=Homo sapiens GN=CHCHD2P9 PE=5 SV=1
Q9Y5B9	FACT complex subunit SPT16 OS=Homo sapiens GN=SUPT16H PE=1 SV=1
Q96C90	Protein phosphatase 1 regulatory subunit 14B OS=Homo sapiens GN=PPP1R14B PE=1 SV=3
P49006	MARCKS-related protein OS=Homo sapiens GN=MARCKSL1 PE=1 SV=2
P20036	HLA class II histocompatibility antigen, DP alpha 1 chain OS=Homo sapiens GN=HLA-DPA1 PE=1 SV=1
Q00577	Transcriptional activator protein Pur-alpha OS=Homo sapiens GN=PURA PE=1 SV=2
Q13011	Delta(3,5)-Delta(2,4)-dienoyl-CoA isomerase, mitochondrial OS=Homo sapiens GN=ECH1 PE=1 SV=2
Q9Y6C9	Mitochondrial carrier homolog 2 OS=Homo sapiens GN=MTCH2 PE=1 SV=1
P24534	Elongation factor 1-beta OS=Homo sapiens GN=EEF1B2 PE=1 SV=3
P15559	NAD(P)H dehydrogenase [quinone] 1 OS=Homo sapiens GN=NQO1 PE=1 SV=1
Q8N766	ER membrane protein complex subunit 1 OS=Homo sapiens GN=EMC1 PE=1 SV=1
P26641	Elongation factor 1-gamma OS=Homo sapiens GN=EEF1G PE=1 SV=3
Q9Y266	Nuclear migration protein nudC OS=Homo sapiens GN=NUDC PE=1 SV=1
Q14847	LIM and SH3 domain protein 1 OS=Homo sapiens GN=LASP1 PE=1 SV=2
P21589	5'-nucleotidase OS=Homo sapiens GN=NT5E PE=1 SV=1
O14979	Heterogeneous nuclear ribonucleoprotein D-like OS=Homo sapiens GN=HNRNPDL PE=1 SV=3
A6NHL2	Tubulin alpha chain-like 3 OS=Homo sapiens GN=TUBAL3 PE=1 SV=2
Q13243	Serine/arginine-rich splicing factor 5 OS=Homo sapiens GN=SRSF5 PE=1 SV=1
Q9UGV2	Protein NDRG3 OS=Homo sapiens GN=NDRG3 PE=1 SV=2
Q99447	Ethanolamine-phosphate cytidylyltransferase OS=Homo sapiens GN=PCYT2 PE=1 SV=1
Q6P587	Acylpyruvase FAHD1, mitochondrial OS=Homo sapiens GN=FAHD1 PE=1 SV=2
Q9ULH0	Kinase D-interacting substrate of 220 kDa OS=Homo sapiens GN=KIDINS220 PE=1 SV=3

Table S3. List of transcripts from RNA-seq data with differential levels in empty vector vs NSUN5 transfected LN229 and A172 cells. Related to Figure 5.

A. Upregulated upon NSUN5 transfection in LN229 cells with oxidative stress

Ensembl ID	bMean	lfc	lfcSE	stat	pvalue	padj	Gene
ENST00000428206	88349.23	7.90	0.24	33.53	1.7E-246	1.1E-241	NSUN5
ENST00000471461	13102.11	11.36	0.40	28.65	1.6E-180	5.2E-176	NSUN5
ENST00000478977	21886.25	12.48	0.81	15.47	5.6E-54	2.9E-50	NSUN5
ENST00000393651	967.76	4.06	0.27	15.29	8.83E-53	4.4E-49	SRPK2
ENST00000564558	576.62	3.33	0.23	14.33	1.35E-46	5.16E-43	ZFP90
ENST00000356448	473.51	7.65	0.55	13.95	3.33E-44	1.23E-40	ARHGEF26
ENST00000265631	3197.50	5.66	0.41	13.83	1.61E-43	5.79E-40	SLC25A13
ENST00000367765	689.13	3.73	0.27	13.58	4.97E-42	1.74E-38	KIFAP3
ENST00000335757	730.02	7.69	0.57	13.51	1.46E-41	4.84E-38	SLC44A2
ENST00000282223	1399.43	4.27	0.34	12.49	8.07E-36	2.23E-32	SPOCK1
ENST00000432648	2729.74	3.80	0.36	10.65	1.67E-26	2.4E-23	AKIRIN1
ENST00000493099	266.49	8.64	0.89	9.74	2.04E-22	2.38E-19	C22orf13
ENST00000561623	4755.79	2.02	0.21	9.72	2.44E-22	2.8E-19	GTF3C1
ENST00000445140	3393.56	2.09	0.23	9.28	1.74E-20	1.71E-17	FXR1
ENST00000537534	224.12	11.25	1.22	9.23	2.76E-20	2.67E-17	UBXN1
ENST00000230901	1685.79	3.46	0.39	8.95	3.46E-19	3.05E-16	BRD8
ENST00000489353	560.01	2.71	0.30	8.91	4.94E-19	4.3E-16	EIF3I
ENST00000537126	609.39	3.28	0.37	8.87	7.55E-19	6.53E-16	MLF2
ENST00000477925	817.44	2.37	0.27	8.68	4.08E-18	3.27E-15	SRPK2
ENST00000268989	56.04	5.56	0.65	8.58	9.33E-18	7.29E-15	SGSM2
ENST00000361016	837.79	2.11	0.25	8.53	1.43E-17	1.1E-14	PCDHB16
ENST00000540530	293.56	11.64	1.40	8.33	8.01E-17	5.71E-14	NDUFS7
ENST00000485775	422.76	6.39	0.77	8.24	1.68E-16	1.14E-13	FAM40A
ENST00000252594	14561.25	12.40	1.52	8.16	3.31E-16	2.18E-13	NSUN5
ENST00000422437	388.66	2.58	0.32	7.98	1.5E-15	9.19E-13	XXbac-BPG300A18.12.1
ENST00000567957	206.41	4.01	0.51	7.89	2.97E-15	1.75E-12	ITFG1
ENST00000549773	34.50	4.64	0.60	7.72	1.14E-14	6.59E-12	METTL1
ENST00000313432	3969.40	28.48	3.81	7.48	7.18E-14	3.68E-11	ATP2A2
ENST00000532920	388.39	2.30	0.34	6.85	7.47E-12	2.97E-09	CYB5R2
ENST00000367010	73.10	7.20	1.07	6.74	1.56E-11	5.99E-09	HHAT
ENST00000220592	269.97	2.22	0.33	6.73	1.74E-11	6.65E-09	EIF2C2
ENST00000268616	1904.71	5.65	0.88	6.45	1.15E-10	3.99E-08	ZCCHC14
ENST00000540900	950.70	4.24	0.68	6.24	4.25E-10	1.36E-07	NSRP1
ENST00000413769	38.97	2.37	0.38	6.24	4.48E-10	1.43E-07	RBMXL1
ENST00000282050	16090.14	13.03	2.11	6.18	6.58E-10	2.06E-07	ATP5A1
ENST00000483517	707.68	3.89	0.67	5.81	6.16E-09	1.71E-06	EIF3I
ENST00000258530	1482.04	11.53	2.04	5.65	1.59E-08	4.11E-06	APPL2
ENST00000552285	2178.54	6.50	1.15	5.65	1.64E-08	4.21E-06	OS9
ENST00000457199	826.85	2.15	0.38	5.60	2.18E-08	5.51E-06	CHORDC1
ENST00000435975	1603.19	11.24	2.02	5.57	2.61E-08	6.5E-06	NCAPH

ENST00000471273	747.51	2.54	0.46	5.57	2.61E-08	6.5E-06	TBC1D23
ENST00000338427	37.69	2.66	0.49	5.48	4.35E-08	1.05E-05	NUDCD3
ENST00000503111	30.98	8.39	1.58	5.33	1E-07	2.28E-05	PIGG
ENST00000541543	20.15	7.77	1.47	5.30	1.18E-07	2.66E-05	CEP41
ENST00000552296	826.71	2.26	0.43	5.20	1.95E-07	4.23E-05	PCBP2
ENST00000426282	149.97	3.23	0.62	5.19	2.11E-07	4.57E-05	CTA-217C2.1.1
ENST00000230459	1474.67	4.14	0.81	5.09	3.57E-07	7.39E-05	COX7A2
ENST00000270649	415.42	9.69	1.95	4.96	6.98E-07	0.000134	ZNF614
ENST00000539480	23.15	7.97	1.61	4.94	7.75E-07	0.000148	NDUFS7
ENST00000531018	54.17	3.13	0.64	4.89	1E-06	0.000186	SYVN1
ENST00000371984	416.07	6.58	1.36	4.85	1.22E-06	0.00022	POMGNT1
ENST00000273963	242.04	3.81	0.80	4.78	1.76E-06	0.000309	KLHL8
ENST00000567873	234.87	8.87	1.89	4.70	2.64E-06	0.000447	STARD13
ENST00000361298	1895.38	5.16	1.11	4.67	3.04E-06	0.000507	NGFRAP1
ENST00000407215	204.69	4.91	1.05	4.66	3.2E-06	0.00053	SCRN2
ENST00000429452	317.96	6.03	1.30	4.64	3.41E-06	0.00056	XXbac-B461K10.4.1
ENST00000416160	605.62	8.37	1.81	4.62	3.79E-06	0.000618	ITSN2
ENST00000507846	172.92	7.70	1.67	4.62	3.87E-06	0.000628	PCDH18
ENST00000265806	175.73	2.70	0.59	4.58	4.61E-06	0.000734	R3HCC1
ENST00000509938	20.02	4.25	0.93	4.56	5.2E-06	0.00082	RP11-148B6.1.1
ENST00000527886	375.29	8.52	1.90	4.48	7.29E-06	0.001111	TOLLIP
ENST00000504986	673.59	5.75	1.28	4.48	7.58E-06	0.001147	TMEM33
ENST00000394186	2736.45	6.24	1.40	4.45	8.45E-06	0.001259	ATP5J2
ENST00000308979	52.55	6.26	1.41	4.45	8.58E-06	0.001274	TLR1
ENST00000483526	5.89	6.00	1.36	4.41	1.05E-05	0.001523	KIAA0895
ENST00000369755	1449.77	5.21	1.18	4.40	1.08E-05	0.001553	SLK
ENST00000411414	6.71	6.19	1.42	4.37	1.25E-05	0.001782	FAM160B1
ENST00000551434	45.98	3.34	0.77	4.35	1.37E-05	0.001934	CNOT2
ENST00000493335	831.47	4.35	1.00	4.33	1.48E-05	0.002064	SMTN
ENST00000469936	25.32	2.11	0.49	4.32	1.55E-05	0.002153	KHK
ENST00000521509	718.20	2.34	0.55	4.28	1.85E-05	0.002517	COPS5
ENST00000422947	27.25	2.59	0.61	4.27	1.97E-05	0.002662	GCDH
ENST00000394906	83.83	3.83	0.90	4.26	2.02E-05	0.002708	FLOT2
ENST00000443967	34.21	5.09	1.21	4.22	2.48E-05	0.003262	C5orf25
ENST00000404826	605.45	5.15	1.23	4.20	2.69E-05	0.003478	SDK1
ENST00000366862	2724.76	2.13	0.51	4.19	2.77E-05	0.003566	FBXO28
ENST00000513192	82.55	7.36	1.76	4.19	2.8E-05	0.003597	PIGG
ENST00000409212	569.09	9.78	2.34	4.18	2.9E-05	0.003695	SH3BP4
ENST00000504491	8.48	6.52	1.56	4.17	3.03E-05	0.003819	INTU
ENST00000374257	1873.79	4.24	1.02	4.14	3.43E-05	0.00423	PTBP3
ENST00000345136	87.60	3.07	0.74	4.13	3.67E-05	0.004501	PLEC
ENST00000335247	194.28	4.28	1.04	4.13	3.7E-05	0.004527	CYP27C1
ENST00000481616	26.28	2.47	0.60	4.12	3.85E-05	0.004697	PDS5B
ENST00000524776	54.41	5.78	1.41	4.11	3.94E-05	0.004793	DPH2
ENST00000367808	2525.51	3.76	0.92	4.09	4.25E-05	0.005123	BLZF1
ENST00000257986	382.62	6.46	1.58	4.09	4.33E-05	0.005214	EPB41L2

ENST00000450411	68.05	7.08	1.73	4.08	4.52E-05	0.005409	ILKAP
ENST00000219345	302.90	3.37	0.83	4.07	4.64E-05	0.005524	PLA2G15
ENST00000398148	8462.94	2.15	0.53	4.07	4.63E-05	0.005524	KHSRP
ENST00000375867	11.97	4.52	1.11	4.05	5.1E-05	0.005987	RP11-1E11.1.1
ENST00000376687	879.27	5.23	1.30	4.03	5.59E-05	0.006451	SUV39H1
ENST00000536530	356.76	5.15	1.28	4.03	5.59E-05	0.006451	ARMCX5
ENST00000391940	20.50	3.22	0.80	4.01	6.12E-05	0.006991	ERCC2
ENST00000381685	1488.79	3.45	0.86	3.99	6.56E-05	0.007395	NOL10
ENST00000294740	7.69	3.85	0.97	3.96	7.35E-05	0.008127	ZNF281
ENST00000162749	1615.92	3.62	0.91	3.96	7.59E-05	0.008348	TNFRSF1A
ENST00000512062	958.05	4.71	1.19	3.95	7.97E-05	0.008679	CYB5B
ENST00000540700	1478.49	5.33	1.35	3.94	8.16E-05	0.00883	UBC
ENST00000467601	46.56	6.11	1.55	3.94	8.29E-05	0.008957	PRRC2C

B. Downregulated upon NSUN5 transfection in LN229 cells with oxidative stress

Ensembl ID	bMean	lfc	lfcSE	stat	pvalue	padj	Gene
ENST00000416240	3194.02	-4.79	0.18	-26.94	7.25E-160	1.88E-155	SLC25A13
ENST00000538366	3804.42	-9.75	0.37	-26.27	4.11E-152	8.88E-148	KIFAP3
ENST00000541377	19979.83	-4.93	0.22	-22.45	1.27E-111	2.06E-107	CLASP1
ENST00000255977	3767.92	-12.49	0.83	-14.98	9.44E-51	4.22E-47	MKRN1
ENST00000414542	2028.46	-14.46	1.20	-12.04	2.24E-33	5.08E-30	BLCAP
ENST00000373963	754.25	-3.22	0.29	-11.12	9.60E-29	1.64E-25	DNPEP
ENST00000467807	409.92	-8.70	0.79	-11.03	2.79E-28	4.58E-25	ZNF542
ENST00000404574	1231.64	-4.98	0.46	-10.82	2.67E-27	4.13E-24	SMTN
ENST00000334298	207.63	-2.15	0.20	-10.79	3.87E-27	5.83E-24	LINC00221
ENST00000552650	127.82	-3.26	0.31	-10.67	1.46E-26	2.15E-23	TARBP2
ENST00000540793	1620.10	-2.51	0.24	-10.55	4.97E-26	6.86E-23	ATF7IP
ENST00000543413	355.08	-2.81	0.28	-10.06	8.67E-24	1.10E-20	SUCLA2
ENST00000340535	1106.99	-5.35	0.55	-9.76	1.70E-22	2.01E-19	MIA3
ENST00000279024	106.36	-6.75	0.69	-9.71	2.74E-22	3.11E-19	KIAA1755
ENST00000546260	1524.12	-8.94	0.92	-9.67	4.22E-22	4.75E-19	SOD2
ENST00000440023	1301.75	-4.47	0.47	-9.61	7.13E-22	7.83E-19	NSDHL
ENST00000430799	296.24	-11.68	1.25	-9.36	8.08E-21	8.32E-18	ARID1A
ENST00000504329	240.24	-11.38	1.22	-9.29	1.53E-20	1.51E-17	TMEM110-MUSTN1
ENST00000435120	349.33	-4.43	0.48	-9.18	4.23E-20	4.06E-17	MLF2
ENST00000484727	1146.28	-3.12	0.35	-8.82	1.20E-18	1.01E-15	ATP1B3
ENST00000367212	186.94	-9.16	1.08	-8.50	1.95E-17	1.50E-14	ZC3H11A
ENST00000537679	88.24	-9.93	1.24	-8.04	8.69E-16	5.47E-13	KPNB1
ENST00000483815	5782.34	-2.05	0.26	-8.00	1.29E-15	8.00E-13	C20orf24
ENST00000249760	3842.22	-4.62	0.59	-7.83	4.76E-15	2.77E-12	IVD
ENST00000525200	73.24	-9.67	1.24	-7.78	7.18E-15	4.16E-12	NADSYN1
ENST00000452723	518.44	-4.53	0.59	-7.63	2.43E-14	1.34E-11	CDK5RAP1
ENST00000336111	2264.35	-28.34	3.75	-7.55	4.33E-14	2.32E-11	OGFOD1
ENST00000568104	486.89	-12.40	1.64	-7.54	4.75E-14	2.51E-11	BBS2
ENST00000336216	2502.47	-4.26	0.58	-7.29	3.13E-13	1.49E-10	HMG20A

ENST00000420743	620.66	-26.56	3.77	-7.05	1.78E-12	7.63E-10	HNRNPC
ENST00000541801	36.76	-8.67	1.25	-6.95	3.62E-12	1.49E-09	ZNF211
ENST00000346315	55.03	-9.25	1.34	-6.89	5.70E-12	2.30E-09	BRCA1
ENST00000454876	652.65	-11.86	1.76	-6.74	1.55E-11	5.97E-09	SLC6A6
ENST00000463639	139.86	-10.60	1.59	-6.66	2.72E-11	1.00E-08	BRD2
ENST00000456556	213.65	-10.25	1.57	-6.53	6.47E-11	2.32E-08	ANKRD36C
ENST00000542654	233.36	-2.47	0.38	-6.43	1.24E-10	4.29E-08	SLC25A13
ENST00000345097	504.81	-10.01	1.56	-6.41	1.43E-10	4.88E-08	FOXN3
ENST00000404233	3307.82	-12.72	2.04	-6.23	4.65E-10	1.48E-07	HYOU1
ENST00000414273	35990.69	-11.99	1.94	-6.19	6.12E-10	1.93E-07	RP5-857K21.6.1
ENST00000380051	61.61	-4.59	0.75	-6.15	7.91E-10	2.44E-07	RPP40
ENST00000462965	336.51	-2.64	0.44	-6.00	1.97E-09	5.82E-07	ARPC5
ENST00000352861	609.89	-7.26	1.25	-5.80	6.45E-09	1.78E-06	SEC61G
ENST00000511217	160.16	-2.87	0.50	-5.79	6.99E-09	1.92E-06	SH3RF2
ENST00000374586	2532.16	-10.33	1.80	-5.76	8.57E-09	2.33E-06	C9orf5
ENST00000412207	54.60	-3.35	0.58	-5.74	9.74E-09	2.62E-06	SMARCE1P5
ENST00000543466	3983.02	-8.87	1.57	-5.65	1.65E-08	4.23E-06	G3BP1
ENST00000313005	2661.09	-9.82	1.76	-5.57	2.48E-08	6.20E-06	BACE1
ENST00000369795	954.74	-6.72	1.22	-5.52	3.47E-08	8.57E-06	FAM40A
ENST00000373302	173.99	-5.78	1.06	-5.45	5.08E-08	1.21E-05	STXBP1
ENST00000553674	166.63	-7.40	1.37	-5.39	7.17E-08	1.68E-05	UBR7
ENST00000407142	1650.89	-8.98	1.68	-5.36	8.22E-08	1.90E-05	PPM1F
ENST00000537043	268.44	-6.76	1.28	-5.28	1.32E-07	2.95E-05	VCL
ENST00000533064	629.10	-10.32	1.96	-5.26	1.47E-07	3.26E-05	TRIM3
ENST00000415149	1515.96	-3.48	0.67	-5.22	1.75E-07	3.85E-05	BBX
ENST00000554908	4209.08	-2.43	0.47	-5.16	2.52E-07	5.37E-05	GMFB
ENST00000252455	6243.86	-6.96	1.38	-5.03	4.89E-07	9.90E-05	PRKCSH
ENST00000403834	119.50	-3.31	0.66	-5.03	4.91E-07	9.92E-05	MED23
ENST00000480674	18.52	-2.72	0.54	-4.99	5.94E-07	0.000117	PRUNE2
ENST00000332396	5130.31	-5.48	1.10	-4.99	6.19E-07	0.000121	MRPL12
ENST00000355739	958.44	-2.60	0.53	-4.91	8.97E-07	0.000168	ERCC5
ENST00000544001	1561.52	-3.78	0.77	-4.90	9.62E-07	0.000179	ITFG1
ENST00000307602	319.20	-8.61	1.76	-4.89	1.02E-06	0.000189	HOOK3
ENST00000545045	26.45	-5.73	1.17	-4.88	1.08E-06	0.000198	ENO2
ENST00000367178	1896.37	-5.32	1.10	-4.84	1.30E-06	0.000234	SCAF8
ENST00000438478	269.37	-9.10	1.89	-4.83	1.39E-06	0.000248	RP11-3P17.3.1
ENST00000297565	43.15	-2.61	0.56	-4.64	3.40E-06	0.000559	OSR2
ENST00000375217	18088.92	-2.23	0.48	-4.61	4.09E-06	0.000656	UBR4
ENST00000280551	1075.70	-2.72	0.59	-4.58	4.60E-06	0.000734	SEC24D
ENST00000423517	197.08	-8.65	1.95	-4.44	9.17E-06	0.001352	PCLO
ENST00000428216	3939.55	-3.07	0.70	-4.39	1.14E-05	0.001632	MAVS
ENST00000568816	4206.65	-5.62	1.29	-4.37	1.23E-05	0.001756	PLDN
ENST00000359834	77.22	-9.74	2.25	-4.33	1.47E-05	0.002055	YES1
ENST00000465189	11.69	-7.02	1.63	-4.31	1.62E-05	0.002244	NSMCE4A
ENST00000359742	41.75	-4.48	1.04	-4.30	1.70E-05	0.00234	GRIP1
ENST00000357681	367.11	-7.96	1.86	-4.28	1.86E-05	0.002526	ATP2B4

ENST00000409906	999.19	-4.83	1.13	-4.27	1.97E-05	0.002662	TCEB2
ENST00000526666	14.93	-3.38	0.80	-4.22	2.44E-05	0.003216	PDE4B
ENST00000437245	9.57	-4.21	1.00	-4.22	2.48E-05	0.003266	CEP350
ENST00000393524	238.96	-3.80	0.90	-4.21	2.58E-05	0.003367	PHLPP2
ENST00000396822	937.92	-5.43	1.29	-4.20	2.69E-05	0.003478	SNAI2
ENST00000531480	131.67	-7.33	1.75	-4.19	2.80E-05	0.003597	CPSF1
ENST00000399408	79.14	-7.32	1.75	-4.19	2.81E-05	0.003603	ABCC1
ENST00000312431	1539.12	-5.94	1.42	-4.18	2.91E-05	0.003702	DST
ENST00000298048	1042.40	-2.27	0.55	-4.15	3.40E-05	0.00421	MELK
ENST00000481918	81.64	-3.02	0.73	-4.14	3.47E-05	0.004271	ANGEL2
ENST00000317538	101.84	-7.27	1.77	-4.11	3.89E-05	0.004738	ZMYM6
ENST00000428708	122.44	-3.44	0.84	-4.10	4.10E-05	0.004972	BAIAP2
ENST00000533799	5.04	-5.80	1.42	-4.08	4.42E-05	0.005306	DPP3
ENST00000494576	734.83	-12.03	2.96	-4.07	4.74E-05	0.005623	UBAC2
ENST00000530095	18.35	-3.20	0.79	-4.06	5.00E-05	0.005893	THUMPD2
ENST00000553208	11.61	-2.61	0.64	-4.05	5.12E-05	0.00601	OS9
ENST00000535458	116.26	-2.03	0.50	-4.04	5.40E-05	0.006282	KPNB1
ENST00000324379	876.65	-2.99	0.75	-4.01	6.04E-05	0.006917	PPIE
ENST00000356577	38.95	-2.03	0.51	-4.01	6.18E-05	0.007043	SON
ENST00000465505	39.24	-2.04	0.51	-4.00	6.35E-05	0.007197	SNX4
ENST00000539768	242.97	-5.87	1.47	-3.98	6.81E-05	0.007635	SLC38A9
ENST00000475896	28.73	-5.42	1.36	-3.98	6.84E-05	0.007652	ABI3BP
ENST00000409855	4.72	-5.71	1.43	-3.98	6.92E-05	0.007731	SCN7A
ENST00000503611	72.89	-6.79	1.71	-3.98	6.95E-05	0.007747	RP11-597D13.9.1
ENST00000530705	15503.76	-6.38	1.60	-3.98	6.96E-05	0.007752	TPT1
ENST00000490910	4.82	-5.74	1.44	-3.98	6.99E-05	0.007779	CRNKL1
ENST00000379091	80.49	-2.45	0.62	-3.95	7.66E-05	0.00841	ZBTB10
ENST00000395923	27.90	-8.27	2.10	-3.94	8.17E-05	0.008835	NCALD

C. Upregulated upon NSUN5 transfection in A172 cells without oxidative stress

Ensembl ID	bMean	lfc	lfcSE	stat	pvalue	padj	Gene
ENST00000428206	141911.11	8.20	0.11	73.05	0	0	NSUN5
ENST00000471461	26813.33	8.93	0.14	61.62	0	0	NSUN5
ENST00000478977	34355.82	12.02	0.31	38.62	0	0	NSUN5
ENST00000479532	1167.94	4.83	0.17	28.74	1.1E-181	1.1E-177	SF3B1
ENST00000554228	1116.26	5.17	0.19	27.79	6.3E-170	5.4E-166	KLC1
ENST00000461347	30170.93	2.30	0.08	27.13	4E-162	3.2E-158	NCL
ENST00000510146	1458.58	3.80	0.19	19.69	2.72E-86	9.01E-83	MLF1IP
ENST00000557450	796.54	4.63	0.24	19.57	2.67E-85	8.21E-82	KLC1
ENST00000497424	822.09	4.59	0.24	19.12	1.66E-81	4.86E-78	PANK2
ENST00000454685	315.90	4.43	0.23	18.92	7.66E-80	2.2E-76	MICALL1
ENST00000370323	891.39	2.10	0.12	17.11	1.17E-65	2.55E-62	MAGEA10
ENST00000373394	1329.84	3.43	0.25	13.59	4.45E-42	5.58E-39	ABCB7
ENST00000326232	287.29	5.61	0.41	13.54	9.4E-42	1.17E-38	SLC37A3
ENST00000378203	670.82	2.29	0.17	13.17	1.31E-39	1.5E-36	RPP38

ENST00000343115	4222.40	7.24	0.56	12.98	1.62E-38	1.76E-35	RDX
ENST00000417037	1725.61	9.77	0.75	12.95	2.36E-38	2.54E-35	MYD88
ENST00000561457	232.59	7.00	0.55	12.76	2.76E-37	2.83E-34	C9orf69
ENST00000409988	3316.98	3.76	0.33	11.52	1.04E-30	7.7E-28	SPATS2L
ENST00000367996	1723.91	2.52	0.22	11.32	1.02E-29	7.24E-27	ADAMTS4
ENST00000461992	153.49	5.53	0.51	10.79	4.03E-27	2.53E-24	C9orf86
ENST00000554280	298.20	3.87	0.39	9.99	1.62E-23	7.79E-21	KLC1
ENST00000556925	385.59	11.92	1.20	9.96	2.21E-23	1.05E-20	NEMF
ENST00000395824	270.60	11.41	1.22	9.34	9.41E-21	3.8E-18	ZNF287
ENST00000398803	2820.43	2.11	0.23	9.16	4.99E-20	1.91E-17	HSDL2
ENST00000296591	4188.57	2.08	0.23	9.00	2.32E-19	8.45E-17	EDIL3
ENST00000271348	1492.87	2.44	0.27	8.98	2.59E-19	9.41E-17	GJA5
ENST00000418388	136.91	7.98	0.90	8.87	7.63E-19	2.62E-16	C9orf69
ENST00000373297	1434.44	13.82	1.63	8.49	2.13E-17	6.55E-15	ZMYM4
ENST00000319432	488.39	12.26	1.46	8.39	4.94E-17	1.46E-14	CCDC93
ENST00000473251	195.74	10.95	1.31	8.37	5.54E-17	1.62E-14	RAD54L
ENST00000313833	401.93	4.72	0.57	8.24	1.78E-16	4.98E-14	FBXO34
ENST00000339526	3224.95	2.11	0.26	8.18	2.94E-16	8.08E-14	GLUL
ENST00000482609	229.25	3.76	0.46	8.16	3.35E-16	9.15E-14	RP11-129B22.1.1
ENST00000497024	90.40	3.74	0.46	8.15	3.78E-16	1.02E-13	ELMO1
ENST00000243326	243.84	4.86	0.60	8.09	5.97E-16	1.58E-13	RIF1
ENST00000313400	43.90	3.33	0.42	8.01	1.15E-15	2.92E-13	ASTN2
ENST00000484302	798.14	3.45	0.44	7.93	2.25E-15	5.58E-13	HOXB6
ENST00000542151	2439.59	3.51	0.47	7.40	1.33E-13	2.68E-11	KDM1A
ENST00000268349	184.14	5.58	0.76	7.33	2.3E-13	4.49E-11	FTO
ENST00000535624	65.66	3.90	0.53	7.31	2.7E-13	5.23E-11	MGST1
ENST00000521195	1788.30	26.48	3.74	7.09	1.34E-12	2.35E-10	FZD6
ENST00000355292	80.63	5.62	0.81	6.94	3.87E-12	6.49E-10	NR3C2
ENST00000293851	546.96	2.07	0.30	6.88	6.04E-12	9.86E-10	PRSS33
ENST00000372638	1057.39	2.02	0.30	6.82	8.98E-12	1.42E-09	CITED4
ENST00000541029	250.13	3.07	0.45	6.76	1.39E-11	2.16E-09	ATN1
ENST00000394224	590.38	25.72	3.81	6.75	1.44E-11	2.24E-09	SIPA1
ENST00000470670	2449.81	3.46	0.52	6.68	2.34E-11	3.54E-09	ARF1
ENST00000437079	143.91	5.02	0.75	6.67	2.52E-11	3.78E-09	VPS8
ENST00000437844	100.97	9.99	1.51	6.60	4.21E-11	6.08E-09	ZEB1
ENST00000392504	78.06	6.16	0.95	6.50	7.87E-11	1.09E-08	DCBLD1
ENST00000379858	175.55	10.79	1.66	6.49	8.59E-11	1.18E-08	TOPORS
ENST00000563462	29.21	5.73	0.89	6.40	1.51E-10	2E-08	KATNB1
ENST00000372155	145.39	4.12	0.64	6.39	1.66E-10	2.17E-08	DDX31
ENST00000295119	194.15	2.38	0.37	6.37	1.94E-10	2.51E-08	NUP35
ENST00000334895	221.97	2.67	0.43	6.27	3.63E-10	4.49E-08	TPM1
ENST00000490982	27.73	8.13	1.31	6.20	5.53E-10	6.62E-08	TRPM2
ENST00000360505	92.86	9.87	1.59	6.19	5.99E-10	7.13E-08	GSTCD
ENST00000472094	65.70	9.37	1.53	6.14	8.19E-10	9.49E-08	NRG4
ENST00000314607	1168.17	2.95	0.48	6.14	8.36E-10	9.64E-08	ZMYM4
ENST00000339647	1391.00	3.03	0.50	6.11	9.71E-10	1.11E-07	UBC

ENST00000448361	20.04	7.66	1.29	5.96	2.55E-09	2.72E-07	COMMD3
ENST00000506054	86.95	2.58	0.44	5.88	4.09E-09	4.21E-07	ARHGAP10
ENST00000520266	15.85	7.32	1.25	5.87	4.3E-09	4.41E-07	MOK
ENST00000388918	57.69	3.57	0.61	5.81	6.08E-09	6.09E-07	TYRP1
ENST00000291577	114.00	5.10	0.88	5.78	7.41E-09	7.22E-07	C21orf33
ENST00000520687	129930.43	2.24	0.39	5.77	7.98E-09	7.72E-07	SPARC
ENST00000222339	25.98	8.03	1.43	5.60	2.12E-08	1.9E-06	ZNF574
ENST00000366516	140.51	4.03	0.73	5.54	3.05E-08	2.67E-06	SMYD3
ENST00000420282	13.42	7.08	1.29	5.51	3.64E-08	3.14E-06	PPP1CB
ENST00000421904	20.85	7.71	1.42	5.44	5.4E-08	4.49E-06	DNAJC27-AS1
ENST00000371160	397.69	4.66	0.88	5.30	1.16E-07	9.04E-06	STAG2
ENST00000562695	76.37	4.12	0.78	5.30	1.16E-07	9.04E-06	C16orf52
ENST00000389087	3090.57	8.40	1.59	5.30	1.17E-07	9.08E-06	FBN1
ENST00000475394	45.27	2.72	0.52	5.27	1.36E-07	1.03E-05	DLG1
ENST00000407142	1413.66	8.36	1.59	5.25	1.54E-07	1.16E-05	PPM1F
ENST00000361922	277.50	2.08	0.40	5.23	1.68E-07	1.24E-05	MAG
ENST00000537536	9.80	6.63	1.28	5.19	2.13E-07	1.55E-05	TBC1D25
ENST00000523938	10.10	6.67	1.30	5.12	3.12E-07	2.19E-05	YWHAZ
ENST00000546382	17.66	3.14	0.62	5.03	4.89E-07	3.3E-05	CTD-2314B22.3.1
ENST00000256062	840.42	12.09	2.40	5.03	4.99E-07	3.36E-05	TMTC1
ENST00000372950	585.56	5.58	1.11	5.02	5.27E-07	3.52E-05	DNAJC9
ENST00000449062	38.43	4.68	0.94	4.98	6.22E-07	4.09E-05	C20orf43
ENST00000372572	549.60	5.42	1.09	4.98	6.46E-07	4.23E-05	FOXJ3
ENST00000411996	1573.35	4.38	0.88	4.97	6.77E-07	4.41E-05	DNM1L
ENST00000359867	271.68	8.97	1.82	4.93	8.08E-07	5.16E-05	CSNK1E
ENST00000457956	29.96	2.37	0.49	4.87	1.11E-06	6.88E-05	BTG3
ENST00000398484	3524.69	11.09	2.29	4.85	1.21E-06	7.4E-05	FGFRL1
ENST00000498586	14.41	4.68	0.97	4.85	1.25E-06	7.65E-05	NCOA4
ENST00000366837	180.99	9.87	2.04	4.84	1.32E-06	7.99E-05	EPHX1
ENST00000453626	335.06	2.42	0.50	4.84	1.33E-06	8.02E-05	DONSON
ENST00000454092	35.31	2.11	0.44	4.77	1.84E-06	0.000107	MIR4461
ENST00000559017	10.32	6.70	1.41	4.75	2.05E-06	0.000119	DCAF11
ENST00000334705	656.25	2.40	0.51	4.70	2.58E-06	0.000145	FAM91A1
ENST00000488497	1275.37	2.14	0.46	4.66	3.17E-06	0.000173	U2SURP
ENST00000517593	285.23	7.04	1.52	4.63	3.68E-06	0.000197	TTC35
ENST00000445856	29.78	3.44	0.75	4.60	4.29E-06	0.000224	EPHX1
ENST00000395411	995.91	5.83	1.27	4.58	4.64E-06	0.000241	CSNK1G3
ENST00000223321	812.71	3.46	0.76	4.58	4.72E-06	0.000244	PSMA2
ENST00000505013	6.65	6.06	1.33	4.56	5.15E-06	0.000263	ACPL2
ENST00000490885	338.96	7.42	1.63	4.54	5.62E-06	0.000284	PTGS2
ENST00000356259	28.69	4.45	0.98	4.52	6.24E-06	0.00031	GIT2
ENST00000372165	222.59	2.12	0.47	4.52	6.3E-06	0.000313	RSPH9
ENST00000456040	136.60	7.24	1.62	4.48	7.61E-06	0.000368	R3HDM1
ENST00000553932	36.71	7.56	1.70	4.46	8.16E-06	0.00039	RP11-566K8.2.1
ENST00000376834	963.09	5.08	1.14	4.45	8.63E-06	0.000409	C9orf41
ENST00000355790	215.76	6.90	1.55	4.45	8.71E-06	0.000412	LRRC20

ENST00000486300	21.13	3.77	0.85	4.44	9.09E-06	0.000428	AFAP1L2
ENST00000255380	79.13	8.67	1.96	4.42	9.7E-06	0.000451	CHRM3
ENST00000394419	2188.98	3.27	0.74	4.38	1.16E-05	0.00053	ACTN1
ENST00000354914	1334.61	2.28	0.52	4.38	1.21E-05	0.000548	SCARA5
ENST00000432399	131.69	10.37	2.38	4.37	1.26E-05	0.000566	MAST4
ENST00000508096	11.39	2.79	0.64	4.34	1.4E-05	0.00062	RP11-298J20.4.1
ENST00000505615	489.14	4.40	1.01	4.34	1.46E-05	0.000642	VCAN
ENST00000461287	8.71	6.46	1.49	4.33	1.51E-05	0.000664	LY6G6E
ENST00000470093	401.76	5.57	1.31	4.26	2.01E-05	0.000854	FBXW4
ENST00000526397	10.20	4.16	0.99	4.23	2.39E-05	0.000994	SERPINH1
ENST00000294737	57.78	9.19	2.17	4.22	2.4E-05	0.000998	DENND1B
ENST00000473565	27.49	5.22	1.24	4.20	2.62E-05	0.001078	CLK1
ENST00000276816	15.29	2.61	0.62	4.19	2.73E-05	0.001116	ZNF16
ENST00000540218	387.12	2.95	0.71	4.16	3.14E-05	0.001256	TYRO3
ENST00000262953	224.95	2.89	0.70	4.14	3.46E-05	0.001364	TLE2
ENST00000478047	228.53	4.36	1.05	4.14	3.46E-05	0.001364	CWF19L1
ENST00000543430	15.98	7.33	1.78	4.13	3.68E-05	0.001437	P2RX4
ENST00000396594	1815.14	4.64	1.13	4.13	3.71E-05	0.001445	GNE
ENST00000464060	23.90	6.02	1.46	4.12	3.74E-05	0.001456	MAGI1
ENST00000463227	15.01	3.05	0.74	4.11	4.03E-05	0.001552	DEDD
ENST00000320394	27.62	8.12	1.99	4.08	4.46E-05	0.00169	IQCK
ENST00000381352	580.64	2.01	0.49	4.08	4.5E-05	0.001699	RASSF8
ENST00000523313	18.68	3.23	0.79	4.06	4.84E-05	0.001809	RP11-697M17.1.1
ENST00000430935	10.79	3.81	0.95	4.03	5.68E-05	0.002065	TTC26
ENST00000343267	2252.07	2.35	0.59	4.01	6.15E-05	0.002207	APOD
ENST00000307659	2140.13	8.46	2.12	4.00	6.41E-05	0.002284	KIAA0232
ENST00000230658	82.40	2.25	0.56	4.00	6.44E-05	0.002292	ISL1
ENST00000356058	63.39	6.12	1.54	3.98	6.86E-05	0.002417	ABCB8
ENST00000404910	417.27	2.95	0.74	3.97	7.3E-05	0.002537	RAMP1
ENST00000462190	495.98	4.84	1.22	3.96	7.39E-05	0.002563	SPATS2L
ENST00000553674	392.74	2.56	0.65	3.96	7.6E-05	0.002625	UBR7
ENST00000518721	4710.40	2.01	0.51	3.95	7.81E-05	0.002687	ASAP1
ENST00000409331	8.10	3.81	0.97	3.93	8.37E-05	0.002849	ELMOD3
ENST00000272433	1805.60	3.73	0.95	3.93	8.5E-05	0.002883	SFXN5
ENST00000517097	4.82	5.60	1.43	3.91	9.42E-05	0.00314	SCARNA3
ENST00000485565	7.56	6.25	1.61	3.89	0.000101	0.00333	SPIN1
ENST00000540331	269.16	6.35	1.64	3.88	0.000105	0.003451	ZNF701
ENST00000397090	15.90	2.97	0.77	3.87	0.00011	0.00359	FGFR1
ENST00000347630	1782.33	5.14	1.33	3.86	0.000114	0.003679	SPOP
ENST00000329143	114.62	2.16	0.56	3.85	0.000117	0.003775	C11orf82
ENST00000400202	132.35	6.32	1.64	3.85	0.000117	0.003777	NRIP1
ENST00000396210	62.70	2.23	0.58	3.84	0.000123	0.003923	MGST1
ENST00000509345	10.42	3.40	0.89	3.83	0.000128	0.004069	MAGEA5
ENST00000361828	1315.33	2.79	0.73	3.82	0.000133	0.004194	OPA1
ENST00000404574	1131.00	4.39	1.16	3.80	0.000145	0.00453	SMTN
ENST00000492211	59.71	5.75	1.52	3.78	0.000159	0.00489	ARHGAP19

ENST00000356839	2644.07	2.68	0.71	3.77	0.000164	0.004998	ACADVL
ENST00000556393	13.16	7.05	1.88	3.76	0.000172	0.005201	L2HGDH
ENST00000302851	1024.04	3.76	1.00	3.74	0.000181	0.005444	ZNF561
ENST00000295598	70840.68	2.82	0.75	3.74	0.000182	0.005468	ATP1A1
ENST00000524558	7303.11	2.63	0.70	3.73	0.000191	0.005685	SERPINH1
ENST00000441447	29.02	2.91	0.78	3.73	0.000192	0.005718	ZMYM4
ENST00000488909	33.90	2.71	0.73	3.73	0.000194	0.005763	ODF2
ENST00000453304	1431.87	13.82	3.72	3.72	0.0002	0.005899	UNC5C
ENST00000421660	64.88	2.74	0.74	3.72	0.000201	0.00591	YEATS2
ENST00000441210	78.57	3.16	0.85	3.72	0.000202	0.005945	TINAGL1
ENST00000337959	206.08	3.65	0.98	3.72	0.000203	0.005949	PACSIN2
ENST00000409963	1287.60	13.66	3.70	3.70	0.000219	0.006313	MRPL38
ENST00000349999	10.29	4.79	1.31	3.66	0.000253	0.007109	HFE
ENST00000263713	458.61	3.56	0.97	3.65	0.000259	0.007243	EPB41L5
ENST00000370314	60.13	8.28	2.27	3.65	0.00026	0.007259	GABRA3
ENST00000370256	307.79	4.64	1.27	3.64	0.000272	0.007523	FNBP1L
ENST00000512871	28.02	2.85	0.78	3.63	0.000285	0.007833	PRRC1
ENST00000425762	1066.05	3.69	1.02	3.63	0.000287	0.007876	PSMB5
ENST00000467131	10.19	2.83	0.78	3.62	0.000299	0.008148	LEPREL1
ENST00000261892	31.82	4.83	1.34	3.61	0.000305	0.008256	SLC24A1
ENST00000420764	26.32	3.23	0.90	3.61	0.000312	0.008424	SLC26A6
ENST00000557666	93.48	4.50	1.25	3.60	0.000321	0.008617	BAG5
ENST00000523303	14.87	3.95	1.10	3.60	0.000322	0.00863	INTS9
ENST00000491976	51.40	2.94	0.82	3.58	0.000344	0.009111	SERINC2
ENST00000509156	578.44	9.05	2.53	3.58	0.000346	0.009154	ZNF131
ENST00000535305	818.45	13.01	3.64	3.57	0.000354	0.009303	TMEM87A
ENST00000546155	38.57	2.45	0.69	3.57	0.000356	0.009343	LTBP4
ENST00000495414	30.67	3.47	0.97	3.57	0.000357	0.009354	C3orf58
ENST00000335144	812.79	13.00	3.65	3.57	0.000364	0.009489	SPIN4
ENST00000548968	15.12	2.45	0.69	3.56	0.000372	0.009649	LRIG3

D. Downregulated upon NSUN5 transfection in A172 cells without oxidative stress

Ensembl ID	bMean	lfc	lfcSE	stat	pvalue	padj	Gene
ENST00000336066	1752.90	-5.81	0.20	-28.77	4.6E-182	4.5E-178	PANK2
ENST00000409601	2123.81	-5.38	0.22	-24.48	2.2E-132	1.2E-128	MTHFD2
ENST00000453712	905.21	-4.54	0.23	-19.62	9.59E-86	3.02E-82	RAP1GDS1
ENST00000316562	719.38	-3.19	0.18	-18.04	9.87E-73	2.55E-69	PANK2
ENST00000345133	552.44	-5.05	0.29	-17.44	3.84E-68	9.02E-65	TGIF1
ENST00000256103	4299.79	-2.73	0.16	-17.25	1.19E-66	2.65E-63	PMP2
ENST00000544910	915.71	-9.40	0.56	-16.77	3.82E-63	7.96E-60	CCDC132
ENST00000263849	1801.19	-2.10	0.13	-16.41	1.61E-60	3.19E-57	FAM164A
ENST00000193403	14100.18	-2.34	0.15	-15.60	6.78E-55	1.2E-51	ACTN1
ENST00000521873	184.84	-4.71	0.33	-14.19	1.06E-45	1.47E-42	FAM164A
ENST00000312431	2341.74	-7.16	0.51	-13.91	5.43E-44	7.01E-41	DST
ENST00000321521	322.91	-5.27	0.41	-12.91	3.94E-38	4.18E-35	PPIP5K2

ENST00000374955	315.12	-2.50	0.20	-12.80	1.63E-37	1.7E-34	PAGE5
ENST00000512621	923.75	-2.55	0.20	-12.71	4.93E-37	4.86E-34	ADH5
ENST00000322635	448.50	-9.54	0.77	-12.44	1.5E-35	1.37E-32	CAMK2G
ENST00000368124	2198.63	-2.18	0.18	-12.27	1.29E-34	1.11E-31	CADM3
ENST00000493099	319.60	-8.72	0.73	-11.93	8.73E-33	7.28E-30	C22orf13
ENST00000257749	126.03	-5.77	0.49	-11.83	2.72E-32	2.16E-29	BACH2
ENST00000560241	86.15	-3.81	0.33	-11.61	3.61E-31	2.76E-28	IL16
ENST00000264867	494.93	-4.40	0.38	-11.53	9.17E-31	6.89E-28	PPARGC1A
ENST00000298049	149.41	-3.61	0.32	-11.17	5.65E-29	3.82E-26	EEF1A2
ENST00000463787	939.21	-2.43	0.22	-10.88	1.38E-27	8.93E-25	MAGED2
ENST00000264071	551.31	-2.16	0.21	-10.41	2.19E-25	1.25E-22	TUBB4A
ENST00000423381	444.14	-12.38	1.21	-10.27	1E-24	5.39E-22	CAMK2G
ENST00000480329	144.34	-5.35	0.53	-10.01	1.44E-23	7E-21	SLC35C2
ENST00000536483	732.53	-3.98	0.40	-10.00	1.53E-23	7.4E-21	PRKDC
ENST00000402643	78.57	-2.89	0.29	-9.89	4.42E-23	2.07E-20	GAPDHP72
ENST00000360760	287.76	-3.11	0.31	-9.89	4.72E-23	2.2E-20	SPATS2L
ENST00000303115	2302.95	-2.11	0.22	-9.46	2.96E-21	1.24E-18	IL7R
ENST00000409707	245.53	-11.53	1.26	-9.15	5.47E-20	2.07E-17	STAMBP
ENST00000313234	1144.80	-2.82	0.32	-8.94	3.93E-19	1.4E-16	KRT80
ENST00000333046	138.34	-8.25	0.94	-8.81	1.25E-18	4.21E-16	TPRN
ENST00000431180	245.82	-9.67	1.11	-8.75	2.16E-18	7.11E-16	SETD2
ENST00000477128	1108.31	-4.85	0.56	-8.71	3.16E-18	1.03E-15	UTP6
ENST00000384756	61.36	-6.33	0.73	-8.62	6.96E-18	2.22E-15	SNORD22
ENST00000362031	248.41	-3.67	0.43	-8.59	9.03E-18	2.85E-15	SNX6
ENST00000484404	197.09	-3.26	0.40	-8.23	1.88E-16	5.25E-14	ITGA4
ENST00000285105	225.25	-4.79	0.60	-8.04	9.34E-16	2.4E-13	AIM1
ENST00000227503	622.14	-3.59	0.45	-8.02	1.05E-15	2.69E-13	SF1
ENST00000521331	433.73	-2.34	0.29	-8.00	1.24E-15	3.15E-13	PRKDC
ENST00000545801	165.42	-9.99	1.26	-7.96	1.78E-15	4.45E-13	ANAPC5
ENST00000445888	2151.71	-3.48	0.45	-7.67	1.71E-14	3.84E-12	TP53
ENST00000545294	274.20	-3.35	0.44	-7.65	1.98E-14	4.39E-12	UHMK1
ENST00000450465	1887.15	-28.20	3.72	-7.58	3.4E-14	7.34E-12	TNRC6A
ENST00000337437	1857.97	-27.53	3.73	-7.37	1.67E-13	3.34E-11	GGA1
ENST00000377902	3505.10	-3.05	0.42	-7.33	2.3E-13	4.5E-11	GNE
ENST00000535318	112.26	-7.53	1.04	-7.23	4.91E-13	9.22E-11	CPNE2
ENST00000409140	320.90	-2.19	0.30	-7.22	5.13E-13	9.59E-11	SPATS2L
ENST00000392692	3182.18	-4.23	0.59	-7.17	7.63E-13	1.4E-10	ECT2
ENST00000480769	1628.71	-6.49	0.92	-7.09	1.31E-12	2.32E-10	IVNS1ABP
ENST00000466787	545.05	-2.32	0.33	-7.04	1.85E-12	3.22E-10	WRB
ENST00000369750	2761.12	-2.89	0.41	-7.03	2.07E-12	3.57E-10	TPBG
ENST00000567690	37.90	-3.98	0.57	-7.01	2.36E-12	4.06E-10	CCNDBP1
ENST00000370196	335.24	-3.85	0.55	-6.97	3.13E-12	5.3E-10	TLX1
ENST00000399464	569.49	-9.88	1.43	-6.92	4.55E-12	7.59E-10	NEURL4
ENST00000434178	825.71	-7.19	1.04	-6.91	4.74E-12	7.84E-10	MGLL
ENST00000369817	8627.43	-3.15	0.47	-6.74	1.58E-11	2.43E-09	RPL10
ENST00000357006	143.57	-5.61	0.84	-6.69	2.25E-11	3.41E-09	ZFP161

ENST00000430479	353.57	-7.02	1.05	-6.68	2.39E-11	3.59E-09	ETV1
ENST00000302628	70.43	-2.32	0.35	-6.68	2.41E-11	3.63E-09	CALB2
ENST00000535581	33.33	-8.64	1.30	-6.64	3.04E-11	4.48E-09	C19orf55
ENST00000455785	424.74	-2.34	0.36	-6.59	4.33E-11	6.23E-09	STMN1
ENST00000252455	5326.20	-6.36	0.97	-6.58	4.73E-11	6.78E-09	PRKCSH
ENST00000218004	215.85	-11.34	1.74	-6.50	7.94E-11	1.1E-08	NXT2
ENST00000258530	281.38	-8.54	1.32	-6.47	9.81E-11	1.33E-08	APPL2
ENST00000358677	253.56	-2.54	0.40	-6.37	1.86E-10	2.42E-08	SPATS2L
ENST00000403258	20.35	-7.93	1.25	-6.35	2.22E-10	2.85E-08	ACTBP8
ENST00000357154	29.91	-6.03	0.95	-6.31	2.77E-10	3.49E-08	SPOCK3
ENST00000269468	129.00	-2.01	0.32	-6.28	3.29E-10	4.11E-08	MBD1
ENST00000439578	2536.12	-4.06	0.67	-6.08	1.23E-09	1.38E-07	SAR1B
ENST00000526926	610.29	-4.33	0.72	-6.01	1.85E-09	2.01E-07	ZNF623
ENST00000305762	26.41	-7.34	1.23	-5.97	2.36E-09	2.54E-07	CAMK2G
ENST00000369135	5596.30	-14.17	2.39	-5.94	2.86E-09	3.03E-07	OTUD7B
ENST00000569028	607.63	-3.70	0.63	-5.85	4.93E-09	4.99E-07	RP11-80F22.9.1
ENST00000339950	12552.90	-2.82	0.49	-5.80	6.54E-09	6.47E-07	USP1
ENST00000413081	201.37	-8.79	1.52	-5.77	7.95E-09	7.69E-07	TNRC18
ENST00000378921	1486.62	-11.68	2.04	-5.73	9.84E-09	9.34E-07	ZC3H13
ENST00000400337	748.59	-5.11	0.91	-5.65	1.63E-08	1.5E-06	COL18A1
ENST00000379597	254.01	-2.23	0.40	-5.61	2.04E-08	1.84E-06	GCNT2
ENST00000282169	554.48	-3.08	0.55	-5.57	2.6E-08	2.31E-06	UHMK1
ENST00000485978	140.15	-3.25	0.59	-5.52	3.33E-08	2.88E-06	SLC2A6
ENST00000520401	14.73	-3.69	0.67	-5.52	3.34E-08	2.89E-06	CTC-554D6.1.1
ENST00000412716	156.80	-3.03	0.55	-5.51	3.6E-08	3.1E-06	DAZAP2
ENST00000440926	152.43	-4.94	0.90	-5.48	4.17E-08	3.54E-06	SLC18A1
ENST00000396526	852.13	-5.57	1.03	-5.42	6.11E-08	5.03E-06	SNX6
ENST00000500728	4999.64	-3.63	0.67	-5.40	6.54E-08	5.36E-06	LRPAP1
ENST00000399119	21.64	-8.02	1.49	-5.39	6.97E-08	5.67E-06	DTNA
ENST00000541394	18.41	-7.79	1.45	-5.39	7.11E-08	5.78E-06	C12orf48
ENST00000379335	516.46	-3.02	0.56	-5.37	7.98E-08	6.42E-06	PHACTR1
ENST00000331456	508.62	-9.72	1.81	-5.36	8.29E-08	6.64E-06	TRAIP
ENST00000534989	1639.90	-9.31	1.74	-5.36	8.56E-08	6.83E-06	COG2
ENST00000409185	3290.43	-2.38	0.45	-5.30	1.14E-07	8.9E-06	FAM168B
ENST00000556752	2200.94	-5.86	1.11	-5.26	1.43E-07	1.08E-05	GNG2
ENST00000395325	2493.31	-3.65	0.70	-5.26	1.48E-07	1.11E-05	DNAJC6
ENST00000441843	1008.59	-2.30	0.44	-5.23	1.69E-07	1.25E-05	LACC1
ENST00000522751	612.54	-3.31	0.64	-5.14	2.68E-07	1.9E-05	ENDOV
ENST00000307885	2528.07	-3.10	0.60	-5.13	2.82E-07	2E-05	ADCY6
ENST00000282541	1655.85	-4.13	0.81	-5.13	2.92E-07	2.07E-05	GPD1L
ENST00000399054	1618.65	-8.63	1.69	-5.11	3.21E-07	2.25E-05	MKKS
ENST00000536429	10.04	-6.91	1.36	-5.09	3.66E-07	2.54E-05	PRKDC
ENST00000357122	1666.33	-3.81	0.76	-5.03	4.96E-07	3.34E-05	SPAG9
ENST00000393567	21.61	-2.80	0.56	-5.02	5.11E-07	3.43E-05	HYDIN
ENST00000394144	3133.33	-2.22	0.44	-5.00	5.67E-07	3.77E-05	TBC1D23
ENST00000007414	849.81	-5.63	1.13	-5.00	5.69E-07	3.78E-05	OSBPL7

ENST00000474050	231.24	-7.99	1.60	-4.99	6.03E-07	3.98E-05	GLIPR2
ENST00000345807	768.76	-2.41	0.48	-4.97	6.66E-07	4.35E-05	CLTB
ENST00000455537	379.38	-7.83	1.58	-4.96	7.04E-07	4.57E-05	KIF5A
ENST00000323833	910.56	-3.22	0.65	-4.92	8.85E-07	5.62E-05	TSFM
ENST00000478866	1085.38	-7.55	1.54	-4.92	8.87E-07	5.63E-05	NDUFS2
ENST00000463732	120.66	-2.79	0.57	-4.90	9.35E-07	5.88E-05	HDAC7
ENST00000357710	2296.02	-3.84	0.79	-4.89	1.03E-06	6.44E-05	PSEN1
ENST00000503162	989.54	-6.60	1.36	-4.85	1.27E-06	7.72E-05	MAEA
ENST00000368209	22562.19	-4.59	0.95	-4.84	1.29E-06	7.87E-05	HDGF
ENST00000349228	132.99	-2.16	0.45	-4.82	1.44E-06	8.6E-05	TNFSF13
ENST00000439657	294.25	-7.33	1.52	-4.81	1.52E-06	9.04E-05	LENG8
ENST00000497571	204.94	-8.82	1.84	-4.79	1.65E-06	9.69E-05	KLF6
ENST00000368068	456.71	-7.74	1.63	-4.74	2.13E-06	0.000123	MED23
ENST00000359707	3619.98	-5.79	1.22	-4.74	2.18E-06	0.000125	G3BP2
ENST00000497692	40.15	-2.34	0.49	-4.73	2.23E-06	0.000127	CCNY
ENST00000360538	3589.47	-4.39	0.93	-4.73	2.25E-06	0.000129	TOPORS
ENST00000556769	17.13	-3.54	0.76	-4.68	2.82E-06	0.000156	ERO1L
ENST00000397872	70.52	-2.34	0.50	-4.68	2.87E-06	0.000159	THOC5
ENST00000504937	146.49	-5.51	1.18	-4.68	2.88E-06	0.000159	TP53
ENST00000510624	267.66	-2.43	0.52	-4.65	3.33E-06	0.00018	LEF1
ENST00000569334	33.06	-2.25	0.49	-4.63	3.59E-06	0.000193	VENTXP1
ENST00000553532	682.26	-4.59	0.99	-4.63	3.72E-06	0.000198	TIMELESS
ENST00000531480	141.58	-8.28	1.80	-4.60	4.2E-06	0.00022	CPSF1
ENST00000540964	30.66	-4.28	0.94	-4.57	4.86E-06	0.00025	TLR5
ENST00000326804	599.12	-4.51	0.99	-4.55	5.32E-06	0.000271	ZNF274
ENST00000348222	142.85	-3.87	0.86	-4.52	6.31E-06	0.000313	DGUOK
ENST00000449290	6.46	-6.28	1.39	-4.51	6.33E-06	0.000314	SPRYD5
ENST00000342056	24.43	-8.20	1.82	-4.51	6.62E-06	0.000326	KCNQ5
ENST00000484734	1630.21	-2.15	0.48	-4.48	7.38E-06	0.00036	THBS1
ENST00000357480	7134.19	-5.85	1.30	-4.48	7.4E-06	0.00036	NFE2L1
ENST00000396822	808.03	-5.89	1.32	-4.47	7.76E-06	0.000374	SNAI2
ENST00000321566	255.07	-9.14	2.04	-4.47	7.86E-06	0.000378	AC011139.1.1
ENST00000368518	310.79	-6.82	1.53	-4.46	8.04E-06	0.000385	C1orf43
ENST00000532461	2329.22	-2.69	0.60	-4.45	8.44E-06	0.000401	RDX
ENST00000423280	594.46	-5.75	1.29	-4.44	8.86E-06	0.000418	PPP1R7
ENST00000531727	139.47	-3.42	0.77	-4.42	1.01E-05	0.000465	CPSF1
ENST00000538582	759.16	-6.77	1.53	-4.42	1.01E-05	0.000466	PYROXD1
ENST00000473686	25.54	-4.53	1.03	-4.39	1.11E-05	0.000508	EFCAB2
ENST00000381227	5473.38	-2.48	0.56	-4.39	1.14E-05	0.000522	POLR2B
ENST00000524037	25.59	-3.47	0.80	-4.36	1.29E-05	0.000576	C8orf83
ENST00000559889	32.22	-3.63	0.84	-4.34	1.4E-05	0.00062	IDH3A
ENST00000299341	85.51	-3.63	0.84	-4.33	1.51E-05	0.000663	STXBP4
ENST00000537034	13643.32	-4.74	1.10	-4.32	1.54E-05	0.000672	CTNNA1
ENST00000288022	19.01	-4.93	1.14	-4.32	1.56E-05	0.000681	PDF
ENST00000374098	229.81	-4.69	1.10	-4.28	1.84E-05	0.000792	TIMM23B
ENST00000362003	2001.91	-4.80	1.12	-4.27	1.95E-05	0.000832	PCGF3

ENST00000547587	3230.27	-5.92	1.39	-4.26	2.08E-05	0.000881	PFKM
ENST00000559020	413.15	-2.84	0.67	-4.24	2.27E-05	0.00095	CERS2
ENST00000476438	111.11	-3.40	0.81	-4.21	2.54E-05	0.001051	PIGQ
ENST00000371941	5339.76	-2.68	0.64	-4.20	2.62E-05	0.001076	PREX1
ENST00000316985	394.91	-3.10	0.74	-4.18	2.97E-05	0.001199	WHSC1L1
ENST00000505472	2079.10	-3.87	0.93	-4.17	3.01E-05	0.001211	SEC31A
ENST00000264658	626.53	-2.79	0.67	-4.16	3.15E-05	0.001259	FBXL20
ENST00000246015	125.51	-7.11	1.72	-4.14	3.53E-05	0.001392	PSMF1
ENST00000380050	2155.84	-2.85	0.69	-4.13	3.62E-05	0.001417	CDK14
ENST00000342697	434.71	-3.87	0.94	-4.13	3.65E-05	0.001426	TP53INP1
ENST00000432763	3937.90	-5.72	1.39	-4.11	3.88E-05	0.001504	SUPT5H
ENST00000394945	7250.00	-4.04	0.98	-4.11	3.94E-05	0.00152	SPOCK1
ENST00000527243	214.03	-5.34	1.30	-4.09	4.24E-05	0.001615	CTSB
ENST00000468227	2421.51	-3.43	0.85	-4.05	5.03E-05	0.001867	UBA5
ENST00000431828	137.69	-5.48	1.35	-4.05	5.18E-05	0.001915	KANSL3
ENST00000461167	10.18	-2.70	0.67	-4.03	5.54E-05	0.002023	FAM129A
ENST00000252891	73.76	-6.60	1.64	-4.02	5.79E-05	0.002102	NUMBL
ENST00000438886	133.75	-2.66	0.66	-4.01	6.14E-05	0.002204	APEX1
ENST00000548214	75.71	-2.38	0.60	-4.00	6.2E-05	0.00222	DYNLL1
ENST00000432072	149.69	-2.45	0.61	-4.00	6.22E-05	0.002225	FN1
ENST00000349157	1139.33	-5.45	1.37	-3.98	6.77E-05	0.00239	PUF60
ENST00000562945	14.32	-4.94	1.24	-3.98	6.79E-05	0.002395	RP11-488I20.9.1
ENST00000366997	8001.52	-4.65	1.17	-3.97	7.07E-05	0.002475	LPGAT1
ENST00000369809	137.13	-2.53	0.64	-3.97	7.08E-05	0.002475	DNASE1L1
ENST00000397231	40.17	-3.97	1.00	-3.97	7.17E-05	0.002496	MYH9
ENST00000058691	471.43	-7.00	1.77	-3.96	7.55E-05	0.002609	HEBP2
ENST00000298004	24.99	-3.80	0.96	-3.95	7.88E-05	0.002706	PIGO
ENST00000412964	243.02	-4.21	1.07	-3.95	7.91E-05	0.002715	CCDC138
ENST00000393256	136.57	-2.55	0.65	-3.94	8.3E-05	0.002829	BCL2L11
ENST00000238738	289.27	-5.42	1.38	-3.93	8.52E-05	0.002888	RHOQ
ENST00000567593	1150.17	-2.81	0.72	-3.91	9.26E-05	0.003099	C16orf61
ENST00000418533	32.65	-2.02	0.52	-3.90	9.6E-05	0.003195	LTBP1
ENST00000531413	6199.98	-5.26	1.35	-3.88	0.000102	0.003371	CFL1
ENST00000310924	225.64	-2.23	0.58	-3.88	0.000105	0.003451	TBC1D16
ENST00000395644	1679.60	-3.88	1.00	-3.88	0.000106	0.003487	C15orf44
ENST00000372739	7769.76	-3.09	0.80	-3.87	0.000109	0.003559	SPTAN1
ENST00000219473	1132.71	-4.49	1.16	-3.86	0.000114	0.003679	USP10
ENST00000503241	100.59	-2.42	0.63	-3.85	0.000117	0.003769	ENSA
ENST00000338920	5049.48	-4.91	1.28	-3.84	0.000125	0.003981	PRRC2C
ENST00000396401	3157.70	-5.03	1.31	-3.83	0.000128	0.004059	TCEA1
ENST00000559775	1789.81	-2.12	0.55	-3.82	0.000131	0.00414	ZFAND6
ENST00000375018	341.29	-4.84	1.27	-3.81	0.000141	0.004411	ANKS6
ENST00000372841	137.35	-4.26	1.13	-3.77	0.00016	0.004905	FUT11
ENST00000428926	415.85	-3.45	0.92	-3.76	0.000168	0.005109	C14orf159
ENST00000412601	3653.76	-2.51	0.67	-3.76	0.00017	0.005158	PRKCSH
ENST00000463545	4.67	-5.81	1.55	-3.75	0.000174	0.005275	DDX50P2

ENST00000433404	1121.10	-2.85	0.76	-3.75	0.000179	0.005381	SAFB
ENST00000368843	985.89	-3.93	1.05	-3.74	0.000182	0.005474	SNX27
ENST00000514940	30.50	-6.05	1.62	-3.73	0.000189	0.005646	ADD1
ENST00000353234	275.98	-4.35	1.17	-3.73	0.000191	0.005675	ANKS6
ENST00000289619	6.57	-3.76	1.01	-3.73	0.000195	0.005779	PAGE5
ENST00000505587	292.80	-2.45	0.66	-3.72	0.0002	0.005896	CCNH
ENST00000316341	2832.45	-8.30	2.24	-3.71	0.000206	0.006022	SLC12A4
ENST00000309428	1445.68	-4.14	1.11	-3.71	0.000207	0.006044	FAM134C
ENST00000433670	11.71	-2.31	0.62	-3.71	0.000208	0.006078	PXDN
ENST00000447027	219.66	-10.40	2.81	-3.71	0.000208	0.006086	MUC5B
ENST00000423692	38.29	-4.77	1.29	-3.71	0.000209	0.006107	SRRT
ENST00000449277	3190.57	-3.88	1.05	-3.71	0.00021	0.006119	MEF2A
ENST00000394688	195.18	-4.05	1.09	-3.70	0.000212	0.006168	BRD7
ENST00000258840	76.18	-6.14	1.66	-3.70	0.000217	0.00626	RALGAPA1
ENST00000376526	1174.52	-2.40	0.65	-3.69	0.000221	0.006346	LENG8
ENST00000482141	230.33	-4.03	1.09	-3.69	0.000222	0.006367	NRM
ENST00000397165	97.86	-2.37	0.64	-3.69	0.000223	0.006416	ZNF682
ENST00000340957	18.51	-7.80	2.11	-3.69	0.000227	0.006508	FAM131A
ENST00000328631	29.28	-4.74	1.29	-3.68	0.000234	0.00667	IP6K2
ENST00000439576	117.38	-4.92	1.34	-3.68	0.000236	0.006704	ACAN
ENST00000525681	41.36	-4.00	1.09	-3.67	0.000239	0.006795	EIF4G2
ENST00000340647	19.35	-3.55	0.98	-3.64	0.000278	0.007676	BRCC3
ENST00000557481	5.24	-5.98	1.65	-3.63	0.000283	0.007785	CTD-3049M7.1.1
ENST00000391781	216.90	-3.58	0.99	-3.63	0.000287	0.007882	ZNF320
ENST00000404104	25.81	-5.81	1.60	-3.63	0.000287	0.007886	SETD7
ENST00000317538	62.50	-6.68	1.85	-3.61	0.000305	0.00827	ZMYM6
ENST00000307641	723.75	-4.45	1.24	-3.60	0.000315	0.008495	NKIRAS2
ENST00000470683	17.41	-2.53	0.70	-3.60	0.000319	0.008569	ST7L
ENST00000525843	1374.48	-3.55	0.99	-3.59	0.000325	0.008698	PUM1
ENST00000504373	1418.01	-3.38	0.94	-3.59	0.000329	0.008771	CD164
ENST00000441222	2021.32	-2.66	0.75	-3.56	0.000367	0.009548	USP46
ENST00000466764	2544.19	-2.43	0.68	-3.56	0.000369	0.009589	RBM3
ENST00000376520	241.11	-5.77	1.62	-3.55	0.000378	0.009778	TLE4
ENST00000381237	3371.11	-4.09	1.15	-3.55	0.000386	0.009938	SLC39A14

bMean: mean of sequencing depth normalized counts.

lfc: log 2 fold change.

lfcSE: lfc Standard Error

stat: Wald-statistics value.

padj: FDR adjusted pvalue.

Table S4. Ribo-seq and RNA-seq fold-change and p-values for RNAs with higher translational efficiency in NSUN5 shRNA depleted DBTRG-05MG cells. Related to Figure S6.

		Ribo-Seq						RNA-Seq					
EnsemblID	Gene Name	Base Mean	log2Fold Change	lfc SE	stat	pvalue	padj	Base Mean	log2Fold Change	lfc SE	stat	pvalue	padj
ENST00000444053	AAMP	4.86	4.64	1.75	2.66	7.78E-03	5.78E-01	2343.96	-0.26	0.32	-0.81	4.16E-01	1.00E+00
ENST00000489767	AAMP	9.41	20.87	3.03	6.89	5.72E-12	1.35E-09	1392.81	-0.52	0.27	-1.92	5.44E-02	7.29E-01
ENST00000265662	ABCA2	7.21	3.27	0.95	3.46	5.35E-04	6.89E-02	0.00	NA	NA	NA	NA	NA
ENST00000301732	ABCA3	9.32	2.40	0.90	2.67	7.54E-03	5.64E-01	3265.27	0.45	1.06	0.42	6.75E-01	1.00E+00
ENST00000427699	ABCC3	4.27	5.66	2.31	2.45	1.43E-02	8.32E-01	89.29	-0.66	1.38	-0.48	6.35E-01	1.00E+00
ENST00000395008	ABHD14B	5.92	6.11	1.85	3.30	9.64E-04	1.15E-01	1626.45	0.45	0.80	0.56	5.77E-01	1.00E+00
ENST00000473912	ABHD14B	2.40	3.54	1.26	2.81	4.92E-03	4.16E-01	29.76	0.49	0.61	0.80	4.22E-01	1.00E+00
ENST00000444970	ABL1	11.91	1.21	0.55	2.22	2.67E-02	9.96E-01	153.48	-0.79	1.58	-0.50	6.16E-01	1.00E+00
ENST00000369267	ABLIM1	9.32	2.49	0.93	2.67	7.52E-03	5.64E-01	4.42	1.90	2.78	0.69	4.93E-01	1.00E+00
ENST00000560397	AC012652.1.1	5.22	5.94	3.03	1.96	5.01E-02	9.96E-01	18.20	-2.23	1.18	-1.90	5.80E-02	7.47E-01
ENST00000560411	AC012652.1.1	4.59	5.76	3.03	1.90	5.76E-02	9.96E-01	10.23	-0.24	1.80	-0.13	8.93E-01	1.00E+00
ENST00000532792	ACAT1	6.17	4.51	1.79	2.52	1.16E-02	7.47E-01	21.26	0.54	0.77	0.70	4.84E-01	1.00E+00
ENST00000478010	ACO2	4.84	4.62	2.42	1.91	5.57E-02	9.96E-01	135.67	-0.03	0.61	-0.05	9.63E-01	1.00E+00
ENST00000487599	ACTR1A	5.38	5.98	3.03	1.97	4.88E-02	9.96E-01	10064.75	-0.65	2.02	-0.32	7.46E-01	1.00E+00
ENST00000494549	ACTR1A	5.54	5.41	2.74	1.97	4.84E-02	9.96E-01	16.28	-1.32	1.25	-1.05	2.94E-01	1.00E+00
ENST00000241416	ACVR2A	10.87	1.37	0.49	2.78	5.45E-03	4.48E-01	1030.02	0.79	0.33	2.37	1.76E-02	4.25E-01
ENST00000464097	ADA	2.83	2.77	1.37	2.02	4.31E-02	9.96E-01	409.35	-1.00	0.69	-1.44	1.49E-01	9.65E-01
ENST00000396140	ADAM10	5.76	6.08	3.03	2.01	4.49E-02	9.96E-01	233.93	-2.27	3.43	-0.66	5.08E-01	1.00E+00
ENST00000498707	ADAMTS9	33.66	1.51	0.69	2.18	2.94E-02	9.96E-01	2233.41	1.64	1.91	0.86	3.89E-01	1.00E+00
ENST00000294016	ADCY9	6.11	1.33	0.70	1.90	5.80E-02	9.96E-01	1546.65	0.19	0.40	0.48	6.31E-01	1.00E+00
ENST00000569534	ADPGK	5.77	6.08	3.03	2.01	4.49E-02	9.96E-01	0.47	0.50	2.16	0.23	8.17E-01	NA
ENST00000360995	AEBP2	3.42	2.58	1.24	2.07	3.80E-02	9.96E-01	219.11	1.15	1.48	0.78	4.38E-01	1.00E+00
ENST00000264409	AGPAT9	18.93	7.80	1.72	4.52	6.15E-06	1.02E-03	1161.55	0.36	1.12	0.33	7.45E-01	1.00E+00
ENST00000217426	AHCY	20.18	21.93	3.03	7.24	4.40E-13	2.39E-10	6925.53	0.60	0.70	0.85	3.94E-01	1.00E+00
ENST00000373176	AK1	56.51	0.64	0.28	2.26	2.36E-02	9.96E-01	5460.61	0.16	0.38	0.42	6.75E-01	1.00E+00
ENST00000374525	AKAP2	7.84	6.52	3.03	2.15	3.14E-02	9.96E-01	3493.96	2.98	1.25	2.38	1.73E-02	4.21E-01
ENST00000426422	AKR1B1	3.07	5.17	2.43	2.13	3.33E-02	9.96E-01	94.51	-0.02	0.42	-0.06	9.53E-01	1.00E+00
ENST00000441729	ALAS1	10.14	20.97	3.03	6.92	4.43E-12	1.08E-09	348.28	0.14	0.69	0.21	8.37E-01	1.00E+00
ENST00000470756	ALCAM	30.12	0.73	0.34	2.12	3.36E-02	9.96E-01	171.41	1.76	0.98	1.79	7.27E-02	8.12E-01
ENST00000297785	ALDH1A1	45.40	2.45	0.46	5.31	1.12E-07	1.99E-05	3532.52	1.47	0.21	6.97	3.25E-12	3.46E-09
ENST00000395240	ALDOA	117.20	1.63	0.53	3.10	1.92E-03	2.04E-01	973.64	-0.41	0.39	-1.06	2.90E-01	1.00E+00
ENST00000566846	ALDOA	9.69	6.83	2.38	2.88	4.01E-03	3.60E-01	46.03	-0.78	0.53	-1.48	1.38E-01	9.52E-01
ENST00000566897	ALDOA	18.87	6.31	2.94	2.14	3.20E-02	9.96E-01	706.85	0.16	0.65	0.25	8.04E-01	1.00E+00
ENST00000461415	ALG3	1.50	3.52	1.51	2.33	1.96E-02	9.96E-01	35.86	0.42	0.64	0.66	5.09E-01	1.00E+00
ENST00000331204	ALYREF	54.66	2.45	1.01	2.42	1.55E-02	8.78E-01	58.09	-0.34	0.60	-0.56	5.78E-01	1.00E+00
ENST00000369864	AMIGO1	4.52	1.71	0.72	2.38	1.71E-02	9.23E-01	1234.41	0.55	0.37	1.49	1.37E-01	9.50E-01
ENST00000447274	ANKRD13D	1.77	4.39	2.27	1.94	5.28E-02	9.96E-01	766.27	0.05	0.24	0.22	8.29E-01	1.00E+00
ENST00000550693	ANKS1B	4.97	3.44	1.73	1.99	4.64E-02	9.96E-01	0.59	0.77	4.06	0.19	8.50E-01	NA
ENST00000447173	ANP32B	12.33	21.25	3.03	7.01	2.32E-12	6.86E-10	137.39	-0.24	0.69	-0.35	7.26E-01	1.00E+00
ENST00000376911	ANXA1	307.17	3.89	1.78	2.18	2.93E-02	9.96E-01	9789.82	0.12	0.39	0.31	7.55E-01	1.00E+00
ENST00000515017	ANXA5	11.76	5.93	2.92	2.03	4.25E-02	9.96E-01	23241.27	-0.42	0.26	-1.63	1.02E-01	8.97E-01
ENST00000522664	ANXA6	7.95	20.62	3.03	6.80	1.01E-11	2.17E-09	213.91	-0.04	0.39	-0.10	9.21E-01	1.00E+00
ENST00000415298	AP1S3	22.41	1.73	0.71	2.44	1.46E-02	8.43E-01	133.53	-1.86	1.40	-1.33	1.85E-01	9.92E-01
ENST00000527917	AP2A2	6.25	1.65	0.72	2.28	2.28E-02	9.96E-01	2.51	-0.01	1.07	-0.01	9.94E-01	NA
ENST00000292807	AP2M1	10.81	6.99	3.03	2.31	2.11E-02	9.96E-01	29821.26	-0.26	0.38	-0.70	4.86E-01	1.00E+00
ENST00000461733	AP2M1	12.41	21.11	3.03	6.97	3.16E-12	8.70E-10	1418.56	0.10	0.55	0.18	8.54E-01	1.00E+00
ENST00000530493	APLP2	4.68	4.00	1.34	3.00	2.73E-03	2.69E-01	853.71	0.10	0.32	0.31	7.55E-01	1.00E+00
ENST00000319136	APOL1	16.79	2.36	0.66	3.59	3.30E-04	4.44E-02	25.92	1.19	1.65	0.72	4.71E-01	1.00E+00
ENST00000456209	APP	9.36	6.78	3.03	2.24	2.52E-02	9.96E-01	59.45	-0.16	0.58	-0.28	7.81E-01	1.00E+00
ENST00000495878	ARAP1	5.04	5.89	2.33	2.53	1.13E-02	7.31E-01	6.94	1.58	1.00	1.58	1.14E-01	9.21E-01
ENST00000356641	ARHGEF12	4.95	5.87	3.03	1.93	5.30E-02	9.96E-01	4843.76	2.00	2.16	0.93	3.54E-01	1.00E+00
ENST00000539576	ARL6IP4	2.47	4.85	2.50	1.94	5.24E-02	9.96E-01	227.26	-0.55	0.55	-1.00	3.19E-01	1.00E+00
ENST00000476566	ARPC3	5.44	3.92	1.81	2.17	3.02E-02	9.96E-01	33.07	-0.37	0.65	-0.57	5.68E-01	1.00E+00

ENST00000294742	ARPC5	5.51	5.41	2.81	1.92	5.44E-02	9.96E-01	875.28	-0.46	0.54	-0.86	3.87E-01	1.00E+00
ENST00000336689	ASAP3	8.42	2.55	0.91	2.79	5.34E-03	4.40E-01	1511.69	1.04	2.29	0.45	6.51E-01	1.00E+00
ENST00000475693	ATG4B	2.99	4.51	2.09	2.16	3.05E-02	9.96E-01	1598.11	-0.65	0.71	-0.91	3.63E-01	1.00E+00
ENST00000236959	ATIC	5.30	5.96	3.03	1.97	4.92E-02	9.96E-01	1901.96	1.17	0.57	2.04	4.12E-02	6.50E-01
ENST00000339159	ATP1A1	87.83	23.93	3.03	7.91	2.64E-15	9.40E-12	359.64	-0.83	0.94	-0.89	3.75E-01	1.00E+00
ENST00000488733	ATP1A1	18.54	1.17	0.62	1.88	5.98E-02	9.96E-01	512.51	-0.80	0.82	-0.97	3.31E-01	1.00E+00
ENST00000537345	ATP1A1	97.35	24.12	3.03	7.97	1.58E-15	7.61E-12	13011.30	0.41	0.80	0.51	6.10E-01	1.00E+00
ENST00000514654	ATP2C1	18.17	7.74	3.03	2.56	1.06E-02	7.01E-01	1775.30	0.45	0.83	0.54	5.87E-01	1.00E+00
ENST00000412323	ATP5A1P2	51.35	0.79	0.35	2.29	2.20E-02	9.96E-01	3.92	0.62	1.07	0.58	5.63E-01	1.00E+00
ENST00000553007	ATP5B	6.10	20.13	3.03	6.64	3.16E-11	6.06E-09	199.13	0.09	0.56	0.15	8.79E-01	1.00E+00
ENST00000464154	ATP5F1	35.77	22.72	3.03	7.51	6.09E-14	6.43E-11	32.97	0.91	0.52	1.74	8.23E-02	8.46E-01
ENST00000566322	ATP6V0D1	4.87	5.84	3.03	1.93	5.41E-02	9.96E-01	12.17	0.31	1.13	0.27	7.87E-01	1.00E+00
ENST00000558401	B2M	50.76	2.44	1.11	2.19	2.86E-02	9.96E-01	13994.50	-0.03	0.32	-0.08	9.35E-01	1.00E+00
ENST00000379731	B4GALT1	115.77	0.55	0.25	2.16	3.09E-02	9.96E-01	17264.54	0.17	0.32	0.52	6.06E-01	1.00E+00
ENST00000467604	B4GALT4	6.37	1.82	0.91	2.01	4.46E-02	9.96E-01	782.12	-1.11	0.68	-1.64	1.01E-01	8.94E-01
ENST00000328735	BACE2	5.64	6.05	3.03	2.00	4.59E-02	9.96E-01	455.47	0.01	0.63	0.02	9.87E-01	1.00E+00
ENST00000466844	BAZ1B	7.05	2.40	0.97	2.48	1.31E-02	7.89E-01	42.12	-1.37	0.50	-2.75	5.91E-03	2.26E-01
ENST00000458587	BCAP31	22.61	5.31	1.48	3.59	3.36E-04	4.50E-02	18776.05	-1.08	0.99	-1.09	2.75E-01	1.00E+00
ENST00000482958	BCHE	2.63	2.38	1.14	2.09	3.64E-02	9.96E-01	16.09	-1.78	2.08	-0.85	3.94E-01	1.00E+00
ENST00000398117	BCL2	2.01	2.73	1.14	2.39	1.66E-02	9.09E-01	252.56	0.88	0.30	2.92	3.52E-03	1.64E-01
ENST00000422920	BCL2L1	10.99	7.01	2.73	2.57	1.03E-02	6.89E-01	196.89	1.01	0.63	1.61	1.08E-01	9.10E-01
ENST00000520626	BMP1	1.98	4.54	2.39	1.90	5.73E-02	9.96E-01	23.32	-0.16	2.05	-0.08	9.37E-01	1.00E+00
ENST00000254900	BRD8	20.90	0.97	0.45	2.17	2.97E-02	9.96E-01	50.11	-1.03	1.09	-0.94	3.45E-01	1.00E+00
ENST00000367846	BRP44	4.43	5.72	1.69	3.39	6.91E-04	8.52E-02	2772.69	0.13	0.37	0.35	7.24E-01	1.00E+00
ENST00000533084	BSDC1	2.88	5.07	2.44	2.08	3.77E-02	9.96E-01	8.22	0.18	0.86	0.21	8.32E-01	1.00E+00
ENST00000456893	BUD31	6.30	6.21	3.03	2.05	4.07E-02	9.96E-01	29.86	0.18	0.68	0.27	7.90E-01	1.00E+00
ENST00000314641	BVES	87.68	0.67	0.24	2.83	4.68E-03	4.04E-01	16478.31	0.66	0.36	1.84	6.59E-02	7.87E-01
ENST00000450637	BZW1	5.53	6.02	3.03	1.98	4.73E-02	9.96E-01	55.59	-0.53	2.35	-0.23	8.21E-01	1.00E+00
ENST00000525256	C11orf58	3.90	3.74	1.39	2.70	7.00E-03	5.38E-01	276.48	-0.05	0.71	-0.08	9.39E-01	1.00E+00
ENST00000549177	C12orf10	1.83	3.79	1.97	1.92	5.43E-02	9.96E-01	309.86	-0.77	0.60	-1.29	1.96E-01	1.00E+00
ENST00000443656	C17orf70	8.94	1.03	0.53	1.94	5.18E-02	9.96E-01	48.88	-0.91	0.74	-1.22	2.23E-01	1.00E+00
ENST00000366687	C1orf96	2.42	4.84	2.28	2.12	3.39E-02	9.96E-01	1290.11	0.02	0.33	0.06	9.54E-01	1.00E+00
ENST00000379286	C20orf30	5.51	5.42	2.78	1.95	5.14E-02	9.96E-01	319.13	-0.34	2.54	-0.13	8.94E-01	1.00E+00
ENST00000480786	C21orf33	5.45	6.01	2.31	2.60	9.41E-03	6.48E-01	274.69	-0.68	0.44	-1.56	1.18E-01	9.29E-01
ENST00000495007	C21orf33	5.78	6.09	2.32	2.62	8.73E-03	6.23E-01	379.33	-0.06	0.28	-0.21	8.33E-01	1.00E+00
ENST00000507191	C5orf15	7.19	20.49	3.03	6.76	1.39E-11	2.80E-09	29.88	0.27	0.78	0.35	7.30E-01	1.00E+00
ENST00000520464	C5orf41	7.66	2.48	1.14	2.19	2.89E-02	9.96E-01	18.71	-1.17	1.22	-0.96	3.36E-01	1.00E+00
ENST00000275227	C6orf192	1.67	4.30	2.28	1.88	5.96E-02	9.96E-01	1322.37	0.18	0.32	0.54	5.88E-01	1.00E+00
ENST00000438444	C7orf44	5.62	2.12	0.99	2.15	3.19E-02	9.96E-01	676.50	-0.54	0.75	-0.72	4.74E-01	1.00E+00
ENST00000481410	C7orf49	1.83	3.11	1.45	2.14	3.21E-02	9.96E-01	173.93	-0.02	0.80	-0.02	9.81E-01	1.00E+00
ENST00000348904	C7orf57	3.23	5.25	1.65	3.19	1.43E-03	1.61E-01	238.02	1.82	0.55	3.32	9.08E-04	6.74E-02
ENST00000297613	C9orf23	18.34	0.87	0.45	1.95	5.16E-02	9.96E-01	810.08	0.24	0.29	0.83	4.09E-01	1.00E+00
ENST00000333916	C9orf82	8.41	1.90	0.71	2.65	7.96E-03	5.87E-01	290.87	0.47	0.73	0.65	5.16E-01	1.00E+00
ENST00000488630	C9orf89	7.71	6.50	2.41	2.70	7.01E-03	5.38E-01	521.97	0.46	0.25	1.84	6.63E-02	7.89E-01
ENST00000542447	CADM1	6.81	6.32	2.47	2.56	1.04E-02	6.94E-01	44.86	0.11	0.43	0.26	7.97E-01	1.00E+00
ENST00000265431	CALB1	11.58	5.39	0.98	5.51	3.66E-08	6.60E-06	966.18	1.91	0.72	2.63	8.46E-03	2.81E-01
ENST00000497376	CALB1	12.81	3.47	1.09	3.17	1.51E-03	1.67E-01	5.88	2.34	0.90	2.60	9.19E-03	2.96E-01
ENST00000342760	CALCOCO1	4.48	5.72	2.48	2.31	2.10E-02	9.96E-01	8.27	0.43	0.89	0.48	6.31E-01	1.00E+00
ENST00000393118	CALD1	5.19	20.04	3.03	6.61	3.89E-11	7.35E-09	169.49	0.10	0.93	0.11	9.13E-01	1.00E+00
ENST00000394524	CAMK2D	5.38	5.98	3.03	1.97	4.87E-02	9.96E-01	1817.48	0.65	0.37	1.78	7.47E-02	8.20E-01
ENST00000297156	CAMLG	22.04	1.51	0.71	2.13	3.30E-02	9.96E-01	2370.37	0.04	0.21	0.17	8.67E-01	1.00E+00
ENST00000372792	CAP1	26.08	22.29	3.03	7.36	1.82E-13	1.32E-10	7493.28	-0.25	0.60	-0.42	6.77E-01	1.00E+00
ENST00000372794	CAP1	26.11	22.29	3.03	7.36	1.82E-13	1.32E-10	11699.93	-0.40	0.26	-1.57	1.17E-01	9.27E-01
ENST00000372798	CAP1	9.36	20.86	3.03	6.89	5.76E-12	1.35E-09	36.17	0.55	0.76	0.73	4.67E-01	1.00E+00
ENST00000372805	CAP1	52.84	23.27	3.03	7.69	1.49E-14	2.62E-11	146.00	-0.02	0.66	-0.03	9.72E-01	1.00E+00
ENST00000527323	CAPN1	51.47	9.24	1.88	4.92	8.75E-07	1.52E-04	5909.66	-2.02	1.61	-1.25	2.10E-01	1.00E+00
ENST00000463997	CAPN2	21.81	1.44	0.74	1.93	5.33E-02	9.96E-01	1093.99	-0.14	0.39	-0.35	7.28E-01	1.00E+00
ENST00000496161	CAPZA2	17.21	3.78	1.06	3.56	3.64E-04	4.83E-02	9.25	-0.83	1.02	-0.81	4.19E-01	1.00E+00

ENST00000361962	CASK	2.55	4.91	2.28	2.15	3.14E-02	9.96E-01	18.24	-0.28	2.07	-0.13	8.93E-01	1.00E+00
ENST00000338252	CAST	26.72	21.44	3.03	7.08	1.43E-12	5.02E-10	60.54	0.23	1.72	0.13	8.94E-01	1.00E+00
ENST00000348386	CAST	12.72	21.29	3.03	7.03	2.11E-12	6.34E-10	113.33	0.63	0.73	0.87	3.85E-01	1.00E+00
ENST00000515063	CAST	27.97	22.38	3.03	7.39	1.43E-13	1.13E-10	249.03	0.97	0.68	1.43	1.52E-01	9.68E-01
ENST00000337620	CBX3	6.40	5.03	2.54	1.98	4.79E-02	9.96E-01	1004.75	-0.93	0.27	-3.40	6.72E-04	5.44E-02
ENST00000225831	CCL2	96.18	0.67	0.26	2.57	1.01E-02	6.80E-01	8129.80	0.48	0.36	1.34	1.79E-01	9.90E-01
ENST00000507798	CCNB1	6.95	6.35	3.03	2.09	3.62E-02	9.96E-01	268.27	-0.78	0.60	-1.29	1.98E-01	1.00E+00
ENST00000398160	CCNB1IP1	4.77	5.81	3.03	1.91	5.57E-02	9.96E-01	20.03	3.43	2.11	1.63	1.04E-01	9.02E-01
ENST00000227507	CCND1	178.51	0.62	0.26	2.34	1.91E-02	9.80E-01	5941.65	0.13	0.70	0.19	8.49E-01	1.00E+00
ENST00000564630	CCNDBP1	4.40	3.82	1.93	1.98	4.80E-02	9.96E-01	242.76	0.09	0.97	0.10	9.24E-01	1.00E+00
ENST00000566833	CCNDBP1	5.38	5.99	3.03	1.97	4.84E-02	9.96E-01	29.66	-0.10	1.02	-0.10	9.20E-01	1.00E+00
ENST00000512163	CCNG1	7.31	20.52	3.03	6.77	1.29E-11	2.66E-09	40.41	4.15	1.51	2.74	6.07E-03	2.30E-01
ENST00000532045	CD151	7.84	5.93	2.74	2.16	3.07E-02	9.96E-01	1726.95	-0.26	0.44	-0.59	5.56E-01	1.00E+00
ENST00000434472	CD44	123.34	2.98	1.05	2.83	4.68E-03	4.04E-01	661.23	0.42	0.38	1.10	2.71E-01	1.00E+00
ENST00000531141	CD44	6.43	2.14	1.12	1.91	5.68E-02	9.96E-01	91.61	0.48	0.49	0.98	3.29E-01	1.00E+00
ENST00000527926	CD59	8.17	6.58	3.03	2.17	2.98E-02	9.96E-01	464.62	-0.38	0.28	-1.32	1.87E-01	9.94E-01
ENST00000468153	CD81	31.27	5.21	1.24	4.20	2.63E-05	4.17E-03	14397.03	-0.21	0.43	-0.48	6.28E-01	1.00E+00
ENST00000382518	CD9	5.25	5.95	3.03	1.96	5.00E-02	9.96E-01	0.81	-0.80	3.92	-0.20	8.38E-01	NA
ENST00000375310	CDC16	5.86	20.21	3.03	6.67	2.63E-11	5.09E-09	1451.55	-0.12	1.50	-0.08	9.39E-01	1.00E+00
ENST00000380665	CDCA2	5.13	20.03	3.03	6.60	4.03E-11	7.57E-09	2065.24	-0.22	0.38	-0.58	5.65E-01	1.00E+00
ENST00000480353	CDK9	4.53	5.74	3.03	1.89	5.86E-02	9.96E-01	324.15	0.57	0.36	1.61	1.06E-01	9.07E-01
ENST00000405375	CDKN1A	23.09	7.50	1.92	3.91	9.06E-05	1.34E-02	8491.21	0.84	0.29	2.92	3.45E-03	1.62E-01
ENST00000478800	CDKN1A	18.02	7.73	3.03	2.55	1.07E-02	7.06E-01	19.68	-0.41	0.93	-0.44	6.58E-01	1.00E+00
ENST00000337090	CEP63	5.36	1.64	0.86	1.89	5.85E-02	9.96E-01	1030.20	-0.04	0.29	-0.13	8.94E-01	1.00E+00
ENST00000309534	CGGBP1	4.91	4.63	2.29	2.02	4.35E-02	9.96E-01	1524.73	-1.20	1.13	-1.06	2.89E-01	1.00E+00
ENST00000523865	CHCHD10	24.82	0.81	0.42	1.90	5.68E-02	9.96E-01	970.61	-0.08	0.83	-0.10	9.24E-01	1.00E+00
ENST00000524737	CHEK1	9.08	6.74	3.03	2.22	2.61E-02	9.96E-01	875.13	0.17	0.20	0.85	3.94E-01	1.00E+00
ENST00000549260	CHST11	8.04	6.56	3.03	2.16	3.04E-02	9.96E-01	1670.30	0.80	0.94	0.85	3.93E-01	1.00E+00
ENST00000567518	CIAPIN1	4.91	3.30	1.75	1.89	5.93E-02	9.96E-01	1528.38	-0.15	1.07	-0.14	8.88E-01	1.00E+00
ENST00000273986	CISD2	1.61	4.26	2.26	1.88	5.98E-02	9.96E-01	2939.06	-0.36	0.23	-1.61	1.07E-01	9.07E-01
ENST00000553878	CKB	17.12	3.21	1.24	2.58	9.77E-03	6.68E-01	89.60	2.25	1.33	1.69	9.12E-02	8.70E-01
ENST00000555366	CKB	9.08	1.91	0.98	1.95	5.09E-02	9.96E-01	227.51	0.12	0.56	0.22	8.29E-01	1.00E+00
ENST00000513761	CLCN3	38.69	0.70	0.36	1.97	4.85E-02	9.96E-01	4263.93	0.02	0.37	0.06	9.51E-01	1.00E+00
ENST00000506927	CLDND1	3.58	5.40	2.23	2.42	1.54E-02	8.73E-01	105.92	-0.39	0.48	-0.81	4.16E-01	1.00E+00
ENST00000375780	CLIC1	32.76	22.58	3.03	7.46	8.65E-14	8.07E-11	20.24	5.03	3.79	1.33	NA	NA
ENST00000298912	CLMN	1.00	3.56	1.72	2.07	3.88E-02	9.96E-01	219.74	1.45	0.53	2.76	5.86E-03	2.26E-01
ENST00000477264	CLSTN1	25.23	21.46	3.03	7.09	1.35E-12	5.02E-10	588.05	0.01	0.26	0.05	9.58E-01	1.00E+00
ENST00000310418	CLTB	5.55	4.83	2.21	2.18	2.92E-02	9.96E-01	1692.56	-0.03	0.57	-0.04	9.65E-01	1.00E+00
ENST00000256722	CMPK2	2.11	4.64	1.60	2.91	3.62E-03	3.34E-01	360.57	1.17	0.83	1.40	1.61E-01	9.75E-01
ENST00000568865	CNN2	7.64	6.49	2.48	2.62	8.84E-03	6.26E-01	1683.56	-0.09	0.38	-0.24	8.10E-01	1.00E+00
ENST00000549890	CNR1	36.62	0.91	0.37	2.45	1.42E-02	8.31E-01	77.73	0.84	0.43	1.94	5.20E-02	7.19E-01
ENST00000264638	CNTNAP1	10.88	1.54	0.54	2.84	4.49E-03	3.93E-01	353.00	0.28	0.82	0.34	7.36E-01	1.00E+00
ENST00000469061	CNTNAP3	5.50	1.59	0.73	2.19	2.89E-02	9.96E-01	1.56	1.44	1.48	0.97	3.32E-01	NA
ENST00000479351	CNTNAP3B	6.22	6.19	3.03	2.04	4.10E-02	9.96E-01	125.49	-0.95	1.62	-0.59	5.57E-01	1.00E+00
ENST00000393603	COG5	11.30	7.05	2.46	2.86	4.18E-03	3.71E-01	1435.67	-0.19	0.68	-0.28	7.78E-01	1.00E+00
ENST00000356340	COL13A1	1.89	3.85	1.99	1.93	5.30E-02	9.96E-01	2.56	-1.66	1.89	-0.88	3.81E-01	NA
ENST00000477429	COL4A5	2.49	4.87	2.28	2.13	3.29E-02	9.96E-01	15.60	-0.80	1.13	-0.71	4.78E-01	1.00E+00
ENST00000486023	COL6A1	4.43	5.10	2.61	1.95	5.11E-02	9.96E-01	46.53	0.32	0.45	0.70	4.81E-01	1.00E+00
ENST00000498614	COL6A1	61.29	23.47	3.03	7.76	8.83E-15	2.03E-11	26669.84	0.31	0.31	0.99	3.21E-01	1.00E+00
ENST00000273342	COL8A1	12.23	21.22	3.03	7.00	2.48E-12	7.15E-10	78.31	1.04	0.95	1.10	2.71E-01	1.00E+00
ENST00000349893	COPE	6.00	6.14	3.03	2.02	4.30E-02	9.96E-01	239.94	-0.61	0.55	-1.10	2.73E-01	1.00E+00
ENST00000538245	COPE	22.35	22.07	3.03	7.29	3.11E-13	1.90E-10	16200.80	-0.51	0.47	-1.09	2.74E-01	1.00E+00
ENST00000299259	COPS2	20.84	1.91	0.85	2.26	2.40E-02	9.96E-01	1289.74	-1.81	0.82	-2.22	2.64E-02	5.25E-01
ENST00000426712	COPS6	9.80	6.85	3.03	2.26	2.38E-02	9.96E-01	614.90	-0.48	0.47	-1.02	3.08E-01	1.00E+00
ENST00000549387	CORO1C	38.06	1.53	0.64	2.40	1.65E-02	9.05E-01	194.73	0.28	0.65	0.43	6.68E-01	1.00E+00
ENST00000567270	COX5A	7.50	6.46	3.03	2.13	3.31E-02	9.96E-01	1235.83	-0.17	0.59	-0.28	7.77E-01	1.00E+00
ENST00000247655	COX7C	5.60	4.85	1.88	2.58	9.98E-03	6.78E-01	11004.29	-0.28	0.42	-0.67	5.03E-01	1.00E+00
ENST00000512055	CPNE4	17.68	1.04	0.44	2.35	1.86E-02	9.66E-01	602.85	0.58	1.56	0.37	7.12E-01	1.00E+00

ENST00000349769	CPSF1	33.82	0.68	0.36	1.91	5.58E-02	9.96E-01	2566.80	-0.39	0.90	-0.44	6.60E-01	1.00E+00
ENST00000265394	CPVL	5.01	5.89	3.03	1.94	5.23E-02	9.96E-01	400.02	3.78	1.56	2.43	1.53E-02	3.94E-01
ENST00000409850	CPVL	9.63	3.32	1.19	2.79	5.30E-03	4.39E-01	31.67	-22.46	3.91	-5.75	NA	NA
ENST00000318080	CRAT	67.80	0.77	0.36	2.15	3.14E-02	9.96E-01	3196.75	1.34	0.74	1.82	6.94E-02	8.00E-01
ENST00000262367	CREBBP	16.11	0.94	0.47	2.00	4.50E-02	9.96E-01	661.28	0.75	2.07	0.36	NA	NA
ENST00000280527	CRIM1	260.96	0.74	0.20	3.79	1.50E-04	2.14E-02	8634.68	0.47	0.54	0.86	3.89E-01	1.00E+00
ENST00000546891	CS	8.37	6.63	2.53	2.62	8.72E-03	6.23E-01	32.54	0.82	0.51	1.61	1.08E-01	9.10E-01
ENST00000530886	CSDE1	5.70	6.07	2.33	2.61	9.07E-03	6.32E-01	2070.71	0.23	1.92	0.12	9.05E-01	1.00E+00
ENST00000329608	CSF1	56.85	0.89	0.30	2.97	2.97E-03	2.85E-01	6656.00	0.76	0.24	3.13	1.74E-03	1.05E-01
ENST00000303052	CSNK1G1	12.76	1.91	0.85	2.24	2.53E-02	9.96E-01	3629.51	0.37	1.42	0.26	7.92E-01	1.00E+00
ENST00000398411	CST3	15.94	21.60	3.03	7.13	9.83E-13	3.93E-10	1087.10	0.04	0.40	0.09	9.29E-01	1.00E+00
ENST00000431742	CSTF3	2.70	4.39	1.77	2.48	1.32E-02	7.90E-01	413.76	-0.27	0.54	-0.50	6.18E-01	1.00E+00
ENST00000563172	CTD-2354A18.1.1	0.95	3.48	1.75	1.99	4.68E-02	9.96E-01	669.71	1.45	0.44	3.31	9.46E-04	6.96E-02
ENST00000302763	CTNNA1	54.51	23.31	3.03	7.70	1.33E-14	2.62E-11	2207.03	-0.47	0.69	-0.68	4.98E-01	1.00E+00
ENST00000374595	CTNNAL1	37.06	1.45	0.58	2.50	1.24E-02	7.71E-01	3582.68	-0.33	0.76	-0.44	6.61E-01	1.00E+00
ENST00000450969	CTNNB1	8.73	6.68	3.03	2.20	2.75E-02	9.96E-01	57.14	0.43	0.91	0.47	6.37E-01	1.00E+00
ENST00000453024	CTNNB1	18.14	5.79	2.99	1.94	5.30E-02	9.96E-01	6152.35	-0.30	2.16	-0.14	8.89E-01	1.00E+00
ENST00000524630	CTNND1	18.67	21.82	3.03	7.21	5.75E-13	2.88E-10	111.22	1.04	2.02	0.52	6.05E-01	1.00E+00
ENST00000415599	CTSB	64.00	23.54	3.03	7.78	7.44E-15	1.82E-11	8.20	0.15	0.93	0.16	8.73E-01	1.00E+00
ENST00000340342	CTSL1	14.45	21.46	3.03	7.09	1.39E-12	5.02E-10	887.60	-0.01	0.50	-0.02	9.83E-01	1.00E+00
ENST00000375894	CTSL1	11.73	21.17	3.03	6.99	2.76E-12	7.78E-10	90.88	-0.59	0.49	-1.20	2.30E-01	1.00E+00
ENST00000375441	CUL4A	8.69	20.76	3.03	6.85	7.36E-12	1.66E-09	230.97	0.57	1.16	0.49	6.27E-01	1.00E+00
ENST00000440279	CUTA	8.43	6.04	2.15	2.81	4.97E-03	4.16E-01	142.43	-0.29	0.49	-0.59	5.55E-01	1.00E+00
ENST00000462802	CUTA	7.80	6.52	2.40	2.71	6.67E-03	5.27E-01	55.40	-1.26	1.00	-1.26	2.09E-01	1.00E+00
ENST00000565912	CX3CL1	9.52	1.58	0.50	3.18	1.47E-03	1.64E-01	132.00	0.30	1.08	0.28	7.82E-01	1.00E+00
ENST00000568342	CYB5B	41.21	2.26	0.74	3.07	2.14E-03	2.24E-01	129.72	0.54	1.09	0.49	6.23E-01	1.00E+00
ENST00000497655	CYB5R1	2.36	2.34	1.18	1.99	4.65E-02	9.96E-01	1105.78	0.06	0.60	0.10	9.18E-01	1.00E+00
ENST00000479935	DAG1	5.07	5.90	2.35	2.51	1.21E-02	7.61E-01	7.30	0.70	0.96	0.73	4.64E-01	1.00E+00
ENST00000491481	DARS	7.23	6.41	3.03	2.11	3.46E-02	9.96E-01	27.99	-0.05	0.73	-0.07	9.43E-01	1.00E+00
ENST00000460901	DBI	7.83	6.52	2.42	2.69	7.16E-03	5.46E-01	187.16	0.68	0.95	0.72	4.73E-01	1.00E+00
ENST00000475783	DBI	7.73	2.18	0.95	2.29	2.21E-02	9.96E-01	258.61	-0.92	0.59	-1.55	1.21E-01	9.33E-01
ENST00000309007	DBN1	13.04	6.84	3.03	2.26	2.39E-02	9.96E-01	5473.92	-1.05	1.03	-1.02	3.06E-01	1.00E+00
ENST00000491465	DCTN1	4.57	5.75	3.03	1.90	5.80E-02	9.96E-01	617.17	-0.43	0.50	-0.84	3.98E-01	1.00E+00
ENST00000550086	DCTN2	4.45	5.71	3.03	1.88	5.98E-02	9.96E-01	4.46	0.40	0.98	0.40	6.86E-01	1.00E+00
ENST00000450997	DDB1	8.33	20.70	3.03	6.83	8.46E-12	1.89E-09	10620.61	-0.08	0.31	-0.26	7.93E-01	1.00E+00
ENST00000535174	DDB1	7.19	20.50	3.03	6.76	1.36E-11	2.78E-09	22.96	0.39	0.66	0.59	5.58E-01	1.00E+00
ENST00000528148	DDHD2	4.27	1.50	0.76	1.98	4.79E-02	9.96E-01	1.63	1.21	1.61	0.75	4.52E-01	NA
ENST00000415136	DDOST	18.56	21.81	3.03	7.20	5.86E-13	2.90E-10	923.43	0.49	0.43	1.15	2.51E-01	1.00E+00
ENST00000486473	DDOST	6.47	6.26	2.39	2.61	8.97E-03	6.30E-01	36.39	-0.13	0.49	-0.27	7.89E-01	1.00E+00
ENST00000403230	DDX17	10.08	20.96	3.03	6.92	4.53E-12	1.10E-09	709.72	0.73	0.77	0.95	3.43E-01	1.00E+00
ENST00000444597	DDX17	5.69	6.07	3.03	2.00	4.55E-02	9.96E-01	33994.21	-0.04	1.35	-0.03	9.78E-01	1.00E+00
ENST00000376177	DDX39B	13.50	21.37	3.03	7.05	1.73E-12	5.63E-10	130.37	-1.26	0.64	-1.96	5.00E-02	7.05E-01
ENST00000481456	DDX39B	32.11	3.07	1.36	2.26	2.39E-02	9.96E-01	7070.34	0.37	0.32	1.17	2.43E-01	1.00E+00
ENST00000545038	DDX47	6.95	6.35	3.03	2.09	3.63E-02	9.96E-01	2941.43	-0.25	0.61	-0.41	6.79E-01	1.00E+00
ENST00000220764	DECR1	14.97	19.60	3.03	6.47	9.68E-11	1.81E-08	3193.09	0.47	0.47	1.01	3.14E-01	1.00E+00
ENST00000373624	DENND1A	15.32	0.92	0.46	2.01	4.47E-02	9.96E-01	1819.81	0.12	0.38	0.30	7.61E-01	1.00E+00
ENST00000532372	DKK3	203.30	1.03	0.50	2.05	4.09E-02	9.96E-01	23.94	-0.28	0.66	-0.43	6.65E-01	1.00E+00
ENST00000537636	DLAT	4.69	3.71	1.70	2.19	2.89E-02	9.96E-01	104.64	2.61	1.66	1.57	1.16E-01	9.27E-01
ENST00000205402	DLD	199.11	0.51	0.21	2.38	1.74E-02	9.33E-01	1726.08	-3.04	1.25	-2.43	NA	NA
ENST00000339266	DLGAP4	10.36	21.00	3.03	6.93	4.17E-12	1.04E-09	0.48	1.73	4.06	0.43	6.70E-01	NA
ENST00000555089	DLST	5.86	20.21	3.03	6.67	2.63E-11	5.09E-09	1219.12	1.36	2.25	0.61	NA	NA
ENST00000357606	DNAJC14	4.40	5.69	2.40	2.37	1.80E-02	9.49E-01	17.82	-5.16	1.62	-3.18	1.46E-03	9.33E-02
ENST00000276440	DOCK5	150.29	0.63	0.20	3.12	1.80E-03	1.94E-01	10129.36	0.81	1.46	0.55	5.82E-01	1.00E+00
ENST00000483783	DPP7	3.24	3.49	1.54	2.27	2.34E-02	9.96E-01	5448.29	0.17	0.58	0.29	7.71E-01	1.00E+00
ENST00000400454	DSCAM	2.55	2.23	0.96	2.33	2.00E-02	9.96E-01	459.72	1.18	0.51	2.29	2.22E-02	4.82E-01
ENST00000296161	DTX3L	81.28	0.72	0.29	2.47	1.34E-02	7.98E-01	4987.10	0.95	0.43	2.19	2.88E-02	5.52E-01
ENST00000240101	DUSP4	5.59	6.03	3.03	1.99	4.66E-02	9.96E-01	196.25	0.46	0.59	0.78	4.33E-01	1.00E+00
ENST00000308385	DUSP6	5.89	6.11	3.03	2.02	4.38E-02	9.96E-01	657.19	0.09	0.30	0.31	7.54E-01	1.00E+00

ENST00000547291	DUSP6	7.79	6.51	2.51	2.60	9.37E-03	6.46E-01	505.75	0.23	0.53	0.44	6.57E-01	1.00E+00
ENST00000563447	ECI1	5.64	6.05	3.03	2.00	4.60E-02	9.96E-01	456.12	0.07	0.32	0.22	8.24E-01	1.00E+00
ENST00000217182	EEF1A2	25.38	0.90	0.39	2.29	2.19E-02	9.96E-01	1725.73	0.62	0.54	1.15	2.51E-01	1.00E+00
ENST00000527741	EEF1D	9.20	6.76	3.03	2.23	2.58E-02	9.96E-01	1182.80	-0.60	0.35	-1.70	8.84E-02	8.63E-01
ENST00000462917	EIF1	152.13	2.47	0.79	3.12	1.81E-03	1.94E-01	1512.35	-0.17	0.28	-0.61	5.42E-01	1.00E+00
ENST00000293831	EIF4A1	158.06	3.37	1.75	1.93	5.42E-02	9.96E-01	71481.25	-0.46	0.35	-1.32	1.86E-01	9.94E-01
ENST00000496382	EIF4A2	9.37	6.79	3.03	2.24	2.51E-02	9.96E-01	9.32	0.48	0.93	0.52	6.04E-01	1.00E+00
ENST00000424196	EIF4G1	68.38	9.65	2.59	3.73	1.91E-04	2.68E-02	663.92	-0.62	0.94	-0.66	5.12E-01	1.00E+00
ENST00000526148	EIF4G2	6.43	6.24	3.03	2.06	3.95E-02	9.96E-01	10.39	-0.35	1.11	-0.32	7.51E-01	1.00E+00
ENST00000440766	EIF6	4.97	5.87	3.03	1.94	5.29E-02	9.96E-01	148.07	-0.32	0.75	-0.42	6.72E-01	1.00E+00
ENST00000541407	ELOVL5	14.88	21.51	3.03	7.10	1.24E-12	4.70E-10	254.16	0.83	1.06	0.78	4.33E-01	1.00E+00
ENST00000541477	ENO2	15.34	21.55	3.03	7.11	1.12E-12	4.39E-10	1310.52	0.94	0.93	1.01	3.11E-01	1.00E+00
ENST00000380935	ERBB2IP	9.22	6.76	2.36	2.87	4.15E-03	3.69E-01	561.26	0.42	0.64	0.65	5.15E-01	1.00E+00
ENST00000543086	ERC1	14.62	7.42	2.47	3.00	2.68E-03	2.67E-01	123.70	2.66	2.29	1.16	2.44E-01	1.00E+00
ENST00000013807	ERCC1	5.60	6.04	2.32	2.61	9.06E-03	6.32E-01	2150.16	-0.27	0.38	-0.72	4.73E-01	1.00E+00
ENST00000460485	ERCC3	1.16	3.77	1.88	2.01	4.46E-02	9.96E-01	90.48	-0.02	0.70	-0.02	9.81E-01	1.00E+00
ENST00000546477	ERP29	13.22	21.34	3.03	7.05	1.85E-12	5.87E-10	3663.24	-0.26	0.57	-0.46	6.46E-01	1.00E+00
ENST00000319349	ETV4	2.98	4.52	1.91	2.36	1.80E-02	9.50E-01	105.97	-0.65	0.82	-0.80	4.24E-01	1.00E+00
ENST00000544779	EXOSC10	42.59	1.16	0.45	2.56	1.04E-02	6.91E-01	2282.08	-0.48	0.89	-0.53	5.93E-01	1.00E+00
ENST00000490537	EXOSC8	4.59	5.76	3.03	1.90	5.76E-02	9.96E-01	1350.67	-0.21	0.31	-0.68	5.00E-01	1.00E+00
ENST00000528159	EXT2	5.47	6.01	3.03	1.98	4.75E-02	9.96E-01	29.85	0.34	0.47	0.73	4.67E-01	1.00E+00
ENST00000220562	EXTL3	23.89	0.68	0.33	2.04	4.09E-02	9.96E-01	7150.92	0.03	1.24	0.02	9.80E-01	1.00E+00
ENST00000517312	FADS2	5.97	6.14	3.03	2.02	4.30E-02	9.96E-01	4.54	-1.04	2.51	-0.41	6.79E-01	1.00E+00
ENST00000494293	FAM54B	1.66	2.98	1.37	2.17	2.98E-02	9.96E-01	378.16	0.44	0.31	1.44	1.50E-01	9.66E-01
ENST00000304916	FAM84B	4.36	2.54	0.84	3.02	2.56E-03	2.59E-01	3766.72	1.07	0.45	2.36	1.84E-02	4.37E-01
ENST00000279259	FAU	6.40	20.33	3.03	6.71	1.99E-11	3.91E-09	1508.50	-0.85	0.69	-1.24	2.16E-01	1.00E+00
ENST00000389087	FBN1	479.27	0.56	0.18	3.20	1.37E-03	1.56E-01	2357.09	-0.73	1.20	-0.60	5.46E-01	1.00E+00
ENST00000376770	FBXO44	1.74	4.34	1.78	2.44	1.45E-02	8.38E-01	166.17	1.00	0.48	2.08	3.78E-02	6.28E-01
ENST00000380339	FBXW9	1.90	4.47	1.76	2.55	1.08E-02	7.06E-01	460.32	-1.46	1.24	-1.18	2.39E-01	1.00E+00
ENST00000477057	FDPS	7.29	6.42	3.03	2.12	3.41E-02	9.96E-01	1237.10	-0.56	0.32	-1.77	7.59E-02	8.23E-01
ENST00000278919	FEZ1	10.46	1.48	0.75	1.99	4.68E-02	9.96E-01	1398.66	0.65	0.44	1.47	1.41E-01	9.56E-01
ENST00000310729	FGFR1	6.47	2.18	0.66	3.29	1.01E-03	1.19E-01	13.01	2.82	1.80	1.57	1.17E-01	9.28E-01
ENST00000464180	FKBP10	3.05	3.43	1.66	2.07	3.84E-02	9.96E-01	437.86	-0.25	0.31	-0.81	4.20E-01	1.00E+00
ENST00000466319	FLNA	49.07	1.90	0.98	1.93	5.31E-02	9.96E-01	170.76	-0.36	0.51	-0.70	4.83E-01	1.00E+00
ENST00000496542	FN1	7.72	2.33	0.85	2.74	6.17E-03	4.96E-01	20.97	-0.08	0.97	-0.08	9.36E-01	1.00E+00
ENST00000498719	FN1	6.72	5.71	2.03	2.81	4.94E-03	4.16E-01	450.26	0.33	1.34	0.24	NA	NA
ENST00000300784	FN3K	56.37	1.34	0.30	4.47	7.99E-06	1.30E-03	1124.82	0.16	0.60	0.27	7.87E-01	1.00E+00
ENST00000380874	FOXC1	21.98	0.88	0.44	2.01	4.40E-02	9.96E-01	1092.63	0.69	1.02	0.68	4.96E-01	1.00E+00
ENST00000317568	FRMD8	23.41	1.29	0.41	3.15	1.62E-03	1.79E-01	2132.84	0.16	0.47	0.35	7.26E-01	1.00E+00
ENST00000405801	FSCN1	4.64	5.76	3.03	1.90	5.75E-02	9.96E-01	27.49	0.87	0.57	1.54	1.23E-01	9.37E-01
ENST00000256759	FST	15.04	1.06	0.50	2.11	3.47E-02	9.96E-01	693.40	1.04	0.96	1.09	2.75E-01	1.00E+00
ENST00000445140	FXR1	10.32	6.33	2.36	2.68	7.39E-03	5.57E-01	2228.43	-1.01	0.83	-1.21	2.25E-01	1.00E+00
ENST00000522367	G3BP1	7.84	6.52	3.03	2.15	3.14E-02	9.96E-01	1.16	0.52	1.60	0.32	7.47E-01	NA
ENST00000393780	GAP43	7.53	6.46	3.03	2.13	3.30E-02	9.96E-01	668.09	0.48	0.91	0.53	5.98E-01	1.00E+00
ENST00000229239	GAPDH	228.10	11.10	3.03	3.67	2.46E-04	3.37E-02	72265.17	-0.55	0.31	-1.74	8.11E-02	8.42E-01
ENST00000396859	GAPDH	16.06	21.61	3.03	7.14	9.62E-13	3.88E-10	1507.86	-0.28	0.31	-0.90	3.70E-01	1.00E+00
ENST00000466588	GAPDH	19.68	5.76	2.98	1.93	5.34E-02	9.96E-01	50.66	0.47	0.65	0.73	4.68E-01	1.00E+00
ENST00000450766	GAS6	28.84	8.41	2.47	3.41	6.57E-04	8.14E-02	8661.20	1.49	1.04	1.43	1.52E-01	9.68E-01
ENST00000480426	GAS6	4.75	5.81	2.72	2.14	3.28E-02	9.96E-01	10155.78	1.23	1.00	1.23	2.19E-01	1.00E+00
ENST00000470952	GGT7	6.58	2.79	1.20	2.32	2.05E-02	9.96E-01	53.00	-0.56	0.65	-0.87	3.82E-01	1.00E+00
ENST00000223293	GIMAP2	1.67	3.64	1.44	2.53	1.15E-02	7.40E-01	306.46	1.00	0.39	2.61	9.14E-03	2.94E-01
ENST00000321612	GLDC	23.56	0.80	0.37	2.15	3.15E-02	9.96E-01	3182.01	0.62	0.62	1.00	3.16E-01	1.00E+00
ENST00000530075	GMDS	2.09	2.26	1.19	1.90	5.69E-02	9.96E-01	18.14	0.05	0.97	0.05	9.57E-01	1.00E+00
ENST00000078429	GNA11	30.92	1.03	0.50	2.05	4.01E-02	9.96E-01	3047.01	-0.10	0.49	-0.20	8.45E-01	1.00E+00
ENST00000490122	GNAI2	12.58	7.21	2.51	2.88	4.02E-03	3.60E-01	8547.97	-0.32	1.36	-0.23	8.17E-01	1.00E+00
ENST00000503494	GNB2L1	43.19	22.99	3.03	7.59	3.10E-14	4.26E-11	79.14	1.97	1.27	1.55	1.20E-01	9.32E-01
ENST00000506312	GNB2L1	6.48	6.25	3.03	2.06	3.91E-02	9.96E-01	298.76	-0.51	0.32	-1.58	1.14E-01	9.22E-01
ENST00000515417	GNB2L1	12.41	21.11	3.03	6.97	3.16E-12	8.70E-10	9282.55	2.53	0.97	2.61	9.05E-03	2.92E-01

ENST00000556752	GNG2	2.32	4.78	2.26	2.11	3.46E-02	9.96E-01	1261.41	4.61	1.54	2.99	2.82E-03	1.42E-01
ENST00000357743	GOLGA7	2.78	5.04	2.24	2.25	2.44E-02	9.96E-01	963.81	1.18	0.57	2.09	3.66E-02	6.18E-01
ENST00000543866	GOT1	32.40	5.00	1.49	3.35	8.21E-04	1.00E-01	35.54	0.80	0.52	1.54	1.24E-01	9.37E-01
ENST00000532758	GPAA1	7.47	20.54	3.03	6.78	1.22E-11	2.52E-09	85.57	-0.33	1.04	-0.32	7.49E-01	1.00E+00
ENST00000377047	GPC6	91.90	0.68	0.36	1.89	5.89E-02	9.96E-01	2650.35	0.06	0.61	0.10	9.22E-01	1.00E+00
ENST00000478722	GPHN	4.92	1.87	0.71	2.65	8.13E-03	5.96E-01	223.45	1.97	0.65	3.02	2.50E-03	1.30E-01
ENST00000515877	GPN1	7.79	20.61	3.03	6.80	1.05E-11	2.24E-09	70.76	4.09	1.39	2.94	3.30E-03	1.58E-01
ENST00000533662	GPR172A	6.50	4.33	1.62	2.68	7.33E-03	5.54E-01	121.90	-1.09	0.63	-1.73	8.34E-02	8.49E-01
ENST00000394705	GPR75	1.35	2.60	1.28	2.04	4.18E-02	9.96E-01	239.39	0.47	0.79	0.60	5.46E-01	1.00E+00
ENST00000407526	GRB10	13.77	21.40	3.03	7.06	1.62E-12	5.37E-10	52.10	0.15	0.82	0.18	8.57E-01	1.00E+00
ENST00000460882	GRHPR	15.25	7.49	3.03	2.47	1.34E-02	7.98E-01	1577.81	-0.17	0.34	-0.50	6.14E-01	1.00E+00
ENST00000327946	GRID1	6.93	2.14	0.74	2.88	3.99E-03	3.60E-01	1011.54	1.86	0.39	4.75	2.08E-06	5.37E-04
ENST00000529301	GRINA	8.12	20.66	3.03	6.82	9.28E-12	2.03E-09	4.63	0.94	1.17	0.80	4.22E-01	1.00E+00
ENST00000443571	GSTK1	4.53	5.74	3.03	1.89	5.86E-02	9.96E-01	270.94	0.83	0.58	1.42	1.54E-01	9.70E-01
ENST00000216044	GTPBP1	36.51	1.23	0.52	2.38	1.75E-02	9.33E-01	1246.56	-0.12	0.41	-0.30	7.66E-01	1.00E+00
ENST00000526355	GUCY1A2	3.20	1.81	0.87	2.07	3.85E-02	9.96E-01	190.69	2.77	0.84	3.29	9.86E-04	7.11E-02
ENST00000375249	HABP4	2.79	5.06	1.76	2.87	4.15E-03	3.69E-01	3865.38	-0.21	0.36	-0.59	5.57E-01	1.00E+00
ENST00000317799	HADHB	41.30	22.93	3.03	7.57	3.63E-14	4.55E-11	801.45	0.32	1.37	0.23	8.17E-01	1.00E+00
ENST00000537713	HADHB	19.56	21.89	3.03	7.23	4.88E-13	2.58E-10	233.11	0.90	1.76	0.51	6.10E-01	1.00E+00
ENST00000282058	HAUS1	2.50	4.87	2.33	2.09	3.69E-02	9.96E-01	2021.23	-0.08	0.37	-0.22	8.28E-01	1.00E+00
ENST00000229330	HCFC2	1.96	3.28	1.48	2.21	2.68E-02	9.96E-01	1112.19	0.29	0.79	0.37	7.14E-01	1.00E+00
ENST00000482974	HEMK1	1.36	3.99	1.84	2.17	3.03E-02	9.96E-01	247.49	-0.36	0.30	-1.19	2.34E-01	1.00E+00
ENST00000568777	HEXA	5.71	5.46	2.33	2.35	1.90E-02	9.78E-01	1039.09	-0.04	0.55	-0.08	9.38E-01	1.00E+00
ENST00000392939	HIST2H4A	32.75	3.23	1.18	2.74	6.14E-03	4.95E-01	326.23	0.45	0.26	1.73	8.38E-02	8.50E-01
ENST00000298649	HK1	34.36	22.67	3.03	7.49	6.92E-14	7.13E-11	3690.17	-1.24	3.73	-0.33	NA	NA
ENST00000359426	HK1	42.74	22.97	3.03	7.59	3.24E-14	4.26E-11	22231.12	0.36	0.71	0.51	6.13E-01	1.00E+00
ENST00000436817	HK1	8.39	19.43	3.03	6.41	1.44E-10	2.69E-08	4.01	-1.71	2.88	-0.60	5.51E-01	1.00E+00
ENST00000494253	HK1	5.88	6.11	3.03	2.02	4.38E-02	9.96E-01	315.55	-1.69	1.19	-1.42	1.57E-01	9.72E-01
ENST00000495183	HLA-A	6.07	6.16	3.03	2.03	4.23E-02	9.96E-01	15791.03	-0.30	0.65	-0.46	6.47E-01	1.00E+00
ENST00000484194	HLA-E	16.25	7.58	3.03	2.50	1.23E-02	7.70E-01	167.46	-0.21	0.38	-0.57	5.69E-01	1.00E+00
ENST00000483417	HMG20B	9.71	6.84	1.32	5.17	2.37E-07	4.17E-05	18.35	0.11	0.85	0.13	8.93E-01	1.00E+00
ENST00000495810	HNRNPA2B1	29.23	1.79	0.93	1.92	5.45E-02	9.96E-01	351.62	-0.41	0.23	-1.81	7.10E-02	8.06E-01
ENST00000352301	HNRNPD	4.24	5.65	2.25	2.51	1.20E-02	7.61E-01	4543.59	0.54	0.47	1.15	2.48E-01	1.00E+00
ENST00000376256	HNRNPK	8.81	20.77	3.03	6.86	7.11E-12	1.63E-09	1828.96	0.13	0.48	0.28	7.80E-01	1.00E+00
ENST00000374612	HNRNPR	7.77	2.84	1.08	2.63	8.46E-03	6.09E-01	286.57	-0.25	0.84	-0.30	7.68E-01	1.00E+00
ENST00000283179	HNRNPU	5.19	20.04	3.03	6.61	3.89E-11	7.35E-09	23108.88	-0.48	0.34	-1.42	1.54E-01	9.70E-01
ENST00000476241	HNRNPU	10.90	7.00	3.03	2.31	2.08E-02	9.96E-01	397.12	0.28	0.89	0.32	7.51E-01	1.00E+00
ENST00000484302	HOXB6	56.73	0.67	0.31	2.19	2.86E-02	9.96E-01	1548.59	-0.88	1.32	-0.67	5.04E-01	1.00E+00
ENST00000371554	HSP90AB1	69.60	23.59	3.03	7.79	6.46E-15	1.80E-11	208.83	0.52	0.42	1.25	2.10E-01	1.00E+00
ENST00000550595	HSP90B1	9.24	20.84	3.03	6.88	6.05E-12	1.40E-09	1314.10	-1.05	0.86	-1.22	2.21E-01	1.00E+00
ENST00000296464	HSPA4L	17.20	1.14	0.57	1.98	4.72E-02	9.96E-01	1970.38	0.56	0.50	1.12	2.64E-01	1.00E+00
ENST00000532091	HSPA8	3.51	3.08	1.51	2.04	4.10E-02	9.96E-01	300.09	-0.76	0.47	-1.61	1.06E-01	9.07E-01
ENST00000248553	HSPB1	87.08	0.90	0.40	2.25	2.47E-02	9.96E-01	16850.89	0.19	0.48	0.39	6.94E-01	1.00E+00
ENST00000448722	HSPB1P2	3.02	1.64	0.83	1.98	4.82E-02	9.96E-01	9.30	-0.05	0.84	-0.06	9.55E-01	1.00E+00
ENST00000503199	HSPD1P1	4.77	5.81	3.03	1.91	5.57E-02	9.96E-01	14.60	0.01	0.80	0.01	9.92E-01	1.00E+00
ENST00000489468	IAH1	1.87	3.78	1.66	2.28	2.25E-02	9.96E-01	99.12	0.44	0.36	1.22	2.23E-01	1.00E+00
ENST00000562225	IFI16	6.27	6.20	3.03	2.05	4.09E-02	9.96E-01	62.29	-0.08	0.53	-0.14	8.85E-01	1.00E+00
ENST00000415816	IFI35	2.51	4.90	2.34	2.09	3.62E-02	9.96E-01	831.10	-0.93	0.71	-1.31	1.90E-01	9.97E-01
ENST00000371936	IL13RA2	10.40	6.93	2.37	2.93	3.38E-03	3.16E-01	4.95	-1.36	3.78	-0.36	7.20E-01	1.00E+00
ENST00000318511	ILF3	7.78	20.58	3.03	6.79	1.11E-11	2.35E-09	104.84	-0.11	2.40	-0.05	NA	NA
ENST00000545283	IMMT	7.22	6.40	3.03	2.11	3.47E-02	9.96E-01	44.49	-0.22	0.43	-0.52	6.02E-01	1.00E+00
ENST00000338791	IMPDH1	2.66	3.73	1.57	2.37	1.76E-02	9.38E-01	7.14	-0.65	1.08	-0.60	5.49E-01	1.00E+00
ENST00000242208	INHBA	2.12	3.36	1.54	2.18	2.89E-02	9.96E-01	437.36	1.62	0.47	3.44	5.74E-04	4.88E-02
ENST00000506437	ITGA3	7.38	4.53	2.06	2.19	2.82E-02	9.96E-01	15100.47	-0.03	0.57	-0.06	9.55E-01	1.00E+00
ENST00000512553	ITGA3	11.04	4.28	2.04	2.10	3.55E-02	9.96E-01	23.44	-0.10	0.72	-0.14	8.89E-01	1.00E+00
ENST00000425522	ITGA4	8.60	6.62	3.03	2.19	2.88E-02	9.96E-01	0.77	1.27	3.92	0.32	7.47E-01	NA
ENST00000464001	ITGB1	14.75	1.37	0.73	1.89	5.92E-02	9.96E-01	21.14	-1.21	1.04	-1.16	2.45E-01	1.00E+00
ENST00000376180	ITGBL1	214.22	1.30	0.41	3.14	1.72E-03	1.86E-01	1646.46	0.86	1.44	0.59	5.53E-01	1.00E+00

ENST00000382303	KANK1	5.13	5.92	3.03	1.95	5.11E-02	9.96E-01	314.11	1.59	1.01	1.58	1.13E-01	9.20E-01
ENST00000534681	KAT5	13.55	7.32	1.68	4.35	1.36E-05	2.20E-03	14.49	1.09	1.72	0.63	5.26E-01	1.00E+00
ENST00000262888	KCNN4	17.21	3.24	1.03	3.15	1.63E-03	1.79E-01	3852.75	0.51	0.33	1.56	1.20E-01	9.32E-01
ENST00000322444	KCTD2	14.51	2.48	1.03	2.41	1.57E-02	8.85E-01	3846.34	-0.11	0.38	-0.30	7.61E-01	1.00E+00
ENST00000314256	KIAA0195	3.54	5.39	2.31	2.34	1.95E-02	9.94E-01	1622.36	0.61	1.81	0.34	7.37E-01	1.00E+00
ENST00000530275	KIAA0754	87.07	0.50	0.25	2.05	4.06E-02	9.96E-01	176.48	0.66	0.92	0.71	4.78E-01	1.00E+00
ENST00000377086	KIF1B	78.11	0.68	0.30	2.25	2.42E-02	9.96E-01	27.29	0.61	2.31	0.26	7.93E-01	1.00E+00
ENST00000508792	KIF20A	9.78	20.90	3.03	6.90	5.28E-12	1.26E-09	3174.23	0.15	2.93	0.05	9.60E-01	1.00E+00
ENST00000252999	LAMA5	129.52	1.68	0.35	4.76	1.96E-06	3.33E-04	8779.62	0.65	0.66	0.99	3.21E-01	1.00E+00
ENST00000514104	LARS	3.17	4.60	2.09	2.20	2.76E-02	9.96E-01	9.45	0.04	0.99	0.04	9.71E-01	1.00E+00
ENST00000545707	LDLR	4.51	3.44	0.98	3.53	4.14E-04	5.46E-02	54.33	0.73	1.61	0.46	6.49E-01	1.00E+00
ENST00000369308	LIX1L	10.25	1.59	0.79	2.02	4.31E-02	9.96E-01	3380.83	-0.07	0.27	-0.25	8.00E-01	1.00E+00
ENST00000347559	LMNA	22.41	22.07	3.03	7.29	3.09E-13	1.90E-10	2252.33	2.43	1.27	1.92	5.54E-02	7.35E-01
ENST00000361308	LMNA	44.73	22.98	3.03	7.59	3.18E-14	4.26E-11	11176.83	0.36	0.54	0.67	5.00E-01	1.00E+00
ENST00000395354	LMNB1	20.85	7.94	3.03	2.62	8.75E-03	6.23E-01	2104.53	-0.03	0.61	-0.05	9.59E-01	1.00E+00
ENST00000377499	LMO7	5.23	5.94	3.03	1.96	5.01E-02	9.96E-01	370.12	-0.75	1.19	-0.63	5.29E-01	1.00E+00
ENST00000374431	LPAR1	4.38	3.62	1.62	2.23	2.56E-02	9.96E-01	143.05	0.33	0.84	0.39	6.96E-01	1.00E+00
ENST00000543006	LPP	13.84	7.35	3.03	2.43	1.53E-02	8.66E-01	3582.25	1.01	1.15	0.88	3.80E-01	1.00E+00
ENST00000249377	LRRC17	19.17	3.42	1.42	2.41	1.60E-02	8.93E-01	787.53	-0.94	0.91	-1.03	3.04E-01	1.00E+00
ENST00000444156	LSM14B	17.75	1.19	0.50	2.40	1.64E-02	9.03E-01	2094.99	0.20	1.69	0.12	9.05E-01	1.00E+00
ENST00000464357	LSS	10.20	20.98	3.03	6.93	4.33E-12	1.07E-09	176.22	4.50	1.35	3.33	NA	NA
ENST00000533227	LUZP2	2.73	2.79	1.43	1.94	5.21E-02	9.96E-01	135.21	1.87	0.54	3.44	5.79E-04	4.91E-02
ENST00000415817	LZTR1	3.63	4.17	1.51	2.76	5.74E-03	4.68E-01	749.66	-0.39	0.66	-0.59	5.58E-01	1.00E+00
ENST00000379919	MAB21L1	65.70	0.74	0.26	2.84	4.50E-03	3.93E-01	2521.94	0.24	0.40	0.59	5.52E-01	1.00E+00
ENST00000545844	MACF1	4.77	5.81	3.03	1.91	5.57E-02	9.96E-01	0.00	NA	NA	NA	NA	NA
ENST00000375060	MAGED2	20.65	7.92	3.03	2.62	8.89E-03	6.27E-01	659.65	-0.47	0.61	-0.76	4.44E-01	1.00E+00
ENST00000463787	MAGED2	16.11	7.56	3.03	2.50	1.25E-02	7.73E-01	55.68	-0.15	0.61	-0.24	8.09E-01	1.00E+00
ENST00000416960	MAGED4	4.09	3.89	1.57	2.48	1.31E-02	7.89E-01	39.15	-0.47	1.31	-0.36	7.20E-01	1.00E+00
ENST00000348428	MALT1	63.81	1.71	0.32	5.34	9.25E-08	1.65E-05	2512.90	3.31	2.35	1.41	NA	NA
ENST00000545096	MAN1B1	3.33	5.30	2.28	2.33	1.99E-02	9.96E-01	23.87	0.61	0.90	0.68	4.97E-01	1.00E+00
ENST00000514430	MANBA	16.43	2.44	1.05	2.33	2.00E-02	9.96E-01	1841.17	-0.02	0.54	-0.04	9.72E-01	1.00E+00
ENST00000373505	MAPKAP1	22.22	1.08	0.44	2.45	1.43E-02	8.32E-01	61.56	1.39	1.81	0.77	4.41E-01	1.00E+00
ENST00000446044	MAPKAPK3	2.08	4.61	2.27	2.03	4.24E-02	9.96E-01	14.10	0.20	1.22	0.17	8.67E-01	1.00E+00
ENST00000361297	MAST2	4.76	1.79	0.90	2.00	4.55E-02	9.96E-01	756.41	-0.12	1.76	-0.07	9.45E-01	1.00E+00
ENST00000509990	MATR3	11.35	7.06	3.03	2.33	1.97E-02	9.96E-01	152.75	1.14	1.60	0.71	4.78E-01	1.00E+00
ENST00000449249	MBOAT7	2.49	4.88	2.28	2.14	3.23E-02	9.96E-01	267.56	0.86	0.84	1.02	3.06E-01	1.00E+00
ENST00000528533	MCAM	21.07	2.26	1.08	2.10	3.57E-02	9.96E-01	66.02	0.49	0.59	0.82	4.10E-01	1.00E+00
ENST00000377716	MCART1	1.72	2.34	1.19	1.96	4.98E-02	9.96E-01	123.23	-0.19	0.60	-0.32	7.47E-01	1.00E+00
ENST00000424167	MDH2	37.20	2.46	1.10	2.24	2.50E-02	9.96E-01	361.95	-0.24	0.45	-0.54	5.88E-01	1.00E+00
ENST00000492663	MDH2	12.31	1.74	0.78	2.24	2.49E-02	9.96E-01	61.41	0.04	0.49	0.07	9.41E-01	1.00E+00
ENST00000529626	MED17	10.55	0.94	0.50	1.89	5.91E-02	9.96E-01	89.19	0.41	0.64	0.64	5.22E-01	1.00E+00
ENST00000378586	MED4	4.72	5.79	2.40	2.41	1.60E-02	8.93E-01	534.82	0.24	0.43	0.55	5.80E-01	1.00E+00
ENST00000337652	MEN1	17.62	1.10	0.46	2.41	1.58E-02	8.85E-01	0.14	1.58	4.07	0.39	6.98E-01	NA
ENST00000378576	MEST	1.97	4.54	2.29	1.99	4.69E-02	9.96E-01	51.44	2.18	0.77	2.85	4.39E-03	1.88E-01
ENST00000341249	METTL23	2.28	4.76	2.33	2.04	4.11E-02	9.96E-01	878.97	0.04	0.28	0.15	8.81E-01	1.00E+00
ENST00000482661	MFN1	5.15	5.91	1.74	3.41	6.56E-04	8.14E-02	60.75	0.19	1.01	0.19	8.50E-01	1.00E+00
ENST00000329548	MFSD5	8.85	6.70	3.03	2.21	2.70E-02	9.96E-01	5170.94	-0.66	0.30	-2.16	3.05E-02	5.70E-01
ENST00000506797	MGST2	8.41	6.63	3.03	2.19	2.87E-02	9.96E-01	1595.59	-0.57	0.46	-1.24	2.15E-01	1.00E+00
ENST00000449934	MICA	6.41	6.24	3.03	2.06	3.96E-02	9.96E-01	4403.86	0.39	0.40	0.99	3.23E-01	1.00E+00
ENST00000390708	MIR708	3.27	1.96	0.86	2.28	2.25E-02	9.96E-01	0.59	0.94	2.06	0.46	6.46E-01	NA
ENST00000303319	MMADHC	8.12	20.67	3.03	6.82	9.18E-12	2.02E-09	709.73	0.02	0.31	0.05	9.59E-01	1.00E+00
ENST00000422154	MORF4L2	4.65	2.85	1.44	1.97	4.83E-02	9.96E-01	80.72	-1.79	1.72	-1.04	2.99E-01	1.00E+00
ENST00000313485	MPRIP	15.83	1.48	0.49	3.00	2.69E-03	2.67E-01	390.07	1.59	1.27	1.25	NA	NA
ENST00000468957	MRPL2	2.22	4.72	2.33	2.03	4.25E-02	9.96E-01	59.09	-0.22	0.56	-0.40	6.88E-01	1.00E+00
ENST00000482352	MRPL20	14.63	2.28	0.75	3.03	2.42E-03	2.46E-01	294.84	0.25	0.42	0.60	5.46E-01	1.00E+00
ENST00000514928	MRPL27	2.00	2.58	1.22	2.12	3.44E-02	9.96E-01	203.35	-0.29	0.33	-0.88	3.80E-01	1.00E+00
ENST00000355151	MRPL52	2.29	4.75	1.89	2.51	1.20E-02	7.61E-01	888.33	-1.01	0.42	-2.41	1.62E-02	4.06E-01
ENST00000477091	MRPS6	37.29	3.82	1.71	2.24	2.50E-02	9.96E-01	3423.97	-0.27	0.36	-0.74	4.58E-01	1.00E+00

ENST00000544857	MSH6	7.00	6.36	3.03	2.10	3.58E-02	9.96E-01	111.02	0.05	0.77	0.06	9.49E-01	1.00E+00
ENST00000525649	MTCH2	7.27	20.50	3.03	6.76	1.34E-11	2.75E-09	1222.43	-0.20	0.76	-0.26	7.94E-01	1.00E+00
ENST00000530428	MTCH2	18.90	20.35	3.03	6.72	1.81E-11	3.61E-09	448.07	0.13	0.47	0.28	7.81E-01	1.00E+00
ENST00000542981	MTCH2	6.24	6.20	3.03	2.04	4.09E-02	9.96E-01	71.53	0.27	0.62	0.43	6.64E-01	1.00E+00
ENST00000398600	MX1	25.60	1.15	0.37	3.15	1.61E-03	1.78E-01	86.18	1.34	1.57	0.85	3.95E-01	1.00E+00
ENST00000381556	MYBBP1A	7.53	20.55	3.03	6.78	1.20E-11	2.50E-09	206.05	-2.52	0.99	-2.55	1.06E-02	3.22E-01
ENST00000486218	MYH9	77.90	1.42	0.70	2.04	4.14E-02	9.96E-01	123.21	-0.28	0.44	-0.63	5.26E-01	1.00E+00
ENST00000550184	MYL6	23.12	2.15	0.88	2.45	1.42E-02	8.31E-01	11303.67	-0.09	0.24	-0.39	6.98E-01	1.00E+00
ENST00000553066	MYL6B	2.47	4.25	1.93	2.20	2.80E-02	9.96E-01	980.02	-1.38	0.90	-1.54	1.24E-01	9.38E-01
ENST00000318217	MYO1D	2.41	1.85	0.91	2.03	4.21E-02	9.96E-01	83.07	2.75	1.10	2.49	1.26E-02	3.55E-01
ENST00000285039	MYO5B	43.08	0.90	0.37	2.44	1.48E-02	8.48E-01	3063.14	1.00	1.93	0.52	6.04E-01	1.00E+00
ENST00000359263	MYOF	101.97	5.47	1.43	3.82	1.36E-04	1.96E-02	31409.69	-1.59	0.84	-1.89	5.87E-02	7.51E-01
ENST00000381804	N4BP2	9.86	6.86	2.36	2.90	3.73E-03	3.41E-01	22.74	3.44	1.81	1.90	5.71E-02	7.42E-01
ENST00000334982	NAA20	7.10	20.48	3.03	6.76	1.42E-11	2.85E-09	5579.07	-0.39	0.33	-1.18	2.39E-01	1.00E+00
ENST00000424702	NAA38	21.40	1.82	0.85	2.14	3.24E-02	9.96E-01	1362.52	-0.27	0.37	-0.73	4.67E-01	1.00E+00
ENST00000567743	NAE1	3.16	4.61	1.65	2.80	5.12E-03	4.27E-01	90.71	0.37	0.48	0.78	4.38E-01	1.00E+00
ENST00000483643	NAP1L4	3.59	4.17	1.92	2.16	3.04E-02	9.96E-01	35.58	0.88	0.45	1.94	5.25E-02	7.21E-01
ENST00000526115	NAP1L4	6.14	6.17	3.03	2.03	4.19E-02	9.96E-01	5262.78	-0.24	1.10	-0.22	8.29E-01	1.00E+00
ENST00000396085	NAV2	25.53	1.13	0.39	2.90	3.74E-03	3.41E-01	870.48	-0.76	1.65	-0.46	6.44E-01	1.00E+00
ENST00000397957	NCAM1	6.16	6.16	1.10	5.59	2.21E-08	4.04E-06	14.33	-3.22	0.95	-3.38	7.29E-04	5.75E-02
ENST00000374082	NCOA4	15.72	1.67	0.84	1.99	4.67E-02	9.96E-01	293.37	-0.55	1.49	-0.37	NA	NA
ENST00000373773	NDRG3	7.93	6.60	3.03	2.18	2.95E-02	9.96E-01	794.08	-1.94	1.31	-1.48	NA	NA
ENST00000538372	NDUFA12	11.14	2.15	0.96	2.24	2.48E-02	9.96E-01	14.49	-2.45	1.71	-1.43	1.52E-01	9.68E-01
ENST00000550187	NDUFA12	3.08	5.18	2.32	2.23	2.55E-02	9.96E-01	53.93	-2.30	1.24	-1.85	6.42E-02	7.80E-01
ENST00000471292	NDUFA3	6.99	1.64	0.81	2.03	4.23E-02	9.96E-01	171.41	0.15	0.46	0.32	7.47E-01	1.00E+00
ENST00000339600	NDUFA4	31.98	4.19	1.36	3.08	2.10E-03	2.21E-01	24359.10	-0.29	0.42	-0.69	4.92E-01	1.00E+00
ENST00000482299	NDUFA4	11.41	4.31	1.46	2.96	3.10E-03	2.95E-01	2887.57	-0.55	0.51	-1.08	2.82E-01	1.00E+00
ENST00000247866	NDUFB2	12.98	21.31	3.03	7.04	1.97E-12	6.02E-10	4222.08	-1.80	1.02	-1.76	7.77E-02	8.30E-01
ENST00000526169	NDUFV1	4.77	5.81	3.03	1.91	5.57E-02	9.96E-01	1738.46	0.42	0.43	0.97	3.33E-01	1.00E+00
ENST00000527355	NDUFV1	7.66	6.50	3.03	2.14	3.21E-02	9.96E-01	65.88	0.05	0.62	0.08	9.33E-01	1.00E+00
ENST00000318388	NDUFV2	7.91	6.54	3.03	2.16	3.11E-02	9.96E-01	8828.09	-0.30	0.45	-0.67	5.02E-01	1.00E+00
ENST00000375631	NEU1	5.73	6.07	3.03	2.00	4.54E-02	9.96E-01	813.42	-0.36	0.45	-0.81	4.15E-01	1.00E+00
ENST00000426654	NFAT5	22.77	0.70	0.34	2.06	3.96E-02	9.96E-01	78.97	-2.08	1.09	-1.92	5.52E-02	7.34E-01
ENST00000473371	NHLRC3	4.31	2.62	1.05	2.49	1.27E-02	7.79E-01	45.07	0.64	0.72	0.89	3.73E-01	1.00E+00
ENST00000324330	NIN	4.59	5.76	3.03	1.90	5.76E-02	9.96E-01	56.51	0.48	3.73	0.13	NA	NA
ENST00000567271	NME3	3.50	1.86	0.93	2.00	4.53E-02	9.96E-01	2.55	1.27	1.32	0.96	3.37E-01	NA
ENST00000490044	NONO	13.63	7.02	3.03	2.32	2.04E-02	9.96E-01	1006.20	-0.35	0.74	-0.48	6.34E-01	1.00E+00
ENST00000471702	NPHP3	21.78	1.93	0.95	2.04	4.13E-02	9.96E-01	154.27	-1.25	2.49	-0.50	6.17E-01	1.00E+00
ENST00000351986	NPM1	11.87	7.12	3.03	2.35	1.87E-02	9.68E-01	14464.08	-0.48	0.27	-1.76	7.79E-02	8.31E-01
ENST00000439109	NQO1	4.50	5.72	3.03	1.89	5.94E-02	9.96E-01	1190.56	-1.08	0.69	-1.56	1.20E-01	9.32E-01
ENST00000374822	NRP1	40.63	8.92	3.00	2.97	2.96E-03	2.85E-01	809.93	0.14	0.82	0.17	8.69E-01	1.00E+00
ENST00000355117	NRP2	14.34	6.81	1.72	3.95	7.77E-05	1.16E-02	86.58	-0.14	0.59	-0.24	8.14E-01	1.00E+00
ENST00000357118	NRP2	99.61	2.64	0.94	2.82	4.86E-03	4.14E-01	14053.36	-0.57	0.65	-0.87	3.83E-01	1.00E+00
ENST00000357785	NRP2	125.73	4.23	1.23	3.43	5.98E-04	7.55E-02	1332.66	-0.23	0.51	-0.44	6.57E-01	1.00E+00
ENST00000479431	NTM	9.12	6.74	3.03	2.22	2.61E-02	9.96E-01	2.45	-1.67	1.32	-1.26	2.08E-01	NA
ENST00000376214	NTRK2	10.04	20.96	3.03	6.92	4.59E-12	1.10E-09	64.95	-0.87	1.79	-0.48	6.29E-01	1.00E+00
ENST00000395882	NTRK2	11.17	5.87	2.42	2.42	1.54E-02	8.73E-01	81.69	1.41	1.98	0.71	NA	NA
ENST00000394480	NTRK3	7.37	2.35	0.75	3.15	1.65E-03	1.80E-01	156.74	1.97	0.62	3.18	1.47E-03	9.37E-02
ENST00000536544	NUMA1	8.15	20.67	3.03	6.82	9.04E-12	2.00E-09	7971.90	1.37	0.84	1.63	1.03E-01	8.99E-01
ENST00000496921	NUP214	1.12	3.72	1.74	2.13	3.28E-02	9.96E-01	217.93	0.14	0.31	0.46	6.46E-01	1.00E+00
ENST00000414849	NUSAP1	11.16	2.19	0.99	2.21	2.70E-02	9.96E-01	120.80	-0.03	0.45	-0.07	9.46E-01	1.00E+00
ENST00000514576	OCIAD2	17.04	3.01	1.32	2.28	2.24E-02	9.96E-01	7993.00	-0.21	0.27	-0.77	4.42E-01	1.00E+00
ENST00000449767	OGDH	2.80	5.03	2.39	2.10	3.53E-02	9.96E-01	287.03	0.68	0.86	0.79	4.29E-01	1.00E+00
ENST00000374103	OGDHL	72.12	2.81	1.00	2.82	4.86E-03	4.14E-01	1364.27	1.43	0.50	2.87	4.09E-03	1.80E-01
ENST00000370469	OGFR	2.59	4.93	2.26	2.18	2.95E-02	9.96E-01	0.00	NA	NA	NA	NA	NA
ENST00000378764	OPTN	6.07	6.16	2.27	2.71	6.71E-03	5.27E-01	42.67	-2.38	3.74	-0.64	NA	NA
ENST00000257966	OS9	19.03	7.80	3.03	2.58	9.96E-03	6.78E-01	893.45	-0.69	0.83	-0.84	4.03E-01	1.00E+00
ENST00000546916	OS9	14.64	2.43	0.95	2.55	1.09E-02	7.11E-01	3733.86	-0.73	0.53	-1.37	1.71E-01	9.83E-01

ENST00000543004	OTUB1	9.54	6.81	3.03	2.25	2.47E-02	9.96E-01	137.67	0.45	1.09	0.41	6.78E-01	1.00E+00
ENST00000440381	P4HA1	4.77	5.82	3.03	1.92	5.52E-02	9.96E-01	690.26	-1.25	1.68	-0.74	4.57E-01	1.00E+00
ENST00000467086	P4HB	33.41	22.62	3.03	7.47	7.86E-14	7.68E-11	108.78	0.37	0.77	0.49	6.26E-01	1.00E+00
ENST00000473021	P4HB	15.08	4.96	1.53	3.25	1.16E-03	1.34E-01	6541.36	0.71	1.29	0.56	5.78E-01	1.00E+00
ENST00000484115	P4HTM	8.20	1.37	0.65	2.11	3.48E-02	9.96E-01	1505.28	0.14	0.37	0.38	7.03E-01	1.00E+00
ENST00000517921	PABPC1	11.98	5.79	3.02	1.91	5.57E-02	9.96E-01	45.21	0.32	0.51	0.63	5.28E-01	1.00E+00
ENST00000522387	PABPC1	7.91	20.63	3.03	6.81	9.97E-12	2.16E-09	12160.94	-0.48	0.44	-1.09	2.75E-01	1.00E+00
ENST00000527224	PACS1	6.73	6.27	3.03	2.07	3.87E-02	9.96E-01	398.39	0.14	0.74	0.19	8.48E-01	1.00E+00
ENST00000551359	PAN2	10.36	2.98	1.20	2.49	1.29E-02	7.85E-01	61.07	0.30	2.21	0.14	8.91E-01	1.00E+00
ENST00000544292	PARD3	3.76	3.33	1.75	1.90	5.68E-02	9.96E-01	70.12	-0.33	0.77	-0.43	6.66E-01	1.00E+00
ENST00000338639	PARK7	17.45	21.73	3.03	7.17	7.25E-13	3.33E-10	4987.89	-0.64	0.45	-1.41	1.60E-01	9.74E-01
ENST00000437198	PARN	6.79	6.32	1.61	3.92	8.90E-05	1.32E-02	3183.66	-0.08	0.31	-0.27	7.84E-01	1.00E+00
ENST00000417220	PARP3	1.92	4.51	2.31	1.95	5.11E-02	9.96E-01	1161.51	-0.86	1.94	-0.44	6.56E-01	1.00E+00
ENST00000489652	PARP9	7.52	6.47	3.03	2.13	3.28E-02	9.96E-01	162.40	0.35	0.43	0.81	4.15E-01	1.00E+00
ENST00000468777	PCCB	6.58	20.37	3.03	6.72	1.82E-11	3.61E-09	52.72	0.55	2.54	0.22	8.30E-01	1.00E+00
ENST00000264360	PCDH10	17.92	0.97	0.39	2.47	1.34E-02	7.98E-01	1803.79	0.65	0.46	1.43	1.52E-01	9.68E-01
ENST00000484979	PCDH17	29.43	0.89	0.36	2.48	1.30E-02	7.89E-01	167.14	-0.48	0.75	-0.64	5.22E-01	1.00E+00
ENST00000361762	PCDH7	9.10	1.09	0.50	2.19	2.89E-02	9.96E-01	528.20	1.24	0.59	2.09	3.62E-02	6.14E-01
ENST00000239446	PCDHB10	6.34	1.28	0.67	1.92	5.47E-02	9.96E-01	881.89	0.77	0.36	2.15	3.16E-02	5.80E-01
ENST00000194155	PCDHB2	13.19	0.85	0.44	1.92	5.50E-02	9.96E-01	1000.35	0.58	0.32	1.82	6.90E-02	7.97E-01
ENST00000522605	PCDHGB2	22.49	0.65	0.34	1.92	5.48E-02	9.96E-01	884.28	0.89	0.45	1.98	4.75E-02	6.89E-01
ENST00000337344	PCID2	5.56	6.03	3.03	1.99	4.67E-02	9.96E-01	505.07	6.64	1.95	3.40	NA	NA
ENST00000379160	PCNA	192.68	11.15	2.01	5.54	3.05E-08	5.53E-06	2333.28	-1.51	0.70	-2.14	3.25E-02	5.85E-01
ENST00000218230	PCSK1N	4.11	5.61	1.18	4.75	2.00E-06	3.38E-04	768.04	1.90	0.54	3.52	4.31E-04	3.97E-02
ENST00000497056	PDCD10	9.68	6.83	3.03	2.25	2.42E-02	9.96E-01	307.24	-0.06	0.46	-0.13	8.99E-01	1.00E+00
ENST00000462577	PDCD4	7.75	20.58	3.03	6.79	1.13E-11	2.37E-09	8.72	0.66	1.01	0.65	5.18E-01	1.00E+00
ENST00000359062	PDE3A	12.58	1.71	0.49	3.51	4.55E-04	5.89E-02	1062.66	1.51	0.54	2.78	5.52E-03	2.17E-01
ENST00000288050	PDPR	4.09	2.12	1.11	1.91	5.57E-02	9.96E-01	1676.43	-0.94	2.34	-0.40	6.87E-01	1.00E+00
ENST00000438447	PDZD2	5.40	1.43	0.70	2.05	4.02E-02	9.96E-01	53.04	-8.41	3.18	-2.64	NA	NA
ENST00000550964	PFDN5	4.25	2.98	1.25	2.39	1.70E-02	9.21E-01	1.16	0.52	1.60	0.32	7.47E-01	NA
ENST00000421751	PFKP	5.86	20.21	3.03	6.67	2.63E-11	5.09E-09	189.50	0.90	0.56	1.61	1.08E-01	9.10E-01
ENST00000497148	PFN2	7.54	6.47	3.03	2.13	3.28E-02	9.96E-01	4.93	0.24	0.87	0.28	7.80E-01	1.00E+00
ENST00000359246	PHF2	10.56	0.92	0.49	1.89	5.84E-02	9.96E-01	3746.03	-0.74	0.90	-0.82	4.12E-01	1.00E+00
ENST00000299167	PHKB	4.88	1.37	0.72	1.90	5.70E-02	9.96E-01	413.10	-0.92	1.70	-0.54	5.86E-01	1.00E+00
ENST00000568171	PHKB	2.10	4.62	2.33	1.98	4.73E-02	9.96E-01	127.99	0.80	0.68	1.17	2.43E-01	1.00E+00
ENST00000400316	PHLPP1	16.91	2.09	0.74	2.83	4.67E-03	4.04E-01	43.24	0.17	0.84	0.20	8.40E-01	1.00E+00
ENST00000393045	PIAS3	6.57	1.77	0.89	1.99	4.70E-02	9.96E-01	196.21	1.10	1.69	0.65	5.14E-01	1.00E+00
ENST00000333590	PIGA	3.19	2.85	1.37	2.08	3.78E-02	9.96E-01	460.77	-0.27	0.92	-0.29	7.68E-01	1.00E+00
ENST00000452740	PIGU	5.87	4.49	2.09	2.15	3.17E-02	9.96E-01	249.82	-1.63	1.40	-1.17	2.44E-01	1.00E+00
ENST00000482866	PION	2.11	4.60	1.44	3.20	1.38E-03	1.56E-01	1344.94	-0.13	0.59	-0.22	8.25E-01	1.00E+00
ENST00000460723	PISD	2.30	4.76	2.51	1.90	5.77E-02	9.96E-01	12.07	0.80	0.69	1.16	2.48E-01	1.00E+00
ENST00000539476	PITPNA	6.91	6.34	3.03	2.09	3.65E-02	9.96E-01	82.65	2.45	1.73	1.42	NA	NA
ENST00000566809	PKM2	8.88	6.76	3.03	2.23	2.57E-02	9.96E-01	102.20	0.23	0.62	0.37	7.14E-01	1.00E+00
ENST00000568743	PKM2	201.51	3.25	0.98	3.32	8.88E-04	1.07E-01	15022.59	-0.91	1.39	-0.66	5.12E-01	1.00E+00
ENST00000569857	PKM2	510.64	4.85	1.79	2.71	6.69E-03	5.27E-01	6226.97	-0.50	0.55	-0.91	3.64E-01	1.00E+00
ENST00000283243	PLA2R1	3.46	4.10	1.23	3.33	8.59E-04	1.04E-01	1589.84	0.48	0.98	0.49	6.26E-01	1.00E+00
ENST00000270189	PLAT	11.60	7.09	3.03	2.34	1.92E-02	9.86E-01	245.17	-1.36	1.53	-0.88	3.76E-01	1.00E+00
ENST00000540511	PLCD3	9.23	2.33	0.69	3.35	7.98E-04	9.78E-02	6685.69	0.32	0.27	1.18	2.38E-01	1.00E+00
ENST00000465571	PLCG1	1.52	4.20	1.75	2.40	1.65E-02	9.04E-01	1.16	0.52	1.60	0.32	7.47E-01	NA
ENST00000538982	PLEKHB2	8.69	20.76	3.03	6.85	7.36E-12	1.66E-09	60.65	-1.09	2.23	-0.49	6.25E-01	1.00E+00
ENST00000358517	PLEKHG1	5.86	6.10	3.03	2.01	4.42E-02	9.96E-01	6077.05	0.06	0.75	0.08	9.34E-01	1.00E+00
ENST00000543070	PLS3	58.40	21.99	3.03	7.27	3.73E-13	2.21E-10	118.56	0.01	1.15	0.01	9.92E-01	1.00E+00
ENST00000449103	PLXNB2	131.75	0.57	0.29	1.95	5.08E-02	9.96E-01	4625.66	1.19	0.59	2.02	4.39E-02	6.67E-01
ENST00000563500	PML	1.87	3.82	1.28	2.99	2.81E-03	2.75E-01	323.81	-0.03	0.35	-0.09	9.32E-01	1.00E+00
ENST00000456433	PMPCB	5.11	5.91	3.03	1.95	5.12E-02	9.96E-01	88.48	-0.11	0.47	-0.24	8.11E-01	1.00E+00
ENST00000498530	PMPCB	15.72	21.58	3.03	7.13	1.03E-12	4.07E-10	257.91	-0.47	0.90	-0.52	6.04E-01	1.00E+00
ENST00000525250	PNPLA2	2.17	4.07	1.91	2.13	3.29E-02	9.96E-01	466.65	-0.01	0.44	-0.02	9.86E-01	1.00E+00
ENST00000461589	POLR3F	2.05	2.79	1.25	2.23	2.56E-02	9.96E-01	23.37	0.24	0.92	0.26	7.93E-01	1.00E+00

ENST00000460403	POMP	17.31	7.67	3.03	2.53	1.13E-02	7.34E-01	282.11	-0.34	0.74	-0.46	6.47E-01	1.00E+00
ENST00000264867	PPARGC1A	29.92	0.98	0.46	2.12	3.40E-02	9.96E-01	4041.46	0.68	0.29	2.34	1.92E-02	4.46E-01
ENST00000395580	PPHLN1	6.47	6.25	2.45	2.55	1.08E-02	7.06E-01	1017.11	-0.37	1.92	-0.19	NA	NA
ENST00000561048	PPIB	22.80	2.68	1.02	2.63	8.46E-03	6.09E-01	441.63	-0.02	0.43	-0.05	9.64E-01	1.00E+00
ENST00000532279	PPP1CA	3.14	5.21	1.89	2.76	5.83E-03	4.73E-01	163.28	-1.42	1.27	-1.12	2.65E-01	1.00E+00
ENST00000422370	PPP1R37	2.57	4.90	1.97	2.49	1.27E-02	7.79E-01	309.00	-0.18	0.40	-0.45	6.54E-01	1.00E+00
ENST00000423280	PPP1R7	4.10	4.37	2.30	1.90	5.73E-02	9.96E-01	424.21	-0.47	1.97	-0.24	NA	NA
ENST00000394413	PPP2R2B	13.77	1.10	0.51	2.17	2.98E-02	9.96E-01	488.40	0.98	1.04	0.94	3.46E-01	1.00E+00
ENST00000563732	PPP4C	4.99	5.87	3.03	1.94	5.29E-02	9.96E-01	71.86	-0.05	0.99	-0.05	9.62E-01	1.00E+00
ENST00000373547	PPP6C	11.47	3.63	1.65	2.20	2.78E-02	9.96E-01	3551.42	0.10	0.47	0.21	8.34E-01	1.00E+00
ENST00000262746	PRDX1	52.70	6.99	1.91	3.65	2.63E-04	3.58E-02	45.23	-2.40	0.94	-2.54	1.12E-02	3.30E-01
ENST00000372079	PRDX1	4.47	5.72	3.03	1.89	5.93E-02	9.96E-01	34.90	0.50	0.69	0.73	4.65E-01	1.00E+00
ENST00000334482	PRDX2	6.97	5.76	2.05	2.81	4.94E-03	4.16E-01	322.47	-0.49	0.50	-0.98	3.29E-01	1.00E+00
ENST00000550448	PRKAG1	5.10	5.90	3.03	1.95	5.16E-02	9.96E-01	63.60	0.40	0.49	0.82	4.14E-01	1.00E+00
ENST00000392711	PRKAR1A	16.36	21.64	3.03	7.14	9.04E-13	3.80E-10	324.72	-0.76	1.62	-0.47	6.39E-01	1.00E+00
ENST00000265563	PRKAR2A	16.17	2.07	0.82	2.51	1.21E-02	7.61E-01	3211.51	-0.29	0.31	-0.92	3.55E-01	1.00E+00
ENST00000466404	PRPF31	2.70	3.73	1.63	2.29	2.18E-02	9.96E-01	175.72	1.88	0.76	2.47	1.37E-02	3.70E-01
ENST00000376033	PRRC2A	7.23	6.41	3.03	2.11	3.46E-02	9.96E-01	5701.01	2.76	0.99	2.78	5.46E-03	2.16E-01
ENST00000428286	PRUNE2	2.69	3.76	1.25	3.01	2.65E-03	2.65E-01	36.05	2.46	1.69	1.46	1.45E-01	9.61E-01
ENST00000394934	PSAP	111.24	6.90	2.92	2.36	1.82E-02	9.55E-01	90746.35	-0.15	0.32	-0.46	6.48E-01	1.00E+00
ENST00000531156	PSMA1	5.69	6.06	3.03	2.00	4.58E-02	9.96E-01	57.00	-0.35	0.50	-0.71	4.78E-01	1.00E+00
ENST00000554812	PSMA3	6.12	6.17	3.03	2.03	4.19E-02	9.96E-01	1170.48	0.32	0.60	0.53	5.97E-01	1.00E+00
ENST00000531653	PSMC3	7.39	5.24	2.75	1.90	5.71E-02	9.96E-01	78.32	-0.19	0.33	-0.57	5.67E-01	1.00E+00
ENST00000526783	PSMD13	8.38	20.71	3.03	6.83	8.29E-12	1.86E-09	688.97	-0.75	0.48	-1.55	1.21E-01	9.33E-01
ENST00000476461	PSMD2	6.05	2.32	1.04	2.22	2.61E-02	9.96E-01	312.15	-0.20	0.70	-0.28	7.79E-01	1.00E+00
ENST00000561059	PSME1	8.62	4.41	2.31	1.91	5.65E-02	9.96E-01	329.53	0.16	0.37	0.43	6.66E-01	1.00E+00
ENST00000409746	PSTPIP2	4.57	1.39	0.64	2.17	3.04E-02	9.96E-01	701.40	1.78	0.31	5.68	1.35E-08	6.80E-06
ENST00000377200	PTAR1	9.48	2.30	1.00	2.31	2.10E-02	9.96E-01	6338.96	0.46	0.63	0.73	4.67E-01	1.00E+00
ENST00000236228	PTBP2	5.15	3.01	1.20	2.50	1.25E-02	7.73E-01	106.61	-1.37	1.49	-0.92	3.57E-01	1.00E+00
ENST00000393083	PTN	8.55	5.73	3.03	1.89	5.87E-02	9.96E-01	4219.44	-0.19	0.27	-0.70	4.85E-01	1.00E+00
ENST00000371621	PTPN1	93.11	0.81	0.23	3.58	3.40E-04	4.54E-02	3954.80	1.36	1.04	1.30	1.93E-01	9.99E-01
ENST00000568108	PTPN9	3.11	5.20	2.29	2.27	2.32E-02	9.96E-01	11.94	0.57	0.82	0.69	4.87E-01	1.00E+00
ENST00000355005	PTPRS	3.73	5.46	2.23	2.45	1.44E-02	8.34E-01	6.80	0.09	0.77	0.12	9.05E-01	1.00E+00
ENST00000511609	QDPR	2.35	2.23	1.10	2.02	4.37E-02	9.96E-01	7.01	0.62	0.96	0.65	5.17E-01	1.00E+00
ENST00000498392	QRICH1	2.47	3.60	1.87	1.92	5.43E-02	9.96E-01	86.96	-0.25	0.72	-0.34	7.33E-01	1.00E+00
ENST00000415040	RAB34	4.64	5.78	3.03	1.90	5.70E-02	9.96E-01	1606.86	-0.64	0.58	-1.12	2.64E-01	1.00E+00
ENST00000485090	RABGAP1	8.59	6.66	3.03	2.20	2.80E-02	9.96E-01	3.60	1.01	1.20	0.84	4.00E-01	1.00E+00
ENST00000552310	RACGAP1	1.57	4.23	2.24	1.89	5.93E-02	9.96E-01	9.18	0.35	1.76	0.20	8.40E-01	1.00E+00
ENST00000435265	RANBP1	7.52	4.81	2.01	2.39	1.66E-02	9.09E-01	700.83	-0.84	0.41	-2.04	4.11E-02	6.50E-01
ENST00000503028	RBM14-RBM4	4.93	5.86	3.03	1.93	5.33E-02	9.96E-01	265.47	0.77	0.52	1.49	1.37E-01	9.50E-01
ENST00000533898	RCN1	7.61	6.48	2.40	2.70	6.90E-03	5.34E-01	954.24	-1.03	0.55	-1.87	6.09E-02	7.65E-01
ENST00000428130	RDH11	6.77	5.72	2.76	2.07	3.86E-02	9.96E-01	6.24	-1.25	1.02	-1.23	2.20E-01	1.00E+00
ENST00000553578	RDH11	23.91	8.13	2.71	3.00	2.66E-03	2.66E-01	1439.60	1.08	0.95	1.13	2.58E-01	1.00E+00
ENST00000554731	RDH11	23.56	8.11	3.03	2.68	7.36E-03	5.56E-01	15.07	-0.10	0.65	-0.16	8.75E-01	1.00E+00
ENST00000557331	RDH11	9.63	6.82	3.03	2.25	2.43E-02	9.96E-01	11.73	-0.21	1.08	-0.20	8.44E-01	1.00E+00
ENST00000540492	REXO2	7.56	1.40	0.65	2.17	3.03E-02	9.96E-01	406.65	0.16	0.31	0.54	5.92E-01	1.00E+00
ENST00000304685	RGL1	15.66	1.64	0.78	2.09	3.64E-02	9.96E-01	173.97	1.85	1.36	1.36	1.74E-01	9.85E-01
ENST00000473074	RHOC	10.12	6.89	3.03	2.28	2.29E-02	9.96E-01	1629.06	-0.95	0.70	-1.37	1.72E-01	9.84E-01
ENST00000544458	RHPN2	2.43	4.84	2.29	2.11	3.44E-02	9.96E-01	219.24	0.32	1.97	0.16	8.71E-01	1.00E+00
ENST00000368323	RIT1	2.31	4.76	2.40	1.98	4.75E-02	9.96E-01	2791.63	0.19	0.24	0.82	4.11E-01	1.00E+00
ENST00000552176	RNASEK	12.05	1.59	0.76	2.10	3.59E-02	9.96E-01	580.87	-0.52	0.45	-1.14	2.53E-01	1.00E+00
ENST00000389100	RNF180	0.90	3.42	1.73	1.98	4.78E-02	9.96E-01	65.26	2.53	1.02	2.49	1.26E-02	3.55E-01
ENST00000311413	RNF26	32.23	1.12	0.54	2.08	3.76E-02	9.96E-01	6884.85	-0.45	0.31	-1.47	1.42E-01	9.56E-01
ENST00000354420	RNH1	8.89	4.35	1.55	2.81	4.89E-03	4.15E-01	74.32	0.97	1.22	0.80	4.24E-01	1.00E+00
ENST00000524780	RNH1	15.50	2.74	1.36	2.01	4.42E-02	9.96E-01	27.52	0.10	1.25	0.08	9.36E-01	1.00E+00
ENST00000563030	ROGDI	2.85	4.44	2.01	2.21	2.72E-02	9.96E-01	1546.74	-0.30	0.35	-0.85	3.95E-01	1.00E+00
ENST00000520819	RP11-11N9.4.1	10.91	1.62	0.69	2.36	1.84E-02	9.62E-01	2.94	0.20	1.52	0.13	8.97E-01	1.00E+00
ENST00000463721	RP11-155D18.11.1	5.71	6.08	2.27	2.68	7.42E-03	5.58E-01	101.71	-0.06	0.77	-0.07	9.42E-01	1.00E+00

ENST00000555399	RP11-566K11.2.1	12.68	21.28	3.03	7.02	2.15E-12	6.41E-10	90.10	-0.80	1.50	-0.54	5.91E-01	1.00E+00
ENST00000547717	RP11-793H13.8.1	5.04	4.70	2.07	2.27	2.30E-02	9.96E-01	4221.59	-0.32	0.36	-0.89	3.74E-01	1.00E+00
ENST00000322203	RPL10A	8.84	6.70	2.56	2.61	9.01E-03	6.31E-01	5169.34	-0.85	0.49	-1.72	8.60E-02	8.56E-01
ENST00000482370	RPL11	6.07	6.16	3.03	2.03	4.23E-02	9.96E-01	5622.45	-0.78	0.66	-1.18	2.39E-01	1.00E+00
ENST00000344063	RPL28	33.64	22.64	3.03	7.48	7.49E-14	7.51E-11	15004.86	-0.76	0.64	-1.19	2.36E-01	1.00E+00
ENST00000560881	RPL28	7.61	6.48	3.03	2.14	3.25E-02	9.96E-01	7995.96	-0.35	0.34	-1.03	3.03E-01	1.00E+00
ENST00000471290	RPL3	5.10	3.37	1.45	2.32	2.05E-02	9.96E-01	359.25	-0.80	0.51	-1.58	1.15E-01	9.23E-01
ENST00000474640	RPL35A	15.03	1.92	0.86	2.23	2.58E-02	9.96E-01	386.84	-0.66	0.56	-1.18	2.37E-01	1.00E+00
ENST00000311111	RPL38	14.42	4.61	1.93	2.39	1.68E-02	9.16E-01	14962.48	-0.72	0.52	-1.39	1.64E-01	9.77E-01
ENST00000552461	RPLP0	5.99	2.55	1.00	2.55	1.08E-02	7.06E-01	893.47	-0.42	0.37	-1.11	2.65E-01	1.00E+00
ENST00000524867	RPLP2	19.00	2.38	0.95	2.50	1.23E-02	7.70E-01	168.62	-0.54	0.48	-1.12	2.62E-01	1.00E+00
ENST00000343986	RPS21	14.97	21.51	3.03	7.10	1.22E-12	4.67E-10	33362.38	-0.32	0.49	-0.65	5.14E-01	1.00E+00
ENST00000470475	RPS8	6.60	6.28	3.03	2.07	3.83E-02	9.96E-01	1525.90	-0.45	0.45	-0.99	3.24E-01	1.00E+00
ENST00000369415	RRAGD	3.27	5.27	1.69	3.11	1.86E-03	1.99E-01	4203.28	0.38	0.57	0.67	5.00E-01	1.00E+00
ENST00000474701	RRM2	31.26	20.54	3.03	6.79	1.16E-11	2.43E-09	609.16	-0.55	0.82	-0.67	5.02E-01	1.00E+00
ENST00000267484	RTN1	10.24	1.96	0.66	2.97	3.02E-03	2.89E-01	887.50	1.71	0.62	2.75	6.01E-03	2.29E-01
ENST00000357732	RTN4	11.73	7.11	3.03	2.35	1.90E-02	9.78E-01	1413.75	0.72	0.49	1.47	1.43E-01	9.57E-01
ENST00000394609	RTN4	15.81	7.54	3.03	2.49	1.28E-02	7.83E-01	19861.46	1.78	1.31	1.36	NA	NA
ENST00000417360	RUVBL1	5.43	20.11	3.03	6.63	3.37E-11	6.43E-09	3262.55	-0.61	1.90	-0.32	7.47E-01	1.00E+00
ENST00000552207	SARNP	12.25	7.17	3.03	2.37	1.80E-02	9.49E-01	211.44	-0.20	0.27	-0.75	4.54E-01	1.00E+00
ENST00000477544	SARS	10.07	6.89	3.03	2.27	2.31E-02	9.96E-01	63.75	-0.80	1.29	-0.62	5.39E-01	1.00E+00
ENST00000490953	SBDS	2.93	4.47	1.69	2.65	8.12E-03	5.96E-01	60.82	0.08	0.95	0.08	9.33E-01	1.00E+00
ENST00000268099	SCAMP2	5.40	5.99	3.03	1.98	4.82E-02	9.96E-01	848.03	-0.84	0.86	-0.97	3.31E-01	1.00E+00
ENST00000405555	SCARF2	3.33	2.09	0.91	2.30	2.13E-02	9.96E-01	264.41	7.11	2.28	3.11	NA	NA
ENST00000459255	SCARNA10	2632.84	0.53	0.23	2.29	2.22E-02	9.96E-01	2.12	1.76	1.94	0.91	3.63E-01	NA
ENST00000458748	SCARNA2	1052.05	1.03	0.50	2.05	4.02E-02	9.96E-01	7.89	1.64	1.56	1.05	2.91E-01	1.00E+00
ENST00000319540	SCD5	130.17	1.02	0.22	4.71	2.44E-06	4.10E-04	11692.83	0.74	0.32	2.27	2.30E-02	4.91E-01
ENST00000356994	SCRIB	12.56	0.93	0.48	1.93	5.34E-02	9.96E-01	833.73	-0.14	0.44	-0.33	7.41E-01	1.00E+00
ENST00000409497	SCRN1	13.22	21.34	3.03	7.05	1.85E-12	5.87E-10	65.90	2.50	1.30	1.93	5.41E-02	7.27E-01
ENST00000520168	SDCBP	9.43	20.87	3.03	6.89	5.71E-12	1.35E-09	90.70	0.02	1.45	0.01	9.88E-01	1.00E+00
ENST00000375549	SDHD	3.73	5.44	1.86	2.93	3.36E-03	3.15E-01	7332.72	0.15	0.39	0.40	6.90E-01	1.00E+00
ENST00000528048	SDHD	5.88	6.11	3.03	2.02	4.37E-02	9.96E-01	160.27	-0.96	0.85	-1.13	2.58E-01	1.00E+00
ENST00000404826	SDK1	24.35	1.12	0.35	3.18	1.49E-03	1.67E-01	1909.86	0.37	0.80	0.47	6.41E-01	1.00E+00
ENST00000392476	SEC14L1	4.87	5.84	3.03	1.93	5.41E-02	9.96E-01	568.68	0.55	1.81	0.30	7.61E-01	1.00E+00
ENST00000395310	SEC31A	8.48	6.64	3.03	2.19	2.84E-02	9.96E-01	260.41	1.00	0.70	1.43	1.53E-01	9.70E-01
ENST00000397613	SEPT9	11.67	21.17	3.03	6.99	2.80E-12	7.82E-10	154.14	-0.43	2.04	-0.21	8.34E-01	1.00E+00
ENST00000380739	SERPINB1	22.12	0.78	0.41	1.92	5.42E-02	9.96E-01	1535.96	0.05	0.28	0.17	8.64E-01	1.00E+00
ENST00000409840	SERPINE2	20.27	21.94	3.03	7.24	4.33E-13	2.39E-10	0.00	NA	NA	NA	NA	NA
ENST00000478966	SERPINE2	25.32	22.14	3.03	7.31	2.62E-13	1.76E-10	74.90	0.74	0.49	1.49	1.37E-01	9.50E-01
ENST00000525876	SERPINH1	55.44	0.51	0.27	1.91	5.59E-02	9.96E-01	168.57	-2.35	1.27	-1.85	NA	NA
ENST00000533595	SF3B2	4.69	5.77	2.02	2.86	4.21E-03	3.73E-01	76.51	0.25	0.49	0.52	6.03E-01	1.00E+00
ENST00000507823	SFXN1	12.88	7.24	2.43	2.98	2.86E-03	2.79E-01	290.65	0.01	1.13	0.00	9.96E-01	1.00E+00
ENST00000265735	SGCE	9.03	6.73	3.03	2.22	2.63E-02	9.96E-01	370.03	-0.17	1.61	-0.11	9.15E-01	1.00E+00
ENST00000326317	SGSH	25.49	1.67	0.68	2.47	1.36E-02	8.03E-01	14.17	1.32	0.96	1.38	1.67E-01	9.80E-01
ENST00000361989	SHF	43.88	1.36	0.43	3.13	1.72E-03	1.86E-01	8.82	0.59	1.06	0.56	5.76E-01	1.00E+00
ENST00000379262	SIRT5	4.48	3.03	1.30	2.33	1.97E-02	9.96E-01	756.22	-0.15	0.33	-0.47	6.39E-01	1.00E+00
ENST00000442759	SLAIN1	11.43	7.07	2.38	2.97	2.94E-03	2.85E-01	776.35	0.36	1.23	0.30	7.68E-01	1.00E+00
ENST00000395348	SLC16A9	9.74	1.28	0.50	2.55	1.08E-02	7.06E-01	2113.62	1.00	0.36	2.82	4.74E-03	1.98E-01
ENST00000312221	SLC22A18	10.14	6.89	3.03	2.28	2.29E-02	9.96E-01	615.00	0.44	1.08	0.40	6.86E-01	1.00E+00
ENST00000463571	SLC22A18	4.59	5.76	3.03	1.90	5.76E-02	9.96E-01	187.32	-0.60	0.59	-1.01	3.12E-01	1.00E+00
ENST00000319017	SLC25A20	3.70	5.45	1.66	3.28	1.06E-03	1.24E-01	155.86	0.40	0.90	0.45	6.53E-01	1.00E+00
ENST00000536651	SLC25A26	4.85	5.84	3.03	1.92	5.44E-02	9.96E-01	274.82	0.46	0.55	0.83	4.05E-01	1.00E+00
ENST00000546766	SLC25A3	62.94	5.52	2.30	2.40	1.63E-02	9.02E-01	24495.06	-0.17	0.32	-0.52	6.07E-01	1.00E+00
ENST00000514629	SLC25A36	1.03	3.57	1.85	1.93	5.41E-02	9.96E-01	345.46	0.22	0.64	0.35	7.29E-01	1.00E+00
ENST00000469537	SLC25A44	3.62	3.35	1.78	1.88	5.99E-02	9.96E-01	728.46	-0.64	1.07	-0.60	5.48E-01	1.00E+00
ENST00000472176	SLC29A1	2.13	4.66	2.33	2.00	4.50E-02	9.96E-01	207.48	0.10	0.32	0.31	7.60E-01	1.00E+00
ENST00000529510	SLC37A4	3.58	2.55	1.22	2.09	3.68E-02	9.96E-01	16.11	-0.77	0.93	-0.83	4.08E-01	1.00E+00
ENST00000549258	SLC38A2	42.81	8.97	2.59	3.47	5.21E-04	6.72E-02	3961.66	-0.07	2.04	-0.04	9.71E-01	1.00E+00

ENST00000354884	SLC39A13	0.95	3.52	1.85	1.90	5.76E-02	9.96E-01	15.22	-0.28	2.87	-0.10	9.23E-01	1.00E+00
ENST00000338663	SLC3A2	34.35	3.38	1.56	2.17	3.02E-02	9.96E-01	1131.30	-0.07	0.39	-0.18	8.55E-01	1.00E+00
ENST00000539458	SLC3A2	5.01	5.29	2.17	2.44	1.47E-02	8.48E-01	111.12	0.11	0.51	0.21	8.34E-01	1.00E+00
ENST00000541649	SLC3A2	5.14	3.75	1.02	3.69	2.25E-04	3.11E-02	426.30	1.95	0.49	3.96	7.57E-05	1.07E-02
ENST00000310317	SLC4A2	4.62	5.77	3.03	1.90	5.74E-02	9.96E-01	4734.61	-0.84	1.36	-0.62	5.34E-01	1.00E+00
ENST00000453097	SLC4A8	5.33	4.79	1.39	3.45	5.51E-04	7.04E-02	234.96	-0.32	0.92	-0.35	7.30E-01	1.00E+00
ENST00000454876	SLC6A6	60.07	1.85	0.44	4.20	2.71E-05	4.25E-03	180.11	-24.83	3.91	-6.35	NA	NA
ENST00000504154	SLIT2	6.43	2.28	0.65	3.51	4.53E-04	5.89E-02	323.33	2.00	1.31	1.53	1.27E-01	9.39E-01
ENST00000400286	SLITRK6	8.48	2.24	0.71	3.15	1.65E-03	1.80E-01	929.90	1.83	0.41	4.51	6.54E-06	1.45E-03
ENST00000448276	SMARCD2	6.87	4.29	1.71	2.51	1.21E-02	7.61E-01	8159.49	-0.43	0.45	-0.97	3.33E-01	1.00E+00
ENST00000544865	SMG6	45.47	0.80	0.33	2.45	1.43E-02	8.32E-01	5978.04	-0.07	0.49	-0.14	8.90E-01	1.00E+00
ENST00000532367	SMPD1	7.58	1.29	0.66	1.95	5.17E-02	9.96E-01	21.67	0.44	0.69	0.64	5.23E-01	1.00E+00
ENST00000394986	SNCA	6.70	1.78	0.93	1.92	5.50E-02	9.96E-01	271.06	0.25	0.56	0.44	6.58E-01	1.00E+00
ENST00000447221	SNHG7	10.51	1.92	0.79	2.43	1.52E-02	8.66E-01	399.57	0.25	0.31	0.82	4.15E-01	1.00E+00
ENST00000364432	SNORA65	31.52	0.87	0.33	2.68	7.29E-03	5.53E-01	45.02	-0.51	0.52	-0.97	3.34E-01	1.00E+00
ENST00000390930	SNORD17	162.38	0.95	0.44	2.14	3.24E-02	9.96E-01	6.41	2.01	1.64	1.22	2.22E-01	1.00E+00
ENST00000363091	SNORD1B	40.20	0.96	0.42	2.28	2.23E-02	9.96E-01	5.71	0.82	0.78	1.06	2.91E-01	1.00E+00
ENST00000383926	SNORD29	27.06	1.15	0.53	2.18	2.94E-02	9.96E-01	86.65	-0.42	1.14	-0.37	7.13E-01	1.00E+00
ENST00000390833	SNORD67	2640.14	0.63	0.28	2.23	2.61E-02	9.96E-01	7.71	0.07	0.73	0.10	9.20E-01	1.00E+00
ENST00000363146	SNORD76	40.58	0.86	0.40	2.18	2.89E-02	9.96E-01	83.80	-0.07	1.23	-0.06	9.52E-01	1.00E+00
ENST00000484372	SNRNP200	5.11	5.91	3.03	1.95	5.14E-02	9.96E-01	48.05	-0.15	0.45	-0.33	7.42E-01	1.00E+00
ENST00000560829	SNX1	12.10	7.15	3.03	2.36	1.82E-02	9.56E-01	4712.38	-0.28	0.51	-0.54	5.89E-01	1.00E+00
ENST00000561026	SNX1	2.39	4.83	2.22	2.18	2.96E-02	9.96E-01	37.48	0.71	2.11	0.33	7.38E-01	1.00E+00
ENST00000344780	SNX21	7.01	2.01	1.04	1.93	5.32E-02	9.96E-01	343.41	0.27	0.81	0.33	7.39E-01	1.00E+00
ENST00000270142	SOD1	15.38	4.49	1.94	2.32	2.05E-02	9.96E-01	25509.64	-1.31	0.42	-3.08	2.06E-03	1.16E-01
ENST00000421541	SON	5.04	5.89	3.03	1.94	5.24E-02	9.96E-01	61.39	1.08	0.82	1.31	1.89E-01	9.96E-01
ENST00000431808	SORBS2	24.98	1.17	0.36	3.23	1.25E-03	1.44E-01	16.81	4.75	1.56	3.04	2.35E-03	1.25E-01
ENST00000260197	SORL1	65.24	1.46	0.58	2.51	1.19E-02	7.61E-01	5059.44	0.74	0.47	1.58	1.15E-01	9.24E-01
ENST00000342665	SOX12	23.39	0.95	0.35	2.74	6.22E-03	4.99E-01	5360.17	0.55	0.99	0.55	5.79E-01	1.00E+00
ENST00000510283	SPAG9	6.41	5.65	2.78	2.03	4.26E-02	9.96E-01	508.97	-0.51	0.70	-0.73	4.63E-01	1.00E+00
ENST00000511312	SPAG9	5.43	6.00	3.03	1.98	4.79E-02	9.96E-01	11.66	1.21	0.95	1.26	2.06E-01	1.00E+00
ENST00000521327	SPARC	9.99	6.88	2.34	2.94	3.29E-03	3.11E-01	61.54	1.21	0.79	1.53	1.25E-01	9.38E-01
ENST00000344120	SPRY4	10.33	0.86	0.44	1.94	5.20E-02	9.96E-01	4775.56	0.86	2.16	0.40	6.92E-01	1.00E+00
ENST00000523259	SRA1	5.15	5.31	1.75	3.03	2.42E-03	2.46E-01	1899.18	-0.54	0.60	-0.90	3.69E-01	1.00E+00
ENST00000519259	SREK1	5.60	3.06	1.17	2.62	8.77E-03	6.24E-01	2186.82	0.11	0.87	0.12	9.03E-01	1.00E+00
ENST00000344595	SRR	12.59	1.33	0.62	2.16	3.09E-02	9.96E-01	2653.26	0.30	0.84	0.36	7.18E-01	1.00E+00
ENST00000370950	SRSF11	5.41	5.99	3.03	1.98	4.81E-02	9.96E-01	1744.78	-0.70	0.38	-1.82	6.91E-02	7.98E-01
ENST00000454579	SSFA2	9.05	20.81	3.03	6.87	6.53E-12	1.50E-09	356.24	1.17	1.73	0.68	4.97E-01	1.00E+00
ENST00000491720	SSFA2	7.67	5.90	2.77	2.13	3.29E-02	9.96E-01	3606.95	1.46	1.47	1.00	3.20E-01	1.00E+00
ENST00000319914	ST3GAL1	7.47	1.83	0.56	3.28	1.05E-03	1.24E-01	5.23	-0.38	1.90	-0.20	8.44E-01	1.00E+00
ENST00000268164	ST8SIA2	3.43	2.54	1.10	2.30	2.15E-02	9.96E-01	533.79	1.75	0.63	2.79	5.24E-03	2.10E-01
ENST00000404395	STAT3	13.91	21.41	3.03	7.07	1.57E-12	5.24E-10	308.27	0.70	0.69	1.00	3.17E-01	1.00E+00
ENST00000286713	STOM	342.63	1.27	0.44	2.91	3.61E-03	3.34E-01	7527.01	0.88	0.22	4.01	6.01E-05	9.00E-03
ENST00000531491	STT3A	17.32	21.71	3.03	7.17	7.47E-13	3.40E-10	824.92	0.51	0.45	1.12	2.63E-01	1.00E+00
ENST00000367479	STXBP5	2.69	3.17	1.65	1.92	5.47E-02	9.96E-01	534.06	0.42	0.57	0.74	4.60E-01	1.00E+00
ENST00000433632	SULF2	11.31	7.06	2.38	2.97	3.01E-03	2.89E-01	235.01	0.80	0.40	2.01	4.47E-02	6.72E-01
ENST00000467815	SULF2	13.94	7.36	3.03	2.43	1.51E-02	8.65E-01	5888.71	-0.61	0.53	-1.16	2.45E-01	1.00E+00
ENST00000272902	SUMF1	20.40	0.79	0.39	2.02	4.36E-02	9.96E-01	2867.93	-0.11	0.28	-0.40	6.91E-01	1.00E+00
ENST00000275607	SUMF2	14.18	7.38	2.51	2.94	3.23E-03	3.07E-01	115.06	0.03	0.44	0.08	9.40E-01	1.00E+00
ENST00000320188	SWI5	13.26	2.69	1.21	2.22	2.63E-02	9.96E-01	133.54	-1.06	1.08	-0.98	3.30E-01	1.00E+00
ENST00000328933	SYNGR1	4.57	3.62	1.11	3.25	1.16E-03	1.34E-01	472.80	1.28	0.66	1.92	5.49E-02	7.31E-01
ENST00000485989	TACC3	6.10	20.13	3.03	6.64	3.16E-11	6.06E-09	16.42	0.81	0.70	1.16	2.47E-01	1.00E+00
ENST00000478033	TAGLN2	21.54	7.98	3.03	2.64	8.36E-03	6.07E-01	425.97	-1.50	1.13	-1.33	NA	NA
ENST00000393917	TAGLN3	3.03	5.15	2.32	2.22	2.64E-02	9.96E-01	179.62	2.11	1.53	1.38	1.67E-01	9.80E-01
ENST00000349155	TBX3	13.34	1.98	0.50	3.96	7.38E-05	1.11E-02	2411.30	1.94	0.38	5.13	2.89E-07	9.97E-05
ENST00000548503	TBX3	2.68	4.99	1.70	2.94	3.32E-03	3.13E-01	1.16	0.52	1.60	0.32	7.47E-01	NA
ENST00000360000	TCEAL8	5.53	6.02	3.03	1.98	4.73E-02	9.96E-01	2870.42	-0.39	0.22	-1.75	7.99E-02	8.38E-01
ENST00000415259	TEX264	5.06	5.90	3.03	1.94	5.19E-02	9.96E-01	1187.01	-0.45	0.52	-0.88	3.81E-01	1.00E+00

ENST00000544508	TFAP2C	5.20	5.94	3.03	1.96	5.04E-02	9.96E-01	57.89	0.49	0.66	0.75	4.56E-01	1.00E+00
ENST00000366930	TGFB2	4.53	5.74	3.03	1.89	5.85E-02	9.96E-01	5682.24	0.00	0.54	0.00	1.00E+00	1.00E+00
ENST00000506699	TGFBI	951.47	3.70	1.59	2.33	2.00E-02	9.96E-01	33103.03	-0.23	0.42	-0.55	5.81E-01	1.00E+00
ENST00000527590	THY1	4.79	5.82	3.03	1.92	5.51E-02	9.96E-01	63.03	1.05	1.04	1.01	3.13E-01	1.00E+00
ENST00000374097	TIMM23B	3.94	5.53	2.33	2.37	1.77E-02	9.38E-01	231.29	-1.43	0.74	-1.94	5.26E-02	7.22E-01
ENST00000266085	TIMP3	132.33	0.55	0.23	2.41	1.58E-02	8.85E-01	19862.01	0.13	0.30	0.44	6.59E-01	1.00E+00
ENST00000450814	TKT	27.14	6.15	2.56	2.40	1.63E-02	9.01E-01	1845.75	-0.37	0.52	-0.71	4.77E-01	1.00E+00
ENST00000376520	TLE4	13.49	3.15	1.62	1.94	5.22E-02	9.96E-01	2.11	-0.16	2.25	-0.07	9.45E-01	NA
ENST00000540444	TLN1	10.78	21.05	3.03	6.95	3.67E-12	9.39E-10	4262.35	1.18	2.04	0.58	NA	NA
ENST00000322019	TM6SF1	3.77	5.48	1.60	3.42	6.22E-04	7.80E-02	293.32	1.34	0.71	1.89	5.91E-02	7.54E-01
ENST00000527851	TM7SF2	1.88	3.82	1.94	1.97	4.86E-02	9.96E-01	58.59	0.54	0.80	0.68	4.94E-01	1.00E+00
ENST00000546914	TMBIM6	12.36	21.24	3.03	7.01	2.33E-12	6.86E-10	4.70	0.84	3.77	0.22	8.24E-01	1.00E+00
ENST00000547798	TMBIM6	34.70	3.62	1.36	2.66	7.92E-03	5.87E-01	55558.49	-0.37	0.30	-1.24	2.15E-01	1.00E+00
ENST00000549130	TMBIM6	9.50	6.81	3.03	2.25	2.47E-02	9.96E-01	632.94	-0.79	1.41	-0.56	5.74E-01	1.00E+00
ENST00000552635	TMBIM6	9.21	3.34	1.71	1.95	5.13E-02	9.96E-01	294.81	-0.74	0.59	-1.25	2.12E-01	1.00E+00
ENST00000507723	TMED9	13.53	21.37	3.03	7.06	1.70E-12	5.60E-10	114.96	-0.31	0.71	-0.44	6.58E-01	1.00E+00
ENST00000540112	TMEM132A	4.90	4.65	2.36	1.97	4.89E-02	9.96E-01	1148.40	0.07	0.62	0.12	9.08E-01	1.00E+00
ENST00000569445	TMEM219	2.31	3.00	1.43	2.10	3.56E-02	9.96E-01	35.66	-0.42	0.43	-0.97	3.31E-01	1.00E+00
ENST00000420455	TMEM50B	20.91	2.06	1.05	1.97	4.90E-02	9.96E-01	862.80	0.09	1.77	0.05	9.60E-01	1.00E+00
ENST00000558205	TMEM85	3.68	3.37	1.57	2.15	3.18E-02	9.96E-01	1013.83	-0.09	0.70	-0.13	9.00E-01	1.00E+00
ENST00000380636	TMSB4X	103.86	2.61	0.99	2.63	8.52E-03	6.13E-01	128331.13	-0.18	0.39	-0.47	6.39E-01	1.00E+00
ENST00000341037	TNC	16.06	21.61	3.03	7.14	9.60E-13	3.88E-10	7906.82	1.44	0.37	3.89	9.93E-05	1.31E-02
ENST00000350763	TNC	503.77	1.31	0.27	4.79	1.69E-06	2.89E-04	919.95	1.30	0.57	2.27	2.32E-02	4.92E-01
ENST00000327473	TNFAIP8L1	1.99	4.53	1.68	2.69	7.13E-03	5.46E-01	2900.90	-0.47	0.40	-1.17	2.41E-01	1.00E+00
ENST00000539372	TNFRSF1A	5.13	5.92	3.03	1.95	5.11E-02	9.96E-01	10.15	0.20	0.82	0.24	8.10E-01	1.00E+00
ENST00000296861	TNFRSF21	8.70	1.20	0.61	1.97	4.93E-02	9.96E-01	707.31	1.06	1.32	0.81	4.19E-01	1.00E+00
ENST00000535933	TOM1L2	5.07	5.90	3.03	1.95	5.16E-02	9.96E-01	133.57	1.09	0.67	1.62	1.05E-01	9.05E-01
ENST00000492561	TOMM22	4.66	5.78	3.03	1.90	5.69E-02	9.96E-01	187.30	0.03	0.53	0.05	9.57E-01	1.00E+00
ENST00000413037	TP53I3	4.07	5.59	2.24	2.49	1.27E-02	7.81E-01	146.38	0.18	0.93	0.20	8.42E-01	1.00E+00
ENST00000369750	TPBG	21.88	0.94	0.34	2.74	6.07E-03	4.91E-01	902.29	-2.00	0.93	-2.14	3.24E-02	5.85E-01
ENST00000558072	TPM1	9.93	2.75	1.05	2.62	8.86E-03	6.26E-01	2.31	0.57	1.24	0.46	6.46E-01	NA
ENST00000378292	TPM2	32.28	22.58	3.03	7.46	8.67E-14	8.07E-11	13650.49	-0.42	0.54	-0.77	4.41E-01	1.00E+00
ENST00000428886	TPP1	31.87	5.63	2.13	2.64	8.41E-03	6.09E-01	179.26	-0.51	0.85	-0.60	5.46E-01	1.00E+00
ENST00000300403	TPX2	14.71	20.95	3.03	6.92	4.59E-12	1.10E-09	20291.63	-0.42	0.31	-1.36	1.73E-01	9.85E-01
ENST00000155926	TRIB2	56.24	0.65	0.24	2.71	6.73E-03	5.27E-01	7460.04	0.99	0.90	1.10	2.72E-01	1.00E+00
ENST00000354852	TRIM6-TRIM34	2.12	4.63	2.35	1.97	4.88E-02	9.96E-01	0.99	0.37	3.88	0.10	9.24E-01	NA
ENST00000403663	TRIOBP	8.64	20.68	3.03	6.82	8.85E-12	1.97E-09	489.74	0.26	0.96	0.27	7.87E-01	1.00E+00
ENST00000476941	TRPC1	8.75	6.69	2.38	2.81	4.96E-03	4.16E-01	176.94	0.36	0.88	0.41	6.84E-01	1.00E+00
ENST00000396388	TSEN34	4.68	5.78	2.29	2.53	1.15E-02	7.43E-01	2982.91	0.03	0.75	0.04	9.64E-01	1.00E+00
ENST00000522793	TTC1	6.33	6.22	3.03	2.05	4.02E-02	9.96E-01	507.13	-0.21	0.41	-0.52	6.00E-01	1.00E+00
ENST00000498660	TUBA4A	4.89	5.85	3.03	1.93	5.37E-02	9.96E-01	19.12	-0.61	0.61	-1.00	3.18E-01	1.00E+00
ENST00000374515	TXN	6.07	6.16	3.03	2.03	4.22E-02	9.96E-01	34.81	0.49	1.33	0.37	7.14E-01	1.00E+00
ENST00000217515	TXNL1	75.06	0.58	0.24	2.40	1.63E-02	9.01E-01	4387.44	0.26	0.48	0.54	5.89E-01	1.00E+00
ENST00000427956	TXNRD1	7.18	20.49	3.03	6.76	1.38E-11	2.80E-09	837.14	-0.22	2.03	-0.11	NA	NA
ENST00000246548	UBA2	7.56	6.48	3.03	2.14	3.26E-02	9.96E-01	9017.02	-0.07	0.35	-0.20	8.45E-01	1.00E+00
ENST00000441890	UBAP2L	7.85	20.62	3.03	6.80	1.02E-11	2.18E-09	3.67	0.40	1.22	0.33	7.42E-01	1.00E+00
ENST00000339647	UBC	14.66	2.59	0.99	2.61	9.04E-03	6.32E-01	147.86	-0.20	0.74	-0.26	7.91E-01	1.00E+00
ENST00000220959	UBR5	5.97	6.14	3.03	2.02	4.30E-02	9.96E-01	360.00	1.97	1.70	1.16	2.48E-01	1.00E+00
ENST00000360834	UFD1L	4.57	5.75	3.03	1.90	5.79E-02	9.96E-01	1069.96	-1.15	0.66	-1.74	8.10E-02	8.41E-01
ENST00000367351	ULBP2	18.41	0.73	0.39	1.90	5.76E-02	9.96E-01	1235.24	-0.24	0.36	-0.66	5.08E-01	1.00E+00
ENST00000401406	UQCR10	2.95	2.13	0.98	2.17	2.97E-02	9.96E-01	1239.04	-0.23	0.41	-0.57	5.71E-01	1.00E+00
ENST00000415995	UQCRC1	13.09	21.33	3.03	7.04	1.92E-12	5.99E-10	2963.68	-0.59	1.32	-0.45	NA	NA
ENST00000268379	UQCRC2	206.14	0.61	0.29	2.14	3.26E-02	9.96E-01	7945.08	-0.08	0.34	-0.23	8.18E-01	1.00E+00
ENST00000337003	USMG5	22.06	4.51	2.19	2.07	3.89E-02	9.96E-01	107.51	-0.11	0.69	-0.16	8.71E-01	1.00E+00
ENST00000450251	USP53	22.04	1.39	0.73	1.90	5.74E-02	9.96E-01	1209.40	1.61	1.87	0.86	3.88E-01	1.00E+00
ENST00000498143	USP54	1.39	3.32	1.34	2.47	1.34E-02	7.97E-01	68.33	-0.27	1.45	-0.19	8.51E-01	1.00E+00
ENST00000463829	USP9X	1.85	3.06	1.37	2.23	2.60E-02	9.96E-01	2.84	0.34	1.56	0.22	8.29E-01	1.00E+00
ENST00000302536	VAT1L	25.04	1.28	0.58	2.23	2.60E-02	9.96E-01	3748.96	0.99	0.34	2.88	3.93E-03	1.76E-01

ENST00000371851	VAV2	60.23	0.86	0.25	3.45	5.69E-04	7.25E-02	1460.08	0.48	0.53	0.92	3.60E-01	1.00E+00
ENST00000342785	VCAN	47.90	5.94	1.83	3.25	1.14E-03	1.32E-01	848.16	0.71	2.05	0.34	7.30E-01	1.00E+00
ENST00000537043	VCL	43.22	22.99	3.03	7.59	3.08E-14	4.26E-11	14.18	-0.19	0.66	-0.28	7.77E-01	1.00E+00
ENST00000530983	VPS28	1.36	4.04	2.05	1.97	4.89E-02	9.96E-01	77.44	0.99	0.90	1.10	2.70E-01	1.00E+00
ENST00000333371	VPS33B	4.82	3.76	1.59	2.37	1.79E-02	9.46E-01	200.48	0.24	0.45	0.53	5.93E-01	1.00E+00
ENST00000566354	VPS4A	8.41	1.65	0.77	2.14	3.22E-02	9.96E-01	1.16	0.52	1.60	0.32	7.47E-01	NA
ENST00000238497	VPS4B	28.67	1.71	0.81	2.10	3.57E-02	9.96E-01	2356.97	0.50	0.45	1.10	2.69E-01	1.00E+00
ENST00000354911	WAC	4.49	5.72	3.03	1.89	5.93E-02	9.96E-01	75.10	0.42	0.55	0.76	4.46E-01	1.00E+00
ENST00000554950	WARS	15.45	7.51	3.03	2.48	1.32E-02	7.90E-01	87.41	1.26	2.04	0.62	5.37E-01	1.00E+00
ENST00000558311	WDR61	14.27	21.45	3.03	7.08	1.43E-12	5.02E-10	1858.47	-0.52	0.34	-1.52	1.29E-01	9.42E-01
ENST00000317025	WHSC1L1	9.04	2.61	0.84	3.12	1.80E-03	1.94E-01	264.88	0.50	1.72	0.29	7.72E-01	1.00E+00
ENST00000322238	XPNPEP1	5.13	5.92	3.03	1.95	5.11E-02	9.96E-01	1393.15	-0.68	1.69	-0.40	NA	NA
ENST00000498684	XPNPEP3	6.92	6.34	3.03	2.09	3.65E-02	9.96E-01	3.32	2.96	1.53	1.94	5.29E-02	7.22E-01
ENST00000460284	XRCC5	27.45	20.63	3.03	6.81	9.53E-12	2.07E-09	2763.27	0.25	0.63	0.39	6.99E-01	1.00E+00
ENST00000478828	YARS	16.42	21.64	3.03	7.15	8.89E-13	3.80E-10	331.15	-0.17	0.40	-0.43	6.70E-01	1.00E+00
ENST00000376899	YIF1A	5.71	6.07	3.03	2.00	4.52E-02	9.96E-01	706.67	-0.04	0.30	-0.13	9.00E-01	1.00E+00
ENST00000372839	YWHAB	43.82	3.71	1.42	2.61	9.13E-03	6.32E-01	15215.33	0.03	0.80	0.04	9.66E-01	1.00E+00
ENST00000479421	YWHAB	17.19	21.71	3.03	7.17	7.60E-13	3.42E-10	72.13	0.03	0.81	0.04	9.72E-01	1.00E+00
ENST00000381844	YWHAQ	93.79	4.10	0.88	4.66	3.12E-06	5.21E-04	754.21	-0.23	0.57	-0.40	6.91E-01	1.00E+00
ENST00000554579	YY1	5.76	2.25	1.11	2.03	4.28E-02	9.96E-01	56.72	-2.14	0.91	-2.35	1.86E-02	4.39E-01
ENST00000322342	ZADH2	12.85	0.99	0.42	2.37	1.78E-02	9.41E-01	3222.92	0.39	0.26	1.50	1.33E-01	9.47E-01
ENST00000471652	ZC3HAV1	9.26	20.35	3.03	6.72	1.86E-11	3.68E-09	349.90	-0.80	0.78	-1.02	3.08E-01	1.00E+00
ENST00000525490	ZDHHC13	5.71	3.13	1.38	2.27	2.31E-02	9.96E-01	1458.79	-0.19	0.91	-0.21	8.32E-01	1.00E+00
ENST00000301318	ZFP28	2.55	2.69	1.21	2.23	2.59E-02	9.96E-01	817.17	0.89	0.38	2.37	1.79E-02	4.31E-01
ENST00000505204	ZFR	1.99	3.27	1.66	1.97	4.91E-02	9.96E-01	12.42	0.02	0.96	0.02	9.81E-01	1.00E+00
ENST00000309060	ZHX3	25.81	1.35	0.61	2.20	2.77E-02	9.96E-01	483.13	8.20	3.75	2.19	NA	NA
ENST00000373988	ZMYM3	3.40	3.25	1.68	1.93	5.33E-02	9.96E-01	5.84	-0.75	3.77	-0.20	8.43E-01	1.00E+00
ENST00000464860	ZNF136	3.25	1.78	0.83	2.16	3.11E-02	9.96E-01	205.90	0.45	0.66	0.68	4.98E-01	1.00E+00
ENST00000532305	ZNF143	3.49	2.87	1.44	1.99	4.65E-02	9.96E-01	154.11	-0.57	1.37	-0.42	6.77E-01	1.00E+00
ENST00000448715	ZNF181	1.39	3.32	1.63	2.03	4.19E-02	9.96E-01	574.47	-0.08	0.81	-0.10	9.24E-01	1.00E+00
ENST00000427606	ZNF252	2.85	5.07	2.29	2.22	2.66E-02	9.96E-01	217.36	0.04	0.39	0.11	9.16E-01	1.00E+00
ENST00000509196	ZNF302	9.62	1.21	0.61	1.98	4.83E-02	9.96E-01	469.90	0.18	0.68	0.27	7.90E-01	1.00E+00
ENST00000262990	ZNF330	14.40	0.77	0.41	1.88	5.98E-02	9.96E-01	1491.20	0.15	0.46	0.31	7.54E-01	1.00E+00
ENST00000391709	ZNF470	2.03	2.26	1.15	1.96	4.99E-02	9.96E-01	529.33	0.46	0.48	0.97	3.34E-01	1.00E+00
ENST00000357666	ZNF816	2.89	2.18	0.97	2.24	2.48E-02	9.96E-01	51.21	0.99	1.01	0.97	3.30E-01	1.00E+00
ENST00000396105	ZNFX1	64.39	0.99	0.30	3.25	1.16E-03	1.34E-01	4522.14	0.24	0.47	0.51	6.08E-01	1.00E+00
ENST00000485387	ZNHIT1	29.00	0.95	0.46	2.06	3.96E-02	9.96E-01	2536.63	-0.31	0.40	-0.77	4.42E-01	1.00E+00
ENST00000449423	ZYX	12.97	21.32	3.03	7.04	1.96E-12	6.02E-10	3012.89	-0.59	0.78	-0.75	4.54E-01	1.00E+00

Table S5. Association between prognostic factors and NSUN5 methylation status in the TCGA dataset and validation cohorts of glioma patients. Related to Figure 7.

CHARACTERISTICS	Glioma TCGA dataset	NSUN5 methylation (N = 497)			Glioma initial validation cohort	NSUN5 methylation (N = 115)			Glioma expanded validation cohort	NSUN5 methylation (N = 418)		
	**N (%)**	**Unmethylated** N (%)	**Methylated** N (%)	**P***	**N (%)**	**Unmethylated** N (%)	**Methylated** N (%)	**P***	**N (%)**	**Unmethylated** N (%)	**Methylated** N (%)	**P***
**Age (years)**												
<50	282 (56%)	192 (68%)	90 (32%)	0.090	47 (41%)	30 (64%)	17 (36%)	0.052	142 (34%)	30 (21%)	112 (79%)	$<10^{-5}$***
>50	213 (43%)	160 (75%)	53 (25%)		68 (59%)	55 (81%)	13 (19%)		105 (25%)	55 (52%)	50 (48%)	
Unknown	2 (1%)	0 (0%)	2 (100%)		0 (0%)	0 (0%)	0 (0%)		171 (41%)	113 (66%)	58 (34%)	
**Gender**												
Male	267 (54%)	187 (70%)	80 (30%)	0.074	68 (59%)	51 (75%)	17 (25%)	0.830	186 (44%)	51 (27%)	135 (73%)	$<10^{-5}$***
Female	228 (45%)	165 (73%)	63 (27%)		47 (41%)	34 (72%)	13 (28%)		61 (15%)	34 (56%)	27 (44%)	
Unknown	2 (1%)	0 (0%)	2 (100%)		0 (0%)	0 (0%)	0 (0%)		171 (41%)	113 (66%)	58 (34%)	
**Grade of glioma**												
Low-grade glioma	374 (75%)	237 (63%)	137 (37%)	$<10^{-5}$***	41 (36%)	25 (61%)	16 (39%)	0.026*	183 (44%)	74 (40%)	109 (60%)	0.014*
Glioblastoma	123 (25%)	115 (93%)	8 (7%)		74 (64%)	60 (81%)	14 (19%)		235 (56%)	124 (53%)	111 (47%)	
**Treatment**												
Radiotherapy	221 (44%)	140 (63%)	81 (37%)	$<10^{-5}$***	8 (7%)	4 (50%)	4 (50%)	0.187	77 (18%)	4 (5%)	73 (95%)	$<10^{-5}$***
Temozolamide	9 (2.5%)	9 (100%)	0 (0%)		49 (43%)	39 (70%)	10 (30%)		112 (27%)	39 (35%)	73 (65%)	
Radiotherapy + Temozolamide	82 (16%)	74 (90%)	8 (10%)		33 (29%)	24 (73%)	9 (27%)		33 (8%)	24 (73%)	9 (27%)	
Others	9 (2.5%)	9 (100%)	0 (0%)		11 (9%)	6 (54%)	5 (46%)		11 (3%)	6 (54%)	5 (46%)	
No treatment	169 (34%)	113 (67%)	56 (33%)		6 (5%)	6 (100%)	0 (0%)		6 (1%)	6 (100%)	0 (0%)	
Unknown	7 (1%)	7 (100%)	0 (0%)		8 (7%)	6 (75%)	2 (25%)		179 (43%)	119 (66%)	60 (34%)	
**IDH1 mutational status**												
Wild-type	203 (41%)	187 (92%)	16 (8%)	$<10^{-5}$***	54 (47%)	47 (87%)	7 (13%)	10^{-4}***	237 (57%)	160 (67%)	77 (33%)	$<10^{-5}$***
Mutated	294 (50%)	165 (56%)	129 (44%)		29 (25%)	13 (45%)	16 (55%)		149 (36%)	13 (9%)	136 (91%)	
Unknown	0 (0%)	0 (0%)	0 (0%)		32 (28%)	25 (78%)	7 (22%)		32 (7%)	25 (78%)	7 (22%)	
**Co-deletion 1p/19q**												
No co-deletion	349 (65%)	261 (75%)	88 (25%)	0.002**	17 (15%)	10 (59%)	7 (41%)	0.999	280 (67%)	123 (44%)	157 (56%)	$<10^{-5}$***
Co-deletion 1p/19q	131 (26%)	77 (59%)	54 (41%)		16 (14%)	9 (56%)	7 (44%)		56 (13%)	9 (16%)	47 (84%)	
Unknown	17 (4%)	14 (82%)	3 (18%)		82 (71%)	66 (80%)	16 (20%)		82 (71%)	66 (80%)	16 (20%)	
**MGMT methylation status**												
Unmethylated	240 (48%)	194 (81%)	46 (19%)	$<10^{-5}$***	25 (22%)	19 (76%)	6 (24%)	0.432	165 (39%)	116 (70%)	49 (30%)	$<10^{-5}$***
Methylated	257 (52%)	158 (61%)	99 (39%)		56 (49%)	45 (80%)	11 (37%)		253 (61%)	82 (32%)	171 (68%)	
Unknown	0 (0%)	0 (0%)	0 (0%)		34 (29%)	21 (62%)	13 (38%)					
**gCIMP phenotype**												
No gCIMP	331 (67%)	226 (68%)	105 (32%)	0.094	-	-	-	-	-	-	-	-
gCIMP	166 (33%)	126 (76%)	40 (24%)		-	-	-		-	-	-	

P-value represents Fisher's exact test or Chi-square function whenever required: statistical significance under 0.05, 0.01** and 0.001***

Table S6. Association between different prognostic co-variables in glioma TCGA dataset and validation cohort. Related to Figure 7.

	Glioma TCGA dataset						Glioma VALIDATION cohort					
	**IDH1 mutation**			**MGMT methylation**			**IDH1 mutation**			**MGMT methylation**		
**IDH1 mutation**	Wild-type N (%)	Mutated N (%)	P*	Unmethylated N (%)	Methylated N (%)	P*	Wild-type N (%)	Mutated N (%)	P*	Unmethylated N (%)	Methylated N (%)	P*
Wild-type	-	-	-	129 (54%)	74 (29%)	$< 10^{-5}$	-	-	-	130 (87%)	107 (45%)	$< 10^{-5}$
Mutated	-	-		111 (46%)	183 (71%)		-	-		19 (13%)	130 (55%)	
**Co-deletion 1p/19q**												
No co-deletion	172 (91%)	177 (61%)	$< 10^{-5}$	206 (90%)	143 (57%)	$< 10^{-5}$	187 (99%)	93 (65%)	$< 10^{-5}$	119 (95%)	161 (76%)	$< 10^{-5}$
Co-deletion 1p/19q	16 (9%)	115 (39%)		23 (10%)	108 (43%)		2 (1%)	51 (35%)		6 (5%)	50 (24%)	

*P-value represents Fisher's exact test: statistical significance under 0.05

Key Resources Table

REAGENT or RESOURCE	SOURCE	IDENTIFIER
**Antibodies**		
Rabbit polyclonal anti-NSUN1 (NOP2)	ThermoFisher	Cat# PA5-34712
Rabbit polyclonal anti-NSUN4	ThermoFisher	Cat# PA5-31695
Rabbit polyclonal anti-NSUN5	Abcam	Cat# ab121633
Mouse monoclonal anti-NQO1	Cell signaling	Cat# 3187
Rabbit polyclonal anti-IDH1	Cell signaling	Cat# 3997
Rabbit polyclonal anti-TET1	Novus Biologicals	Cat# NBP2-19290
Rabbit polyclonal anti-TET3	Abcam	Cat# ab139311
Rabbit polyclonal anti-KIDINS220	Novus Biologicals	Cat# NBP1-31216
Rabbit polyclonal anti-NDRG3	Cell signaling	Cat# 5846
Mouse monoclonal anti-ACO2	ThermoFisher	Cat# MA1-029
Monoclonal anti-β-Actin	Sigma	Cat# A3854
Rabbit polyclonal anti-Lamin B1	Abcam	Cat# ab16048
Mouse monoclonal anti-αTubulin	Abcam	Cat# ab40742
**Cell lines**		
DBTRG-05MG	American Type Culture Collection	Cat# CRL-2020™
M059J	American Type Culture Collection	Cat# CRL-2366™
CAS-1	The Biological Bank of the IRCCS	Cat# ICLC HTL97009
A172	American Type Culture Collection	Cat# CRL-1620™
LN229	American Type Culture Collection	Cat# CRL-2611™
KS-1	Japanese Collection of Research Bioresources Cell Bank	Cat# IFO50436
SW1088	American Type Culture Collection	Cat# HTB-12™
BT142 mut/-	American Type Culture Collection	Cat# ACS-1018™
MOG-G-CCM	Sigma	Cat# 86022702-1VL
**Oligonucleotides**		
**Q-PCR**		
NSUN5 Fd	CAAGGTTTCTCCTATCAGGGTC	
NSUN5 Rv	AGGCAAAGATCTTCCCTTGGT	
NQO1 Fd	AAAGGACCCTTCCGGAGTAA	
NQO1 Rv	CCATCCTTCCAGGATTTGAA	
GAPDH Fd	TGCACCACCAACTGCTTAGC	
GAPDH Rv	GGCATGGACTGTGGTCATGAG	
**Cloning**		
NSUN5 Cloning Fd	AAAAAAAAGAATTCGCCGCCACCATGGGGCTGTATGCTGCAGCTGCAGGCG TGTTGGCCGGCGTGGAGAGCCGCCAGGGCTCTATCAAGGGGT	
NSUN5 Cloning Rv	AAAAAAAATCTAGATCACTTATCGTCGTCATCCTTGTAATCGCCGGAGCCCCTTG GCACCTCGACCCGTTCAATTACAGCAACGAAGAAGCCACTGCTGAGTGTGGTCTC	
NSUN5 mut Cloning Fd	AAAAAAAAGAATTCGCCGCCACCATGGGGCTGTATGCTGCAGCTGCAGGCGTGT TGGCCGGCGTGGAGAGCC	
NSUN5 mut Cloning Rv	AAAAAAAACTAGTTCAGGCGTAGTCGGGCACGTCGTAGGGGTAGCCGCTGCCGCC CGGAGCCTCTGCTATGTGCAAGGCGGTGTGCAAGCACCGGCTGCGGCTCTTTGCT	
NSUN5 mut PCR fusion A Fd	ATGGGGCTGTATGCTGCAGCTG	
NSUN5 mut PCR fusion A Rv	CATGGATGCCAGCAGCTTGGCATCCAGCAGAAAGGCAAAGATCTTCCCTTGGTTCT TCAGAAGAGCAGCCAAGTGACTGGTCTTATTGCC	

NSUN5 mut PCR fusion B Fd	GATCTTTGCCTTTCTGCTGGATGCCAAGCTGCTGGCATCCATGGCCACGCTGCTGG CCCGGGCTGGCGTCTCTTGCTGTGAACTGGCTGAG
NSUN5 mut PCR fusion B Rv	TGCAGGAAGGCAGCAGCAGGATGTAGTGGACCTCATGGTAGCGTGG
NSUN5 mut PCR fusion C Fd	CATCCTGCTGCTGCCTTCCTGCAGTGGCTCGGGTATGCCGAGC
NSUN5 mut PCR fusion C Rv	GGCAGAGGGACACCGTGGAGTAGACGAGCCGCTGCAGGGAAGGG
NSUN5 mut PCR fusion D Fd	GTCTACTCCACGGTGTCCCTCTGCCAGGAGGAGAATGAAGACGTGGTGC
NSUN5 mut PCR fusion D Rv	tttttttACTAGTTCACTTgTCGTCGTCgTCCTTGTAgTCgCCgctgCCGCCCCGGAGCCTCTG CTATGTGCAAGGCGGTGTGCAAGCACCGGCTGCGGCTCTTTGCTGT
**DNA bisulfite sequencing PCR**	
NSUN5 BSP Fd	TAAAAATTTAAGAATTAGAGAAGAAGTGG
NSUN5 BSP Rv	AAAACAAACTAAATAAAAACTCCCC
GAPDH BSP Fd	TTTTTAAAGTTTTTTTGTTTTATTTAAG
GAPDH BSP Rv	CCCAAAATCTTAAAACCTAAACTAC
OOEP BSP Fd	TGGAGATTTAATAGGAGGTTAAATGAATA
OOEP BSP Rv	AATAAAACACCAAAAAATCTCTCAATT
**RNA bisulfite sequencing PCR**	
NSUN5 C3782 28S rRNA Fd	GTGATTTTTGTTTAGTGTTTTGAATGTTAA
NSUN5 C3782 28S rRNA Rv	AAAACCTCCCACTTATTCTACACCTCTC
NSUN1 C4447 28S rRNA Fd	TAGTGTTAGGTGGGGAGTTTGATTG
NSUN1 C4447 28S rRNA Rv	AAATTCTACTTCACAATAATAAAAAAAACC
**ShRNA**	
NSUN5 ShRNA 1 Fd	gatccGCTCCGATGATGTAGTTGATTTCAAGAGAA TCAACTACATCATCGGAGTTTTTTACGCGTg
NSUN5 ShRNA 1 Rv	aattcACGCGTAAAAAACTCCGATGATGTAGTTGAT TCTCTTGAAATCAACTACATCATCGGAGCg
NSUN5 ShRNA 2 Fd	gatccGCCATGAGGTCCACTACATCTTCAAGAGAG ATGTAGTGGACCTCATGGTTTTTTACGCGTg
NSUN5 ShRNA 2 Rv	aattcACGCGTAAAAAACCATGAGGTCCACTACATC TCTCTTGAAGATGTAGTGGACCTCATGGCg
NSUN5 ShRNA 3 Fd	gatccGCCGATGATGTAGTTGATTATTCAAGAGATA ATCAACTACATCATCGGTTTTTTACGCGTg
NSUN5 ShRNA 3 Rv	aattcACGCGTAAAAAACCGATGATGTAGTTGATTA TCTCTTGAATAATCAACTACATCATCGGCg
NSUN5 ShRNA 4 Fd	gatccGTGCTAGTGTATGAGTTGTTTCAAGAGAACA ACTCATACACTAGCACTTTTTTACGCGTg
NSUN5 ShRNA 4 Rv	attcACGCGTAAAAAAGTGCTAGTGTATGAGTTGTT CTCTTGAAACAACTCATACACTAGCACg
**Pyrosequencing**	
NSUN5 PCR Pyroseq Fd	Biot-ATGGGGTTGTATGTTGTAGTTG
NSUN5 PCR Pyroseq Rv	CCCACCCCRAATTCCTCACT
NSUN5 SeqS1 Pyroseq Rv	TCCCCACCCCTACTA
NSUN5 SeqS2 Pyroseq Rv	CCCTTAATAAAACCC
**Deposited Data**	
RNA-Seq: BioProject: PRJNA395552	https://dataview.ncbi.nlm.nih.gov/object/PRJNA395552?reviewer=60ndb08i1g6na5nkoaaiad9a6o
bsRNA-Seq: BioProject: PRJNA395575	https://www.ncbi.nlm.nih.gov/bioproject/395575
Ribo-Seq: BioProject: PRJNA395570	https://www.ncbi.nlm.nih.gov/bioproject/395570
